# Shared and Independent Genetic Basis of Resistance to Bt Toxin Cry2Ab in Two Strains of Pink Bollworm

**DOI:** 10.1038/s41598-020-64811-w

**Published:** 2020-05-14

**Authors:** Jeffrey A. Fabrick, Dannialle M. LeRoy, Gopalan C. Unnithan, Alex J. Yelich, Yves Carrière, Xianchun Li, Bruce E. Tabashnik

**Affiliations:** 1 USDA ARS, U.S. Arid Land Agricultural Research Center, Maricopa, AZ 85138 USA; 2 Department of Entomology, University of Arizona, Tucson, AZ 85721 USA

**Keywords:** Agricultural genetics, Mutation, RNA splicing, Evolutionary genetics, Plant biotechnology

## Abstract

Evolution of pest resistance threatens the benefits of crops genetically engineered to produce insecticidal proteins from *Bacillus thuringiensis* (Bt). Field populations of the pink bollworm (*Pectinophora gossypiella*), a global pest of cotton, have evolved practical resistance to transgenic cotton producing Bt toxin Cry2Ab in India, but not in the United States. Previous results show that recessive mutations disrupting an autosomal ATP-binding cassette gene (*PgABCA2*) are associated with pink bollworm resistance to Cry2Ab in field-selected populations from India and in one lab-selected strain from the United States (Bt4-R2). Here we discovered that an independently derived, lab-selected Cry2Ab-resistant pink bollworm strain from the United States (BX-R) also harbors mutations that disrupt *PgABCA2*. Premature stop codons introduced by mis-splicing of *PgABCA2* pre-mRNA were prevalent in field-selected larvae from India and in both lab-selected strains. The most common mutation in field-selected larvae from India was also detected in both lab-selected strains. Results from interstrain crosses indicate BX-R has at least one additional mechanism of resistance to Cry2Ab that does not involve *PgABCA2* and is not completely recessive or autosomal. We conclude that recessive mutations disrupting *PgABCA2* are the primary, but not the only, mechanism of resistance to Cry2Ab in pink bollworm.

## Introduction

Crops genetically engineered to produce insecticidal proteins from the bacterium *Bacillus thuringiensis* (Bt) have revolutionized management of some of the world’s most devastating pests^1^. In 2018, millions of farmers in 15 countries grew more than 104 million hectares of transgenic Bt crops^2^. Bt crops can suppress pests, increase yield and farmer profits, and decrease conventional insecticide use, which translates to health and environmental benefits^1,3–7^. However, evolution of resistance to Bt crops by pests reduces such benefits^8–11^. Practical resistance to Bt crops, which is field-evolved resistance that has practical consequences for pest management, has been documented in at least nine species of major pests targeted by Bt crops^8–11^.

Better understanding of the mechanisms and genetic basis of resistance to Bt toxins is needed to monitor, manage, and counter pest resistance. For Cry1 toxins, the first family of lepidopteran-active crystalline (Cry) Bt toxins deployed widely in transgenic crops, the molecular genetic basis of resistance has been studied extensively^12–16^. However, much less is known about this issue for Cry2 toxins, the second such family. While each of the first Bt crops produced a single toxin from the Cry1 family (e.g., Cry1Ac), most Bt crops grown now produce Cry2Ab in combination with one or more Cry1 toxins^17^. For lab-selected strains of the lepidopteran pests *Helicoverpa armigera* and *Helicoverpa punctigera*, resistance to Cry2Ab is associated with reduced binding to larval midgut membranes and linked with autosomal recessive mutations that disrupt the ATP-binding cassette protein ABCA2^18,19^. Deleting portions of the gene encoding ABCA2 caused resistance to Cry2Ab in *H. armigera, Trichoplusia ni*, and *Bombyx mori*^20–22^.

Related work with pink bollworm (*Pectinophora gossypiella*), one of the world’s most damaging pests of cotton^23,24^, has provided insights into its lab- and field-selected resistance to Cry2Ab^25–28^. The sustained susceptibility to Bt cotton producing Cry1Ac and Cry2Ab was essential for the recent declaration of eradication of pink bollworm from the continental United States^9,29,30^. Also, populations in China remain susceptible to the Cry1Ac-producing cotton grown there^9,31,32^. By contrast, pink bollworm in India rapidly evolved resistance to Cry1Ac and Cry2Ab produced by Bt cotton^33–35^. Our previous work revealed that mutations disrupting *PgABCA2*, the gene encoding the pink bollworm ABCA2 protein, are associated with resistance to Cry2Ab in field-selected populations from India and in Bt4-R2, a lab-selected strain from the United States^28^. We previously reported that resistance to Cry2Ab was autosomal and recessive in the Bt4-R2 strain^27,28^ and in BX-R, an independently derived lab-selected, strain from the United States^25,26^.

Here we tested the hypothesis that resistance to Cry2Ab in BX-R is associated with mutations in *PgABCA2*. We conducted interstrain complementation tests for allelism between BX-R and Bt4-R2, and analyzed *PgABCA2* cDNA and gDNA from BX-R. We discovered that BX-R harbors disruptive mutations in *PgABCA2* cDNA, including many novel mutations as well as one reported previously from both India and Bt4-R2, and another reported only from India. The new results also provide evidence that BX-R has a second mechanism of resistance to Cry2Ab with inheritance that is not autosomal or recessive.

## Results

### Dominance, Sex Linkage and Maternal Effects

**Figure 1:**
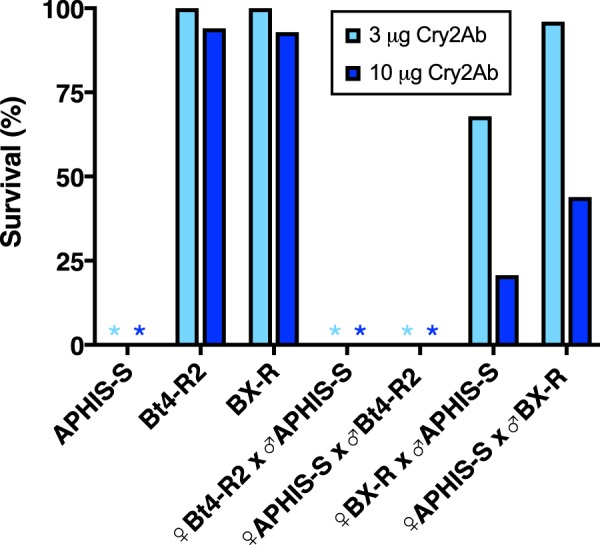
Survival of pink bollworm larvae from two resistant strains (Bt4-R2 and BX-R), a susceptible strain (APHIS-S), and their F_1_ progeny from mass crosses. Asterisks indicate 0% survival for APHIS-S and the F_1_ progeny from the two reciprocal mass crosses between Bt4-R2 and APHIS-S. Light blue: 3 μg Cry2Ab per mL diet (n = 32 larvae per bar or asterisk). Dark blue: 10 μg Cry2Ab per mL diet (n = 48 per bar or asterisk).

In the first and second set of bioassays, respectively, survival at 10 μg Cry2Ab per mL diet was 95.7 and 92.3% for Bt4-R2 (mean = 94.0%), 100 and 85.7% for BX-R (mean = 92.9%), and 0% (both sets) for the susceptible strain APHIS-S (Fig. 1). At 3 μg Cry2Ab per mL diet, which was tested only in the first set of bioassays, survival was 100% for both resistant strains and 0% for APHIS-S (Fig. 1).

Survival of F_1_ progeny from mass crosses between Bt4-R2 and APHIS-S was 0% at 3 and 10 μg Cry2Ab per mL diet for both reciprocal crosses, indicating resistance was autosomal and completely recessive (*h* = 0 at both concentrations), with sex linkage and maternal effects not evident (Fig. 1). By contrast, BX-R resistance to Cry2Ab was not entirely autosomal or recessive. In the first and second set of bioassays, respectively, at 10 μg Cry2Ab per mL diet, survival of F_1_ progeny from mass crosses was 21.4 and 20.0% for ♀ BX-R X ♂ APHIS-S (mean = 20.7%) and 44.0 and 43.8% for ♀ APHIS-S X ♂ BX-R (mean = 43.9%, Fig. 1). At 3 μg Cry2Ab per mL diet, survival of F_1_ progeny from mass crosses was 67.9% for ♀ BX-R X ♂ APHIS-S and 96.0% for ♀ APHIS-S X ♂ BX-R (Fig. 1). In all three pairwise comparisons between reciprocal crosses (first and second bioassays at 10 μg Cry2Ab per mL diet and first bioassay at 3 μg Cry2Ab per mL diet), survival was higher for ♀ APHIS-S X ♂ BX-R than for ♀ BX-R X ♂ APHIS-S (mean difference = 25%, SE = 2%, paired t-test, *t* = 14.9, *df* = 2, *P* = 0.0045).

The higher survival on treated diet of F_1_ progeny from crosses with BX-R fathers versus BX-R mothers is not consistent with maternally inherited resistance, which would be expected to cause the opposite pattern. Although the observed pattern of survival on treated diet is consistent with sex-linked inheritance of resistance, the sex ratio of the survivors is not. Under sex-linked inheritance of resistance, an excess of sons is expected in the F_1_ survivors on treated diet from crosses with BX-R mothers relative to BX-R fathers (see Methods). However, the proportion of males was not higher for the F_1_ survivors from crosses with BX-R mothers (0.54, 7 of 13) than BX-R fathers (0.85, 17 of 20). On untreated diet, the proportion of F_1_ males did not differ from the expected 0.50 for the crosses with BX-R fathers (0.50, 4 of 8) or mothers (5 of 8, 0.63, Fisher’s exact test, P = 1.0 for each type of cross). In this experiment, the proportion of F_1_ males on untreated diet pooled for both types of cross (0.56, 9 of 16) was similar to the mean proportion of males on non-Bt cotton in previous studies (0.55, SE = 0.005, n = 4 studies^36–39^).

Based on the mean survival of F_1_ progeny at 10 μg Cry2Ab per mL diet from the first and second bioassays, the dominance parameter *h* was 0.22 for the crosses with mothers from BX-R and 0.47 for the crosses with fathers from BX-R (mean = 0.35). Based on survival at 3 μg Cry2Ab per mL diet, *h* was 0.68 for crosses with BX-R mothers and 0.96 for crosses with BX-R fathers (mean = 0.82). Because *h* varies from 0 for completely recessive resistance to 1 for completely dominant resistance, these results show that BX-R resistance to Cry2Ab was not completely recessive at either concentration and was close to completely dominant for crosses with BX-R fathers at 3 μg Cry2Ab per mL diet.

### Interstrain Complementation Test for Allelism

**Figure 2:**
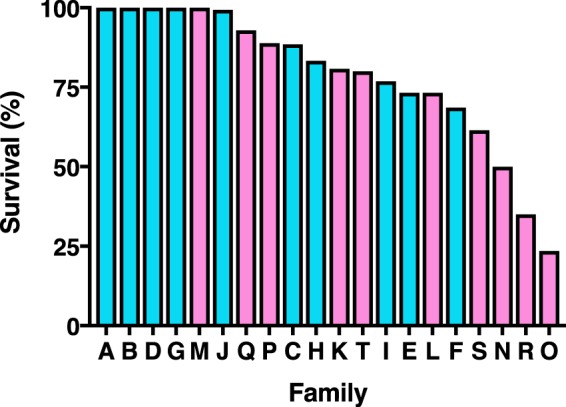
Survival of pink bollworm larvae from single-pair crosses between resistant strains Bt4-R2 and BX-R tested at 10 μg Cry2Ab per mL diet. Families A-J (blue) from female Bt4-R2 X male BX-R and K-T (pink) from female BX-R X male Bt4-R2.

In the F_1_ progeny of 20 families from single-pair crosses between BX-R and Bt4-R2, mean survival at 10 μg Cry2Ab per mL diet was 78.8% (SE = 5.0%), which differs significantly from the 0% survival expected if resistance in both strains was recessive and controlled entirely by loci that differed between strains (one-sample t-test, *t* = 15.9, *df* = 19, *P* < 0.0001). The striking heterogeneity in survival among the 20 F_1_ families (range = 24 to 100%, Fig. 2) from crosses between BX-R and Bt4-R2 indicates genetic variation affecting resistance occurred within the strains, between the strains, or both. Because resistance was not completely dominant at this concentration in either strain (*h* = 0 in Bt4-R2 and mean *h* = 0.35 in BX-R), the results showing six F_1_ families with survival of 99.4% (J) or 100.0% (A, B, D, G, and M) imply that at the same locus in both strains, neither parent for these families had alleles conferring susceptibility to Cry2Ab. Conversely, survival of <70% in five families (including 24% in family O, 35% in R, and 50% in N) indicates that in these families, the parents from BX-R and  Bt4-R2 did not share a locus where both parents had no alleles conferring susceptibility.

Consistent with the results described above showing that inheritance of resistance for F_1_ progeny from mass crosses between BX-R and APHIS-S was not entirely autosomal, mean survival was 20% higher for the 10 families with the father from BX-R (A-J: 89%, SE = 4%) than for the 10 families with the mother from BX-R (K-T: 69%, SE = 8.1%, t-test, *t* = 2.3, *df* = 9, *P* = 0.041, Fig. 2). Relative to the crosses between BX-R and APHIS-S, mean survival in the crosses between BX-R and Bt4-R2 was 45% higher for the families with a BX-R father (one-sample t-test, *t* = 11.3, *df* = 9, *P* < 0.0001) and 47% higher for the crosses with the mother from BX-R (one-sample t-test, *t* = 5.9, *df* = 9, *P* = 0.0002). The significantly higher survival in F_1_ progeny from crosses between BX-R and Bt4-R2 relative to crosses between BX-R and APHIS-S confirms the two resistant strains shared at least one locus harboring one or more alleles conferring resistance to Cry2Ab.

### PCR Screening of gDNA from BX-R for Mutation *r*_*A1*_ of *PgABCA2*

Because previous work identified mutations in *PgABCA2* linked with resistance to Cry2Ab in Bt4-R2^28^, we hypothesized that this is the shared locus where resistance mutations also occur in some individuals from BX-R. In particular, we previously found that mutation *r*_*A1*_, which introduces a premature stop codon in *PgABCA2*, occurred in all gDNA screened from Bt4-R2 and in none from APHIS-S^28^. Results in the present study using allele-specific PCR conducted simultaneously for the three strains (n = 10 individuals and 20 alleles per strain) confirmed that the *r*_*A1*_ mutation was fixed in Bt4-R2 (no wild-type) and absent from APHIS-S. The results here also revealed *r*_*A1*_ was absent from BX-R. Moreover, in 59 F_1_ survivors on 10 μg Cry2Ab per mL diet from 20 BX-R X Bt4-R2 single-pair families, every individual screened had one *PgABCA2* allele with the *r*_*A1*_ mutation. Because *r*_*A1*_ was detected in all gDNA screened from Bt4-R2 and none from BX-R, we infer that the single allele containing *r*_*A1*_ in each of the 59 F_1_ survivors came from the Bt4-R2 parent. These results show that *r*_*A1*_ does not confer resistance to Cry2Ab in BX-R, but they do not exclude contributions to Cry2Ab resistance in BX-R from other mutations in *PgABCA2*.

### Mutations in *PgABCA2* cDNA and gDNA from BX-R

**Figure 3:**
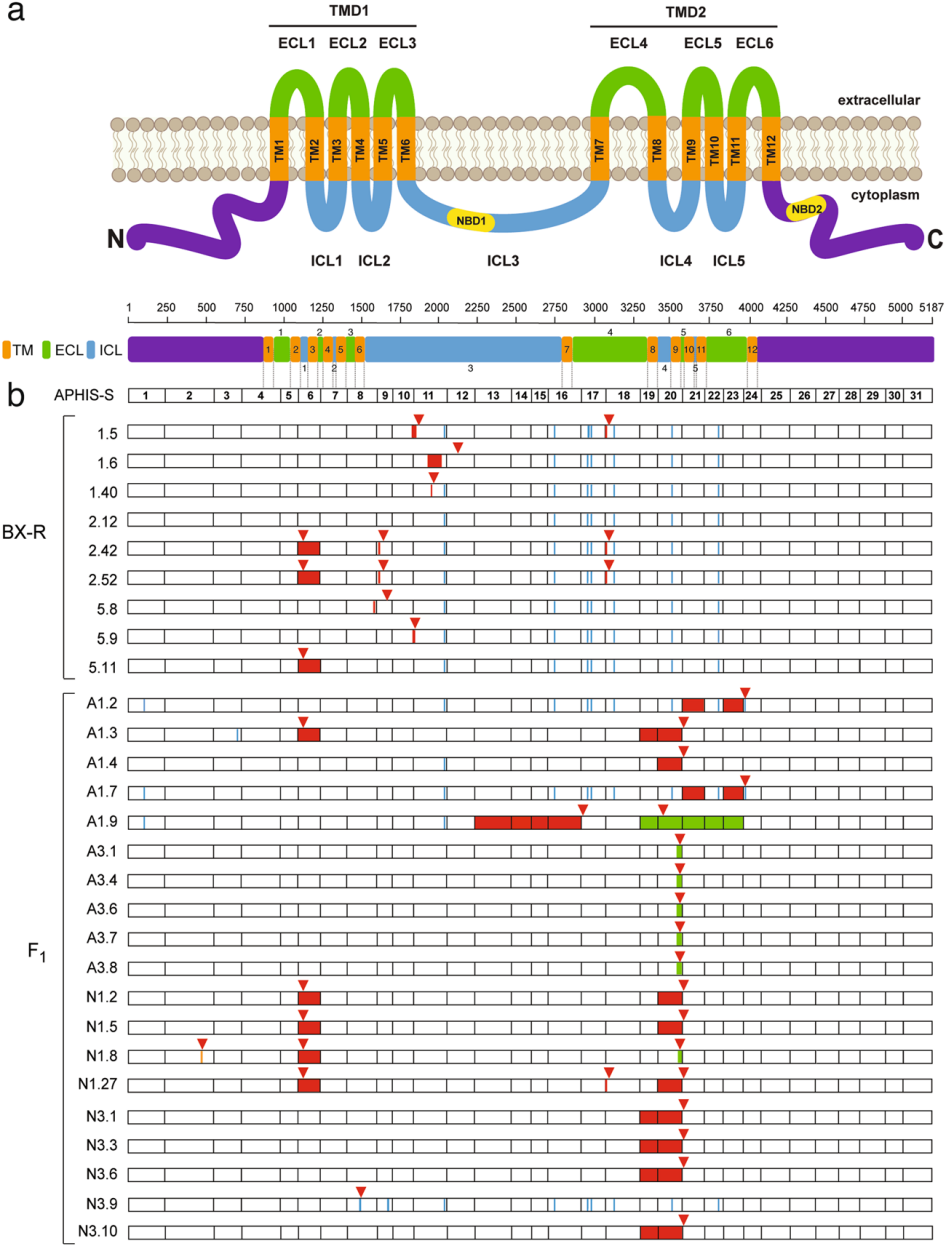
Mutations in 28 *PgABCA2* cDNA sequences from Cry2Ab-resistant strain BX-R and Cry2Ab-resistant F_1_ progeny from BX-R X Bt4-R2. (**a**) The predicted PgABCA2 protein includes amino (N) and carboxyl (C) termini (purple) and two transmembrane domains (TMD1 and TMD2). Each TMD contains six transmembrane-spanning regions (TM, orange), three extracellular loops (ECL, green), and two intracellular loops (ICL, blue). The two TMDs are connected by a single intracellular loop (ICL3). ICL3 and the C-terminal domain each contain a nucleotide-binding domain (NBD, yellow). (**b**) Mutations in *PgABCA2* cDNAs from BX-R (three clones from each of three individuals: 1, 2 and 5) and resistant F_1_ progeny from BX-R X Bt4-R2 (4-5 clones from each of four individuals: A1 and A3 from family A, N1 and N3 from family N, see Fig. 2) relative to the susceptible strain APHIS-S (MG637361). Numbers to the right of the decimal point for each individual indicate the clone sequenced. Exon numbers are shown for APHIS-S. Red triangles above bars indicate premature stop codons, which occur in all sequences except 2.12 from BX-R. Colors within bars show mutations: red for deletions, blue for substitutions, green for insertions and deletions, and orange for the single-bp insertion in exon 2 of N1.8 from the resistant F_1_ progeny.

To determine if BX-R harbored mutations in *PgABCA2* other than *r*_*A1*_, we sequenced cDNA from three clones from each of three BX-R larvae. Confirming the results from the PCR screening of gDNA for the *r*_*A1*_ mutation described above, all nine *PgABCA2* cDNA sequences from the three BX-R larvae lacked disruptive indels near exon 20 (Fig. 3 and Table 1) characteristic of the *r*_*A1*_ mutation^28^. However, all nine sequences from BX-R had other mutations affecting the predicted amino acid sequence (Fig. 3, Table 1, Supplementary Fig. S1). Eight of the nine sequences had at least one premature stop codon caused by a deletion (Fig. 3, Table 1, Supplementary Table S1and Supplementary Fig. S1). The exceptional sequence was from BX-R larva 2, clone 12 (2.12), which had only amino acid substitutions and no deletions, insertions, or premature stop codons (Supplementary Fig. S1).

**Table 1:** Fifteen cDNA mutations in ***PgABCA2*** in three Cry2Ab-resistant larvae from the BX-R strain.

cDNA mutation	Codon^a^	Exon^b^	Type^c^	Effect^d^	In gDNA^e^	Larva.clone^f^
c.1090_1234del	364	6	fs	stop at 373	No	2.42, 2.52, 5.11
c.1609del	537	8	fs	stop at 552	No	5.8
c.1622_1623del	541	9	fs	stop at 517^g^	No	2.42, 2.52
c.1786_1843del	596	10	fs	stop at 597	No	1.5
c.1832_1850del	611	10	fs	stop at 615	No	5.9
c.1905_2031del	635	11	fs	stop at 662	No	1.6
c.1963del	655	11	fs	stop at 664^g^	No	1.40
c.3097_3100del	1033	18	fs	stop at 1027^g,h^	No	1.5, 2.42, 2.52
c.2030 A > T	677	11	ms	E677V	Yes	All but 1.6
c.2753 G > C	918	16	ms	C918S	Yes	All
c.2969 T > C	990	17	ms	L990S	Yes	All
c.2975 C > T	992	17	ms	P992L	Yes	All
c.3163 C > A	1055	18	ms	H1055N	Yes	All
c.3517 G > A	1173	20	ms	V1173I	Yes	All
c.3820 A > G	1274	22	ms	S1274G	Yes	All

^a^Codon where the mutation occurs in the full-length *PgABCA2* cDNA sequence (MG637361.1).

^b^Exon where the mutation occurs.

^c^fs, frameshift caused by deletion; ms, missense (point mutation, single amino acid substitution caused by single bp change).

^d^Position of premature stop codon (first eight mutations) or amino acid substitution (last seven mutations).

^e^Indicates if cDNA mutation also occurs in corresponding gDNA.

^f^Larva (1, 2 or 5) and clone where mutation occurred (e.g., 2.42 indicates larva 2, clone 42); three clones were sequenced from each larva (total of nine clones).

^g^Affected codons in the mutant and wild-type sequence (from “Codon” column) do not match because additional mutations occur upstream of this mutation.

^h^Premature stop codon at 1027 in 1.5; at 997 in 2.42 and 2.52 because of deletion upstream of c.3097_3100del.

Overall, the nine sequences harbored 15 different mutations and a total of 75 mutations counting the multiple occurrences for 10 of the 15 mutations (Table 1). Of the 15 mutations, eight are deletions causing frameshifts that introduce premature stop codons and seven are point mutations that cause single amino acid substitutions (Table 1). Of the seven amino acid substitutions, two are conservative and five are not conservative (E677V, L990S, P992L, H1055N, and S1274G). Each of the eight deletions occurred in at most three sequences from either one or two larvae. By contrast, six of the seven point mutations occurred in all nine sequences and the other occurred in eight of the nine sequences. Only two of the nine sequences were identical; these were from BX-R larva 2, clones 42 and 52 (2.42 and 2.52, Fig. 3, Supplementary Fig. S1).

Sequencing of gDNA fragments corresponding to each of the 15 *PgABCA2* cDNA mutations from BX-R revealed that all eight point mutations, but none of the seven deletions, occurred in gDNA (Table 1, Supplementary Table S2). Mutation c1090_1234del is a 145-bp deletion that causes the complete loss of exon 6 because of mis-splicing (Mathew *et al*. 2018)^28^. Mutations c.1786_1843del, c.1832_1850del, and c.3097_3100del do not cause complete loss of any exons, but are at or span predicted exon-intron junctions and are therefore probably caused by mis-spliced pre-mRNA. Although not precisely located at exon-intron junctions, mutations c.1609del, c.1622_1623del, and c.1963del are within 3–8 bp of their respective splice junctions and could have resulted from incorrect mRNA splicing. The c.1905_2031del mutation is a 127-bp deletion in cDNA not reflected in gDNA that occurs 72 bp upstream of the exon 11 junction and 31 bp downstream of the exon 12 junction. The cause of this deletion is unknown.

### Mutations in *PgABCA2* cDNA from Cry2Ab-resistant F_1_ Progeny of BX-R X Bt4-R2 Single-Pair Crosses

We sequenced *PgABCA2* cDNA from 19 clones from four resistant F_1_ progeny of BX-R X Bt4-R2 single-pair crosses that survived exposure to 10 μg Cry2Ab per mL diet (larvae A1, A3, N1, and N3 from families A and N, Fig. 2; 4 clones from N1 and 5 clones from each of the other larvae, Fig. 3). All 19 cDNA clones sequenced from these four Cry2Ab-resistant larvae had at least one premature stop codon introduced by a frameshift mutation (Fig. 3, Table 2, and Supplementary Fig. S3).

**Table 2:** Twenty-two cDNA mutations in *PgABCA2* in four Cry2Ab-resistant F_1_ larvae from BX-R X Bt4-R2.

Previous^a^	cDNA mutation	Codon^b^	Exon^c^	Type^d^	Effect^e^	In gDNA^f^	Larva.clone^g^
Neither	c.432_433insT	144	2	fs	stop at 145	Yes	N1.8
Both	c.1090_1234del	364	6	fs	stop at 373	No^h^	A1.3, N1.2, N1.5, N1.8, N1.27
Neither	c.2230_2930del	744	13–16	fs	stop at 751	No^i^	A1.9
BX-R	c.3097_3100del	1033	18	fs	stop at 997j	No^k^	N1.27
Bt4-R2	c.3313_3589del	1105	19–20	fs	stop at 1112j,l	No^h^	A1.3, N3.1, N3.3, N3.6, N3.10
Neither	c.3313_3967delins^m^	1106	19–23	fs	stop at 897j	No^n^	A1.9
Bt4-R2	c.3418_3589del	1140	20	fs	stop at 1147°o	No^h^	A1.4, N1.2, N1.5, N1.27
Bt4-R2	c.3556_3588delins^m^	1186	20	fs	stop at 1220j,p	No^n^	A3.1, A3.4, A3.6, A3.7, A3.8, N1.8
Neither	c.3840_3967del	1280	23	fs	stop at 1291	No^h^	A1.2, A1.7
Neither	c.1454 C > A	485	8	ns	stop at 485	Yes	N3.9
Neither	c.3972 A > T	1325	24	ns	stop at 1240j	Yes	A1.2, A1.7
Neither	c.3590_3715del	1197	21	InF	loss of 42 aa	No^h^	A1.2, A1.7
Neither	c.101 C > T	34	1	ms	T34M	Yes	A1.2, A1.7, A1.9
Neither	c.728 A > T	243	3	ms	D243V	Yes	A1.3
Neither	c.1702C > A	568	9	ms	Q568K	Yes	N3.9
BX-R	c.2030 A > T	677	11	ms	E677V	Yes	A1.2, A1.4, A1.7, A1.9, N3.9
BX-R	c.2753 G > C	918	16	ms	C918S	Yes	A1.2, A1.7, N3.9
BX-R	c.2969 T > C	990	17	ms	L990S	Yes	A1.2, A1.7, N3.9
BX-R	c.2975 C > T	992	17	ms	P992L	Yes	A1.2, A1.7, N3.9
BX-R	c.3163 C > A	1055	18	ms	H1055N	Yes	A1.2, A1.7, N3.9
BX-R	c.3517 G > A	1173	20	ms	V1173I	Yes	A1.2, A1.7, N3.9
BX-R	c.3820 A > G	1274	22	ms	S1274G	Yes	A1.2, A1.7, N3.9

^a^cDNA mutation found previously in BX-R (Table 1), Bt4-R2 (Mathew *et al*. 2018), both, or neither.

^b^Codon where the mutation occurs in the full-length *PgABCA2* cDNA sequence (MG637361.1).

^c^Exon where the mutation occurs.

^d^fs, frameshift caused by deletion; ns, nonsense mutation (single base-pair substitution that introduces premature stop codon); InF, in-frame deletion; ms, missense (point mutation, single amino acid substitution caused by single bp change).

^e^Position of premature stop codon (first 11 mutations), amino acids lost (c.3590_3715del), or amino acid substitution (last 10 mutations).

^f^Indicates if cDNA mutation also occurs in corresponding gDNA.

^g^Larva (A1, A3, N1, or N3) and clone where mutation occurred (e.g., N1.8 indicates larva 1 from family N, clone 8); 4–5 clones were sequenced from each larva (total of 19 clones).

^h^Mis-spliced, deletion caused by exon skipping.

^i^Mis-spliced, deletion due to alternative 5′ and 3′ splice sites.

^j^Affected codons in the mutant and wild-type sequence (from “Codon” column) do not match because additional mutations occur upstream of this mutation.

^k^Mis-spliced, deletion due to alternative 3′ splice site.

^l^Premature stop codon at 1063 in A1.3 because of deletion upstream of c.3313_3589del.

^m^Sequence corresponding to insertion not shown due to large number of nucleotide bases.

^n^Mis-spliced, indel caused by intron retention.

^o^Premature stop codon at 1097 in N1.27 and at 1098 in N1.2 and N1.5 because of deletions upstream of c.3418_3589del.

^p^Premature stop codon at 1172 in N1.8 because of deletion upstream of c.3556_3588delins.

Overall, the 19 sequences from the resistant F_1_ progeny harbored 22 different mutations and a total of 59 mutations counting the multiple occurrences for 15 of the 22 mutations (Table 2). Of the 22 different mutations in the resistant F_1_ progeny, twelve were previously identified from BX-R, Bt4-R2, or both; including seven point mutations and one new deletion in BX-R discovered here (Fig. 3, Tables 1 and 2) and four mutations in Bt4-R2 reported by Mathew *et al*. (2018)^28^ (Fig. 3, Table 2). Only one mutation (c.1090_1234del) was found previously in both BX-R and Bt4-R2 (Fig. 3, Table 2). Ten mutations in the resistant F_1_ progeny were not found previously in either BX-R or Bt4-R2 (Fig. 3, Table 2). Nine of the 22 mutations in the resistant F_1_ progeny probably reflect mis-splicing (Table 2, Fig. 3, Supplementary Table S3 and Supplementary Fig. S3). Whereas five of these nine mutations were caused by exon skipping, one involved alternative 5′ and 3′ splice sites, one involved an alternative 3′ splice site, and two indel mutations were caused by intron retention (Table 2, Supplementary Fig. S3). Mutation c.3097_3100del, which was also found in BX-R and in a Cry2Ab-resistant larva (KT-1) from India^28^, is caused by the alternative use of a 3′ splice site (Table 2, Supplementary Fig. S3).

## Discussion

We found that two independently derived, lab-selected strains of pink bollworm, BX-R and Bt4-R2, share a mechanism of resistance to Cry2Ab, and at least one additional mechanism of resistance occurs in BX-R. The results here confirm our previous findings that disruptive mutations in the ABC transporter gene *PgABCA2* are associated with the autosomal, recessive resistance of Bt4-R2 to Cry2Ab^28^. We discovered in this study that resistance of BX-R to Cry2Ab is also associated with disruptive mutations in *PgABCA2*. We previously found that mutations disrupting *PgABCA2* are also associated with resistance to Cry2Ab in field-selected populations of pink bollworm from India^28^. Thus, mutations disrupting *PgABCA2* are the primary, but not the only, mechanism of resistance to Cry2Ab in pink bollworm.

The recessive mutation *r*_*A1*_, which introduces a premature stop codon in *PgABCA2*, was fixed in Bt4-R2 in the current and previous study^28^, but was not detected in BX-R. Nonetheless, BX-R harbored other mutations disrupting *PgABCA2*. For six of the 20 families from single-pair crosses between BX-R and Bt4-R2, larval survival at 10 μg Cry2Ab per mL diet was >99%, implying the parents of these families from both strains had resistance alleles at the same locus (i.e., *PgABCA2*). However, for five of the 20 single-pair families, larval survival was less than 70% at the same toxin concentration. In light of the >92% survival of each strain at this toxin concentration and the high frequency of *r*_*A1*_ in Bt4-R2, the <70% survival in five of the 20 single-pair families implies that mutations at one or more loci other than *PgABCA2* contributed to Cry2Ab resistance in BX-R.

Moreover, in contrast with all results from Bt4-R2 and previous results from BX-R^25^, resistance of BX-R to Cry2Ab was not entirely autosomal or recessive in the current study. We previously reported that BX-R resistance to Cry2Ab was autosomal and completely recessive (*h* = 0) at concentrations of 3 and 10 μg Cry2Ab per mL diet^25^. Here, mean survival was 25% higher for F_1_ progeny from crosses between BX-R and susceptible strain APHIS-S with BX-R fathers than BX-R mothers, indicating non-autosomal inheritance of resistance. In this study, mean values of *h* for BX-R were 0.82 at 3 μg Cry2Ab per mL diet and 0.35 at 10 μg Cry2Ab per mL diet, indicating non-recessive inheritance of resistance.

We hypothesize that the differences between the current versus previous results with BX-R reflect changes that occurred during the decade (>100 generations) between the two studies. Given that the mutations in *PgABCA2* confer autosomal, recessive resistance to Cry2Ab, we infer this was the primary mechanism of resistance in BX-R at the time of the previous study^25^. Unfortunately, we do not have the preserved samples required to test this hypothesis. We also hypothesize that after the previous study, the frequency of non-autosomal, non-recessive resistance increased in BX-R. The one or more non-autosomal, non-recessive mechanisms of resistance to Cry2Ab in BX-R remain to be determined, but probably involve something other than reduced toxin binding, which is typically inherited as a recessive trait^13,40^.

The results here for BX-R resistance to Cry2Ab have some striking parallels with resistance to Cry1Ac in the field-selected R strain of *Plutella xylostella* from the Philippines^41^. In the R strain, resistance was associated with reduced binding of Cry1Ac and inheritance of reduced binding was autosomal and recessive. However, as for BX-R, survival was higher for F_1_ progeny from crosses with resistant fathers and susceptible mothers than for the reciprocal cross, yet sex ratios of the F_1_ progeny did not fit expectations for sex linkage. Thus, similar to our results with BX-R, the R strain had an autosomal recessive mechanism of resistance (reduced binding), as well as at least one additional mechanism that was not autosomal, not recessive, and did not fit expectations for either maternal or sex-linked inheritance. In a related study, Petzold-Maxwell *et al*. (2012)^42^ also reported higher resistance to Cry3Bb1 of F_1_ progeny from crosses with resistant fathers and susceptible mothers relative to the reciprocal cross in a lab-selected strain of western corn rootworm (*Diabrotica virgifera virgifera*). In this case, because males are heterogametic in Coleoptera (the opposite of Lepidoptera), F_1_ progeny from crosses with resistant fathers and susceptible mothers are expected to have lower resistance relative to the progeny from the reciprocal cross under sex-linked inheritance. Thus, this represents a third example of non-autosomal inheritance of Bt resistance that does not fit either maternal or sex-linked inheritance.

Together with previous findings^28^, the results here show that many mutations in *PgABCA2* occur in Cry2Ab-resistant pink bollworm from BX-R, Bt4-R2, and field-selected populations from India. Here we found 15 different *PgABCA2* mutations in three larvae from BX-R and 22 different mutations in four F_1_ progeny from crosses between BX-R and Bt4-R2. Collectively, in the current and previous study^28^, we have identified 47 different cDNA mutations in *PgABCA2* from the 29 larvae analyzed from BX-R, Bt4-R2, and India.

We found only one of the 47 mutations, c1090_1234del, that was common to Cry2Ab-resistant larvae from BX-R, Bt4-R2 and India. This mutation, which causes complete skipping of exon 6 and introduces a premature stop codon, occurred in two of three BX-R larvae (Table 1), two of 14 Bt4-R2 larvae^28^, and three of four resistant F_1_ progeny from BX-R X Bt4-R2 (Table 2). This was also the most common mutation in the field-selected Cry2Ab-resistant larvae from India^28^. It occurred in six of eight resistant larvae tested and was found in larvae from all four states of India studied (Andhra Pradesh, Karnataka, Maharashtra and Telangana)^28^. Another mutation, c.3097_3100del, was detected in two of the three larvae from BX-R, one of the four Cry2Ab-resistant F_1_ progeny from BX-R X Bt4-R2, and in one larva from India (KT-1, mislabeled as c.3098_3101del in Mathew *et al*. 2018^28^), but not in Bt4-R2. We found each of the other 45 mutations only in one of the three sources of Cry2Ab-resistant pink bollworm we studied: BX-R, Bt4-R2 or India.

Overall, 59% (28 of 47) of the cDNA mutations identified in *PgABCA2* introduce a premature stop codon, including all three mutations mentioned above. Mis-splicing of *PgABCA2* pre-mRNA is implicated in eight of the 15 different mutations we found in BX-R (Table 1), including both of the mutations mentioned above that were also found in field-selected larvae from India. Mis-splicing is also involved in all eight mutations previously analyzed from Bt4-R2^28^, four of the ten mutations in F_1_ progeny from BX-R X Bt4-R2 not identified from previous analyses (Table 2), and in five of the six mutations analyzed from India^28^. Overall, we estimate mis-splicing is involved in 61% (22 of 36) of the cDNA mutations in *PgABCA2* characterized here and previously^28^.

Similar to the results showing a prominent role of mis-splicing and diverse mutations disrupting *PgABCA2* in pink bollworm resistance to Cry2Ab in both lab- and field-selected resistance to Cry2Ab, mis-splicing and diverse mutations disrupting the cadherin gene *PgCad1* are important in lab- and field-selected pink bollworm resistance to Cry1Ac^32,37–39,43,44^. In field populations from China that have remained mostly susceptible to Cry1Ac, the most common *PgCad1* resistance allele (*r1*) was first identified from a lab-selected strain in the United States^32^. However, unlike the two deletions in *PgABCA2* (c1090_1234del and c.3098_3101del) associated with Cry2Ab resistance that are shared between field-selected populations from India and one or both lab-selected strains from the United States, none of the specific *PgCad1* mutations in field-selected populations from India were detected in lab-selected strains from the U.S. and China^32,44^. For *H. armigera* from China, mutations in *HaCad* and a tetraspanin gene (*HaTSPAN1)* linked with resistance to Cry1Ac in lab-selected strains also occur in field-selected populations where early warning of resistance has been reported^15,45^.

Aside from pink bollworm in India, the only other currently documented case of practical resistance to Cry2Ab involves *Helicoverpa zea* in the United States^9,46,47^. It will be intriguing to determine if this resistance is caused by mutations affecting *ABCA2*, other mechanisms, or both.

## Methods

### Insects

We used three strains of pink bollworm that originated from the southwestern United States: APHIS-S, Bt4-R2, and BX-R. APHIS-S is a susceptible strain that had been reared in the laboratory for more than 30 years without exposure to Bt toxins or insecticides^48,49^. Bt4-R2 was obtained from the Bt4R strain in 2010 and had >10,000-fold resistance to Cry2Ab relative to APHIS-S that is associated with a mutation in and mis-splicing of *PgABCA2*^27,28^. BX-R was started in 2006 by pooling 875 pupae from substrains BX-R1 and BX-R2, which had been selected in the laboratory with Cry2Ab^25,50^ and had 210- to 240-fold resistance to Cry2Ab relative to APHIS-S^25,51^. Larvae were fed wheat germ diet^52^. We conducted rearing, crosses and bioassays at 26 °C, 14 h light:10 h dark. For several years before this study, we selected resistance in Bt4-R2 and BX-R by rearing larvae on diet containing 3 μg Cry2Ab and 10 μg Cry1Ac per mL diet approximately every six generations.

### Bt toxin

We used Cry2A.127, an engineered variant of Cry2Ab, that was prepared as described previously^53^ and provided by DuPont Pioneer (now Corteva Agriscience) as a purified and solubilized protoxin. Cry2A.127 shares 98.6% amino acid sequence identity with Cry2Ab1 and Cry2Ab2 (9 substitutions out of 633 amino acids) and is referred to here as Cry2Ab.

### Diet Bioassays

We used diet incorporation bioassays to evaluate susceptibility to Cry2Ab^25,36,54^. Individual neonates were placed in wells of bioassay trays (BIO-BA-128, Pitman, NJ) containing ~1 g of diet and covered with Pull N’ Peel tab tray covers (BIO-CU-16, Pitman, NJ). We used 0 (control), 3, or 10 μg Cry2Ab per mL diet. We scored live fourth instars, pupae, and adults as survivors after 21 and 18 days in the first and second set of bioassays from mass crosses, respectively. We used the shorter duration in the second set to facilitate preservation of survivors before they emerged as moths. Survival at 10 μg Cry2Ab per mL diet did not differ significantly between the first and second set of bioassays for each strain or group of F_1_ progeny (Fisher’s exact test, P > 0.54 for each comparison).

### Dominance, Sex Linkage, and Maternal Effects

To evaluate dominance, sex linkage, and maternal effects of resistance to Cry2Ab in Bt4-R2 and BX-R, we used two sets of bioassays with larvae produced by mass crosses. We started the first and second sets of bioassays in February and March 2015, with the F_45_ and F_46_ generations of Bt4-R2 and the F_84_ and F_85_ generations of BX-R, respectively. In the generation immediately preceding the first set of bioassays, we had selected both strains on diet containing 3 μg Cry2Ab + 10 μg Cry1Ac per mL diet. In both sets of bioassays, we tested F_1_ progeny from the following seven mass crosses: three within-strain crosses (APHIS-S, Bt4-R2, and BX-R) and four reciprocal crosses between each resistant strain and the susceptible strain APHIS-S (♀ Bt4-R2 X ♂ APHIS-S, ♀ APHIS-S X ♂ Bt4-R2, ♀ BX-R X ♂ APHIS-S, and ♀ APHIS-S X ♂ BX-R). For each mass cross, we put 30 pupae of each sex in paper cups (350 mL) and allowed adults to emerge in the cups, mate, and oviposit^27^. In the first set of bioassays, we tested 32 larvae from the F_1_ progeny of each mass cross at 0 (control), 3 or 10 μg Cry2Ab per mL diet. In the second set of bioassays, we tested 16 larvae from the F_1_ progeny of each mass cross at 0 or 10 μg Cry2Ab per mL diet (total n for both sets = 872).

### Interstrain Complementation Test for Allelism

To determine if the locus or loci conferring resistance to Cry2Ab differed between Bt4-R2 and BX-R, we performed interstrain complementation tests for allelism^54–56^. We conducted the single-pair crosses and associated bioassays simultaneously with the second set of mass crosses and bioassays described above. In each of 40 plastic cups (30 mL plastic Solo cups, Dart Container Cooperation, Mason, MI), we put one female Bt4-R2 pupa and one male BX-R pupa. In each of another 40 cups, we put one female BX-R pupa and one male Bt4-R2 pupa. After adults eclosed, lids were replaced with new ones containing a vial of 15% sucrose and paper for oviposition pinned inside. We tested F_1_ progeny from 10 families from each of the two reciprocal crosses on control diet without Cry2Ab (12–16 neonates per family) or on diet with 10 μg Cry2Ab per mL diet (17–32 neonates per family). After 18 days, we recorded survival and collected all surviving 4^th^ instars and pupae.

### Statistical Analyses of Bioassay Data

#### Adjusted Survival

For all bioassays, we calculated adjusted survival (%) as the survival (%) for a strain or group of F_1_ progeny tested on treated diet divided by the survival (%) for the same strain or group of F_1_ progeny tested simultaneously on untreated (control) diet. All values for survival reported in Results and used for analyses reflect adjusted survival. Mean survival on control diet was 78.1% (SE = 3.4%) and 88.4% (SE = 2.9%) in the first and second sets, respectively.

#### Sex Linkage and Maternal Effects

To evaluate sex linkage and maternal effects influencing resistance to Cry2Ab, we compared survival of F_1_ progeny between the two reciprocal crosses with APHIS-S for Bt-R2 and separately for BX-R^48,54^. If no significant difference occurs between the reciprocal crosses for a resistant and susceptible strain, inheritance of resistance is considered autosomal, with no evidence of sex linkage or maternal effects. Maternal effects can cause higher survival for the progeny from resistant mothers and susceptible fathers than from susceptible mothers and resistant fathers. Conversely, for Lepidoptera (but not most other insects), sex-linked inheritance of resistance can cause higher survival for the progeny from resistant fathers and susceptible mothers than from resistant mothers and resistant fathers. This is because, for the sex chromosome in Lepidoptera (but not most other insects), females are heterogametic (ZW) and males are homogametic (ZZ)^57^. This means that for sex-linked resistance, females are hemizygotic and carry only one allele at each locus affecting resistance, whereas males carry two such alleles.

With sex linkage inheritance of resistance, the genotypes for a resistant strain can be written as Z^r^W for females and Z^r^Z^r^ for males; genotypes for a susceptible strain as Z^s^W for females and Z^s^Z^s^ for males^41^. Thus, crosses between resistant mothers (Z^r^W) and susceptible fathers (Z^s^Z^s^) produce Z^s^W daughters and Z^r^Z^s^ sons. Crosses between resistant fathers (Z^r^Z^r^) and susceptible mothers (Z^s^W) produce Z^r^W daughters and Z^r^Z^s^ sons. Because survival is expected to be higher for Z^r^W than Z^s^W daughters, higher survival on treated diet is expected in the progeny from crosses with resistant fathers than with resistant mothers. Also, because of the low expected survival of Z^s^W daughters on treated diet, the proportion of males is expected to be higher in the crosses with resistant mothers than with resistant fathers.

#### Dominance

We evaluated the dominance parameter *h*, which varies from 0 for completely recessive resistance to 1 for completely dominant resistance. We calculated *h* for Bt4-R2 and BX-R as: *h* = (*w*_12_ – *w*_22_)/(*w*_11_ – *w*_22_), where *w*_11_, *w*_12_, and *w*_22_ are the fitnesses at a particular toxin concentration for resistant homozygotes, heterozygotes, and susceptible homozygotes, respectively^58^. As typically done, we applied this method by assuming survival in bioassays is proportional to fitness, the resistant (*R*) strain is homozygous for resistance, the susceptible (*S*) strain is homozygous for susceptibility, and their F_1_ progeny are heterozygotes, which yields: *h* = (F_1_ survival − *S* survival)/(*R* survival − *S* survival), where the *S* strain is APHIS-S, and the *R* strain is either Bt4-R2 or BX-R.

### Sex Determination of F_1_ Survivors from Mass Cross Bioassays

Because male fourth instar pink bollworm larvae have purple or dark brown colored testis visible through the dorsal side of their cuticle at ~6–7^th^ segment^59^, we determined the sex of preserved F_1_ progeny from mass crosses using a dissecting microscope. For specimens for which testis were not observed, larvae were dissected to confirm they lacked testis and/or possessed female reproductive tissue (e.g. ovaries). For samples preserved as pupae, we used the methods by Butt and Cantu (1962)^60^ for sex determination.

### Allele-Specific PCR of gDNA

We used PCR genotyping of gDNA adapted from Mathew *et al*. (2018)^28^ to determine whether survivors on 10 μg Cry2A.127 per mL diet from 20 single-pair families from BX-R X Bt4-R2 (Fig. 2) had 0, 1 or 2 copies of the *r*_*A1*_ mutation at the *PgABCA2* locus. We extracted gDNA of three survivors from 19 of the 20 families and the only two survivors from family O (total n = 59). We used 0.1–0.6 μg gDNA as template for PCR with primers r_A1_-F and r_A1_-R. PCR amplicons were separated on 4% agarose gels containing 1X SYBR Safe DNA Gel Stain (Thermo Fisher Scientific, Waltham, MA) and checked for the 305-bp *r*_*A1*_ fragment and the 343-bp wild-type fragment. We performed a second genotyping PCR on each gDNA sample using primers r_A1_-F2 and r_A1_-R (Supplementary Table S4). These amplify only the wild-type sequence in this region of *PgABCA2* because r_A1_-F2 corresponds to a wild-type sequence that is missing in alleles with the *r*_*A1*_ mutation.

### cDNA Cloning

For cDNA cloning of *PgABCA2*, we used three 4^th^ instar survivors on 3 μg Cry2Ab per mL diet from the BX-R strain as well as two 4^th^ instar F_1_ survivors on 10 μg Cry2Ab per mL diet from two single-pair cross families. Total RNA was extracted using TRI Reagent (Thermo Fisher Scientific) and treated with DNase I (Thermo Fisher Scientific). cDNA was prepared using 1 μg of total RNA with both random hexamer primers and oligo-dT primers with a SuperScript IV First-Strand cDNA synthesis kit (Thermo Fisher Scientific) according to the manufacturer’s instruction. *PgABCA2* cDNA was amplified in PCR using Platinum SuperFi Green PCR Master Mix (Thermo Fisher Scientific) DNA polymerase and 1 μM 163pgABCA2–5 and 166pgABCA2–3 oligonucleotide primers (Supplementary Table S4) at: 98 °C for 30 s (1 cycle); 40 cycles of 98 °C for 10 s, 50.9 °C for 10 s and 72 °C for 3 min; then 72 °C for 5 min. PCR products were separated on 1% agarose gels stained with SYBR Safe (Thermo Fisher Scientific) and viewed using an LED UV Illuminator (Maestrogen, Hsinchu City, Taiwan). Bands were gel-purified using E.Z.N.A. Gel Extraction Kit (Omega Bio-Tek, Norcross, GA) and ligated into pCR-XL-2-TOPO (Thermo Fisher Scientific). Plasmids were propagated in TOP10 OneShot Chemically Competent *E. coli* by growing on LB agar plates containing 50 μg per mL Carbenicillin (Research Products International, Mount Prospect, IL) for 72 h at room temperature. Colonies were prescreened using PCR and plasmid DNA was purified using a QIAprep Spin MiniPrep kit with a QIAcube system (Qiagen, Hilden, Germany). Inserts were sequenced by Retrogen (San Diego, CA) with primers: M13 reverse, T7, 63PgABCA2–3, 64PgABCA2–5, 65PgABCA2–3, 66PgABCA2–5, 67PgABCA2–3, 68PgABCA2–5, 69PgABCA2–3, 70PgABCA2–5, 71PgABCA2–3, 72PgABCA2–5, 73PgABCA2–3, 74PgABCA2–5, 75PgABCA2–3, 76PgABCA2–5, 77PgABCA2–5 and 78PgABCA2–5 (Supplementary Table S4). *PgABCA2* coding sequences obtained from BX-R and F_1_ progeny between BX-R X Bt4-R2 are deposited in the GenBank public database (MT152670-MT152697). Mutation names are based on guidelines from the Human Genome Variation Society (http://www.hgvs.org/).

### gDNA Cloning

To assess if *PgABCA2* mutations in BX-R, initially identified in cDNA from three BX-R individuals, were caused by mutations in gDNA or in messenger RNA (mRNA), we PCR amplified, cloned, and sequenced corresponding gDNA PCR fragments. We extracted gDNA from 4^th^ instar heads using the Gentra Puregene Tissue Kit (Qiagen) according to the manufacturer’s instructions. *PgABCA2* gDNA fragments were PCR amplified using the Platinum SuperFi Green PCR Master Mix (Thermo Fisher Scientific) and gene-specific oligonucleotide primers (Supplementary Table S4) designed from exons adjacent to mutations identified from cDNA sequencing (Table 1). PCR products were analyzed by agarose gel electrophoresis. DNA bands were gel-purified and cloned into pCR2.1-TOPO (Thermo Fisher Scientific) and Sanger sequenced as above.

## Supplementary information

Supplementary information.

Supplementary figure S1.

Supplementary figure S2.

Supplementary figure S3.

Supplementary table S1.

Supplementary table S2.

Supplementary table S3.

Supplementary table S4.
