# Supplementary material for: Shared and Independent Genetic Basis of Resistance to Bt Toxin Cry2Ab in Two Strains of Pink Bollworm
Source: Sci Rep. 2020 May 14;10:7988. doi: 10.1038/s41598-020-64811-w (PMC7224296; doi:10.1038/s41598-020-64811-w)
Supplement: Supplementary file 1 — Supplementary information. [file 41598_2020_64811_MOESM1_ESM.docx]

**SUPPLEMENTARY INFORMATION**

**Shared and Independent Genetic Basis of Resistance to Bt Toxin Cry2Ab in Two Strains of Pink Bollworm**

Jeffrey A. Fabrick^1,*^, Dannialle M. LeRoy^1^, Gopalan C. Unnithan^2^, Alex J. Yelich^2^, Yves Carrière^2^, Xianchun Li^2^, and Bruce E. Tabashnik^2^

^1^ USDA ARS, U.S. Arid Land Agricultural Research Center, Maricopa, AZ 85138 USA

^2^ Department of Entomology, University of Arizona, Tucson, AZ 85721 USA

^*^ Corresponding author:

Jeffrey A. Fabrick

USDA ARS, U.S. Arid Land Agricultural Research Center

21881 N. Cardon Lane

Maricopa, AZ 85138, USA

Phone: 520-316-6335

Email: jeff.fabrick@usda.gov

**SUPPLEMENTARY INFORMATION includes:**

**Supplementary Tables S1-S4**

**Supplementary Figure Legends**

**Supplementary Figures S1-S3**

**Supplementary Figure S1. Alignment of *PgABCA2* cDNA sequences from BX-R with susceptible *PgABCA2* (MG637361.1) from APHIS-S.** Nine cDNA sequences from three clones from each of three BX-R individuals (1, 2, and 5 refer to the individuals and the numbers to the right of the decimal point refer to the clones) are aligned with the wild-type *PgABCA2* (MG637361.1) from susceptible strain APHIS-S. Alignment was generated using Clustal Omega (<https://www.ebi.ac.uk/Tools/msa/clustalo/>). Stars show bases conserved in all sequences. Deletions are highlighted in red, missense substitutions in blue, and predicted exon/intron splice sites in gray. Sequences of specific primers used to amplify gDNA fragments near cDNA mutations are underlined.

**Supplementary Figure S2. Alignment of *PgABCA2* gDNA sequences corresponding to cDNA mutations from BX-R.** Consensus partial gDNA sequences were generated from at least three independent clones, intron sequences were removed, and sequences were aligned with the wild-type *PgABCA2* (MG637361.1) from APHIS-S and mutant BX-R cDNA sequences corresponding to 68PgABCA2-5 + 141PgABCA2-3 (A), 126PgABCA2-5 + 127PgABCA2-3 (B), 86PgABC5 + 87PgABC3 (C), 124PgABCA2-5 + 154PgABCA2-3 (D), 85PgABC5 + 88PgABC3 (E), rA1-F + 82PgABCA2-3 (F), 89PgABC5 + 90PgABC3 (G), 143PgABCA2-5 + 90PgABCA2-3 (H), and 186PgABCA2-5 + 185PgABCA2-3 (I). The order of sequences is based on their similarity determined by Clustal Omega (<https://www.ebi.ac.uk/Tools/msa/clustalo/>). Stars show nucleotide bases conserved in all of the sequences. Deletions are highlighted in red, missense substitutions in blue, and predicted exon/intron splice sites in gray. *PgABCA2*-specific oligonucleotide primers designed from exons adjacent to mutations and used to amplify gDNA fragments are underlined.

**Supplementary Figure S3. Alignment of *PgABCA2* cDNA sequences from BX-R X Bt4-R2 F_1_ survivors on 10 μg Cry2Ab per mL diet from two single-pair cross families.** A total of 19 cDNA sequences were obtained from two individuals from BX-R X Bt4-R2 single pair families A and N. The order of sequences is based on their similarity determined by Clustal Omega (<https://www.ebi.ac.uk/Tools/msa/clustalo/>). Stars show nucleotide bases conserved in all of the sequences. Deletions are highlighted in red, insertions and deletions (indels) in green, insertions in orange, missense substitutions in blue, and predicted exon/intron splice sites in gray.
