# Supplementary material for: Shared and Independent Genetic Basis of Resistance to Bt Toxin Cry2Ab in Two Strains of Pink Bollworm
Source: Sci Rep. 2020 May 14;10:7988. doi: 10.1038/s41598-020-64811-w (PMC7224296; doi:10.1038/s41598-020-64811-w)
Supplement: Supplementary file 2 — Supplementary figure S1. [file 41598_2020_64811_MOESM2_ESM.docx]

MG637361.1 ATGCGGGCGCGTGGAGAGCGGAAGGAGGCGGGCTCATGGGTGAAGTTTAGGCTGTTGATG 60

1.5 ATGCGGGCGCGTGGAGAGCGGAAGGAGGCGGGCTCATGGGTGAAGTTTAGGCTGTTGATG 60

1.6 ATGCGGGCGCGTGGAGAGCGGAAGGAGGCGGGCTCATGGGTGAAGTTTAGGCTGTTGATG 60

1.40 ATGCGGGCGCGTGGAGAGCGGAAGGAGGCGGGCTCATGGGTGAAGTTTAGGCTGTTGATG 60

2.12 ATGCGGGCGCGTGGAGAGCGGAAGGAGGCGGGCTCATGGGTGAAGTTTAGGCTGTTGATG 60

2.42 ATGCGGGCGCGTGGAGAGCGGAAGGAGGCGGGCTCATGGGTGAAGTTTAGGCTGTTGATG 60

2.52 ATGCGGGCGCGTGGAGAGCGGAAGGAGGCGGGCTCATGGGTGAAGTTTAGGCTGTTGATG 60

5.8 ATGCGGGCGCGTGGAGAGCGGAAGGAGGCGGGCTCATGGGTGAAGTTTAGGCTGTTGATG 60

5.9 ATGCGGGCGCGTGGAGAGCGGAAGGAGGCGGGCTCATGGGTGAAGTTTAGGCTGTTGATG 60

5.11 ATGCGGGCGCGTGGAGAGCGGAAGGAGGCGGGCTCATGGGTGAAGTTTAGGCTGTTGATG 60

************************************************************

MG637361.1 TGGAAGAACTTCGTGCAGCAGTTGAGGCACCCAGTGCAGACGGCGGCTGAGCTGCTGCTA 120

1.5 TGGAAGAACTTCGTGCAGCAGTTGAGGCACCCAGTGCAGACGGCGGCTGAGCTGCTGCTA 120

1.6 TGGAAGAACTTCGTGCAGCAGTTGAGGCACCCAGTGCAGACGGCGGCTGAGCTGCTGCTA 120

1.40 TGGAAGAACTTCGTGCAGCAGTTGAGGCACCCAGTGCAGACGGCGGCTGAGCTGCTGCTA 120

2.12 TGGAAGAACTTCGTGCAGCAGTTGAGGCACCCAGTGCAGACGGCGGCTGAGCTGCTGCTA 120

2.42 TGGAAGAACTTCGTGCAGCAGTTGAGGCACCCAGTGCAGACGGCGGCTGAGCTGCTGCTA 120

2.52 TGGAAGAACTTCGTGCAGCAGTTGAGGCACCCAGTGCAGACGGCGGCTGAGCTGCTGCTA 120

5.8 TGGAAGAACTTCGTGCAGCAGTTGAGGCACCCAGTGCAGACGGCGGCTGAGCTGCTGCTA 120

5.9 TGGAAGAACTTCGTGCAGCAGTTGAGGCACCCAGTGCAGACGGCGGCTGAGCTGCTGCTA 120

5.11 TGGAAGAACTTCGTGCAGCAGTTGAGGCACCCAGTGCAGACGGCGGCTGAGCTGCTGCTA 120

************************************************************

MG637361.1 CCAGTCCTAACCATGAGCCTGGTCCTGGTGCTACGGTCACAGATCGACCCCGAAGTCTTG 180

1.5 CCAGTCCTAACCATGAGCCTGGTCCTGGTGCTACGGTCACAGATCGACCCCGAAGTCTTG 180

1.6 CCAGTCCTAACCATGAGCCTGGTCCTGGTGCTACGGTCACAGATCGACCCCGAAGTCTTG 180

1.40 CCAGTCCTAACCATGAGCCTGGTCCTGGTGCTACGGTCACAGATCGACCCCGAAGTCTTG 180

2.12 CCAGTCCTAACCATGAGCCTGGTCCTGGTGCTACGGTCACAGATCGACCCCGAAGTCTTG 180

2.42 CCAGTCCTAACCATGAGCCTGGTCCTGGTGCTACGGTCACAGATCGACCCCGAAGTCTTG 180

2.52 CCAGTCCTAACCATGAGCCTGGTCCTGGTGCTACGGTCACAGATCGACCCCGAAGTCTTG 180

5.8 CCAGTCCTAACCATGAGCCTGGTCCTGGTGCTACGGTCACAGATCGACCCCGAAGTCTTG 180

5.9 CCAGTCCTAACCATGAGCCTGGTCCTGGTGCTACGGTCACAGATCGACCCCGAAGTCTTG 180

5.11 CCAGTCCTAACCATGAGCCTGGTCCTGGTGCTACGGTCACAGATCGACCCCGAAGTCTTG 180

************************************************************

MG637361.1 GAAACCAGAACCTACCCGCCAATACCAGCCCACACTTTAAACTATTCCGTGACTGTTTTG 240

1.5 GAAACCAGAACCTACCCGCCAATACCAGCCCACACTTTAAACTATTCCGTGACTGTTTTG 240

1.6 GAAACCAGAACCTACCCGCCAATACCAGCCCACACTTTAAACTATTCCGTGACTGTTTTG 240

1.40 GAAACCAGAACCTACCCGCCAATACCAGCCCACACTTTAAACTATTCCGTGACTGTTTTG 240

2.12 GAAACCAGAACTTACCCGCCAATACCAGCCCACACTTTAAACTATTCCGTGACTGTTTTG 240

2.42 GAAACCAGAACCTACCCGCCAATACCAGCCCACACTTTAAACTATTCCGTGACTGTTTTG 240

2.52 GAAACCAGAACCTACCCGCCAATACCAGCCCACACTTTAAACTATTCCGTGACTGTTTTG 240

5.8 GAAACCAGAACCTACCCGCCAATACCAGCCCACACTTTAAACTATTCCGTGACTGTTTTG 240

5.9 GAAACCAGAACCTACCCGCCAATACCAGCCCACACTTTAAACTATTCCGTGACTGTTTTG 240

5.11 GAAACCAGAACCTACCCGCCAATACCAGCCCACACTTTAAACTATTCCGTGACTGTTTTG 240

*********** ************************************************

MG637361.1 GGCGGAATGAATTTAACAAGAATGTCCATGGCATTCTCACCCGAGAATGCCGTATTGAGG 300

1.5 GGCGGAATGAATTTAACAAGAATGTCCATGGCATTCTCACCCGAGAATGCCGTATTGAGG 300

1.6 GGCGGAATGAATTTAACAAGAATGTCCATGGCATTCTCACCCGAGAATGCCGTATTGAGG 300

1.40 GGCGGAATGAATTTAACAAGAATGTCCATGGCATTCTCACCCGAGAATGCCGTATTGAGG 300

2.12 GGCGGAATGAATTTAACAAGAATGTCCATGGCATTCTCACCCGAGAATGCCGTATTGAGG 300

2.42 GGCGGAATGAATTTAACAAGAATGTCCATGGCATTCTCACCCGAGAATGCCGTATTGAGG 300

2.52 GGCGGAATGAATTTAACAAGAATGTCCATGGCATTCTCACCCGAGAATGCCGTATTGAGG 300

5.8 GGCGGAATGAATTTAACAAGAATGTCCATGGCATTCTCACCCGAGAATGCCGTATTGAGG 300

5.9 GGCGGAATGAATTTAACAAGAATGTCCATGGCATTCTCACCCGAGAATGCCGTATTGAGG 300

5.11 GGCGGAATGAATTTAACAAGAATGTCCATGGCATTCTCACCCGAGAATGCCGTATTGAGG 300

************************************************************

MG637361.1 GACGTCGTATCCAGTGCTACAACAAAGTTACTGCTTAAAAACATGAGAGACCAAGTACTG 360

1.5 GACGTCGTATCCAGTGCTACAACAAAGTTACTGCTTAAAAACATGAGAGACCAAGTACTG 360

1.6 GACGTCGTATCCAGTGCTACAACAAAGTTACTGCTTAAAAACATGAGAGACCAAGTACTG 360

1.40 GACGTCGTATCCAGTGCTACAACAAAGTTACTGCTTAAAAACATGAGAGACCAAGTACTG 360

2.12 GACGTCGTATCCAGTGCTACAACAAAGTTACTGCTTAAAAACATGAGAGACCAAGTACTG 360

2.42 GACGTCGTATCCAGTGCTACAACAAAGTTACTGCTTAAAAACATGAGAGACCAAGTACTG 360

2.52 GACGTCGTATCCAGTGCTACAACAAAGTTACTGCTTAAAAACATGAGAGACCAAGTACTG 360

5.8 GACGTCGTATCCAGTGCTACAACAAAGTTACTGCTTAAAAACATGAGAGACCAAGTACTG 360

5.9 GACGTCGTATCCAGTGCTACAACAAAGTTACTGCTTAAAAACATGAGAGACCAAGTACTG 360

5.11 GACGTCGTATCCAGTGCTACAACAAAGTTACTGCTTAAAAACATGAGAGACCAAGTACTG 360

************************************************************

MG637361.1 CCCATCATTGAGGCATTGCCAATAGAAATACCGCCGGGACTGGTAAACTCGTCACAGGTG 420

1.5 CCCATCATTGAGGCATTGCCAATAGAAATACCGCCGGGACTGGTAAACTCGTCACAGGTG 420

1.6 CCCATCATTGAGGCATTGCCAATAGAAATACCGCCGGGACTGGTAAACTCGTCACAGGTG 420

1.40 CCCATCATTGAGGCATTGCCAATAGAAATACCGCCGGGACTGGTAAACTCGTCACAGGTG 420

2.12 CCCATCATTGAGGCATTGCCAATAGAAATACCGCCGGGACTGGTAAACTCGTCACAGGTG 420

2.42 CCCATCATTGAGGCATTGCCAATAGAAATACCGCCGGGACTGGTAAACTCGTCACAGGTG 420

2.52 CCCATCATTGAGGCATTGCCAATAGAAATACCGCCGGGACTGGTAAACTCGTCACAGGTG 420

5.8 CCCATCATTGAGGCATTGCCAATAGAAATACCGCCGGGACTGGTAAACTCGTCACAGGTG 420

5.9 CCCATCATTGAGGCATTGCCAATAGAAATACCGCCGGGACTGGTAAACTCGTCACAGGTG 420

5.11 CCCATCATTGAGGCATTGCCAATAGAAATACCGCCGGGACTGGTAAACTCGTCACAGGTG 420

************************************************************

MG637361.1 TACGAAATAGTTAAATTATTTGTCGACGAGAACGTTGTTACCGGATACAATAGCAGTGCG 480

1.5 TACGAAATAGTTAAATTATTTGTCGACGAGAACGTTGTTACCGGATACAATAGCAGTGCG 480

1.6 TACGAAATAGTTAAATTATTTGTCGACGAGAACGTTGTTACCGGATACAATAGCAGTGCG 480

1.40 TACGAAATAGTTAAATTATTTGTCGACGAGAACGTTGTTACCGGATACAATAGCAGTGCG 480

2.12 TACGAAATAGTTAAATTATTTGTCGACGAGAACGTTGTTACCGGATACAATAGCAGTGCG 480

2.42 TACGAAATAGTTAAATTATTTGTCGACGAGAACGTTGTTACCGGATACAATAGCAGTGCG 480

2.52 TACGAAATAGTTAAATTATTTGTCGACGAGAACGTTGTTACCGGATACAATAGCAGTGCG 480

5.8 TACGAAATAGTTAAATTATTTGTCGACGAGAACGTTGTTACCGGATACAATAGCAGTGCG 480

5.9 TACGAAATAGTTAAATTATTTGTCGACGAGAACGTTGTTACCGGATACAATAGCAGTGCG 480

5.11 TACGAAATAGTTAAATTATTTGTCGACGAGAACGTTGTTACCGGATACAATAGCAGTGCG 480

************************************************************

MG637361.1 GCAATGAGAGGAATATACGCAGAGGAAGAAGCCACGAGAAGGGTGATAGCTGGCATAGAA 540

1.5 GCAATGAGAGGAATATACGCAGAGGAAGAAGCCACGAGAAGGGTGATAGCTGGCATAGAA 540

1.6 GCAATGAGAGGAATATACGCAGAGGAAGAAGCCACGAGAAGGGTGATAGCTGGCATAGAA 540

1.40 GCAATGAGAGGAATATACGCAGAGGAAGAAGCCACGAGAAGGGTGATAGCTGGCATAGAA 540

2.12 GCAATGAGAGGAATATACGCAGAGGAAGAAGCCACGAGAAGGGTGATAGCTGGCATAGAA 540

2.42 GCAATGAGAGGAATATACGCAGAGGAAGAAGCCACGAGAAGGGTGATAGCTGGCATAGAA 540

2.52 GCAATGAGAGGAATATACGCAGAGGAAGAAGCCACGAGAAGGGTGATAGCTGGCATAGAA 540

5.8 GCAATGAGAGGAATATACGCAGAGGAAGAAGCCACGAGAAGGGTGATAGCTGGCATAGAA 540

5.9 GCAATGAGAGGAATATACGCAGAGGAAGAAGCCACAAGAAGGGTGATAGCTGGCATAGAA 540

5.11 GCAATGAGAGGAATATACGCAGAGGAAGAAGCCACGAGAAGGGTGATAGCTGGCATAGAA 540

*********************************** ************************

MG637361.1 TTCGATGACTCATTGCGTGAAATAACGGAGCTACCACTAGACTTGTCGTATGCGCTTCGT 600

1.5 TTCGATGACTCATTGCGTGAAATAACGGAGCTACCACTAGACTTGTCGTATGCGCTTCGT 600

1.6 TTCGATGACTCATTGCGTGAAATAACGGAGCTACCACTAGACTTGTCGTATGCGCTTCGT 600

1.40 TTCGATGACTCATTGCGTGAAATAACGGAGCTACCACTAGACTTGTCGTATGCGCTTCGT 600

2.12 TTCGATGACTCATTGCGTGAAATAACGGAGCTACCACTAGACTTGTCGTATGCGCTTCGT 600

2.42 TTCGATGACTCATTGCGTGAAATAACGGAGCTACCACTAGACTTGTCGTATGCGCTTCGT 600

2.52 TTCGATGACTCATTGCGTGAAATAACGGAGCTACCACTAGACTTGTCGTATGCGCTTCGT 600

5.8 TTCGATGACTCATTGCGTGAAATAACGGAGCTACCACTAGACTTGTCGTATGCGCTTCGT 600

5.9 TTCGATGACTCATTGCGTGAAATAACGGAGCTACCACTAGACTTGTCGTATGCGCTTCGT 600

5.11 TTCGATGACTCATTGCGTGAAATAACGGAGCTACCACTAGACTTGTCGTATGCGCTTCGT 600

************************************************************

MG637361.1 TTTCCGGAGAGACCTCGCTTGAATTCCTTCTTCATGACAGGCGGTCGGACTTGGCGCACA 660

1.5 TTTCCGGAGAGACCTCGCTTGAATTCCTTCTTCATGACAGGCGGTCGGACTTGGCGCACA 660

1.6 TTTCCGGAGAGACCTCGCTTGAATTCCTTCTTCATGACAGGCGGTCGGACTTGGCGCACA 660

1.40 TTTCCGGAGAGACCTCGCTTGAATTCCTTCTTCATGACAGGCGGTCGGACTTGGCGCACA 660

2.12 TTTCCGGAGAGACCTCGCTTGAATTCCTTCTTCATGACAGGCGGTCGGACTTGGCGCACA 660

2.42 TTTCCGGAGAGACCTCGCTTGAATTCCTTCTTCATGACAGGCGGTCGGACTTGGCGCACA 660

2.52 TTTCCGGAGAGACCTCGCTTGAATTCCTTCTTCATGACAGGCGGTCGGACTTGGCGCACA 660

5.8 TTTCCGGAGAGACCTCGCTTGAATTCCTTCTTCATGACAGGCGGTCGGACTTGGCGCACA 660

5.9 TTTCCGGAGAGACCTCGCTTGAATTCCTTCTTCATGACAGGCGGTCGGACTTGGCGCACA 660

5.11 TTTCCGGAGAGACCTCGCTTGAATTCCTTCTTCATGACAGGCGGTCGGACTTGGCGCACA 660

************************************************************

MG637361.1 GATAACGTGTTTCCTATGTTCGAAGTTCCCGGACCTCGCTTTCCGTATTCATGGGAAGGT 720

1.5 GATAACGTGTTTCCTATGTTCGAAGTTCCCGGACCTCGCTTTCCGTATTCATGGGAAGGT 720

1.6 GATAACGTGTTTCCTATGTTCGAAGTTCCCGGACCTCGCTTTCCGTATTCATGGGAAGGT 720

1.40 GATAACGTGTTTCCTATGTTCGAAGTTCCCGGACCTCGCTTTCCGTATTCATGGGAAGGT 720

2.12 GATAACGTGTTTCCTATGTTCGAAGTTCCCGGACCTCGCTTTCCGTATTCATGGGAAGGT 720

2.42 GATAACGTGTTTCCTATGTTCGAAGTTCCCGGACCTCGCTTTCCGTATTCATGGGAAGGT 720

2.52 GATAACGTGTTTCCTATGTTCGAAGTTCCCGGACCTCGCTTTCCGTATTCATGGGAAGGT 720

5.8 GATAACGTGTTTCCTATGTTCGAAGTTCCCGGACCTCGCTTTCCGTATTCATGGGAAGGT 720

5.9 GATAACGTGTTTCCTATGTTCGAAGTTCCCGGACCTCGCTTTCCGTATTCATGGGAAGGT 720

5.11 GATAACGTGTTTCCTATGTTCGAAGTTCCCGGACCTCGCTTTCCGTATTCATGGGAAGGT 720

************************************************************

MG637361.1 GGAAATGATCCAGGATACGTAAACGAGATGTTCATAGCCTTGCAGCACATGATATCTTCA 780

1.5 GGAAATGATCCAGGATACGTAAACGAGATGTTCATAGCCTTGCAGCACATGATATCTTCA 780

1.6 GGAAATGATCCAGGATACGTAAACGAGATGTTCATAGCCTTGCAGCACATGATATCTTCA 780

1.40 GGAAATGATCCAGGATACGTAAACGAGATGTTCATAGCCTTGCAGCACATGATATCTTCA 780

2.12 GGAAATGATCCAGGATACGTAAACGAGATGTTCATAGCCTTGCAGCACATGATATCTTCA 780

2.42 GGAAATGATCCAGGATACGTAAACGAGATGTTCATAGCCTTGCAGCACATGATATCTTCA 780

2.52 GGAAATGATCCAGGATACGTAAACGAGATGTTCATAGCCTTGCAGCACATGATATCTTCA 780

5.8 GGAAATGATCCAGGATACGTAAACGAGATGTTCATAGCCTTGCAGCACATGATATCTTCA 780

5.9 GGAAATGATCCAGGATACGTAAACGAGATGTTCATAGCCTTGCAGCACATGATATCTTCA 780

5.11 GGAAATGATCCAGGATACGTAAACGAGATGTTCATAGCCTTGCAGCACATGATATCTTCA 780

************************************************************

MG637361.1 GAACTGGTATCTAAAGTGGCGGGAGTGAACCTAGACTTCGATGTGCACATACAGAGGTAC 840

1.5 GAACTGGTATCTAAAGTGGCGGGAGTGAACCTAGACTTCGATGTGCACATACAGAGGTAC 840

1.6 GAACTGGTATCTAAAGTGGCGGGAGTGAACCTAGACTTCGATGTGCACATACAGAGGTAC 840

1.40 GAACTGGTATCTAAAGTGGCGGGAGTGAACCTAGACTTCGATGTGCACATACAGAGGTAC 840

2.12 GAACTGGTATCTAAAGTGGCGGGAGTGAACCTAGACTTCGATGTGCACATACAGAGGTAC 840

2.42 GAACTGGTATCTAAAGTGGCGGGAGTGAACCTAGACTTCGATGTGCACATACAGAGGTAC 840

2.52 GAACTGGTATCTAAAGTGGCGGGAGTGAACCTAGACTTCGATGTGCACATACAGAGGTAC 840

5.8 GAACTGGTATCTAAAGTGGCGGGAGTGAACCTAGACTTCGATGTGCACATACAGAGGTAC 840

5.9 GAACTGGTATCTAAAGTGGCGGGAGTGAACCTAGACTTCGATGTGCACATACAGAGGTAC 840

5.11 GAACTGGTATCTAAAGTGGCGGGAGTGAACCTAGACTTCGATGTGCACATACAGAGGTAC 840

************************************************************

MG637361.1 CCACATCCAGCATACATCATGGACTTGGCGAAGGAAGCCCTGCAGTTCCTCTTCCCATCA 900

1.5 CCACATCCAGCATACATCATGGACTTGGCGAAGGAAGCCCTGCAGTTCCTCTTCCCATCA 900

1.6 CCACATCCAGCATACATCATGGACTTGGCGAAGGAAGCCCTGCAGTTCCTCTTCCCATCA 900

1.40 CCACATCCAGCATACATCATGGACTTGGCGAAGGAAGCCCTGCAGTTCCTCTTCCCATCA 900

2.12 CCACATCCAGCATACATCATGGACTTGGCGAAGGAAGCCCTGCAGTTCCTCTTCCCATCA 900

2.42 CCACATCCAGCATACATCATGGACTTGGCGAAGGAAGCCCTGCAGTTCCTCTTCCCATCA 900

2.52 CCACATCCAGCATACATCATGGACTTGGCGAAGGAAGCCCTGCAGTTCCTCTTCCCATCA 900

5.8 CCACATCCAGCATACATCATGGACTTGGCGAAGGAAGCCCTGCAGTTCCTCTTCCCATCA 900

5.9 CCACATCCAGCATACATCATGGACTTGGCGAAGGAAGCCCTGCAGTTCCTCTTCCCATCA 900

5.11 CCACATCCAGCATACATCATGGACTTGGCGAAGGAAGCCCTGCAGTTCCTCTTCCCATCA 900

************************************************************

MG637361.1 TTCATCATGATCAGCTTCAGTTACACCGCTATCAATATTATACGATCCGTGACCGTGGAA 960

1.5 TTCATCATGATCAGCTTCAGTTACACCGCTATCAATATTATACGATCCGTGACCGTGGAA 960

1.6 TTCATCATGATCAGCTTCAGTTACACCGCTATCAATATTATACGATCCGTGACCGTGGAA 960

1.40 TTCATCATGATCAGCTTCAGTTACACCGCTATCAATATTATACGATCCGTGACCGTGGAA 960

2.12 TTCATCATGATCAGCTTCAGTTACACCGCTATCAATATTATACGATCCGTGACCGTGGAA 960

2.42 TTCATCATGATCAGCTTCAGTTACACCGCTATCAATATTATACGATCCGTGACCGTGGAA 960

2.52 TTCATCATGATCAGCTTCAGTTACACCGCTATCAATATTATACGATCCGTGACCGTGGAA 960

5.8 TTCATCATGATCAGCTTCAGTTACACCGCTATCAATATTATACGATCCGTGACCGTGGAA 960

5.9 TTCATCATGATCAGCTTCAGTTACACCGCTATCAATATTATACGATCCGTGACCGTGGAA 960

5.11 TTCATCATGATCAGCTTCAGTTACACCGCTATCAATATTATACGATCCGTGACCGTGGAA 960

************************************************************

MG637361.1 AAAGAAATGCAATTGAAGGAAACGATGAAGATCATGGGACTCCCAACGTGGCTGCATTGG 1020

1.5 AAAGAAATGCAATTGAAGGAAACGATGAAGATCATGGGACTCCCAACGTGGCTGCATTGG 1020

1.6 AAAGAAATGCAATTGAAGGAAACGATGAAGATCATGGGACTCCCAACGTGGCTGCATTGG 1020

1.40 AAAGAAATGCAATTGAAGGAAACGATGAAGATCATGGGACTCCCAACGTGGCTGCATTGG 1020

2.12 AAAGAAATGCAATTGAAGGAAACGATGAAGATCATGGGACTCCCAACGTGGCTGCATTGG 1020

2.42 AAAGAAATGCAATTGAAGGAAACGATGAAGATCATGGGACTCCCAACGTGGCTGCATTGG 1020

2.52 AAAGAAATGCAATTGAAGGAAACGATGAAGATCATGGGACTCCCAACGTGGCTGCATTGG 1020

5.8 AAAGAAATGCAATTGAAGGAAACGATGAAGATCATGGGACTCCCAACGTGGCTGCATTGG 1020

5.9 AAAGAAATGCAATTGAAGGAAACGATGAAGATCATGGGACTCCCAACGTGGCTGCATTGG 1020

5.11 AAAGAAATGCAATTGAAGGAAACGATGAAGATCATGGGACTCCCAACGTGGCTGCATTGG 1020

************************************************************

MG637361.1 ATGGCATGGTTTTTTAAACAATTTATTTATTTGCTGATTGCTTCGGTTTTGATACTTGTT 1080

1.5 ATGGCATGGTTTTTTAAACAATTTATTTATTTGCTAATTGCTTCGGTTTTGATACTTGTT 1080

1.6 ATGGCATGGTTTTTTAAACAATTTATTTATTTGCTAATTGCTTCGGTTTTGATACTTGTT 1080

1.40 ATGGCATGGTTTTTTAAACAATTTATTTATTTGCTAATTGCTTCGGTTTTGATACTTGTT 1080

2.12 ATGGCATGGTTTTTTAAACAATTTATTTATTTGCTAATTGCTTCGGTTTTGATACTTGTT 1080

2.42 ATGGCATGGTTTTTTAAACAATTTATTTATTTGCTAATTGCTTCGGTTTTGATACTTGTT 1080

2.52 ATGGCATGGTTTTTTAAACAATTTATTTATTTGCTAATTGCTTCGGTTTTGATACTTGTT 1080

5.8 ATGGCATGGTTTTTTAAACAATTTATTTATTTGCTAATTGCTTCGGTTTTGATACTTGTT 1080

5.9 ATGGCATGGTTTTTTAAACAATTTATTTATTTGCTAATTGCTTCGGTTTTGATACTTGTT 1080

5.11 ATGGCATGGTTTTTTAAACAATTTATTTATTTGCTAATTGCTTCGGTTTTGATACTTGTT 1080

*********************************** ************************

MG637361.1 ATATTAAAGGTAAATTGGTTTACTACAGAAGAAGGCTTTAGCGACTATGCCGTATTCACT 1140

1.5 ATATTAAAGGTAAATTGGTTTACTACAGAAGAAGGCTTTAGCGACTATGCCGTATTCACT 1140

1.6 ATATTAAAGGTAAATTGGTTTACTACAGAAGAAGGCTTTAGCGACTATGCCGTATTCACT 1140

1.40 ATATTAAAGGTAAATTGGTTTACTACAGAAGAAGGCTTTAGCGACTATGCCGTATTCACT 1140

2.12 ATATTAAAGGTAAATTGGTTTACTACAGAAGAAGGCTTTAGCGACTATGCCGTATTCACT 1140

2.42 ATATTAAAG--------------------------------------------------- 1089

2.52 ATATTAAAG--------------------------------------------------- 1089

5.8 ATATTAAAGGTAAATTGGTTTACTACAGAAGAAGGCTTTAGCGACTATGCCGTATTCACT 1140

5.9 ATATTAAAGGTAAATTGGTTTACTACAGAAGAAGGCTTTAGCGACTATGCCGTATTCACT 1140

5.11 ATATTAAAG--------------------------------------------------- 1089

*********

MG637361.1 AATACACCTTGGACCGTCCTCTTCTTCTTCCTAACACTGTATCTTACGTGTACCATATTT 1200

1.5 AATACACCTTGGACCGTCCTCTTCTTCTTCCTAACACTGTATCTTACGTGTACCATATTT 1200

1.6 AATACACCTTGGACCGTCCTCTTCTTCTTCCTAACACTGTATCTTACGTGTACCATATTT 1200

1.40 AATACACCTTGGACCGTCCTCTTCTTCTTCCTAACACTGTATCTTACGTGTACCATATTT 1200

2.12 AATACACCTTGGACCGTCCTCTTCTTCTTCCTAACACTGTATCTTACGTGTACCATATTT 1200

2.42 ------------------------------------------------------------ 1089

2.52 ------------------------------------------------------------ 1089

5.8 AATACACCTTGGACCGTCCTCTTCTTCTTCCTAACACTGTATCTTACGTGTACCATATTT 1200

5.9 AATACACCTTGGACCGTCCTCTTCTTCTTCCTAACACTGTATCTTACGTGTACCATATTT 1200

5.11 ------------------------------------------------------------ 1086

MG637361.1 TTCTGTTTCATGATAAGTGGTTTCTTTTCAAAAGCCAGTACAGCGGCGTTGTTTGGTGGG 1260

1.5 TTCTGTTTCATGATAAGTGGTTTCTTTTCAAAAGCCAGTACAGCGGCGTTGTTTGGTGGG 1260

1.6 TTCTGTTTCATGATAAGTGGTTTCTTTTCAAAAGCCAGTACAGCGGCGTTGTTTGGTGGG 1260

1.40 TTCTGTTTCATGATAAGTGGTTTCTTTTCAAAAGCCAGTACAGCGGCGTTGTTTGGTGGG 1260

2.12 TTCTGTTTCATGATAAGTGGTTTCTTTTCAAAAGCCAGTACAGCGGCGTTGTTTGGTGGG 1260

2.42 ----------------------------------CCAGTACAGCGGCGTTGTTTGGTGGG 1115

2.52 ----------------------------------CCAGTACAGCGGCGTTGTTTGGTGGG 1115

5.8 TTCTGTTTCATGATAAGTGGTTTCTTTTCAAAAGCCAGTACAGCGGCGTTGTTTGGTGGG 1260

5.9 TTCTGTTTCATGATAAGTGGTTTCTTTTCAAAAGCCAGTACAGCGGCGTTGTTTGGTGGG 1260

5.11 ----------------------------------CCAGTACAGCGGCGTTGTTTGGTGGG 1115

**************************

MG637361.1 GTGATCTGGTTTCTGACGTATATCCCCGCATTCCTCCTGGCTATGGACGTGAACATGTCT 1320

1.5 GTGATCTGGTTTCTGACGTATATCCCCGCATTCCTCCTGGCTATGGACGTGAACATGTCT 1320

1.6 GTGATCTGGTTTCTGACGTATATCCCCGCATTCCTCCTGGCTATGGACGTGAACATGTCT 1320

1.40 GTGATCTGGTTTCTGACGTATATCCCCGCATTCCTCCTGGCTATGGACGTGAACATGTCT 1320

2.12 GTGATCTGGTTTCTGACGTATATCCCCGCATTCCTCCTGGCTATGGACGTGAACATGTCT 1320

2.42 GTGATCTGGTTTCTGACGTATATCCCCGCATTCCTCCTGGCTATGGACGTGAACATGTCT 1175

2.52 GTGATCTGGTTTCTGACGTATATCCCCGCATTCCTCCTGGCTATGGACGTGAACATGTCT 1175

5.8 GTGATCTGGTTTCTGACGTATATCCCCGCATTCCTCCTGGCTATGGACGTGAACATGTCT 1320

5.9 GTGATCTGGTTTCTGACGTATATCCCCGCATTCCTCCTGGCTATGGACGTGAACATGTCT 1320

5.11 GTGATCTGGTTTCTGACGTATATCCCCGCATTCCTCCTGGCTATGGACGTGAACATGTCT 1175

************************************************************

MG637361.1 ACCTCTCTACAAGCGGTCACCTGCCTAATGCTCAACTCCGCCATGTCTTACGGCTTCCAG 1380

1.5 ACCTCTCTACAAGCGGTCACCTGCCTAATGCTCAACTCCGCCATGTCTTACGGCTTCCAG 1380

1.6 ACCTCTCTACAAGCGGTCACCTGCCTAATGCTCAACTCCGCCATGTCTTACGGCTTCCAG 1380

1.40 ACCTCTCTACAAGCGGTCACCTGCCTAATGCTCAACTCCGCCATGTCTTACGGCTTCCAG 1380

2.12 ACCTCTCTACAAGCGGTCACCTGCCTAATGCTCAACTCCGCCATGTCTTACGGCTTCCAG 1380

2.42 ACCTCTCTACAAGCGGTCACCTGCCTAATGCTCAACTCCGCCATGTCTTACGGCTTCCAG 1235

2.52 ACCTCTCTACAAGCGGTCACCTGCCTAATGCTCAACTCCGCCATGTCTTACGGCTTCCAG 1235

5.8 ACCTCTCTACAAGCGGTCACCTGCCTAATGCTCAACTCCGCCATGTCTTACGGCTTCCAG 1380

5.9 ACCTCTCTACAAGCGGTCACCTGCCTAATGCTCAACTCCGCCATGTCTTACGGCTTCCAG 1380

5.11 ACCTCTCTACAAGCGGTCACCTGCCTAATGCTCAACTCCGCCATGTCTTACGGCTTCCAG 1235

************************************************************

MG637361.1 CTGTTACTGGCCCGGGAAAGTACCGGAGGAATGCAGTGGGGTGATTTTATGACGTCACCA 1440

1.5 CTGTTACTGGCCCGGGAAAGTACCGGAGGAATGCAGTGGGGTGATTTTATGACGTCACCA 1440

1.6 CTGTTACTGGCCCGGGAAAGTACCGGAGGAATGCAGTGGGGTGATTTTATGACGTCACCA 1440

1.40 CTGTTACTGGCCCGGGAAAGTACCGGAGGAATGCAGTGGGGTGATTTTATGACGTCACCA 1440

2.12 CTGTTACTGGCCCGGGAAAGTACCGGAGGAATGCAGTGGGGTGATTTTATGACGTCACCA 1440

2.42 CTGTTACTGGCCCGGGAAAGTACCGGAGGAATGCAGTGGGGTGATTTTATGACGTCACCA 1295

2.52 CTGTTACTGGCCCGGGAAAGTACCGGAGGAATGCAGTGGGGTGATTTTATGACGTCACCA 1295

5.8 CTGTTACTGGCCCGGGAAAGTACCGGAGGAATGCAGTGGGGTGATTTTATGACGTCACCA 1440

5.9 CTGTTACTGGCCCGGGAAAGTACCGGAGGAATGCAGTGGGGTGATTTTATGACGTCACCA 1440

5.11 CTGTTACTGGCCCGGGAAAGTACCGGAGGAATGCAGTGGGGTGATTTTATGACGTCACCA 1295

************************************************************

MG637361.1 GCAACGGACTCGTCACGATTCGTATTCGGTCACGTCGTTATAATGATGGCTTTGAACTGT 1500

1.5 GCAACGGACTCGTCACGATTCGTATTCGGTCACGTCGTTATAATGATGGCTTTGAACTGT 1500

1.6 GCAACGGACTCGTCACGATTCGTATTCGGTCACGTCGTTATAATGATGGCTTTGAACTGT 1500

1.40 GCAACGGACTCGTCACGATTCGTATTCGGTCACGTCGTTATAATGATGGCTTTGAACTGT 1500

2.12 GCAACGGACTCGTCACGATTCGTATTCGGTCACGTCGTTATAATGATGGCTTTGAACTGT 1500

2.42 GCAACGGACTCGTCACGATTCGTATTCGGTCACGTCGTTATAATGATGGCTTTGAACTGT 1355

2.52 GCAACGGACTCGTCACGATTCGTATTCGGTCACGTCGTTATAATGATGGCTTTGAACTGT 1355

5.8 GCAACGGACTCGTCACGATTCGTATTCGGTCACGTCGTTATAATGATGGCTTTGAACTGT 1500

5.9 GCAACGGACTCGTCACGATTCGTATTCGGTCACGTCGTTATAATGATGGCTTTGAACTGT 1500

5.11 GCAACGGACTCGTCACGATTCGTATTCGGTCACGTCGTTATAATGATGGCTTTGAACTGT 1355

************************************************************

MG637361.1 GTGCTCTACATGTTGATTGCCCTATATCTAGAGCAAGTACTACCCGGGCCGTATGGCACA 1560

1.5 GTGCTCTACATGTTGATTGCCCTATATCTAGAGCAAGTACTACCCGGGCCGTATGGCACA 1560

1.6 GTGCTCTACATGTTGATTGCCCTATATCTAGAGCAAGTACTACCCGGGCCGTATGGCACA 1560

1.40 GTGCTCTACATGTTGATTGCCCTATATCTAGAGCAAGTACTACCCGGGCCGTATGGCACA 1560

2.12 GTGCTCTACATGTTGATTGCCCTATATCTAGAGCAAGTACTACCCGGGCCGTATGGCACA 1560

2.42 GTGCTCTACATGTTGATTGCCCTATATCTAGAGCAAGTACTACCCGGGCCGTATGGCACA 1415

2.52 GTGCTCTACATGTTGATTGCCCTATATCTAGAGCAAGTACTACCCGGGCCGTATGGCACA 1415

5.8 GTGCTCTACATGTTGATTGCCCTATATCTAGAGCAAGTACTACCCGGGCCGTATGGCACA 1560

5.9 GTGCTCTACATGTTGATTGCCCTATATCTAGAGCAAGTACTACCCGGGCCGTATGGCACA 1560

5.11 GTGCTCTACATGTTGATTGCCCTATATCTAGAGCAAGTACTACCCGGGCCGTATGGCACA 1415

************************************************************

MG637361.1 CCGAAGCCCTGGTATTTCTTCGTCCAAAGACAGTTCTGGTGTAGCAGCAAAACTACTCAT 1620

1.5 CCGAAGCCCTGGTATTTCTTCGTCCAAAGACAGTTCTGGTGTAGCAGCAAAACTACTCAT 1620

1.6 CCGAAGCCCTGGTATTTCTTCGTCCAAAGACAGTTCTGGTGTAGCAGCAAAACTACTCAT 1620

1.40 CCGAAGCCCTGGTATTTCTTCGTCCAAAGACAGTTCTGGTGTAGCAGCAAAACTACTCAT 1620

2.12 CCGAAGCCCTGGTATTTCTTCGTCCAAAGACAGTTCTGGTGTAGCAGCAAAACTACTCAT 1620

2.42 CCGAAGCCCTGGTATTTCTTCGTCCAAAGACAGTTCTGGTGTAGCAGCAAAACTACTCAT 1475

2.52 CCGAAGCCCTGGTATTTCTTCGTCCAAAGACAGTTCTGGTGTAGCAGCAAAACTACTCAT 1475

5.8 CCGAAGCCCTGGTATTTCTTCGTCCAAAGACAGTTCTGGTGTAGCAGCA-AACTACTCAT 1619

5.9 CCGAAGCCCTGGTATTTCTTCGTCCAAAGACAGTTCTGGTGTAGCAGCAAAACTACTCAT 1620

5.11 CCGAAGCCCTGGTATTTCTTCGTCCAAAGACAGTTCTGGTGTAGCAGCAAAACTACTCAT 1475

************************************************* **********

MG637361.1 GATATCGGTACAGACAACAGCGACACATCAAGTTTAACAAAAGAAAGCGACCCTACAGAC 1680

1.5 GATATCGGTACAGACAACAGCGACACATCAAGTTTAACAAAAGAAAGCGACCCTACAGAC 1680

1.6 GATATCGGTACAGACAACAGCGACACATCAAGTTTAACAAAAGAAAGCGACCCTACAGAC 1680

1.40 GATATCGGTACAGACAACAGCGACACATCAAGTTTAACAAAAGAAAGCGACCCTACAGAC 1680

2.12 GATATCGGTACAGACAACAGCGACACATCAAGTTTAACAAAAGAAAGCGACCCTACAGAC 1680

2.42 G--ATCGGTACAGACAACAGCGACACATCAAGTTTAACAAAAGAAAGCGACCCTACAGAC 1533

2.52 G--ATCGGTACAGACAACAGCGACACATCAAGTTTAACAAAAGAAAGCGACCCTACAGAC 1533

5.8 GATATCGGTACAGACAACAGCGACACATCAAGTTTAACAAAAGAAAGCGACCCTACAGAC 1679

5.9 GATATCGGTACAGACAACAGCGACACATCAAGTTTAACAAAAGAAAGCGACCCTACAGAC 1680

5.11 GATATCGGTACAGACAACAGCGACACATCAAGTTTAACAAAAGAAAGCGACCCTACAGAC 1535

* *********************************************************

MG637361.1 CTTCCGATTGGAGTTAAAATACAAAACCTTAAAAAGGTTTACGGGAGCAACGTTGCGGTA 1740

1.5 CTTCCGATTGGAGTTAAAATACAAAACCTTAAAAAGGTTTACGGGAGCAACGTTGCGGTA 1740

1.6 CTTCCGATTGGAGTTAAAATACAAAACCTTAAAAAGGTTTACGGGAGCAACGTTGCGGTA 1740

1.40 CTTCCGATTGGAGTTAAAATACAAAACCTTAAAAAGGTTTACGGGAGCAACGTTGCGGTA 1740

2.12 CTTCCGATTGGAGTTAAAATACAAAACCTTAAAAAGGTTTACGGGAGCAACGTTGCGGTA 1740

2.42 CTTCCGATTGGAGTTAAAATACAAAACCTTAAAAAGGTTTACGGGAGCAACGTTGCGGTA 1593

2.52 CTTCCGATTGGAGTTAAAATACAAAACCTTAAAAAGGTTTACGGGAGCAACGTTGCGGTA 1593

5.8 CTTCCGATTGGAGTTAAAATACAAAACCTTAAAAAGGTTTACGGGAGCAACGTTGCGGTA 1739

5.9 CTTCCGATTGGAGTTAAAATACAAAACCTTAAAAAGGTTTACGGGAGCAACGTTGCGGTA 1740

5.11 CTTCCGATTGGAGTTAAAATACAAAACCTTAAAAAGGTTTACGGGAGCAACGTTGCGGTA 1595

************************************************************

MG637361.1 AACAATTTATCCCTCAACATTTACGACGACCAAATCACGGTTCTACTTGGACACAACGGA 1800

1.5 AACAATTTATCCCTCAATATTTACGACGACCAAATCACGGTTCTAAC------------- 1787

1.6 AACAATTTATCCCTCAATATTTACGACGACCAAATCACGGTTCTACTTGGACACAACGGA 1800

1.40 AACAATTTATCCCTCAATATTTACGACGACCAAATCACGGTTCTACTTGGACACAACGGA 1800

2.12 AACAATTTATCCCTCAATATTTACGACGACCAAATCACGGTTCTACTTGGACACAACGGA 1800

2.42 AACAATTTATCCCTCAATATTTACGACGACCAAATCACGGTTCTACTTGGACACAACGGA 1653

2.52 AACAATTTATCCCTCAATATTTACGACGACCAAATCACGGTTCTACTTGGACACAACGGA 1653

5.8 AACAATTTATCCCTCAATATTTACGACGACCAAATCACGGTTCTACTTGGACACAACGGA 1799

5.9 AACAATTTATCCCTCAATATTTACGACGACCAAATCACGGTTCTACTTGGACACAACGGA 1800

5.11 AACAATTTATCCCTCAATATTTACGACGACCAAATCACGGTTCTACTTGGACACAACGGA 1655

***************** ***************************

MG637361.1 GCGGGAAAATCCACAACCATTTCAATGCTCACAGGTAACGTGGACATAACCAGCGGGTCG 1860

1.5 ---------------------------------------------ATAACCAGCGGGTCG 1802

1.6 GCGGGAAAATCCACGACCATTTCAATGCTCACAGGTAACGTGGACATAACCAGCGGGTCG 1860

1.40 GCGGGAAAATCCACGACCATTTCAATGCTCACAGGTAACGTGGACATAACCAGCGGGTCG 1860

2.12 GCGGGAAAATCCACGACCATTTCAATGCTCACAGGTAACGTGGACATAACCAGCGGGTCG 1860

2.42 GCGGGAAAATCCACGACCATTTCAATGCTCACAGGTAACGTGGACATAACCAGCGGGTCG 1713

2.52 GCGGGAAAATCCACGACCATTTCAATGCTCACAGGTAACGTGGACATAACCAGCGGGTCG 1713

5.8 GCGGGAAAATCCACGACCATTTCAATGCTCACAGGTAACGTGGACATAACCAGCGGGTCG 1859

5.9 GCGGGAAAATCCACGACCATTTCAATGCTCACA-------------------GCGGGTCG 1841

5.11 GCGGGAAAATCCACGACCATTTCAATGCTCACAGGTAACGTGGACATAACCAGCGGGTCG 1715

********

MG637361.1 GTGACGGTGGCTGGCTACGACATAGAAAAACAAACAAGTTCAGCACGCTCACACATTGGA 1920

1.5 GTGACGGTGGCTGGCTACGACATAGAAAAACAAACAAGTTCAGCACGCTCACACATTGGA 1862

1.6 GTGACGGTGGCTGGCTACGACATAGAAAAACAAACAAGTTCAGCAC-------------- 1906

1.40 GTGACGGTGGCTGGCTACGACATAGAAAAACAAACAAGTTCAGCACGCTCACACATTGGA 1920

2.12 GTGACGGTGGCTGGCTACGACATAGAAAAACAAACAAGTTCAGCACGCTCACACATTGGA 1920

2.42 GTGACGGTGGCTGGCTACGACATAGAAAAACAAACAAGTTCAGCACGCTCACACATTGGA 1773

2.52 GTGACGGTGGCTGGCTACGACATAGAAAAACAAACAAGTTCAGCACGCTCACACATTGGA 1773

5.8 GTGACGGTGGCTGGCTACGACATAGAAAAACAAACAAGTTCAGCACGCTCACACATTGGA 1919

5.9 GTGACGGTGGCTGGCTACGACATAGAAAAACAAACAAGTTCAGCACGCTCACACATTGGA 1901

5.11 GTGACGGTGGCTGGCTACGACATAGAAAAACAAACAAGTTCAGCACGCTCACACATTGGA 1775

**********************************************

MG637361.1 CTCTGCCCTCAACATAACGTACTCTTCAACGAACTCACAGTCAAAGAACATTTACAGTTC 1980

1.5 CTCTGCCCTCAACATAACGTACTCTTCAACGAACTCACAGTCAAAGAACATTTACAGTTC 1922

1.6 ------------------------------------------------------------ 1906

1.40 CTCTGCCCTCAACATAACGTACTCTTCAACGAACTCACAGTCAA-GAACATTTACAGTTC 1979

2.12 CTCTGCCCTCAACATAACGTACTCTTCAACGAACTCACAGTCAAAGAACATTTACAGTTC 1980

2.42 CTCTGCCCTCAACATAACGTACTCTTCAACGAACTCACAGTCAAAGAACATTTACAGTTC 1833

2.52 CTCTGCCCTCAACATAACGTACTCTTCAACGAACTCACAGTCAAAGAACATTTACAGTTC 1833

5.8 CTCTGCCCTCAACATAACGTACTCTTCAACGAACTCACAGTCAAAGAACATTTACAGTTC 1979

5.9 CTCTGCCCTCAACATAACGTACTCTTCAACGAACTCACAGTCAAAGAACATTTACAGTTC 1961

5.11 CTCTGCCCTCAACATAACGTACTCTTCAACGAACTCACAGTCAAAGAACATTTACAGTTC 1835

MG637361.1 TTCTCTCGTCTGAAAGGCTTCAGCGGTAAAGAGTTGGATGAAGAAATTGAGACGCTTATT 2040

1.5 TTCTCTCGTCTGAAAGGCTTCAGCGGTAAAGAGTTGGATGAAGAAATTGTGACGCTTATT 1982

1.6 -----------------------------------------------------GCTTATT 1913

1.40 TTCTCTCGTCTGAAAGGCTTCAGCGGTAAAGAGTTGGATGAAGAAATTGTGACGCTTATT 2039

2.12 TTCTCTCGTCTGAAAGGCTTCAGCGGTAAAGAGTTGGATGAAGAAATTGTGACGCTTATT 2040

2.42 TTCTCTCGTCTGAAAGGCTTCAGCGGTAAAGAGTTGGATGAAGAAATTGTGACGCTTATT 1893

2.52 TTCTCTCGTCTGAAAGGCTTCAGCGGTAAAGAGTTGGATGAAGAAATTGTGACGCTTATT 1893

5.8 TTCTCTCGTCTGAAAGGCTTCAGCGGTAAAGAGTTGGATGAAGAAATTGTGACGCTTATT 2039

5.9 TTCTCTCGTCTGAAAGGCTTCAGCGGTAAAGAGTTGGATGAAGAAATTGTGACGCTTATT 2021

5.11 TTCTCTCGTCTGAAAGGCTTCAGCGGTAAAGAGTTGGATGAAGAAATTGTGACGCTTATT 1895

*******

MG637361.1 GAAAAATTGGAATTGCAAGAAAAGAGGGATTACCAATCAGCGGGGTTATCAGGGGGACAG 2100

1.5 GAAAAATTGGAATTGCAAGAAAAGAGGGATTACCAATCAGCGGGATTATCAGGGGGACAG 2042

1.6 GAAAAATTGGAATTGCAAGAAAAGAGGGATTACCAATCAGCGGGATTATCAGGGGGACAG 1973

1.40 GAAAAATTGGAATTGCAAGAAAAGAGGGATTACCAATCAGCGGGATTATCAGGGGGACAG 2099

2.12 GAAAAATTGGAATTGCAAGAAAAGAGGGATTACCAATCAGCGGGATTATCAGGGGGACAG 2100

2.42 GAAAAATTGGAATTGCAAGAAAAGAGGGATTACCAATCAGCGGGATTATCAGGGGGACAG 1953

2.52 GAAAAATTGGAATTGCAAGAAAAGAGGGATTACCAATCAGCGGGATTATCAGGGGGACAG 1953

5.8 GAAAAATTGGAATTGCAAGAAAAGAGGGATTACCAATCAGCGGGATTATCAGGGGGACAG 2099

5.9 GAAAAATTGGAATTGCAAGAAAAGAGGGATTACCAATCAGCGGGATTATCAGGGGGACAG 2081

5.11 GAAAAATTGGAATTGCAAGAAAAGAGGGATTACCAATCAGCGGGATTATCAGGGGGACAG 1955

******************************************** ***************

MG637361.1 AAGCGACGATTAGGAGTGGGCGTCGCGCTATGCGGGGCGGCTAAAGTGGTTCTACTGGAC 2160

1.5 AAGCGACGACTAGGAGTGGGCGTCGCGCTATGCGGGGCGGCTAAAGTGGTTCTACTGGAC 2102

1.6 AAGCGACGACTAGGAGTGGGCGTCGCGCTATGCGGGGCGGCTAAAGTGGTTCTACTGGAC 2033

1.40 AAGCGACGACTAGGAGTGGGCGTCGCGCTATGCGGGGCGGCTAAAGTGGTTCTACTGGAC 2159

2.12 AAGCGACGACTAGGAGTGGGCGTCGCGCTATGCGGGGCGGCTAAAGTGGTTCTACTGGAC 2160

2.42 AAGCGACGACTAGGAGTGGGCGTCGCGCTATGCGGGGCGGCTAAAGTGGTTCTACTGGAC 2013

2.52 AAGCGACGACTAGGAGTGGGCGTCGCGCTATGCGGGGCGGCTAAAGTGGTTCTACTGGAC 2013

5.8 AAGCGACGACTAGGAGTGGGCGTCGCGCTATGCGGGGCGGCTAAAGTGGTTCTACTGGAC 2159

5.9 AAGCGACGACTAGGAGTGGGCGTCGCGCTATGCGGGGCGGCTAAAGTGGTTCTACTGGAC 2141

5.11 AAGCGACGACTAGGAGTGGGCGTCGCGCTATGCGGGGCGGCTAAAGTGGTTCTACTGGAC 2015

********* **************************************************

MG637361.1 GAGCCCACTTCTGGCATGGACCCGGCCTCACGTCGTGCCCTATGGGACTTGTTGCAGAGA 2220

1.5 GAGCCCACTTCTGGCATGGACCCGGCCTCACGTCGTGCCCTGTGGGACTTGTTGCAGAGA 2162

1.6 GAGCCCACTTCTGGCATGGACCCGGCCTCACGTCGTGCCCTGTGGGACTTGTTGCAGAGA 2093

1.40 GAGCCCACTTCTGGCATGGACCCGGCCTCACGTCGTGCCCTGTGGGACTTGTTGCAGAGA 2219

2.12 GAGCCCACTTCTGGCATGGACCCGGCCTCACGTCGTGCCCTGTGGGACTTGTTGCAGAGA 2220

2.42 GAGCCCACTTCTGGCATGGACCCGGCCTCACGTCGTGCCCTGTGGGACTTGTTGCAGAGA 2073

2.52 GAGCCCACTTCTGGCATGGACCCGGCCTCACGTCGTGCCCTGTGGGACTTGTTGCAGAGA 2073

5.8 GAGCCCACTTCTGGCATGGACCCGGCCTCACGTCGTGCCCTGTGGGACTTGTTGCAGAGA 2219

5.9 GAGCCCACTTCTGGCATGGACCCGGCCTCACGTCGTGCCCTGTGGGACTTGTTGCAGAGA 2201

5.11 GAGCCCACTTCTGGCATGGACCCGGCCTCACGTCGTGCCCTGTGGGACTTGTTGCAGAGA 2075

***************************************** ******************

MG637361.1 GAGAAGAAAGGTCGATCGATGATCCTGACGACACACTTCATGGACGAAGCGGACATATTA 2280

1.5 GAGAAGAAAGGTCGATCGATGATCCTGACGACACACTTCATGGACGAAGCGGACATATTA 2222

1.6 GAGAAGAAAGGTCGATCGATGATCCTGACGACACACTTCATGGACGAAGCGGACATATTA 2153

1.40 GAGAAGAAAGGTCGATCGATGATCCTGACGACACACTTCATGGACGAAGCGGACATATTA 2279

2.12 GAGAAGAAAGGTCGATCGATGATCCTGACGACACACTTCATGGACGAAGCGGACATATTA 2280

2.42 GAGAAGAAAGGTCGATCGATGATCCTGACGACACACTTCATGGACGAAGCGGACATATTA 2133

2.52 GAGAAGAAAGGTCGATCGATGATCCTGACGACACACTTCATGGACGAAGCGGACATATTA 2133

5.8 GAGAAGAAAGGTCGATCGATGATCCTGACGACACACTTCATGGACGAAGCGGACATATTA 2279

5.9 GAGAAGAAAGGTCGATCGATGATCCTGACGACACACTTCATGGACGAAGCGGACATATTA 2261

5.11 GAGAAGAAAGGTCGATCGATGATCCTGACGACACACTTCATGGACGAAGCGGACATATTA 2135

************************************************************

MG637361.1 GGGGATAGAGTTGCCATTATGGCGGACGGTCGTCTCCAGTGCGTGGGCTCACCTTACTTC 2340

1.5 GGGGATAGAGTTGCCATTATGGCGGACGGTCGTCTCCAGTGCGTGGGCTCACCTTACTTC 2282

1.6 GGGGATAGAGTTGCCATTATGGCGGACGGTCGTCTCCAGTGCGTGGGCTCACCTTACTTC 2213

1.40 GGGGATAGAGTTGCCATTATGGCGGACGGTCGTCTCCAGTGCGTGGGCTCACCTTACTTC 2339

2.12 GGGGATAGAGTTGCCATTATGGCGGACGGTCGTCTCCAGTGCGTGGGCTCACCTTACTTC 2340

2.42 GGGGATAGAGTTGCCATTATGGCGGACGGTCGTCTCCAGTGCGTGGGCTCACCTTACTTC 2193

2.52 GGGGATAGAGTTGCCATTATGGCGGACGGTCGTCTCCAGTGCGTGGGCTCACCTTACTTC 2193

5.8 GGGGATAGAGTTGCCATTATGGCGGACGGTCGTCTCCAGTGCGTGGGCTCACCTTACTTC 2339

5.9 GGGGATAGAGTTGCCATTATGGCGGACGGTCGTCTCCAGTGCGTGGGCTCACCTTACTTC 2321

5.11 GGGGATAGAGTTGCCATTATGGCGGACGGTCGTCTCCAGTGCGTGGGCTCACCTTACTTC 2195

************************************************************

MG637361.1 CTCAAGAGACACTATGGAGTCGGCTACACGCTAGTTGTGGTCAAGAAGGAAGATTTCCGA 2400

1.5 CTCAAGAGACACTATGGAGTCGGCTACACGCTAGTTGTGGTCAAGAAGGAAGATTTCCGA 2342

1.6 CTCAAGAGACACTATGGAGTCGGCTACACGCTAGTTGTGGTCAAGAAGGAAGATTTCCGA 2273

1.40 CTCAAGAGACACTATGGAGTCGGCTACACGCTAGTTGTGGTCAAGAAGGAAGATTTCCGA 2399

2.12 CTCAAGAGACACTATGGAGTCGGCTACACGCTAGTTGTGGTCAAGAAGGAAGATTTCCGA 2400

2.42 CTCAAGAGACACTATGGAGTCGGCTACACGCTAGTTGTGGTCAAGAAGGAAGATTTCCGA 2253

2.52 CTCAAGAGACACTATGGAGTCGGCTACACGCTAGTTGTGGTCAAGAAGGAAGATTTCCGA 2253

5.8 CTCAAGAGACACTATGGAGTCGGCTACACGCTAGTTGTGGTCAAGAAGGAAGATTTCCGA 2399

5.9 CTCAAGAGACACTATGGAGTCGGCTACACGCTAGTTGTGGTCAAGAAGGAAGATTTCCGA 2381

5.11 CTCAAGAGACACTATGGAGTCGGCTACACGCTAGTTGTGGTCAAGAAGGAAGATTTCCGA 2255

************************************************************

MG637361.1 CTGGACACCTGCACAGAGCTGATCAATAGATACATCCCTGGAACTGTTGTGAAGGAAGAC 2460

1.5 CTGGACACCTGCACAGAGCTGATCAATAGATACATCCCTGGAACTGTTGTGAAGGAAGAC 2402

1.6 CTGGACACCTGCACAGAGCTGATCAATAGATACATCCCTGGAACTGTTGTGAAGGAAGAC 2333

1.40 CTGGACACCTGCACAGAGCTGATCAATAGATACATCCCTGGAACTGTTGTGAAGGAAGAC 2459

2.12 CTGGACACCTGCACAGAGCTGATCAATAGATACATCCCTGGAACTGTTGTGAAGGAAGAC 2460

2.42 CTGGACACCTGCACAGAGCTGATCAATAGATACATCCCTGGAACTGTTGTGAAGGAAGAC 2313

2.52 CTGGACACCTGCACAGAGCTGATCAATAGATACATCCCTGGAACTGTTGTGAAGGAAGAC 2313

5.8 CTGGACACCTGCACAGAGCTGATCAATAGATACATCCCTGGAACTGTTGTGAAGGAAGAC 2459

5.9 CTGGACACCTGCACAGAGCTGATCAATAGATACATCCCTGGAACTGTTGTGAAGGAAGAC 2441

5.11 CTGGACACCTGCACAGAGCTGATCAATAGATACATCCCTGGAACTGTTGTGAAGGAAGAC 2315

************************************************************

MG637361.1 CGAGGCACTGAAGTGACATATAGCATGACTAATGAGTATTCGCACGTGTTTGAATCTATG 2520

1.5 CGAGGCACTGAAGTGACATATAGCATGACTAATGAGTATTCGCACGTGTTTGAATCTATG 2462

1.6 CGAGGCACTGAAGTGACATATAGCATGACTAATGAGTATTCGCACGTGTTTGAATCTATG 2393

1.40 CGAGGCACTGAAGTGACATATAGCATGACTAATGAGTATTCGCACGTGTTTGAATCTATG 2519

2.12 CGAGGCACTGAAGTGACATATAGCATGACTAATGAGTATTCGCACGTGTTTGAATCTATG 2520

2.42 CGAGGCACTGAAGTGACATATAGCATGACTAATGAGTATTCGCACGTGTTTGAATCTATG 2373

2.52 CGAGGCACTGAAGTGACATATAGCATGACTAATGAGTATTCGCACGTGTTTGAATCTATG 2373

5.8 CGAGGCACTGAAGTGACATATAGCATGACTAATGAGTATTCGCACGTGTTTGAATCTATG 2519

5.9 CGAGGCACTGAAGTGACATATAGCATGACTAATGAGTATTCGCACGTGTTTGAATCTATG 2501

5.11 CGAGGCACTGAAGTGACATATAGCATGACTAATGAGTATTCGCACGTGTTTGAATCTATG 2375

************************************************************

MG637361.1 CTGCGCGATTTGGAGGCAAAGGCCGATGAGATAAACTTTAAAAACTACGGCCTACTGGCT 2580

1.5 CTGCGCGATTTGGAGGCAAAAGCCGATGAGATAAACTTTAAAAACTACGGCCTACTGGCT 2522

1.6 CTGCGCGATTTGGAGGCAAAAGCCGATGAGATAAACTTTAAAAACTACGGCCTACTGGCT 2453

1.40 CTGCGCGATTTGGAGGCAAAAGCCGATGAGATAAACTTTAAAAACTACGGCCTACTGGCT 2579

2.12 CTGCGCGATTTGGAGGCAAAAGCCGATGAGATAAACTTTAAAAACTACGGCCTACTGGCT 2580

2.42 CTGCGCGATTTGGAGGCAAAAGCCGATGAGATAAACTTTAAAAACTACGGCCTACTGGCT 2433

2.52 CTGCGCGATTTGGAGGCAAAAGCCGATGAGATAAACTTTAAAAACTACGGCCTACTGGCT 2433

5.8 CTGCGCGATTTGGAGGCAAAAGCCGATGAGATAAACTTTAAAAACTACGGCCTACTGGCT 2579

5.9 CTGCGCGATTTGGAGGCAAAAGCCGATGAGATAAACTTTAAAAACTACGGCCTACTGGCT 2561

5.11 CTGCGCGATTTGGAGGCAAAAGCCGATGAGATAAACTTTAAAAACTACGGCCTACTGGCT 2435

******************** ***************************************

MG637361.1 ACTACATTAGAAGATGTGTTCATGTCCGTGGGCACAGATGTGGTCGCAACTTCAGATGTG 2640

1.5 ACTACATTAGAAGATGTGTTCATGTCCGTGGGCACAGATGTGGTCGCAACTTCAGATGTG 2582

1.6 ACTACATTAGAAGATGTGTTCATGTCCGTGGGCACAGATGTGGTCGCAACTTCAGATGTG 2513

1.40 ACTACATTAGAAGATGTGTTCATGTCCGTGGGCACAGATGTGGTCGCAACTTCAGATGTG 2639

2.12 ACTACATTAGAAGATGTGTTCATGTCCGTGGGCACAGATGTGGTCGCAACTTCAGATGTG 2640

2.42 ACTACATTAGAAGATGTGTTCATGTCCGTGGGCACAGATGTGGTCGCAACTTCAGATGTG 2493

2.52 ACTACATTAGAAGATGTGTTCATGTCCGTGGGCACAGATGTGGTCGCAACTTCAGATGTG 2493

5.8 ACTACATTAGAAGATGTGTTCATGTCCGTGGGCACAGATGTGGTCGCAACTTCAGATGTG 2639

5.9 ACTACATTAGAAGATGTGTTCATGTCCGTGGGCACAGATGTGGTCGCAACTTCAGATGTG 2621

5.11 ACTACATTAGAAGATGTGTTCATGTCCGTGGGCACAGATGTGGTCGCAACTTCAGATGTG 2495

************************************************************

MG637361.1 GACGACAATACAACCGTTTCATCTAGTGCTGATACTCTAGCATTTGAATATGATTCTTTA 2700

1.5 GACGACAATACAACCGTTTCATCTAGTGCTGATACTCTAGCATTTGAATATGATTCTTTA 2642

1.6 GACGACAATACAACCGTTTCATCTAGTGCTGATACTCTAGCATTTGAATATGATTCTTTA 2573

1.40 GACGACAATACAACCGTTTCATCTAGTGCTGATACTCTAGCATTTGAATATGATTCTTTA 2699

2.12 GACGACAATACAACCGTTTCATCTAGTGCTGATACTCTAGCATTTGAATATGATTCTTTA 2700

2.42 GACGACAATACAACCGTTTCATCTAGTGCTGATACTCTAGCATTTGAATATGATTCTTTA 2553

2.52 GACGACAATACAACCGTTTCATCTAGTGCTGATACTCTAGCATTTGAATATGATTCTTTA 2553

5.8 GACGACAATACAACCGTTTCATCTAGTGCTGATACTCTAGCATTTGAATATGATTCTTTA 2699

5.9 GACGACAATACAACCGTTTCATCTAGTGCTGATACTCTAGCATTTGAATATGATTCTTTA 2681

5.11 GACGACAATACAACCGTTTCATCTAGTGCTGATACTCTAGCATTTGAATATGATTCTTTA 2555

************************************************************

MG637361.1 GAAAAATTGGACGGGACTGGCTATGGGGATGAAAAAGGGATCCGATTAATTTGCCAACAC 2760

1.5 GAAAAATTGGACGGGACTGGCTATGGGGATGAAAAAGGGATCCGATTAATTTCCCAACAC 2702

1.6 GAAAAATTGGACGGGACTGGCTATGGGGATGAAAAAGGGATCCGATTAATTTCCCAACAC 2633

1.40 GAAAAATTGGACGGGACTGGCTATGGGGATGAAAAAGGGATCCGATTAATTTCCCAACAC 2759

2.12 GAAAAATTGGACGGGACTGGCTATGGGGATGAAAAAGGGATCCGATTAATTTCCCAACAC 2760

2.42 GAAAAATTGGACGGGACTGGCTATGGGGATGAAAAAGGGATCCGATTAATTTCCCAACAC 2613

2.52 GAAAAATTGGACGGGACTGGCTATGGGGATGAAAAAGGGATCCGATTAATTTCCCAACAC 2613

5.8 GAAAAATTGGACGGGACTGGCTATGGGGATGAAAAAGGGATCCGATTAATTTCCCAACAC 2759

5.9 GAAAAATTGGACGGGACTGGCTATGGGGATGAAAAAGGGATCCGATTAATTTCCCAACAC 2741

5.11 GAAAAATTGGACGGGACTGGCTATGGGGATGAAAAAGGGATCCGATTAATTTCCCAACAC 2615

**************************************************** *******

MG637361.1 GTGGTAGCAATATGGATGAAACTGTTTCTGGTGCTGACAAGGTCTTGGCTTATCCTGTTG 2820

1.5 GTGGTAGCAATATGGATGAAACTGTTTCTGGTGCTGACAAGGTCTTGGCTTATCCTGTTG 2762

1.6 GTGGTAGCAATATGGATGAAACTGTTTCTGGTGCTGACAAGGTCTTGGCTTATCCTGTTG 2693

1.40 GTGGTAGCAATATGGATGAAACTGTTTCTGGTGCTGACAAGGTCTTGGCTTATCCTGTTG 2819

2.12 GTGGTAGCAATATGGATGAAACTGTTTCTGGTGCTGACAAGGTCTTGGCTTATCCTGTTG 2820

2.42 GTGGTAGCAATATGGATGAAACTGTTTCTGGTGCTGACAAGGTCTTGGCTTATCCTGTTG 2673

2.52 GTGGTAGCAATATGGATGAAACTGTTTCTGGTGCTGACAAGGTCTTGGCTTATCCTGTTG 2673

5.8 GTGGTAGCAATATGGATGAAACTGTTTCTGGTGCTGACAAGGTCTTGGCTTATCCTGTTG 2819

5.9 GTGGTAGCAATATGGATGAAACTGTTTCTGGTGCTGACAAGGTCTTGGCTTATCCTGTTG 2801

5.11 GTGGTAGCAATATGGATGAAACTGTTTCTGGTGCTGACAAGGTCTTGGCTTATCCTGTTG 2675

************************************************************

MG637361.1 CTCCAAGTATTGGTGTCCTTGGTACAAATCATTGCCACACTCGGAGTCATGCAGTATGTC 2880

1.5 CTCCAAGTATTGGTGTCCTTGGTACAAATCATTGCCACACTCGGAGTCATGCAGTATGTC 2822

1.6 CTCCAAGTATTGGTGTCCTTGGTACAAATCATTGCCACACTCGGAGTCATGCAGTATGTC 2753

1.40 CTCCAAGTATTGGTGTCCTTGGTACAAATCATTGCCACACTCGGAGTCATGCAGTATGTC 2879

2.12 CTCCAAGTATTGGTGTCCTTGGTACAAATCATTGCCACACTCGGAGTCATGCAGTATGTC 2880

2.42 CTCCAAGTATTGGTGTCCTTGGTACAAATCATTGCCACACTCGGAGTCATGCAGTATGTC 2733

2.52 CTCCAAGTATTGGTGTCCTTGGTACAAATCATTGCCACACTCGGAGTCATGCAGTATGTC 2733

5.8 CTCCAAGTATTGGTGTCCTTGGTACAAATCATTGCCACACTCGGAGTCATGCAGTATGTC 2879

5.9 CTCCAAGTATTGGTGTCCTTGGTACAAATCATTGCCACACTCGGAGTCATGCAGTATGTC 2861

5.11 CTCCAAGTATTGGTGTCCTTGGTACAAATCATTGCCACACTCGGAGTCATGCAGTATGTC 2735

************************************************************

MG637361.1 ATCTCTATGACCGAGCATATACAAAGAAGAGAACTTTCATTGGCTGAAGGTTTCGCAGGC 2940

1.5 ATCTCTATGACCGAGCATATACAAAGAAGAGAACTTTCATTGGCTGAAGGTTTCGCAGGC 2882

1.6 ATCTCTATGACCGAGCATATACAAAGAAGAGAACTTTCATTGGCTGAAGGTTTCGCAGGC 2813

1.40 ATCTCTATGACCGAGCATATACAAAGAAGAGAACTTTCATTGGCTGAAGGTTTCGCAGGC 2939

2.12 ATCTCTATGACCGAGCATATACAAAGAAGAGAACTTTCATTGGCTGAAGGTTTCGCAGGC 2940

2.42 ATCTCTATGACCGAGCATATACAAAGAAGAGAACTTTCATTGGCTGAAGGTTTCGCAGGC 2793

2.52 ATCTCTATGACCGAGCATATACAAAGAAGAGAACTTTCATTGGCTGAAGGTTTCGCAGGC 2793

5.8 ATCTCTATGACCGAGCATATACAAAGAAGAGAACTTTCATTGGCTGAAGGTTTCGCAGGC 2939

5.9 ATCTCTATGACCGAGCATATACAAAGAAGAGAACTTTCATTGGCTGAAGGTTTCGCAGGC 2921

5.11 ATCTCTATGACCGAGCATATACAAAGAAGAGAACTTTCATTGGCTGAAGGTTTCGCAGGC 2795

************************************************************

MG637361.1 ACAGAAACATTAGTTAGTTTCAAAGGGTTGTCCCCTACATCGACAGGTTCGCTAGCGAAG 3000

1.5 ACAGAAACGCTAGTTAGTTTCAAAGGGTCGTCCCTTACATCGACAGGTTCGCTAGCGAAG 2942

1.6 ACAGAAACGCTAGTTAGTTTCAAAGGGTCGTCCCTTACATCGACAGGTTCGCTAGCGAAG 2873

1.40 ACAGAAACGCTAGTTAGTTTCAAAGGGTCGTCCCTTACATCGACAGGTTCGCTAGCGAAG 2999

2.12 ACAGAAACGCTAGTTAGTTTCAAAGGGTCGTCCCTTACATCGACAGGTTCGCTAGCGAAG 3000

2.42 ACAGAAACGCTAGTTAGTTTCAAAGGGTCGTCCCTTACATCGACAGGTTCGCTAGCGAAG 2853

2.52 ACAGAAACGCTAGTTAGTTTCAAAGGGTCGTCCCTTACATCGACAGGTTCGCTAGCGAAG 2853

5.8 ACAGAAACGCTAGTTAGTTTCAAAGGGTCGTCCCTTACATCGACAGGTTCGCTAGCGAAG 2999

5.9 ACAGAAACGCTAGTTAGTTTCAAAGGGTCGTCCCTTACATCGACAGGTTCGCTAGCGAAG 2981

5.11 ACAGAAACGCTAGTTAGTTTCAAAGGGTCGTCCCTTACATCGACAGGTTCGCTAGCGAAG 2855

******** ****************** ***** *************************

MG637361.1 GCTGCCTACGAGTCGATATTTGTAACCGCCAATAATCCCACAATGGAAATCACTGTTGTT 3060

1.5 GCTGCCTACGAGTCGATATTTGTAACCGCCAATAATCCCACAATGGAAATCACTGTTGTT 3002

1.6 GCTGCCTACGAGTCGATATTTGTAACCGCCAATAATCCCACAATGGAAATCACTGTTGTT 2933

1.40 GCTGCCTACGAGTCGATATTTGTAACCGCCAATAATCCCACAATGGAAATCACTGTTGTT 3059

2.12 GCTGCCTACGAGTCGATATTTGTAACCGCCAATAATCCCACAATGGAAATCACTGTTGTT 3060

2.42 GCTGCCTACGAGTCGATATTTGTAACCGCCAATAATCCCACAATGGAAATCACTGTTGTT 2913

2.52 GCTGCCTACGAGTCGATATTTGTAACCGCCAATAATCCCACAATGGAAATCACTGTTGTT 2913

5.8 GCTGCCTACGAGTCGATATTTGTAACCGCCAATAATCCCACAATGGAAATCACTGTTGTT 3059

5.9 GCTGCCTACGAGTCGATATTTGTAACCGCCAATAATCCCACAATGGAAATCACTGTTGTT 3041

5.11 GCTGCCTACGAGTCGATATTTGTAACCGCCAATAATCCCACAATGGAAATCACTGTTGTT 2915

************************************************************

MG637361.1 GATAATACACCTATAGATGAATATTATTTGGAAAGAACAGATGACGTATCAGCGATGGCG 3120

1.5 GATAATACACCTATAGATGAATATTATTTGGAAAGAA----TGACGTATCAGCGATGGCG 3058

1.6 GATAATACACCTATAGATGAATATTATTTGGAAAGAACAGATGACGTATCAGCGATGGCG 2993

1.40 GATAATACACCTATAGATGAATATTATTTGGAAAGAACAGATGACGTATCAGCGATGGCG 3119

2.12 GATAATACACCTATAGATGAATATTATTTGGAAAGAACAGATGACGTATCAGCGATGGCG 3120

2.42 GATAATACACCTATAGATGAATATTATTTGGAAAGAA----TGACGTATCAGCGATGGCG 2969

2.52 GATAATACACCTATAGATGAATATTATTTGGAAAGAA----TGACGTATCAGCGATGGCG 2969

5.8 GATAATACACCTATAGATGAATATTATTTGGAAAGAACAGATGACGTATCAGCGATGGCG 3119

5.9 GATAATACACCTATAGATGAATATTATTTGGAAAGAACAGATGACGTATCAGCGATGGCG 3101

5.11 GATAATACACCTATAGATGAATATTATTTGGAAAGAACAGATGACGTATCAGCGATGGCG 2975

************************************* ******************

MG637361.1 GTGCTCCGGCACAGTCTGTTGATCGGCGCGACGTTCGACGACCACTCCGCGACCGCGTGG 3180

1.5 GTGCTCCGGCACAGTCTGTTGATCGGCGCGACGTTCGACGACAACTCCGCGACCGCGTGG 3118

1.6 GTGCTCCGGCACAGTCTGTTGATCGGCGCGACGTTCGACGACAACTCCGCGACCGCGTGG 3053

1.40 GTGCTCCGGCACAGTCTGTTGATCGGCGCGACGTTCGACGACAACTCCGCGACCGCGTGG 3179

2.12 GTGCTCCGGCACAGTCTGTTGATCGGCGCGACGTTCGACGACAACTCCGCGACCGCGTGG 3180

2.42 GTGCTCCGGCACAGTCTGTTGATCGGCGCGACGTTCGACGACAACTCCGCGACCGCGTGG 3029

2.52 GTGCTCCGGCACAGTCTGTTGATCGGCGCGACGTTCGACGACAACTCCGCGACCGCGTGG 3029

5.8 GTGCTCCGGCACAGTCTGTTGATCGGCGCGACGTTCGACGACAACTCCGCGACCGCGTGG 3179

5.9 GTGCTCCGGCACAGTCTGTTGATCGGCGCGACGTTCGACGACAACTCCGCGACCGCGTGG 3161

5.11 GTGCTCCGGCACAGTCTGTTGATCGGCGCGACGTTCGACGACAACTCCGCGACCGCGTGG 3035

****************************************** *****************

MG637361.1 TTCAGCAACTTCGGTTACCACGACGTGGCCATGTCACTGGCTGCTGTGCACGCCGCCTTG 3240

1.5 TTCAGCAACTTCGGTTACCACGACGTGGCCATGTCACTGGCGGCTGTGCACGCCGCCTTG 3178

1.6 TTCAGCAACTTCGGTTACCACGACGTGGCCATGTCACTGGCGGCTGTGCACGCCGCCTTG 3113

1.40 TTCAGCAACTTCGGTTACCACGACGTGGCCATGTCACTGGCGGCTGTGCACGCCGCCTTG 3239

2.12 TTCAGCAACTTCGGTTACCACGACGTGGCCATGTCACTGGCGGCTGTGCACGCCGCCTTG 3240

2.42 TTCAGCAACTTCGGTTACCACGACGTGGCCATGTCACTGGCGGCTGTGCACGCCGCCTTG 3089

2.52 TTCAGCAACTTCGGTTACCACGACGTGGCCATGTCACTGGCGGCTGTGCACGCCGCCTTG 3089

5.8 TTCAGCAACTTCGGTTACCACGACGTGGCCATGTCACTGGCGGCTGTGCACGCCGCCTTG 3239

5.9 TTCAGCAACTTCGGTTACCACGACGTGGCCATGTCACTGGCGGCTGTGCACGCCGCCTTG 3221

5.11 TTCAGCAACTTCGGTTACCACGACGTGGCCATGTCACTGGCGGCTGTGCACGCCGCCTTG 3095

***************************************** ******************

MG637361.1 CTCAGAGCTGTCAATCCTGCAGCCAACTTGACTGTTTACAACCACCCACTTGAGGCCAAT 3300

1.5 CTCAGAGCTGTCAATCCTGCAGCCAACTTGACTGTTTACAACCACCCACTTGAGGCCAAT 3238

1.6 CTCAGAGCTGTCAATCCTGCAGCCAACTTGACTGTTTACAACCACCCACTTGAGGCCAAT 3173

1.40 CTCAGAGCTGTCAATCCTGCAGCCAACTTGACTGTTTACAACCACCCACTTGAGGCCAAT 3299

2.12 CTCAGAGCTGTCAATCCTGCAGCCAACTTGACTGTTTACAACCACCCACTTGAGGCCAAT 3300

2.42 CTCAGAGCTGTCAATCCTGCAGCCAACTTGACTGTTTACAACCACCCACTTGAGGCCAAT 3149

2.52 CTCAGAGCTGTCAATCCTGCAGCCAACTTGACTGTTTACAACCACCCACTTGAGGCCAAT 3149

5.8 CTCAGAGCTGTCAATCCTGCAGCCAACTTGACTGTTTACAACCACCCACTTGAGGCCAAT 3299

5.9 CTCAGAGCTGTCAATCCTGCAGCCAACTTGACTGTTTACAACCACCCACTTGAGGCCAAT 3281

5.11 CTCAGAGCTGTCAATCCTGCAGCCAACTTGACTGTTTACAACCACCCACTTGAGGCCAAT 3155

************************************************************

MG637361.1 TATGTCAACCAGAACGACATGCAAACAATGGTAGCGTTCCTCTCGATGCAACTTGCGTCG 3360

1.5 TATGTCAACCAGAACGACATGCAAACGATGGTAGCGTTCCTCTCGATGCAACTTGCGTCG 3298

1.6 TATGTCAACCAGAACGACATGCAAACGATGGTAGCGTTCCTCTCGATGCAACTTGCGTCG 3233

1.40 TATGTCAACCAGAACGACATGCAAACGATGGTAGCGTTCCTCTCGATGCAACTTGCGTCG 3359

2.12 TATGTCAACCAGAACGACATGCAAACGATGGTAGCGTTCCTCTCGATGCAACTTGCGTCG 3360

2.42 TATGTCAACCAGAACGACATGCAAACGATGGTAGCGTTCCTCTCGATGCAACTTGCGTCG 3209

2.52 TATGTCAACCAGAACGACATGCAAACGATGGTAGCGTTCCTCTCGATGCAACTTGCGTCG 3209

5.8 TATGTCAACCAGAACGACATGCAAACGATGGTAGCGTTCCTCTCGATGCAACTTGCGTCG 3359

5.9 TATGTCAACCAGAACGACATGCAAACGATGGTAGCGTTCCTCTCGATGCAACTTGCGTCG 3341

5.11 TATGTCAACCAGAACGACATGCAAACGATGGTAGCGTTCCTCTCGATGCAACTTGCGTCG 3215

************************** *********************************

MG637361.1 GGCATCGGCAGCAGTCTGTCAATTGTCAGTGCTGTGTTCATCATGTTCTATATCAAGGAG 3420

1.5 GGCATCGGCAGCAGTCTGTCAATTGTCAGTGCTGTGTTCATCATGTTCTATATCAAGGAG 3358

1.6 GGCATCGGCAGCAGTCTGTCAATTGTCAGTGCTGTGTTCATCATGTTCTATATCAAGGAG 3293

1.40 GGCATCGGCAGCAGTCTGTCAATTGTCAGTGCTGTGTTCATCATGTTCTATATCAAGGAG 3419

2.12 GGCATCGGCAGCAGTCTGTCAATTGTCAGTGCTGTGTTCATCATGTTCTATATCAAGGAG 3420

2.42 GGCATCGGCAGCAGTCTGTCAATTGTCAGTGCTGTGTTCATCATGTTCTATATCAAGGAG 3269

2.52 GGCATCGGCAGCAGTCTGTCAATTGTCAGTGCTGTGTTCATCATGTTCTATATCAAGGAG 3269

5.8 GGCATCGGCAGCAGTCTGTCAATTGTCAGTGCTGTGTTCATCATGTTCTATATCAAGGAG 3419

5.9 GGCATCGGCAGCAGTCTGTCAATTGTCAGTGCTGTGTTCATCATGTTCTATATCAAGGAG 3401

5.11 GGCATCGGCAGCAGTCTGTCAATTGTCAGTGCTGTGTTCATCATGTTCTATATCAAGGAG 3275

************************************************************

MG637361.1 CGAGTATCTCGCGCCAAGCTGCTGCAGAAGGCGGCAGGCATCCAGCCGTTAGTGATGTGG 3480

1.5 CGAGTATCTCGCGCCAAGCTGCTGCAGAAGGCGGCAGGCATCCAGCCGTTAGTGATGTGG 3418

1.6 CGAGTATCTCGCGCCAAGCTGCTGCAGAAGGCGGCAGGCATCCAGCCGTTAGTGATGTGG 3353

1.40 CGAGTATCTCGCGCCAAGCTGCTGCAGAAGGCGGCAGGCATCCAGCCGTTAGTGATGTGG 3479

2.12 CGAGTATCTCGCGCCAAGCTGCTGCAGAAGGCGGCAGGCATCCAGCCGTTAGTGATGTGG 3480

2.42 CGAGTGTCTCGCGCCAAGCTGCTGCAGAAGGCGGCAGGCATCCAGCCGTTAGTGATGTGG 3329

2.52 CGAGTGTCTCGCGCCAAGCTGCTGCAGAAGGCGGCAGGCATCCAGCCGTTAGTGATGTGG 3329

5.8 CGAGTATCTCGCGCCAAGCTGCTGCAGAAGGCGGCAGGCATCCAGCCGTTAGTGATGTGG 3479

5.9 CGAGTATCTCGCGCCAAGCTGCTGCAGAAGGCGGCAGGCATCCAGCCGTTAGTGATGTGG 3461

5.11 CGAGTATCTCGCGCCAAGCTGCTGCAGAAGGCGGCAGGCATCCAGCCGTTAGTGATGTGG 3335

***** ******************************************************

MG637361.1 CTCAGCGCCGCCGTGTTCGACTGGATCTGGTTCTGCGTCATCGCCGTCGGCATCGTTATC 3540

1.5 CTCAGCGCCGCCGTGTTCGACTGGATCTGGTTCTGCATCATCGCCGTCGGCATCGTTATC 3478

1.6 CTCAGCGCCGCCGTGTTCGACTGGATCTGGTTCTGCATCATCGCCGTCGGCATCGTTATC 3413

1.40 CTCAGCGCCGCCGTGTTCGACTGGATCTGGTTCTGCATCATCGCCGTCGGCATCGTTATC 3539

2.12 CTCAGCGCCGCCGTGTTCGACTGGATCTGGTTCTGCATCATCGCCGTCGGCATCGTTATC 3540

2.42 CTCAGCGCCGCCGTGTTCGACTGGATCTGGTTCTGCATCATCGCCGTCGGCATCGTTATC 3389

2.52 CTCAGCGCCGCCGTGTTCGACTGGATCTGGTTCTGCATCATCGCCGTCGGCATCGTTATC 3389

5.8 CTCAGCGCCGCCGTGTTCGACTGGATCTGGTTCTGCATCATCGCCGTCGGCATCGTTATC 3539

5.9 CTCAGCGCCGCCGTGTTCGACTGGATCTGGTTCTGCATCATCGCCGTCGGCATCGTTATC 3521

5.11 CTCAGCGCCGCCGTGTTCGACTGGATCTGGTTCTGCATCATCGCCGTCGGCATCGTTATC 3395

************************************ ***********************

MG637361.1 GCCTGCGCCGCTTTTAACGTCATTGGGCTCTCTTCTGTCGATGAACTGGGTCGGATGTAC 3600

1.5 GCCTGCGCCGCTTTTAACGTCATTGGGCTCTCTTCTGTCGATGAACTGGGTCGGATGTAC 3538

1.6 GCCTGCGCCGCTTTTAACGTCATTGGGCTCTCTTCTGTCGATGAACTGGGTCGGATGTAC 3473

1.40 GCCTGCGCCGCTTTTAACGTCATTGGGCTCTCTTCTGTCGATGAACTGGGTCGGATGTAC 3599

2.12 GCCTGCGCCGCTTTTAACGTCATTGGGCTCTCTTCTGTCGATGAACTGGGTCGGATGTAC 3600

2.42 GCCTGCGCCGCTTTTAACGTCATTGGGCTCTCTTCTGTCGATGAACTGGGTCGGATGTAC 3449

2.52 GCCTGCGCCGCTTTTAACGTCATTGGGCTCTCTTCTGTCGATGAACTGGGTCGGATGTAC 3449

5.8 GCCTGCGCCGCTTTTAACGTCATTGGGCTCTCTTCTGTCGATGAACTGGGTCGGATGTAC 3599

5.9 GCCTGCGCCGCTTTTAACGTCATTGGGCTCTCTTCTGTCGATGAACTGGGTCGGATGTAC 3581

5.11 GCCTGCGCCGCTTTTAACGTCATTGGGCTCTCTTCTGTCGATGAACTGGGTCGGATGTAC 3455

************************************************************

MG637361.1 TTGTGCATCATAGTGTATGGCGCCGCCAGTCTACCGATAGGCTACGTGTTCTCCTATTTC 3660

1.5 TTGTGCATCATAGTGTATGGCGCCGCCAGTCTGCCGATAGGCTACGTGTTCTCCTATTTC 3598

1.6 TTGTGCATCATAGTGTATGGCGCCGCCAGTCTGCCGATAGGCTACGTGTTCTCCTATTTC 3533

1.40 TTGTGCATCATAGTGTATGGCGCCGCCAGTCTGCCGATAGGCTACGTGTTCTCCTATTTC 3659

2.12 TTGTGCATCATAGTGTATGGCGCCGCCAGTCTGCCGATAGGCTACGTGTTCTCCTATTTC 3660

2.42 TTGTGCATCATAGTGTATGGCGCCGCCAGTCTGCCGATAGGCTACGTGTTCTCCTATTTC 3509

2.52 TTGTGCATCATAGTGTATGGCGCCGCCAGTCTGCCGATAGGCTACGTGTTCTCCTATTTC 3509

5.8 TTGTGCATCATAGTGTATGGCGCCGCCAGTCTGCCGATAGGCTACGTGTTCTCCTATTTC 3659

5.9 TTGTGCATCATAGTGTATGGCGCCGCCAGTCTGCCGATAGGCTACGTGTTCTCCTATTTC 3641

5.11 TTGTGCATCATAGTGTATGGCGCCGCCAGTCTGCCGATAGGCTACGTGTTCTCCTATTTC 3515

******************************** ***************************

MG637361.1 TTCAAAGGCCCTGCCGTCGGTTTTGTCACCATGTTCTTTATCAACATTCTCTTTGGTATG 3720

1.5 TTCAAAGGCCCTGCCGTCGGTTTTGTCACCATGTTCTTTATCAACATTCTCTTTGGTATG 3658

1.6 TTCAAAGGCCCTGCCGTCGGTTTTGTCACCATGTTCTTTATCAACATTCTCTTTGGTATG 3593

1.40 TTCAAAGGCCCTGCCGTCGGTTTTGTCACCATGTTCTTTATCAACATTCTCTTTGGTATG 3719

2.12 TTCAAAGGCCCTGCCGTCGGTTTTGTCACCATGTTCTTTATCAACATTCTCTTTGGTATG 3720

2.42 TTCAAAGGCCCTGCCGTCGGTTTTGTCACCATGTTCTTTATCAACATTCTCTTTGGTATG 3569

2.52 TTCAAAGGCCCTGCCGTCGGTTTTGTCACCATGTTCTTTATCAACATTCTCTTTGGTATG 3569

5.8 TTCAAAGGCCCTGCCGTCGGTTTTGTCACCATGTTCTTTATCAACATTCTCTTTGGTATG 3719

5.9 TTCAAAGGCCCTGCCGTCGGTTTTGTCACCATGTTCTTTATCAACATTCTCTTTGGTATG 3701

5.11 TTCAAAGGCCCTGCCGTCGGTTTTGTCACCATGTTCTTTATCAACATTCTCTTTGGTATG 3575

************************************************************

MG637361.1 ATGGGGGCGCAGATTGTGGAGGCCTTGTTGTCACCGCAGCTTGATACTGAAAATGTCGCT 3780

1.5 ATGGGGGCGCAGATTGTGGAGGCCTTGTTGTCACCGCAGCTTGATACTGAAAATGTCGCT 3718

1.6 ATGGGGGCGCAGATTGTGGAGGCCTTGTTGTCACCGCAGCTTGATACTGAAAATGTCGCT 3653

1.40 ATGGGGGCGCAGATTGTGGAGGCCTTGTTGTCACCGCAGCTTGATACTGAAAATGTCGCT 3779

2.12 ATGGGGGCGCAGATTGTGGAGGCCTTGTTGTCACCGCAGCTTGATACTGAAAATGTCGCT 3780

2.42 ATGGGGGCGCAGATTGTGGAGGCCTTGTTGTCACCGCAGCTTGATACTGAAAATGTCGCT 3629

2.52 ATGGGGGCGCAGATTGTGGAGGCCTTGTTGTCACCGCAGCTTGATACTGAAAATGTCGCT 3629

5.8 ATGGGGGCGCAGATTGTGGAGGCCTTGTTGTCACCGCAGCTTGATACTGAAAATGTCGCT 3779

5.9 ATGGGGGCGCAGATTGTGGAGGCCTTGTTGTCACCGCAGCTTGATACTGAAAATGTCGCT 3761

5.11 ATGGGGGCGCAGATTGTGGAGGCCTTGTTGTCACCGCAGCTTGATACTGAAAATGTCGCT 3635

************************************************************

MG637361.1 AATATACTTGACTCCATCTTGCAATTCTTCCCACTCTATAGTCTTGTCACATCTGCCAGA 3840

1.5 AATATACTTGACTCCATCTTGCAATTCTTCCCACTCTATGGTCTTGTCACATCTGCCAGA 3778

1.6 AATATACTTGACTCCATCTTGCAATTCTTCCCACTCTATGGTCTTGTCACATCTGCCAGA 3713

1.40 AATATACTTGACTCCATCTTGCAATTCTTCCCACTCTATGGTCTTGTCACATCTGCCAGA 3839

2.12 AATATACTTGACTCCATCTTGCAATTCTTCCCACTCTATGGTCTTGTCACATCTGCCAGA 3840

2.42 AATATACTTGACTCCATCTTGCAATTCTTCCCACTCTATGGTCTTGTCACATCTGCCAGA 3689

2.52 AATATACTTGACTCCATCTTGCAATTCTTCCCACTCTATGGTCTTGTCACATCTGCCAGA 3689

5.8 AATATACTTGACTCCATCTTGCAATTCTTCCCACTCTATGGTCTTGTCACATCTGCCAGA 3839

5.9 AATATACTTGACTCCATCTTGCAATTCTTCCCACTCTATGGTCTTGTCACATCTGCCAGA 3821

5.11 AATATACTTGACTCCATCTTGCAATTCTTCCCACTCTATGGTCTTGTCACATCTGCCAGA 3695

*************************************** ********************

MG637361.1 CTGTTGAATCAGGTGGGACTGCTGGAGTGGTCGTGCCTGCAGAACTGCGAGTACCTGTCC 3900

1.5 CTGTTGAATCAGGTGGGACTGCTGGAGTGGTCATGCCTGCAGAACTGCGAGTACCTGTCC 3838

1.6 CTGTTGAATCAGGTGGGACTGCTGGAGTGGTCATGCCTGCAGAACTGCGAGTACCTGTCC 3773

1.40 CTGTTGAATCAGGTGGGACTGCTGGAGTGGTCATGCCTGCAGAACTGCGAGTACCTGTCC 3899

2.12 CTGTTGAATCAGGTGGGACTGCTGGAGTGGTCATGCCTGCAGAACTGCGAGTACCTGTCC 3900

2.42 CTGTTGAATCAGGTGGGACTGCTGGAGTGGTCATGCCTGCAGAACTGCGAGTACCTGTCC 3749

2.52 CTGTTGAATCAGGTGGGACTGCTGGAGTGGTCATGCCTGCAGAACTGCGAGTACCTGTCC 3749

5.8 CTGTTGAATCAGGTGGGACTGCTGGAGTGGTCATGCCTGCAGAACTGCGAGTACCTGTCC 3899

5.9 CTGTTGAATCAGGTGGGACTGCTGGAGTGGTCATGCCTGCAGAACTGCGAGTACCTGTCC 3881

5.11 CTGTTGAATCAGGTGGGACTGCTGGAGTGGTCATGCCTGCAGAACTGCGAGTACCTGTCC 3755

******************************** ***************************

MG637361.1 GCAGTGATGCCCAACTTGACCGAATGCTCCATGGACGTTATGTGCCAGACGTTCTCACAA 3960

1.5 GCAGTGATGCCCAACTTGACCGAATGCTCCATGGACGTAATGTGCCAGACGTTCTCACAA 3898

1.6 GCAGTGATGCCCAACTTGACCGAATGCTCCATGGACGTAATGTGCCAGACGTTCTCACAA 3833

1.40 GCAGTGATGCCCAACTTGACCGAATGCTCCATGGACGTAATGTGCCAGACGTTCTCACAA 3959

2.12 GCAGTGATGCCCAACTTGACCGAATGCTCCATGGACGTAATGTGCCAGACGTTCTCACAA 3960

2.42 GCAGTGATGCCCAACTTGACCGAATGCTCCATGGACGTAATGTGCCAGACGTTCTCACAA 3809

2.52 GCAGTGATGCCCAACTTGACCGAATGCTCCATGGACGTAATGTGCCAGACGTTCTCACAA 3809

5.8 GCAGTGATGCCCAACTTGACCGAATGCTCCATGGACGTAATGTGCCAGACGTTCTCACAA 3959

5.9 GCAGTGATGCCCAACTTGACCGAATGCTCCATGGACGTAATGTGCCAGACGTTCTCACAA 3941

5.11 GCAGTGATGCCCAACTTGACCGAATGCTCCATGGACGTAATGTGCCAGACGTTCTCACAA 3815

************************************** *********************

MG637361.1 TGTTGCATCCCAGACGATCCTTGGTTCATGTGGGATCACCCTGGAGTACTCCGCTACATA 4020

1.5 TGTTGCATCCCTGACGATCCTTGGTTCATGTGGGATCACCCTGGAGTACTCCGCTACATA 3958

1.6 TGTTGCATCCCTGACGATCCTTGGTTCATGTGGGATCACCCTGGAGTACTCCGCTACATA 3893

1.40 TGTTGCATCCCTGACGATCCTTGGTTCATGTGGGATCACCCTGGAGTACTCCGCTACATA 4019

2.12 TGTTGCATCCCTGACGATCCTTGGTTCATGTGGGATCACCCTGGAGTACTCCGCTACATA 4020

2.42 TGTTGCATCCCTGACGATCCTTGGTTCATGTGGGATCACCCTGGAGTACTCCGCTACATA 3869

2.52 TGTTGCATCCCTGACGATCCTTGGTTCATGTGGGATCACCCTGGAGTACTCCGCTACATA 3869

5.8 TGTTGCATCCCTGACGATCCTTGGTTCATGTGGGATCACCCTGGAGTACTCCGCTACATA 4019

5.9 TGTTGCATCCCTGACGATCCTTGGTTCATGTGGGATCACCCTGGAGTACTCCGCTACATA 4001

5.11 TGTTGCATCCCTGACGATCCTTGGTTCATGTGGGATCACCCTGGAGTACTCCGCTACATA 3875

*********** ************************************************

MG637361.1 GTATGCATGATCGTCAGTGGAGTTGTCATGTGGTTCGTACTCTTGATCGCCGAGTATCGA 4080

1.5 GTATGCATGATCGTCAGTGGAGTTGTCATGTGGTTCGTACTCTTGATCGCCGAGTATCGA 4018

1.6 GTATGCATGATCGTCAGTGGAGTTGTCATGTGGTTCGTACTCTTGATCGCCGAGTATCGA 3953

1.40 GTATGCATGATCGTCAGTGGAGTTGTCATGTGGTTCGTACTCTTGATCGCCGAGTATCGA 4079

2.12 GTATGCATGATCGTCAGTGGAGTTGTCATGTGGTTCGTACTCTTGATCGCCGAGTATCGA 4080

2.42 GTATGCATGATCGTCAGTGGAGTTGTCATGTGGTTCGTACTCTTGATCGCCGAGTATCGA 3929

2.52 GTATGCATGATCGTCAGTGGAGTTGTCATGTGGTTCGTACTCTTGATCGCCGAGTATCGA 3929

5.8 GTATGCATGATCGTCAGTGGAGTTGTCATGTGGTTCGTACTCTTGATCGCCGAGTATCGA 4079

5.9 GTATGCATGATCGTCAGTGGAGTTGTCATGTGGTTCGTACTCTTGATCGCCGAGTATCGA 4061

5.11 GTATGCATGATCGTCAGTGGAGTTGTCATGTGGTTCGTACTCTTGATCGCCGAGTATCGA 3935

************************************************************

MG637361.1 TTGTTCCAGAAGGTGATCTACCGGGAAAAGAAAGCTCCTCCAGTTGATGAGAGCGCACTG 4140

1.5 TTGTTCCAGAAGGTGATCTACCGGGAAAAGAAAGCTCCTCCAGTTGATGAGAGCGCACTG 4078

1.6 TTGTTCCAGAAGGTGATCTACCGGGAAAAGAAAGCTCCTCCAGTTGATGAGAGCGCACTG 4013

1.40 TTGTTCCAGAAGGTGATCTACCGGGAAAAGAAAGCTCCTCCAGTTGATGAGAGCGCACTG 4139

2.12 TTGTTCCAGAAGGTGATCTACCGGGAAAAGAAAGCTCCTCCAGTTGATGAGAGCGCACTG 4140

2.42 TTGTTCCAGAAGGTGATCTACCGGGAAAAGAAAGCTCCTCCAGTTGATGAGAGCGCACTG 3989

2.52 TTGTTCCAGAAGGTGATCTACCGGGAAAAGAAAGCTCCTCCAGTTGATGAGAGCGCACTG 3989

5.8 TTGTTCCAGAAGGTGATCTACCGGGAAAAGAAAGCTCCTCCAGTTGATGAGAGCGCACTG 4139

5.9 TTGTTCCAGAAGGTGATCTACCGGGAAAAGAAAGCTCCTCCAGTTGATGAGAGCGCACTG 4121

5.11 TTGTTCCAGAAGGTGATCTACCGGGAAAAGAAAGCTCCTCCAGTTGATGAGAGCGCACTG 3995

************************************************************

MG637361.1 GACAATGACGTGGCGGACGAGGCCAGACACGTGGCGCGAGTTGGAGCAGGAGCAATCCTC 4200

1.5 GACAATGACGTGGCGGACGAGGCCAGACACGTGGCGCGAGTTGGAGCAGGAGCAATCCTC 4138

1.6 GACAATGACGTGGCGGACGAGGCCAGACACGTGGCGCGAGTTGGAGCAGGAGCAATCCTC 4073

1.40 GACAATGACGTGGCGGACGAGGCCAGACACGTGGCGCGAGTTGGAGCAGGAGCAATCCTC 4199

2.12 GACAATGACGTGGCGGACGAGGCCAGACACGTGGCGCGAGTTGGAGCAGGAGCAATCCTC 4200

2.42 GACAATGACGTGGCGGACGAGGCCAGACACGTGGCGCGAGTTGGAGCAGGAGCAATCCTC 4049

2.52 GACAATGACGTGGCGGACGAGGCCAGACACGTGGCGCGAGTTGGAGCAGGAGCAATCCTC 4049

5.8 GACAATGACGTGGCGGACGAGGCCAGACACGTGGCGCGAGTTGGAGCAGGAGCAATCCTC 4199

5.9 GACAATGACGTGGCGGACGAGGCCAGACACGTGGCGCGAGTTGGAGCAGGAGCAATCCTC 4181

5.11 GACAATGACGTGGCGGACGAGGCCAGACACGTGGCGCGAGTTGGAGCAGGAGCAATCCTC 4055

************************************************************

MG637361.1 GGGCAGCACAGCCTAGTAGCAAATGGCCTCACCAAGTATTATGGGAAACACCTTGCAGTC 4260

1.5 GGGCAGCACAGCCTAGTAGCAAATGGCCTCACCAAGTATTATGGGAAACACCTTGCAGTC 4198

1.6 GGGCAGCACAGCCTAGTAGCAAATGGCCTCACCAAGTATTATGGGAAACACCTTGCAGTC 4133

1.40 GGGCAGCACAGCCTAGTAGCAAATGGCCTCACCAAGTATTATGGGAAACACCTTGCAGTC 4259

2.12 GGGCAGCACAGCCTAGTAGCAAATGGCCTCACCAAGTATTATGGGAAACACCTTGCAGTC 4260

2.42 GGGCAGCACAGCCTAGTAGCAAATGGCCTCACCAAGTATTATGGGAAACACCTTGCAGTC 4109

2.52 GGGCAGCACAGCCTAGTAGCAAATGGCCTCACCAAGTATTATGGGAAACACCTTGCAGTC 4109

5.8 GGGCAGCACAGCCTAGTAGCAAATGGCCTCACCAAGTATTATGGGAAACACCTTGCAGTC 4259

5.9 GGGCAGCACAGCCTAGTAGCAAATGGCCTCACCAAGTATTATGGGAAACACCTTGCAGTC 4241

5.11 GGGCAGCACAGCCTAGTAGCAAATGGCCTCACCAAGTATTATGGGAAACACCTTGCAGTC 4115

************************************************************

MG637361.1 AATCAAGTGTCATTCACCGTGGGCGACACGGAATGCTTTGGTCTTCTGGGTGTGAACGGC 4320

1.5 AATCAAGTGTCATTCACCGTGGGCGACACGGAATGCTTTGGTCTTCTGGGTGTGAACGGC 4258

1.6 AATCAAGTGTCATTCACCGTGGGCGACACGGAATGCTTTGGTCTTCTGGGTGTGAACGGC 4193

1.40 AATCAAGTGTCATTCACCGTGGGCGACACGGAATGCTTTGGTCTTCTGGGTGTGAACGGC 4319

2.12 AATCAAGTGTCATTCACCGTGGGCGACACGGAATGCTTTGGTCTTCTGGGTGTGAACGGC 4320

2.42 AATCAAGTGTCATTCACCGTGGGCGACACGGAATGCTTTGGTCTTCTGGGTGTGAACGGC 4169

2.52 AATCAAGTGTCATTCACCGTGGGCGACACGGAATGCTTTGGTCTTCTGGGTGTGAACGGC 4169

5.8 AATCAAGTGTCATTCACCGTGGGCGACACGGAATGCTTTGGTCTTCTGGGTGTGAACGGC 4319

5.9 AATCAAGTGTCATTCACCGTGGGCGACACGGAATGCTTTGGTCTTCTGGGTGTGAACGGC 4301

5.11 AATCAAGTGTCATTCACCGTGGGCGACACGGAATGCTTTGGTCTTCTGGGTGTGAACGGC 4175

************************************************************

MG637361.1 GCCGGTAAGACGACCACCTTCAAGATGTTGATGGGAGATGAGACCGTCTCCAGCGGAGAT 4380

1.5 GCCGGTAAGACGACCACCTTCAAGATGTTGATGGGAGATGAGACCGTCTCCAGCGGAGAT 4318

1.6 GCCGGTAAGACGACCACCTTCAAGATGTTGATGGGAGATGAGACCGTCTCCAGCGGAGAT 4253

1.40 GCCGGTAAGACGACCACCTTCAAGATGTTGATGGGAGATGAGACCGTCTCCAGCGGAGAT 4379

2.12 GCCGGTAAGACGACCACCTTCAAGATGTTGATGGGAGATGAGACCGTCTCCAGCGGAGAT 4380

2.42 GCCGGTAAGACGACCACCTTCAAGATGTTGATGGGAGATGAGACCGTCTCCAGCGGAGAT 4229

2.52 GCCGGTAAGACGACCACCTTCAAGATGTTGATGGGAGATGAGACCGTCTCCAGCGGAGAT 4229

5.8 GCCGGTAAGACGACCACCTTCAAGATGTTGATGGGAGATGAGACCGTCTCCAGCGGAGAT 4379

5.9 GCCGGTAAGACGACCACCTTCAAGATGTTGATGGGAGATGAGACCGTCTCCAGCGGAGAT 4361

5.11 GCCGGTAAGACGACCACCTTCAAGATGTTGATGGGAGATGAGACCGTCTCCAGCGGAGAT 4235

************************************************************

MG637361.1 GCCTTCGTGAGTGGCCATTCTGTCAAGACTAATATCACTCAAGTTTACAAAAATATTGGT 4440

1.5 GCCTTCGTGAGTGGCCATTCTGTCAAGACTAATATCACTCAAGTTTACAAAAATATTGGT 4378

1.6 GCCTTCGTGAGTGGCCATTCTGTCAAGACTAATATCACTCAAGTTTACAAAAATATTGGT 4313

1.40 GCCTTCGTGAGTGGCCATTCTGTCAAGACTAATATCACTCAAGTTTACAAAAATATTGGT 4439

2.12 GCCTTCGTGAGTGGCCATTCTGTCAAGACTAATATCACTCAAGTTTACAAAAATATTGGT 4440

2.42 GCCTTCGTGAGTGGCCATTCTGTCAAGACTAATATCACTCAAGTTTACAAAAATATTGGT 4289

2.52 GCCTTCGTGAGTGGCCATTCTGTCAAGACTAATATCACTCAAGTTTACAAAAATATTGGT 4289

5.8 GCCTTCGTGAGTGGCCATTCTGTCAAGACTAATATCACTCAAGTTTACAAAAATATTGGT 4439

5.9 GCCTTCGTGAGTGGCCATTCTGTCAAGACTAATATCACTCAAGTTTACAAAAATATTGGT 4421

5.11 GCCTTCGTGAGTGGCCATTCTGTCAAGACTAATATCACTCAAGTTTACAAAAATATTGGT 4295

************************************************************

MG637361.1 TACTGTCCGCAATTCGAAGCGACATTCGGCGAGCTGACGGGACGCGAGACACTACGGCTG 4500

1.5 TACTGTCCGCAATTCGAAGCGACATTCGGCGAGCTGACGGGACGCGAGACACTACGGCTG 4438

1.6 TACTGTCCGCAATTCGAAGCGACATTCGGCGAGCTGACGGGACGCGAGACACTACGGCTG 4373

1.40 TACTGTCCGCAATTCGAAGCGACATTCGGCGAGCTGACGGGACGCGAGACACTACGGCTG 4499

2.12 TACTGTCCGCAATTCGAAGCGACATTCGGCGAGCTGACGGGACGCGAGACACTACGGCTG 4500

2.42 TACTGTCCGCAATTCGAAGCGACATTCGGCGAGCTGACGGGACGCGAGACACTACGGCTG 4349

2.52 TACTGTCCGCAATTCGAAGCGACATTCGGCGAGCTGACGGGACGCGAGACACTACGGCTG 4349

5.8 TACTGTCCGCAATTCGAAGCGACATTCGGCGAGCTGACGGGACGCGAGACACTACGGCTG 4499

5.9 TACTGTCCGCAATTCGAAGCGACATTCGGCGAGCTGACGGGACGCGAGACACTACGGCTG 4481

5.11 TACTGTCCGCAATTCGAAGCGACATTCGGCGAGCTGACGGGACGCGAGACACTACGGCTG 4355

************************************************************

MG637361.1 TTCTCGGCGCTGCGAGGGTTGCCAGTGCGAGGCGCCACGCTCCACGCGGAGGCCTTAGCA 4560

1.5 TTCTCGGCGCTGCGAGGGTTGCCAGTGCGAGGCGCCACGCTCCACGCGGAGGCCTTAGCA 4498

1.6 TTCTCGGCGCTGCGAGGGTTGCCAGTGCGAGGCGCCACGCTCCACGCGGAGGCCTTAGCA 4433

1.40 TTCTCGGCGCTGCGAGGGTTGCCAGTGCGAGGCGCCACGCTCCACGCGGAGGCCTTAGCA 4559

2.12 TTCTCGGCGCTGCGAGGGTTGCCAGTGCGAGGCGCCACGCTCCACGCGGAGGCCTTAGCA 4560

2.42 TTCTCGGCGCTGCGAGGGTTGCCAGTGCGAGGCGCCACGCTCCACGCGGAGGCCTTAGCA 4409

2.52 TTCTCGGCGCTGCGAGGGTTGCCAGTGCGAGGCGCCACGCTCCACGCGGAGGCCTTAGCA 4409

5.8 TTCTCGGCGCTGCGAGGGTTGCCAGTGCGAGGCGCCACGCTCCACGCGGAGGCCTTAGCA 4559

5.9 TTCTCGGCGCTGCGAGGGTTGCCAGTGCGAGGCGCCACGCTCCACGCGGAGGCCTTAGCA 4541

5.11 TTCTCGGCGCTGCGAGGGTTGCCAGTGCGAGGCGCCACGCTCCACGCGGAGGCCTTAGCA 4415

************************************************************

MG637361.1 CATGCTCTTGGTTTCTATAAGCATCTTGATAAAAGGGTGGACCACTACTCTGGTGGCAAC 4620

1.5 CATGCTCTTGGTTTCTATAAGCATCTTGATAAAAGGGTGGACCACTACTCTGGTGGCAAC 4558

1.6 CATGCTCTTGGTTTCTATAAGCATCTTGATAAAAGGGTGGACCACTACTCTGGTGGCAAC 4493

1.40 CATGCTCTTGGTTTCTATAAGCATCTTGATAAAAGGGTGGACCACTACTCTGGTGGCAAC 4619

2.12 CATGCTCTTGGTTTCTATAAGCATCTTGATAAAAGGGTGGACCACTACTCTGGTGGCAAC 4620

2.42 CATGCTCTTGGTTTCTATAAGCATCTTGATAAAAGGGTGGACCACTACTCTGGTGGCAAC 4469

2.52 CATGCTCTTGGTTTCTATAAGCATCTTGATAAAAGGGTGGACCACTACTCTGGTGGCAAC 4469

5.8 CATGCTCTTGGTTTCTATAAGCATCTTGATAAAAGGGTGGACCACTACTCTGGTGGCAAC 4619

5.9 CATGCTCTTGGTTTCTATAAGCATCTTGATAAAAGGGTGGACCACTACTCTGGTGGCAAC 4601

5.11 CATGCTCTTGGTTTCTATAAGCATCTTGATAAAAGGGTGGACCACTACTCTGGTGGCAAC 4475

************************************************************

MG637361.1 AAGCGCAAGTTGAGCACGGCTGTGGCGTTGCTGGGGCGCACGCGGCTTATATTCGTCGAC 4680

1.5 AAGCGCAAGTTGAGCACGGCTGTGGCGTTGCTTGGGCGCACGCGGCTTATATTCGTCGAC 4618

1.6 AAGCGCAAGTTGAGCACGGCTGTGGCGTTGCTTGGGCGCACGCGGCTTATATTCGTCGAC 4553

1.40 AAGCGCAAGTTGAGCACGGCTGTGGCGTTGCTTGGGCGCACGCGGCTTATATTCGTCGAC 4679

2.12 AAGCGCAAGTTGAGCACGGCTGTGGCGTTGCTTGGGCGCACGCGGCTTATATTCGTCGAC 4680

2.42 AAGCGCAAGTTGAGCACGGCTGTGGCGTTGCTTGGGCGCACGCGGCTTATATTCGTCGAC 4529

2.52 AAGCGCAAGTTGAGCACGGCTGTGGCGTTGCTTGGGCGCACGCGGCTTATATTCGTCGAC 4529

5.8 AAGCGCAAGTTGAGCACGGCTGTGGCGTTGCTTGGGCGCACGCGGCTTATATTCGTCGAC 4679

5.9 AAGCGCAAGTTGAGCACGGCTGTGGCGTTGCTTGGGCGCACGCGGCTTATATTCGTCGAC 4661

5.11 AAGCGCAAGTTGAGCACGGCTGTGGCGTTGCTTGGGCGCACGCGGCTTATATTCGTCGAC 4535

******************************** ***************************

MG637361.1 GAACCCACTACTGGAGTCGATCCTGCTGCTAAGAGACAGATGTGGAACGCGGTTCGAGAA 4740

1.5 GAACCCACTACTGGAGTCGATCCCGCTGCTAAGAGACAGATGTGGAACGCGGTTCGAGAA 4678

1.6 GAACCCACTACTGGAGTCGATCCCGCTGCTAAGAGACAGATGTGGAACGCGGTTCGAGAA 4613

1.40 GAACCCACTACTGGAGTCGATCCCGCTGCTAAGAGACAGATGTGGAACGCGGTTCGAGAA 4739

2.12 GAACCCACTACTGGAGTCGATCCCGCTGCTAAGAGACAGATGTGGAACGCGGTTCGAGAA 4740

2.42 GAACCCACTACTGGAGTCGATCCCGCTGCTAAGAGACAGATGTGGAACGCGGTTCGAGAA 4589

2.52 GAACCCACTACTGGAGTCGATCCCGCTGCTAAGAGACAGATGTGGAACGCGGTTCGAGAA 4589

5.8 GAACCCACTACTGGAGTCGATCCCGCTGCTAAGAGACAGATGTGGAACGCGGTTCGAGAA 4739

5.9 GAACCCACTACTGGAGTCGATCCCGCTGCTAAGAGACAGATGTGGAACGCGGTTCGAGAA 4721

5.11 GAACCCACTACTGGAGTCGATCCCGCTGCTAAGAGACAGATGTGGAACGCGGTTCGAGAA 4595

*********************** ************************************

MG637361.1 GCTCGCCGGTCGGGTCGTGGTGTGGTGCTGACATCACACAGCATGGAGGAGTGTGAGGCT 4800

1.5 GCTCGCCGGTCGGGTCGTGGTGTGGTGCTGACATCACACAGCATGGAGGAGTGTGAGGCT 4738

1.6 GCTCGCCGGTCGGGTCGTGGTGTGGTGCTGACATCACACAGCATGGAGGAGTGTGAGGCT 4673

1.40 GCTCGCCGGTCGGGTCGTGGTGTGGTGCTGACATCACACAGCATGGAGGAGTGTGAGGCT 4799

2.12 GCTCGCCGGTCGGGTCGTGGTGTGGTGCTGACATCACACAGCATGGAGGAGTGTGAGGCT 4800

2.42 GCTCGCCGGTCGGGTCGTGGTGTGGTGCTGACATCACACAGCATGGAGGAGTGTGAGGCT 4649

2.52 GCTCGCCGGTCGGGTCGTGGTGTGGTGCTGACATCACACAGCATGGAGGAGTGTGAGGCT 4649

5.8 GCTCGCCGGTCGGGTCGTGGTGTGGTGCTGACATCACACAGCATGGAGGAGTGTGAGGCT 4799

5.9 GCTCGCCGGTCGGGTCGTGGTGTGGTGCTGACATCACACAGCATGGAGGAGTGTGAGGCT 4781

5.11 GCTCGCCGGTCGGGTCGTGGTGTGGTGCTGACATCACACAGCATGGAGGAGTGTGAGGCT 4655

************************************************************

MG637361.1 CTGTGCTCGCGGCTCACAATCATGGTCAACGGACAGTTCCAGTGCCTCGGCACGCCGCAA 4860

1.5 CTGTGCTCGCGGCTCACAATCATGGTCAACGGACAGTTCCAGTGCCTCGGCACGCCGCAA 4798

1.6 CTGTGCTCGCGGCTCACAATCATGGTCAACGGACAGTTCCAGTGCCTCGGCACGCCGCAA 4733

1.40 CTGTGCTCGCGGCTCACAATCATGGTCAACGGACAGTTCCAGTGCCTCGGCACGCCGCAA 4859

2.12 CTGTGCTCGCGGCTCACAATCATGGTCAACGGACAGTTCCAGTGCCTCGGCACGCCGCAA 4860

2.42 CTGTGCTCGCGGCTCACAATCATGGTCAACGGACAGTTCCAGTGCCTCGGCACGCCGCAA 4709

2.52 CTGTGCTCGCGGCTCACAATCATGGTCAACGGACAGTTCCAGTGCCTCGGCACGCCGCAA 4709

5.8 CTGTGCTCGCGGCTCACAATCATGGTCAACGGACAGTTCCAGTGCCTCGGCACGCCGCAA 4859

5.9 CTGTGCTCGCGGCTCACAATCATGGTCAACGGACAGTTCCAGTGCCTCGGCACGCCGCAA 4841

5.11 CTGTGCTCGCGGCTCACAATCATGGTCAACGGACAGTTCCAGTGCCTCGGCACGCCGCAA 4715

************************************************************

MG637361.1 CATTTAAAGAATAAGTTCTCTGAAGGTTTCACATTGACAATTAAAATTAAAGTGGACGAC 4920

1.5 CATTTAAAGAATAAGTTCTCTGAAGGTTTCACATTGACAATTAAAATTAAAGTGGACGAC 4858

1.6 CATTTAAAGAATAAGTTCTCTGAAGGTTTCACATTGACAATTAAAATTAAAGTGGACGAC 4793

1.40 CATTTAAAGAATAAGTTCTCTGAAGGTTTCACATTGACAATTAAAATTAAAGTGGACGAC 4919

2.12 CATTTAAAGAATAAGTTCTCTGAAGGTTTCACATTGACAATTAAAATTAAAGTGGACGAC 4920

2.42 CATTTAAAGAATAAGTTCTCTGAAGGTTTCACATTGACAATTAAAATTAAAGTGGACGAC 4769

2.52 CATTTAAAGAATAAGTTCTCTGAAGGTTTCACATTGACAATTAAAATTAAAGTGGACGAC 4769

5.8 CATTTAAAGAATAAGTTCTCTGAAGGTTTCACATTGACAATTAAAATTAAAGTGGACGAC 4919

5.9 CATTTAAAGAATAAGTTCTCTGAAGGTTTCACATTGACAATTAAAATTAAAGTGGACGAC 4901

5.11 CATTTAAAGAATAAGTTCTCTGAAGGTTTCACATTGACAATTAAAATTAAAGTGGACGAC 4775

************************************************************

MG637361.1 GAGACGAAGACTGTACGGCCTGAAGTCTGCGATGCTGTGAAGCATTACGTCAGTACCAAC 4980

1.5 GAGACGAAGACTGTACGGCCTGAAGTCTGCGATGCTGTGAAGCATTACGTCAGTACCAAC 4918

1.6 GAGACGAAGACTGTACGGCCTGAAGTCTGCGATGCTGTGAAGCATTACGTCAGTACCAAC 4853

1.40 GAGACGAAGACTGTACGGCCTGAAGTCTGCGATGCTGTGAAGCATTACGTCAGTACCAAC 4979

2.12 GAGACGAAGACTGTACGGCCTGAAGTCTGCGATGCTGTGAAGCATTACGTCAGTACCAAC 4980

2.42 GAGACGAAGACTGTACGGCCTGAAGTCTGCGATGCTGTGAAGCATTACGTCAGTACCAAC 4829

2.52 GAGACGAAGACTGTACGGCCTGAAGTCTGCGATGCTGTGAAGCATTACGTCAGTACCAAC 4829

5.8 GAGACGAAGACTGTACGGCCTGAAGTCTGCGATGCTGTGAAGCATTACGTCAGTACCAAC 4979

5.9 GAGACGAAGACTGTACGGCCTGAAGTCTGCGATGCTGTGAAGCATTACGTCAGTACCAAC 4961

5.11 GAGACGAAGACTGTACGGCCTGAAGTCTGCGATGCTGTGAAGCATTACGTCAGTACCAAC 4835

************************************************************

MG637361.1 TTCAGAGAGCCGAAGATTATGGAGGAGTACCAGGGTCTGTTAACATACTATTTGCCAGAC 5040

1.5 TTCAGAGAGCCGAAGATTATGGAGGAGTACCAGGGTCTGTTAACATACTATTTGCCAGAC 4978

1.6 TTCAGAGAGCCGAAGATTATGGAGGAGTACCAGGGTCTGTTAACATACTATTTGCCAGAC 4913

1.40 TTCAGAGAGCCGAAGATTATGGAGGAGTACCAGGGTCTGTTAACATACTATTTGCCAGAC 5039

2.12 TTCAGAGAGCCGAAGATTATGGAGGAGTACCAGGGTCTGTTAACATACTATTTGCCAGAC 5040

2.42 TTCAGAGAGCCGAAGATTATGGAGGAGTACCAGGGTCTGTTAACATACTATTTGCCAGAC 4889

2.52 TTCAGAGAGCCGAAGATTATGGAGGAGTACCAGGGTCTGTTAACATACTATTTGCCAGAC 4889

5.8 TTCAGAGAGCCGAAGATTATGGAGGAGTACCAGGGTCTGTTAACATACTATTTGCCAGAC 5039

5.9 TTCAGAGAGCCGAAGATTATGGAGGAGTACCAGGGTCTGTTAACATACTATTTGCCAGAC 5021

5.11 TTCAGAGAGCCGAAGATTATGGAGGAGTACCAGGGTCTGTTAACATACTATTTGCCAGAC 4895

************************************************************

MG637361.1 AAGTCGGTGGCGTGGTCCAGAATGTTCGGCATAATGGAGGCGGCCAAACGCGACCTCCCC 5100

1.5 AAGTCGGTGGCGTGGTCCAGAATGTTCGGCATAATGGAGGCGGCCAAACGCGACCTCCCC 5038

1.6 AAGTCGGTGGCGTGGTCCAGAATGTTCGGCATAATGGAGGCGGCCAAACGCGACCTCCCC 4973

1.40 AAGTCGGTGGCGTGGTCCAGAATGTTCGGCATAATGGAGGCGGCCAAACGCGACCTCCCC 5099

2.12 AAGTCGGTGGCGTGGTCCAGAATGTTCGGCATAATGGAGGCGGCCAAACGCGACCTCCCC 5100

2.42 AAGTCGGTGGCGTGGTCCAGAATGTTCGGCATAATGGAGGCGGCCAAACGCGACCTCCCC 4949

2.52 AAGTCGGTGGCGTGGTCCAGAATGTTCGGCATAATGGAGGCGGCCAAACGCGACCTCCCC 4949

5.8 AAGTCGGTGGCGTGGTCCAGAATGTTCGGCATAATGGAGGCGGCCAAACGCGACCTCCCC 5099

5.9 AAGTCGGTGGCGTGGTCCAGAATGTTCGGCATAATGGAGGCGGCCAAACGCGACCTCCCC 5081

5.11 AAGTCGGTGGCGTGGTCCAGAATGTTCGGCATAATGGAGGCGGCCAAACGCGACCTCCCC 4955

************************************************************

MG637361.1 GTCGAAGACTACAGCATATCACAAACTACCCTCGAGCAGATATTCCTACAGTTCACAAAG 5160

1.5 GTCGAAGACTACAGCATATCACAAACTACCCTCGAG------------------------ 5074

1.6 GTCGAAGACTACAGCATATCACAAACTACCCTCGAG------------------------ 5009

1.40 GTCGAAGACTACAGCATATCACAAACTACCCTCGAG------------------------ 5135

2.12 GTCGAAGACTACAGCATATCACAAACTACCCTCGAG------------------------ 5136

2.42 GTCGAAGACTACAGCATATCACAAACTACCCTCGAG------------------------ 4985

2.52 GTCGAAGACTACAGCATATCACAAACTACCCTCGAG------------------------ 4985

5.8 GTCGAAGACTACAGCATATCACAAACTACCCTCGAG------------------------ 5135

5.9 GTCGAAGACTACAGCATATCACAAACTACCCTCGAA------------------------ 5117

5.11 GTCGAAGACTACAGCATATCACAAACTACCCTCGAG------------------------ 4991

***********************************

MG637361.1 TATCAACATGAAGCACAACAGACATAA 5187

1.5 --------------------------- 5074

1.6 --------------------------- 5009

1.40 --------------------------- 5135

2.12 --------------------------- 5136

2.42 --------------------------- 4985

2.52 --------------------------- 4985

5.8 --------------------------- 5135

5.9 --------------------------- 5117

5.11 --------------------------- 4991
