# Supplementary material for: Shared and Independent Genetic Basis of Resistance to Bt Toxin Cry2Ab in Two Strains of Pink Bollworm
Source: Sci Rep. 2020 May 14;10:7988. doi: 10.1038/s41598-020-64811-w (PMC7224296; doi:10.1038/s41598-020-64811-w)
Supplement: Supplementary file 3 — Supplementary figure S2. [file 41598_2020_64811_MOESM3_ESM.docx]

A. 68+141

MG637361.1 AACAATTTATCCCTCAACATTTACGACGACCAAATCACGGTTCTACTTGGACACAACGGA 1800

BX-R_1_68+141 -------------------TTTACGACGACCAAATCACGGTTCTACTTGGACACAACGGA

BX-R_2_68+141 -------------------TTTACGACGACCAAATCACGGTTCTACTTGGACACAACGGA

BX-R_5_68+141 -------------------TTTACGACGACCAAATCACGGTTCTACTTGGACACAACGGA

BX-R_2.42 AACAATTTATCCCTCAATATTTACGACGACCAAATCACGGTTCTACTTGGACACAACGGA

BX-R_2.52 AACAATTTATCCCTCAATATTTACGACGACCAAATCACGGTTCTACTTGGACACAACGGA

BX-R_1.5 AACAATTTATCCCTCAATATTTACGACGACCAAATCACGGTTCTA---------------

BX-R_5.9 AACAATTTATCCCTCAATATTTACGACGACCAAATCACGGTTCTACTTGGACACAACGGA

BX-R_5.11 AACAATTTATCCCTCAATATTTACGACGACCAAATCACGGTTCTACTTGGACACAACGGA

BX-R_5.8 AACAATTTATCCCTCAATATTTACGACGACCAAATCACGGTTCTACTTGGACACAACGGA

BX-R_2.12 AACAATTTATCCCTCAATATTTACGACGACCAAATCACGGTTCTACTTGGACACAACGGA

BX-R_1.6 AACAATTTATCCCTCAATATTTACGACGACCAAATCACGGTTCTACTTGGACACAACGGA

BX-R_1.40 AACAATTTATCCCTCAATATTTACGACGACCAAATCACGGTTCTACTTGGACACAACGGA

**************************

MG637361.1 GCGGGAAAATCCACAACCATTTCAATGCTCACAGGTAACGTGGACATAACCAGCGGGTCG 1860

BX-R_1_68+141 GCGGGAAAATCCACGACCATTTCAATGCTCACAGGTAACGTGGACATAACCAGCGGGTCG

BX-R_2_68+141 GCGGGAAAATCCACGACCATTTCAATGCTCACAGGTAACGTGGACATAACCAGCGGGTCG

BX-R_5_68+141 GCGGGAAAATCCACGACCATTTCAATGCTCACAGGTAACGTGGACATAACCAGCGGGTCG

BX-R_2.42 GCGGGAAAATCCACGACCATTTCAATGCTCACAGGTAACGTGGACATAACCAGCGGGTCG

BX-R_2.52 GCGGGAAAATCCACGACCATTTCAATGCTCACAGGTAACGTGGACATAACCAGCGGGTCG

BX-R_1.5 ---------------------------------------------ATAACCAGCGGGTCG

BX-R_5.9 GCGGGAAAATCCACGACCATTTCAATGCTCACA-------------------GCGGGTCG

BX-R_5.11 GCGGGAAAATCCACGACCATTTCAATGCTCACAGGTAACGTGGACATAACCAGCGGGTCG

BX-R_5.8 GCGGGAAAATCCACGACCATTTCAATGCTCACAGGTAACGTGGACATAACCAGCGGGTCG

BX-R_2.12 GCGGGAAAATCCACGACCATTTCAATGCTCACAGGTAACGTGGACATAACCAGCGGGTCG

BX-R_1.6 GCGGGAAAATCCACGACCATTTCAATGCTCACAGGTAACGTGGACATAACCAGCGGGTCG

BX-R_1.40 GCGGGAAAATCCACGACCATTTCAATGCTCACAGGTAACGTGGACATAACCAGCGGGTCG

********

MG637361.1 GTGACGGTGGCTGGCTACGACATAGAAAAACAAACAAGTTCAGCACGCTCACACATTGGA 1920

BX-R_1_68+141 GTGACGGTGGCTGGCTACGACATAGAAAAACAAACAAGTTCAGCACGCTCACACATTGGA

BX-R_2_68+141 GTGACGGTGGCTGGCTACGACATAGAAAAACAAACAAGTTCAGCACGCTCACACATTGGA

BX-R_5_68+141 GTGACGGTGGCTGGCTACGACATAGAAAAACAAACAAGTTCAGCACGCTCACACATTGGA

BX-R_2.42 GTGACGGTGGCTGGCTACGACATAGAAAAACAAACAAGTTCAGCACGCTCACACATTGGA

BX-R_2.52 GTGACGGTGGCTGGCTACGACATAGAAAAACAAACAAGTTCAGCACGCTCACACATTGGA

BX-R_1.5 GTGACGGTGGCTGGCTACGACATAGAAAAACAAACAAGTTCAGCACGCTCACACATTGGA

BX-R_5.9 GTGACGGTGGCTGGCTACGACATAGAAAAACAAACAAGTTCAGCACGCTCACACATTGGA

BX-R_5.11 GTGACGGTGGCTGGCTACGACATAGAAAAACAAACAAGTTCAGCACGCTCACACATTGGA

BX-R_5.8 GTGACGGTGGCTGGCTACGACATAGAAAAACAAACAAGTTCAGCACGCTCACACATTGGA

BX-R_2.12 GTGACGGTGGCTGGCTACGACATAGAAAAACAAACAAGTTCAGCACGCTCACACATTGGA

BX-R_1.6 GTGACGGTGGCTGGCTACGACATAGAAAAACAAACAAGTTCAGCAC--------------

BX-R_1.40 GTGACGGTGGCTGGCTACGACATAGAAAAACAAACAAGTTCAGCACGCTCACACATTGGA

**********************************************

MG637361.1 CTCTGCCCTCAACATAACGTACTCTTCAACGAACTCACAGTCAAAGAACATTTACAGTTC 1980

BX-R_1_68+141 CTCTGCCCTCAACATAACGTACTCTTCAACGAACTCACAGTCAAAGAACATTTACAGTTC

BX-R_2_68+141 CTCTGCCCTCAACATAACGTACTCTTCAACGAACTCACAGTCAAAGAACATTTACAGTTC

BX-R_5_68+141 CTCTGCCCTCAACATAACGTACTCTTCAACGAACTCACAGTCAAAGAACATTTACAGTTC

BX-R_2.42 CTCTGCCCTCAACATAACGTACTCTTCAACGAACTCACAGTCAAAGAACATTTACAGTTC

BX-R_2.52 CTCTGCCCTCAACATAACGTACTCTTCAACGAACTCACAGTCAAAGAACATTTACAGTTC

BX-R_1.5 CTCTGCCCTCAACATAACGTACTCTTCAACGAACTCACAGTCAAAGAACATTTACAGTTC

BX-R_5.9 CTCTGCCCTCAACATAACGTACTCTTCAACGAACTCACAGTCAAAGAACATTTACAGTTC

BX-R_5.11 CTCTGCCCTCAACATAACGTACTCTTCAACGAACTCACAGTCAAAGAACATTTACAGTTC

BX-R_5.8 CTCTGCCCTCAACATAACGTACTCTTCAACGAACTCACAGTCAAAGAACATTTACAGTTC

BX-R_2.12 CTCTGCCCTCAACATAACGTACTCTTCAACGAACTCACAGTCAAAGAACATTTACAGTTC

BX-R_1.6 ------------------------------------------------------------

BX-R_1.40 CTCTGCCCTCAACATAACGTACTCTTCAACGAACTCACAGTC-AAGAACATTTACAGTTC

MG637361.1 TTCTCTCGTCTGAAAGGCTTCAGCGGTAAAGAGTTGGATGAAGAAATTGAGACGCTTATT 2040

BX-R_1_68+141 TTCTCTCGTCTGAAAGGCTTCAGCGGTAAAGAGTTGGATGAAGAAATTGTGACGCTTATT

BX-R_2_68+141 TTCTCTCGTCTGAAAGGCTTCAGCGGTAAAGAGTTGGATGAAGAAATTGTGACGCTTATT

BX-R_5_68+141 TTCTCTCGTCTGAAAGGCTTCAGCGGTAAAGAGTTGGATGAAGAAATTGTGACGCTTATT

BX-R_2.42 TTCTCTCGTCTGAAAGGCTTCAGCGGTAAAGAGTTGGATGAAGAAATTGTGACGCTTATT

BX-R_2.52 TTCTCTCGTCTGAAAGGCTTCAGCGGTAAAGAGTTGGATGAAGAAATTGTGACGCTTATT

BX-R_1.5 TTCTCTCGTCTGAAAGGCTTCAGCGGTAAAGAGTTGGATGAAGAAATTGTGACGCTTATT

BX-R_5.9 TTCTCTCGTCTGAAAGGCTTCAGCGGTAAAGAGTTGGATGAAGAAATTGTGACGCTTATT

BX-R_5.11 TTCTCTCGTCTGAAAGGCTTCAGCGGTAAAGAGTTGGATGAAGAAATTGTGACGCTTATT

BX-R_5.8 TTCTCTCGTCTGAAAGGCTTCAGCGGTAAAGAGTTGGATGAAGAAATTGTGACGCTTATT

BX-R_2.12 TTCTCTCGTCTGAAAGGCTTCAGCGGTAAAGAGTTGGATGAAGAAATTGTGACGCTTATT

BX-R_1.6 ---------------------------------------------------ACGCTTATT

BX-R_1.40 TTCTCTCGTCTGAAAGGCTTCAGCGGTAAAGAGTTGGATGAAGAAATTGTGACGCTTATT

*********

MG637361.1 GAAAAATTGGAATTGCAAGAAAAGAGGGATTACCAATCAGCGGGGTTATCAGGGGGACAG 2100

BX-R_1_68+141 GAAAAATTGGAATTGCAAGAAAAGAGGGATTACCAATCAGCGGGGTTATCAG--------

BX-R_2_68+141 GAAAAATTGGAATTGCAAGAAAAGAGGGATTACCAATCAGCGGGGTTATCAG--------

BX-R_5_68+141 GAAAAATTGGAATTGCAAGAAAAGAGGGATTACCAATCAGCGGGGTTATCAG--------

BX-R_2.42 GAAAAATTGGAATTGCAAGAAAAGAGGGATTACCAATCAGCGGGATTATCAGGGGGACAG

BX-R_2.52 GAAAAATTGGAATTGCAAGAAAAGAGGGATTACCAATCAGCGGGATTATCAGGGGGACAG

BX-R_1.5 GAAAAATTGGAATTGCAAGAAAAGAGGGATTACCAATCAGCGGGATTATCAGGGGGACAG

BX-R_5.9 GAAAAATTGGAATTGCAAGAAAAGAGGGATTACCAATCAGCGGGATTATCAGGGGGACAG

BX-R_5.11 GAAAAATTGGAATTGCAAGAAAAGAGGGATTACCAATCAGCGGGATTATCAGGGGGACAG

BX-R_5.8 GAAAAATTGGAATTGCAAGAAAAGAGGGATTACCAATCAGCGGGATTATCAGGGGGACAG

BX-R_2.12 GAAAAATTGGAATTGCAAGAAAAGAGGGATTACCAATCAGCGGGATTATCAGGGGGACAG

BX-R_1.6 GAAAAATTGGAATTGCAAGAAAAGAGGGATTACCAATCAGCGGGATTATCAGGGGGACAG

BX-R_1.40 GAAAAATTGGAATTGCAAGAAAAGAGGGATTACCAATCAGCGGGATTATCAGGGGGACAG

******************************************** *******

B. 126+127

MG637361.1 CGAGGCACTGAAGTGACATATAGCATGACTAATGAGTATTCGCACGTGTTTGAATCTATG 2520

BX-R_2.42 CGAGGCACTGAAGTGACATATAGCATGACTAATGAGTATTCGCACGTGTTTGAATCTATG

BX-R_2.52 CGAGGCACTGAAGTGACATATAGCATGACTAATGAGTATTCGCACGTGTTTGAATCTATG

BX-R_5.11 CGAGGCACTGAAGTGACATATAGCATGACTAATGAGTATTCGCACGTGTTTGAATCTATG

BX-R_5.9 CGAGGCACTGAAGTGACATATAGCATGACTAATGAGTATTCGCACGTGTTTGAATCTATG

BX-R_5.8 CGAGGCACTGAAGTGACATATAGCATGACTAATGAGTATTCGCACGTGTTTGAATCTATG

BX-R_2.12 CGAGGCACTGAAGTGACATATAGCATGACTAATGAGTATTCGCACGTGTTTGAATCTATG

BX-R_1.40 CGAGGCACTGAAGTGACATATAGCATGACTAATGAGTATTCGCACGTGTTTGAATCTATG

BX-R_1.6 CGAGGCACTGAAGTGACATATAGCATGACTAATGAGTATTCGCACGTGTTTGAATCTATG

BX-R_1.5 CGAGGCACTGAAGTGACATATAGCATGACTAATGAGTATTCGCACGTGTTTGAATCTATG

BX-R_1_126+127 -------------TGACATATAGCATGACTAATGAGTATTCGCACGTGTTTGAATCTATG

BX-R_2_126+127 -------------TGACATATAGCATGACTAATGAGTATTCGCACGTGTTTGAATCTATG

BX-R_5_126+127 -------------TGACATATAGCATGACTAATGAGTATTCGCACGTGTTTGAATCTATG

***********************************************

MG637361.1 CTGCGCGATTTGGAGGCAAAGGCCGATGAGATAAACTTTAAAAACTACGGCCTACTGGCT 2580

BX-R_2.42 CTGCGCGATTTGGAGGCAAAAGCCGATGAGATAAACTTTAAAAACTACGGCCTACTGGCT

BX-R_2.52 CTGCGCGATTTGGAGGCAAAAGCCGATGAGATAAACTTTAAAAACTACGGCCTACTGGCT

BX-R_5.11 CTGCGCGATTTGGAGGCAAAAGCCGATGAGATAAACTTTAAAAACTACGGCCTACTGGCT

BX-R_5.9 CTGCGCGATTTGGAGGCAAAAGCCGATGAGATAAACTTTAAAAACTACGGCCTACTGGCT

BX-R_5.8 CTGCGCGATTTGGAGGCAAAAGCCGATGAGATAAACTTTAAAAACTACGGCCTACTGGCT

BX-R_2.12 CTGCGCGATTTGGAGGCAAAAGCCGATGAGATAAACTTTAAAAACTACGGCCTACTGGCT

BX-R_1.40 CTGCGCGATTTGGAGGCAAAAGCCGATGAGATAAACTTTAAAAACTACGGCCTACTGGCT

BX-R_1.6 CTGCGCGATTTGGAGGCAAAAGCCGATGAGATAAACTTTAAAAACTACGGCCTACTGGCT

BX-R_1.5 CTGCGCGATTTGGAGGCAAAAGCCGATGAGATAAACTTTAAAAACTACGGCCTACTGGCT

BX-R_1_126+127 CTGCGCGATTTGGAGGCAAAAGCCGATGAGATAAACTTTAAAAACTACGGCCTACTGGCT

BX-R_2_126+127 CTGCGCGATTTGGAGGCAAAAGCCGATGAGATAAACTTTAAAAACTACGGCCTACTGGCT

BX-R_5_126+127 CTGCGCGATTTGGAGGCAAAAGCCGATGAGATAAACTTTAAAAACTACGGCCTACTGGCT

******************** ***************************************

MG637361.1 ACTACATTAGAAGATGTGTTCATGTCCGTGGGCACAGATGTGGTCGCAACTTCAGATGTG 2640

BX-R_2.42 ACTACATTAGAAGATGTGTTCATGTCCGTGGGCACAGATGTGGTCGCAACTTCAGATGTG

BX-R_2.52 ACTACATTAGAAGATGTGTTCATGTCCGTGGGCACAGATGTGGTCGCAACTTCAGATGTG

BX-R_5.11 ACTACATTAGAAGATGTGTTCATGTCCGTGGGCACAGATGTGGTCGCAACTTCAGATGTG

BX-R_5.9 ACTACATTAGAAGATGTGTTCATGTCCGTGGGCACAGATGTGGTCGCAACTTCAGATGTG

BX-R_5.8 ACTACATTAGAAGATGTGTTCATGTCCGTGGGCACAGATGTGGTCGCAACTTCAGATGTG

BX-R_2.12 ACTACATTAGAAGATGTGTTCATGTCCGTGGGCACAGATGTGGTCGCAACTTCAGATGTG

BX-R_1.40 ACTACATTAGAAGATGTGTTCATGTCCGTGGGCACAGATGTGGTCGCAACTTCAGATGTG

BX-R_1.6 ACTACATTAGAAGATGTGTTCATGTCCGTGGGCACAGATGTGGTCGCAACTTCAGATGTG

BX-R_1.5 ACTACATTAGAAGATGTGTTCATGTCCGTGGGCACAGATGTGGTCGCAACTTCAGATGTG

BX-R_1_126+127 ACTACATTAGAAGATGTGTTCATGTCCGTGGGCACAGATGTGGTCGCAACTTCAGATGTG

BX-R_2_126+127 ACTACATTAGAAGATGTGTTCATGTCCGTGGGCACAGATGTGGTCGCAACTTCAGATGTG

BX-R_5_126+127 ACTACATTAGAAGATGTGTTCATGTCCGTGGGCACAGATGTGGTCGCAACTTCAGATGTG

************************************************************

MG637361.1 GACGACAATACAACCGTTTCATCTAGTGCTGATACTCTAGCATTTGAATATGATTCTTTA 2700

BX-R_2.42 GACGACAATACAACCGTTTCATCTAGTGCTGATACTCTAGCATTTGAATATGATTCTTTA

BX-R_2.52 GACGACAATACAACCGTTTCATCTAGTGCTGATACTCTAGCATTTGAATATGATTCTTTA

BX-R_5.11 GACGACAATACAACCGTTTCATCTAGTGCTGATACTCTAGCATTTGAATATGATTCTTTA

BX-R_5.9 GACGACAATACAACCGTTTCATCTAGTGCTGATACTCTAGCATTTGAATATGATTCTTTA

BX-R_5.8 GACGACAATACAACCGTTTCATCTAGTGCTGATACTCTAGCATTTGAATATGATTCTTTA

BX-R_2.12 GACGACAATACAACCGTTTCATCTAGTGCTGATACTCTAGCATTTGAATATGATTCTTTA

BX-R_1.40 GACGACAATACAACCGTTTCATCTAGTGCTGATACTCTAGCATTTGAATATGATTCTTTA

BX-R_1.6 GACGACAATACAACCGTTTCATCTAGTGCTGATACTCTAGCATTTGAATATGATTCTTTA

BX-R_1.5 GACGACAATACAACCGTTTCATCTAGTGCTGATACTCTAGCATTTGAATATGATTCTTTA

BX-R_1_126+127 GACGACAATACAACCGTTTCATCTAGTGCTGATACTCTAGCATTTGAATATGATTCTTTA

BX-R_2_126+127 GACGACAATACAACCGTTTCATCTAGTGCTGATACTCTAGCATTTGAATATGATTCTTTA

BX-R_5_126+127 GACGACAATACAACCGTTTCATCTAGTGCTGATACTCTAGCATTTGAATATGATTCTTTA

************************************************************

MG637361.1 GAAAAATTGGACGGGACTGGCTATGGGGATGAAAAAGGGATCCGATTAATTTGCCAACAC 2760

BX-R_2.42 GAAAAATTGGACGGGACTGGCTATGGGGATGAAAAAGGGATCCGATTAATTTCCCAACAC

BX-R_2.52 GAAAAATTGGACGGGACTGGCTATGGGGATGAAAAAGGGATCCGATTAATTTCCCAACAC

BX-R_5.11 GAAAAATTGGACGGGACTGGCTATGGGGATGAAAAAGGGATCCGATTAATTTCCCAACAC

BX-R_5.9 GAAAAATTGGACGGGACTGGCTATGGGGATGAAAAAGGGATCCGATTAATTTCCCAACAC

BX-R_5.8 GAAAAATTGGACGGGACTGGCTATGGGGATGAAAAAGGGATCCGATTAATTTCCCAACAC

BX-R_2.12 GAAAAATTGGACGGGACTGGCTATGGGGATGAAAAAGGGATCCGATTAATTTCCCAACAC

BX-R_1.40 GAAAAATTGGACGGGACTGGCTATGGGGATGAAAAAGGGATCCGATTAATTTCCCAACAC

BX-R_1.6 GAAAAATTGGACGGGACTGGCTATGGGGATGAAAAAGGGATCCGATTAATTTCCCAACAC

BX-R_1.5 GAAAAATTGGACGGGACTGGCTATGGGGATGAAAAAGGGATCCGATTAATTTCCCAACAC

BX-R_1_126+127 GAAAAATTGGACGGGACTGGCTATGGGGATGAAAAAGGGATCCGATTAATTTCCCAACAC

BX-R_2_126+127 GAAAAATTGGACGGGACTGGCTATGGGGATGAAAAAGGGATCCGATTAATTTCCCAACAC

BX-R_5_126+127 GAAAAATTGGACGGGACTGGCTATGGGGATGAAAAAGGGATCCGATTAATTTCCCAACAC

**************************************************** *******

MG637361.1 GTGGTAGCAATATGGATGAAACTGTTTCTGGTGCTGACAAGGTCTTGGCTTATCCTGTTG 2820

BX-R_2.42 GTGGTAGCAATATGGATGAAACTGTTTCTGGTGCTGACAAGGTCTTGGCTTATCCTGTTG

BX-R_2.52 GTGGTAGCAATATGGATGAAACTGTTTCTGGTGCTGACAAGGTCTTGGCTTATCCTGTTG

BX-R_5.11 GTGGTAGCAATATGGATGAAACTGTTTCTGGTGCTGACAAGGTCTTGGCTTATCCTGTTG

BX-R_5.9 GTGGTAGCAATATGGATGAAACTGTTTCTGGTGCTGACAAGGTCTTGGCTTATCCTGTTG

BX-R_5.8 GTGGTAGCAATATGGATGAAACTGTTTCTGGTGCTGACAAGGTCTTGGCTTATCCTGTTG

BX-R_2.12 GTGGTAGCAATATGGATGAAACTGTTTCTGGTGCTGACAAGGTCTTGGCTTATCCTGTTG

BX-R_1.40 GTGGTAGCAATATGGATGAAACTGTTTCTGGTGCTGACAAGGTCTTGGCTTATCCTGTTG

BX-R_1.6 GTGGTAGCAATATGGATGAAACTGTTTCTGGTGCTGACAAGGTCTTGGCTTATCCTGTTG

BX-R_1.5 GTGGTAGCAATATGGATGAAACTGTTTCTGGTGCTGACAAGGTCTTGGCTTATCCTGTTG

BX-R_1_126+127 GTGGTAGCAATATGGATGAAACTGTTTCTGGTGCTGACAAGGTCTTGGCTTATCCTGTTG

BX-R_2_126+127 GTGGTAGCAATATGGATGAAACTGTTTCTGGTGCTGACAAGGTCTTGGCTTATCCTGTTG

BX-R_5_126+127 GTGGTAGCAATATGGATGAAACTGTTTCTGGTGCTGACAAGGTCTTGGCTTATCCTGTTG

************************************************************

MG637361.1 CTCCAAGTATTGGTGTCCTTGGTACAAATCATTGCCACACTCGGAGTCATGCAGTATGTC 2880

BX-R_2.42 CTCCAAGTATTGGTGTCCTTGGTACAAATCATTGCCACACTCGGAGTCATGCAGTATGTC

BX-R_2.52 CTCCAAGTATTGGTGTCCTTGGTACAAATCATTGCCACACTCGGAGTCATGCAGTATGTC

BX-R_5.11 CTCCAAGTATTGGTGTCCTTGGTACAAATCATTGCCACACTCGGAGTCATGCAGTATGTC

BX-R_5.9 CTCCAAGTATTGGTGTCCTTGGTACAAATCATTGCCACACTCGGAGTCATGCAGTATGTC

BX-R_5.8 CTCCAAGTATTGGTGTCCTTGGTACAAATCATTGCCACACTCGGAGTCATGCAGTATGTC

BX-R_2.12 CTCCAAGTATTGGTGTCCTTGGTACAAATCATTGCCACACTCGGAGTCATGCAGTATGTC

BX-R_1.40 CTCCAAGTATTGGTGTCCTTGGTACAAATCATTGCCACACTCGGAGTCATGCAGTATGTC

BX-R_1.6 CTCCAAGTATTGGTGTCCTTGGTACAAATCATTGCCACACTCGGAGTCATGCAGTATGTC

BX-R_1.5 CTCCAAGTATTGGTGTCCTTGGTACAAATCATTGCCACACTCGGAGTCATGCAGTATGTC

BX-R_1_126+127 CTCCAAGTATTGGTGTCCTTGGTACAAATCATTGCCACACTCG-----------------

BX-R_2_126+127 CTCCAAGTATTGGTGTCCTTGGTACAAATCATTGCCACACTCG-----------------

BX-R_5_126+127 CTCCAAGTATTGGTGTCCTTGGTACAAATCATTGCCACACTCG-----------------

*******************************************

C. 86+87

MG637361.1 GTGGTAGCAATATGGATGAAACTGTTTCTGGTGCTGACAAGGTCTTGGCTTATCCTGTTG 2820

BX-R_2.42 GTGGTAGCAATATGGATGAAACTGTTTCTGGTGCTGACAAGGTCTTGGCTTATCCTGTTG

BX-R_2.52 GTGGTAGCAATATGGATGAAACTGTTTCTGGTGCTGACAAGGTCTTGGCTTATCCTGTTG

BX-R_1.5 GTGGTAGCAATATGGATGAAACTGTTTCTGGTGCTGACAAGGTCTTGGCTTATCCTGTTG

BX-R_5.9 GTGGTAGCAATATGGATGAAACTGTTTCTGGTGCTGACAAGGTCTTGGCTTATCCTGTTG

BX-R_5.11 GTGGTAGCAATATGGATGAAACTGTTTCTGGTGCTGACAAGGTCTTGGCTTATCCTGTTG

BX-R_5.8 GTGGTAGCAATATGGATGAAACTGTTTCTGGTGCTGACAAGGTCTTGGCTTATCCTGTTG

BX-R_2.12 GTGGTAGCAATATGGATGAAACTGTTTCTGGTGCTGACAAGGTCTTGGCTTATCCTGTTG

BX-R_1.6 GTGGTAGCAATATGGATGAAACTGTTTCTGGTGCTGACAAGGTCTTGGCTTATCCTGTTG

BX-R_1.40 GTGGTAGCAATATGGATGAAACTGTTTCTGGTGCTGACAAGGTCTTGGCTTATCCTGTTG

BX-R_1_86+87 ----------------------------------------------GGCTTATCCTGTTG

BX-R_2_86+87 ----------------------------------------------GGCTTATCCTGTTG

BX-R_5_86+87 ----------------------------------------------GGCTTATCCTGTTG

**************

MG637361.1 CTCCAAGTATTGGTGTCCTTGGTACAAATCATTGCCACACTCGGAGTCATGCAGTATGTC 2880

BX-R_2.42 CTCCAAGTATTGGTGTCCTTGGTACAAATCATTGCCACACTCGGAGTCATGCAGTATGTC

BX-R_2.52 CTCCAAGTATTGGTGTCCTTGGTACAAATCATTGCCACACTCGGAGTCATGCAGTATGTC

BX-R_1.5 CTCCAAGTATTGGTGTCCTTGGTACAAATCATTGCCACACTCGGAGTCATGCAGTATGTC

BX-R_5.9 CTCCAAGTATTGGTGTCCTTGGTACAAATCATTGCCACACTCGGAGTCATGCAGTATGTC

BX-R_5.11 CTCCAAGTATTGGTGTCCTTGGTACAAATCATTGCCACACTCGGAGTCATGCAGTATGTC

BX-R_5.8 CTCCAAGTATTGGTGTCCTTGGTACAAATCATTGCCACACTCGGAGTCATGCAGTATGTC

BX-R_2.12 CTCCAAGTATTGGTGTCCTTGGTACAAATCATTGCCACACTCGGAGTCATGCAGTATGTC

BX-R_1.6 CTCCAAGTATTGGTGTCCTTGGTACAAATCATTGCCACACTCGGAGTCATGCAGTATGTC

BX-R_1.40 CTCCAAGTATTGGTGTCCTTGGTACAAATCATTGCCACACTCGGAGTCATGCAGTATGTC

BX-R_1_86+87 CTCCAAGTATTGGTGTCCTTGGTACAAATCATTGCCACACTCGGAGTCATGCAGTATGTC

BX-R_2_86+87 CTCCAAGTATTGGTGTCCTTGGTACAAATCATTGCCACACTCGGAGTCATGCAGTATGTC

BX-R_5_86+87 CTCCAAGTATTGGTGTCCTTGGTACAAATCATTGCCACACTCGGAGTCATGCAGTATGTC

************************************************************

MG637361.1 ATCTCTATGACCGAGCATATACAAAGAAGAGAACTTTCATTGGCTGAAGGTTTCGCAGGC 2940

BX-R_2.42 ATCTCTATGACCGAGCATATACAAAGAAGAGAACTTTCATTGGCTGAAGGTTTCGCAGGC

BX-R_2.52 ATCTCTATGACCGAGCATATACAAAGAAGAGAACTTTCATTGGCTGAAGGTTTCGCAGGC

BX-R_1.5 ATCTCTATGACCGAGCATATACAAAGAAGAGAACTTTCATTGGCTGAAGGTTTCGCAGGC

BX-R_5.9 ATCTCTATGACCGAGCATATACAAAGAAGAGAACTTTCATTGGCTGAAGGTTTCGCAGGC

BX-R_5.11 ATCTCTATGACCGAGCATATACAAAGAAGAGAACTTTCATTGGCTGAAGGTTTCGCAGGC

BX-R_5.8 ATCTCTATGACCGAGCATATACAAAGAAGAGAACTTTCATTGGCTGAAGGTTTCGCAGGC

BX-R_2.12 ATCTCTATGACCGAGCATATACAAAGAAGAGAACTTTCATTGGCTGAAGGTTTCGCAGGC

BX-R_1.6 ATCTCTATGACCGAGCATATACAAAGAAGAGAACTTTCATTGGCTGAAGGTTTCGCAGGC

BX-R_1.40 ATCTCTATGACCGAGCATATACAAAGAAGAGAACTTTCATTGGCTGAAGGTTTCGCAGGC

BX-R_1_86+87 ATCTCTATGACCGAGCATATACAAAGAAGAGAACTTTCATTGGCTGAAGGGTTCGCAGGC

BX-R_2_86+87 ATCTCTATGACCGAGCATATACAAAGAAGAGAACTTTCATTGGCTGAAGGGTTCGCAGGC

BX-R_5_86+87 ATCTCTATGACCGAGCATATACAAAGAAGAGAACTTTCATTGGCTGAAGGGTTCGCAGGC

************************************************** *********

MG637361.1 ACAGAAACATTAGTTAGTTTCAAAGGGTTGTCCCCTACATCGACAGGTTCGCTAGCGAAG 3000

BX-R_2.42 ACAGAAACGCTAGTTAGTTTCAAAGGGTCGTCCCTTACATCGACAGGTTCGCTAGCGAAG

BX-R_2.52 ACAGAAACGCTAGTTAGTTTCAAAGGGTCGTCCCTTACATCGACAGGTTCGCTAGCGAAG

BX-R_1.5 ACAGAAACGCTAGTTAGTTTCAAAGGGTCGTCCCTTACATCGACAGGTTCGCTAGCGAAG

BX-R_5.9 ACAGAAACGCTAGTTAGTTTCAAAGGGTCGTCCCTTACATCGACAGGTTCGCTAGCGAAG

BX-R_5.11 ACAGAAACGCTAGTTAGTTTCAAAGGGTCGTCCCTTACATCGACAGGTTCGCTAGCGAAG

BX-R_5.8 ACAGAAACGCTAGTTAGTTTCAAAGGGTCGTCCCTTACATCGACAGGTTCGCTAGCGAAG

BX-R_2.12 ACAGAAACGCTAGTTAGTTTCAAAGGGTCGTCCCTTACATCGACAGGTTCGCTAGCGAAG

BX-R_1.6 ACAGAAACGCTAGTTAGTTTCAAAGGGTCGTCCCTTACATCGACAGGTTCGCTAGCGAAG

BX-R_1.40 ACAGAAACGCTAGTTAGTTTCAAAGGGTCGTCCCTTACATCGACAGGTTCGCTAGCGAAG

BX-R_1_86+87 ACAGAAACGCTAGTTAGTTTCAAAGGGTCGTCCCTTACATCGACAGGTTCGCTAGCGAAG

BX-R_2_86+87 ACAGAAACGCTAGTTAGTTTCAAAGGGTCGTCCCTTACATCGACAGGTTCGCTAGCGAAG

BX-R_5_86+87 ACAGAAACGCTAGTTAGTTTCAAAGGGTCGTCCCTTACATCGACAGGTTCGCTAGCGAAG

******** ****************** ***** *************************

MG637361.1 GCTGCCTACGAGTCGATATTTGTAACCGCCAATAATCCCACAATGGAAATCACTGTTGTT 3060

BX-R_2.42 GCTGCCTACGAGTCGATATTTGTAACCGCCAATAATCCCACAATGGAAATCACTGTTGTT

BX-R_2.52 GCTGCCTACGAGTCGATATTTGTAACCGCCAATAATCCCACAATGGAAATCACTGTTGTT

BX-R_1.5 GCTGCCTACGAGTCGATATTTGTAACCGCCAATAATCCCACAATGGAAATCACTGTTGTT

BX-R_5.9 GCTGCCTACGAGTCGATATTTGTAACCGCCAATAATCCCACAATGGAAATCACTGTTGTT

BX-R_5.11 GCTGCCTACGAGTCGATATTTGTAACCGCCAATAATCCCACAATGGAAATCACTGTTGTT

BX-R_5.8 GCTGCCTACGAGTCGATATTTGTAACCGCCAATAATCCCACAATGGAAATCACTGTTGTT

BX-R_2.12 GCTGCCTACGAGTCGATATTTGTAACCGCCAATAATCCCACAATGGAAATCACTGTTGTT

BX-R_1.6 GCTGCCTACGAGTCGATATTTGTAACCGCCAATAATCCCACAATGGAAATCACTGTTGTT

BX-R_1.40 GCTGCCTACGAGTCGATATTTGTAACCGCCAATAATCCCACAATGGAAATCACTGTTGTT

BX-R_1_86+87 GCTGCCTACGAGTCGATATTTGTAACCGCCAATAATCCC---------------------

BX-R_2_86+87 GCTGCCTACGAGTCGATATTTGTAACCGCCAATAATCCC---------------------

BX-R_5_86+87 GCTGCCTACGAGTCGATATTTGTAACCGCCAATAATCCC---------------------

***************************************

D. 124+154

MG637361.1 ACAGAAACATTAGTTAGTTTCAAAGGGTTGTCCCCTACATCGACAGGTTCGCTAGCGAAG 3000

BX-R_2.42 ACAGAAACGCTAGTTAGTTTCAAAGGGTCGTCCCTTACATCGACAGGTTCGCTAGCGAAG

BX-R_2.52 ACAGAAACGCTAGTTAGTTTCAAAGGGTCGTCCCTTACATCGACAGGTTCGCTAGCGAAG

BX-R_1.5 ACAGAAACGCTAGTTAGTTTCAAAGGGTCGTCCCTTACATCGACAGGTTCGCTAGCGAAG

BX-R_5.11 ACAGAAACGCTAGTTAGTTTCAAAGGGTCGTCCCTTACATCGACAGGTTCGCTAGCGAAG

BX-R_5.9 ACAGAAACGCTAGTTAGTTTCAAAGGGTCGTCCCTTACATCGACAGGTTCGCTAGCGAAG

BX-R_5.8 ACAGAAACGCTAGTTAGTTTCAAAGGGTCGTCCCTTACATCGACAGGTTCGCTAGCGAAG

BX-R_2.12 ACAGAAACGCTAGTTAGTTTCAAAGGGTCGTCCCTTACATCGACAGGTTCGCTAGCGAAG

BX-R_1.40 ACAGAAACGCTAGTTAGTTTCAAAGGGTCGTCCCTTACATCGACAGGTTCGCTAGCGAAG

BX-R_1.6 ACAGAAACGCTAGTTAGTTTCAAAGGGTCGTCCCTTACATCGACAGGTTCGCTAGCGAAG

BX-R_1_124+154 -----------------------------------------------TTCGCTAGCGAAG

BX-R_2_124+154 -----------------------------------------------TTCGCTAGCGAAG

*************

MG637361.1 GCTGCCTACGAGTCGATATTTGTAACCGCCAATAATCCCACAATGGAAATCACTGTTGTT 3060

BX-R_2.42 GCTGCCTACGAGTCGATATTTGTAACCGCCAATAATCCCACAATGGAAATCACTGTTGTT

BX-R_2.52 GCTGCCTACGAGTCGATATTTGTAACCGCCAATAATCCCACAATGGAAATCACTGTTGTT

BX-R_1.5 GCTGCCTACGAGTCGATATTTGTAACCGCCAATAATCCCACAATGGAAATCACTGTTGTT

BX-R_5.11 GCTGCCTACGAGTCGATATTTGTAACCGCCAATAATCCCACAATGGAAATCACTGTTGTT

BX-R_5.9 GCTGCCTACGAGTCGATATTTGTAACCGCCAATAATCCCACAATGGAAATCACTGTTGTT

BX-R_5.8 GCTGCCTACGAGTCGATATTTGTAACCGCCAATAATCCCACAATGGAAATCACTGTTGTT

BX-R_2.12 GCTGCCTACGAGTCGATATTTGTAACCGCCAATAATCCCACAATGGAAATCACTGTTGTT

BX-R_1.40 GCTGCCTACGAGTCGATATTTGTAACCGCCAATAATCCCACAATGGAAATCACTGTTGTT

BX-R_1.6 GCTGCCTACGAGTCGATATTTGTAACCGCCAATAATCCCACAATGGAAATCACTGTTGTT

BX-R_1_124+154 GCTGCCTACGAGTCGATATTTGTAACCGCCAATAATCCCACAATGGAAATCACTGTTGTT

BX-R_2_124+154 GCTGCCTACGAGTCGATATTTGTAACCGCCAATAATCCCACAATGGAAATCACTGTTGTT

************************************************************

MG637361.1 GATAATACACCTATAGATGAATATTATTTGGAAAGAACAGATGACGTATCAGCGATGGCG 3120

BX-R_2.42 GATAATACACCTATAGATGAATATTATTTGGAAAGA----ATGACGTATCAGCGATGGCG

BX-R_2.52 GATAATACACCTATAGATGAATATTATTTGGAAAGA----ATGACGTATCAGCGATGGCG

BX-R_1.5 GATAATACACCTATAGATGAATATTATTTGGAAAGA----ATGACGTATCAGCGATGGCG

BX-R_5.11 GATAATACACCTATAGATGAATATTATTTGGAAAGAACAGATGACGTATCAGCGATGGCG

BX-R_5.9 GATAATACACCTATAGATGAATATTATTTGGAAAGAACAGATGACGTATCAGCGATGGCG

BX-R_5.8 GATAATACACCTATAGATGAATATTATTTGGAAAGAACAGATGACGTATCAGCGATGGCG

BX-R_2.12 GATAATACACCTATAGATGAATATTATTTGGAAAGAACAGATGACGTATCAGCGATGGCG

BX-R_1.40 GATAATACACCTATAGATGAATATTATTTGGAAAGAACAGATGACGTATCAGCGATGGCG

BX-R_1.6 GATAATACACCTATAGATGAATATTATTTGGAAAGAACAGATGACGTATCAGCGATGGCG

BX-R_1_124+154 GATAATACACCTATAGATGAATATTATTTGGAAAGAACAGATGACGTATCAGCGATGGCG

BX-R_2_124+154 GATAATACACCTATAGATGAATATTATTTGGAAAGAACAGATGACGTATCAGCGATGGCG

************************************ ********************

MG637361.1 GTGCTCCGGCACAGTCTGTTGATCGGCGCGACGTTCGACGACCACTCCGCGACCGCGTGG 3180

BX-R_2.42 GTGCTCCGGCACAGTCTGTTGATCGGCGCGACGTTCGACGACAACTCCGCGACCGCGTGG

BX-R_2.52 GTGCTCCGGCACAGTCTGTTGATCGGCGCGACGTTCGACGACAACTCCGCGACCGCGTGG

BX-R_1.5 GTGCTCCGGCACAGTCTGTTGATCGGCGCGACGTTCGACGACAACTCCGCGACCGCGTGG

BX-R_5.11 GTGCTCCGGCACAGTCTGTTGATCGGCGCGACGTTCGACGACAACTCCGCGACCGCGTGG

BX-R_5.9 GTGCTCCGGCACAGTCTGTTGATCGGCGCGACGTTCGACGACAACTCCGCGACCGCGTGG

BX-R_5.8 GTGCTCCGGCACAGTCTGTTGATCGGCGCGACGTTCGACGACAACTCCGCGACCGCGTGG

BX-R_2.12 GTGCTCCGGCACAGTCTGTTGATCGGCGCGACGTTCGACGACAACTCCGCGACCGCGTGG

BX-R_1.40 GTGCTCCGGCACAGTCTGTTGATCGGCGCGACGTTCGACGACAACTCCGCGACCGCGTGG

BX-R_1.6 GTGCTCCGGCACAGTCTGTTGATCGGCGCGACGTTCGACGACAACTCCGCGACCGCGTGG

BX-R_1_124+154 GTGCTCCGGCACAGTCTGTTGATCGGCGCGACGTTCGAC---------------------

BX-R_2_124+154 GTGCTCCGGCACAGTCTGTTGATCGGCGCGACGTTCGAC---------------------

***************************************

E. 85+88

MG637361.1 GATAATACACCTATAGATGAATATTATTTGGAAAGAACAGATGACGTATCAGCGATGGCG 3120

BX-R_2.42 GATAATACACCTATAGATGAATATTATTTGGAAAG----AATGACGTATCAGCGATGGCG

BX-R_2.52 GATAATACACCTATAGATGAATATTATTTGGAAAG----AATGACGTATCAGCGATGGCG

BX-R_1.5 GATAATACACCTATAGATGAATATTATTTGGAAAG----AATGACGTATCAGCGATGGCG

BX-R_5.11 GATAATACACCTATAGATGAATATTATTTGGAAAGAACAGATGACGTATCAGCGATGGCG

BX-R_5.9 GATAATACACCTATAGATGAATATTATTTGGAAAGAACAGATGACGTATCAGCGATGGCG

BX-R_5.8 GATAATACACCTATAGATGAATATTATTTGGAAAGAACAGATGACGTATCAGCGATGGCG

BX-R_2.12 GATAATACACCTATAGATGAATATTATTTGGAAAGAACAGATGACGTATCAGCGATGGCG

BX-R_1.40 GATAATACACCTATAGATGAATATTATTTGGAAAGAACAGATGACGTATCAGCGATGGCG

BX-R_1.6 GATAATACACCTATAGATGAATATTATTTGGAAAGAACAGATGACGTATCAGCGATGGCG

BX-R_1_85+88 --------------------------------------AGATGACGTATCAGCGATGGCG

BX-R_2_85+88 --------------------------------------AGATGACGTATCAGCGATGGCG

BX-R_5_85+88 --------------------------------------AGATGACGTATCAGCGATGGCG

********************

MG637361.1 GTGCTCCGGCACAGTCTGTTGATCGGCGCGACGTTCGACGACCACTCCGCGACCGCGTGG 3180

BX-R_2.42 GTGCTCCGGCACAGTCTGTTGATCGGCGCGACGTTCGACGACAACTCCGCGACCGCGTGG

BX-R_2.52 GTGCTCCGGCACAGTCTGTTGATCGGCGCGACGTTCGACGACAACTCCGCGACCGCGTGG

BX-R_1.5 GTGCTCCGGCACAGTCTGTTGATCGGCGCGACGTTCGACGACAACTCCGCGACCGCGTGG

BX-R_5.11 GTGCTCCGGCACAGTCTGTTGATCGGCGCGACGTTCGACGACAACTCCGCGACCGCGTGG

BX-R_5.9 GTGCTCCGGCACAGTCTGTTGATCGGCGCGACGTTCGACGACAACTCCGCGACCGCGTGG

BX-R_5.8 GTGCTCCGGCACAGTCTGTTGATCGGCGCGACGTTCGACGACAACTCCGCGACCGCGTGG

BX-R_2.12 GTGCTCCGGCACAGTCTGTTGATCGGCGCGACGTTCGACGACAACTCCGCGACCGCGTGG

BX-R_1.40 GTGCTCCGGCACAGTCTGTTGATCGGCGCGACGTTCGACGACAACTCCGCGACCGCGTGG

BX-R_1.6 GTGCTCCGGCACAGTCTGTTGATCGGCGCGACGTTCGACGACAACTCCGCGACCGCGTGG

BX-R_1_85+88 GTGCTCCGGCACAGTCTGTTGATCGGCGCGACGTTCGACGACAACTCCGCGACCGCGTGG

BX-R_2_85+88 GTGCTCCGGCACAGTCTGTTGATCGGCGCGACGTTCGACGACAACTCCGCGACCGCGTGG

BX-R_5_85+88 GTGCTCCGGCACAGTCTGTTGATCGGCGCGACGTTCGACGACAACTCCGCGACCGCGTGG

****************************************** *****************

MG637361.1 TTCAGCAACTTCGGTTACCACGACGTGGCCATGTCACTGGCTGCTGTGCACGCCGCCTTG 3240

BX-R_2.42 TTCAGCAACTTCGGTTACCACGACGTGGCCATGTCACTGGCGGCTGTGCACGCCGCCTTG

BX-R_2.52 TTCAGCAACTTCGGTTACCACGACGTGGCCATGTCACTGGCGGCTGTGCACGCCGCCTTG

BX-R_1.5 TTCAGCAACTTCGGTTACCACGACGTGGCCATGTCACTGGCGGCTGTGCACGCCGCCTTG

BX-R_5.11 TTCAGCAACTTCGGTTACCACGACGTGGCCATGTCACTGGCGGCTGTGCACGCCGCCTTG

BX-R_5.9 TTCAGCAACTTCGGTTACCACGACGTGGCCATGTCACTGGCGGCTGTGCACGCCGCCTTG

BX-R_5.8 TTCAGCAACTTCGGTTACCACGACGTGGCCATGTCACTGGCGGCTGTGCACGCCGCCTTG

BX-R_2.12 TTCAGCAACTTCGGTTACCACGACGTGGCCATGTCACTGGCGGCTGTGCACGCCGCCTTG

BX-R_1.40 TTCAGCAACTTCGGTTACCACGACGTGGCCATGTCACTGGCGGCTGTGCACGCCGCCTTG

BX-R_1.6 TTCAGCAACTTCGGTTACCACGACGTGGCCATGTCACTGGCGGCTGTGCACGCCGCCTTG

BX-R_1_85+88 TTCAGCAACTTCGGTTACCACGACGTGGCCATGTCACTGGCGGCTGTGCACGCCGCCTTG

BX-R_2_85+88 TTCAGCAACTTCGGTTACCACGACGTGGCCATGTCACTGGCGGCTGTGCACGCCGCCTTG

BX-R_5_85+88 TTCAGCAACTTCGGTTACCACGACGTGGCCATGTCACTGGCGGCTGTGCACGCCGCCTTG

***************************************** ******************

MG637361.1 CTCAGAGCTGTCAATCCTGCAGCCAACTTGACTGTTTACAACCACCCACTTGAGGCCAAT 3300

BX-R_2.42 CTCAGAGCTGTCAATCCTGCAGCCAACTTGACTGTTTACAACCACCCACTTGAGGCCAAT

BX-R_2.52 CTCAGAGCTGTCAATCCTGCAGCCAACTTGACTGTTTACAACCACCCACTTGAGGCCAAT

BX-R_1.5 CTCAGAGCTGTCAATCCTGCAGCCAACTTGACTGTTTACAACCACCCACTTGAGGCCAAT

BX-R_5.11 CTCAGAGCTGTCAATCCTGCAGCCAACTTGACTGTTTACAACCACCCACTTGAGGCCAAT

BX-R_5.9 CTCAGAGCTGTCAATCCTGCAGCCAACTTGACTGTTTACAACCACCCACTTGAGGCCAAT

BX-R_5.8 CTCAGAGCTGTCAATCCTGCAGCCAACTTGACTGTTTACAACCACCCACTTGAGGCCAAT

BX-R_2.12 CTCAGAGCTGTCAATCCTGCAGCCAACTTGACTGTTTACAACCACCCACTTGAGGCCAAT

BX-R_1.40 CTCAGAGCTGTCAATCCTGCAGCCAACTTGACTGTTTACAACCACCCACTTGAGGCCAAT

BX-R_1.6 CTCAGAGCTGTCAATCCTGCAGCCAACTTGACTGTTTACAACCACCCACTTGAGGCCAAT

BX-R_1_85+88 CTCAGAGCTGTCAATCCTGCAGCCAACTTGACT---------------------------

BX-R_2_85+88 CTCAGAGCTGTCAATCCTGCAGCCAACTTGACT---------------------------

BX-R_5_85+88 CTCAGAGCTGTCAATCCTGCAGCCAACTTGACT---------------------------

*********************************

F. rA1-F+82

MG637361.1 CGAGTATCTCGCGCCAAGCTGCTGCAGAAGGCGGCAGGCATCCAGCCGTTAGTGATGTGG 3480

BX-R_2.42 CGAGTGTCTCGCGCCAAGCTGCTGCAGAAGGCGGCAGGCATCCAGCCGTTAGTGATGTGG

BX-R_2.52 CGAGTGTCTCGCGCCAAGCTGCTGCAGAAGGCGGCAGGCATCCAGCCGTTAGTGATGTGG

BX-R_5.11 CGAGTATCTCGCGCCAAGCTGCTGCAGAAGGCGGCAGGCATCCAGCCGTTAGTGATGTGG

BX-R_5.9 CGAGTATCTCGCGCCAAGCTGCTGCAGAAGGCGGCAGGCATCCAGCCGTTAGTGATGTGG

BX-R_5.8 CGAGTATCTCGCGCCAAGCTGCTGCAGAAGGCGGCAGGCATCCAGCCGTTAGTGATGTGG

BX-R_2.12 CGAGTATCTCGCGCCAAGCTGCTGCAGAAGGCGGCAGGCATCCAGCCGTTAGTGATGTGG

BX-R_1.40 CGAGTATCTCGCGCCAAGCTGCTGCAGAAGGCGGCAGGCATCCAGCCGTTAGTGATGTGG

BX-R_1.6 CGAGTATCTCGCGCCAAGCTGCTGCAGAAGGCGGCAGGCATCCAGCCGTTAGTGATGTGG

BX-R_1.5 CGAGTATCTCGCGCCAAGCTGCTGCAGAAGGCGGCAGGCATCCAGCCGTTAGTGATGTGG

BX-R_1_rA1-F+82 -----------CGCCAAGCTGCTGCAGAAGGCGGCAGGCATCCAGCCGTTAGTGATGTGG

BX-R_2_rA1-F+82 -----------CGCCAAGCTGCTGCAGAAGGCGGCAGGCATCCAGCCGTTAGTGATGTGG

BX-R_5_rA1-F+82 -----------CGCCAAGCTGCTGCAGAAGGCGGCAGGCATCCAGCCGTTAGTGATGTGG

*************************************************

MG637361.1 CTCAGCGCCGCCGTGTTCGACTGGATCTGGTTCTGCGTCATCGCCGTCGGCATCGTTATC 3540

BX-R_2.42 CTCAGCGCCGCCGTGTTCGACTGGATCTGGTTCTGCATCATCGCCGTCGGCATCGTTATC

BX-R_2.52 CTCAGCGCCGCCGTGTTCGACTGGATCTGGTTCTGCATCATCGCCGTCGGCATCGTTATC

BX-R_5.11 CTCAGCGCCGCCGTGTTCGACTGGATCTGGTTCTGCATCATCGCCGTCGGCATCGTTATC

BX-R_5.9 CTCAGCGCCGCCGTGTTCGACTGGATCTGGTTCTGCATCATCGCCGTCGGCATCGTTATC

BX-R_5.8 CTCAGCGCCGCCGTGTTCGACTGGATCTGGTTCTGCATCATCGCCGTCGGCATCGTTATC

BX-R_2.12 CTCAGCGCCGCCGTGTTCGACTGGATCTGGTTCTGCATCATCGCCGTCGGCATCGTTATC

BX-R_1.40 CTCAGCGCCGCCGTGTTCGACTGGATCTGGTTCTGCATCATCGCCGTCGGCATCGTTATC

BX-R_1.6 CTCAGCGCCGCCGTGTTCGACTGGATCTGGTTCTGCATCATCGCCGTCGGCATCGTTATC

BX-R_1.5 CTCAGCGCCGCCGTGTTCGACTGGATCTGGTTCTGCATCATCGCCGTCGGCATCGTTATC

BX-R_1_rA1-F+82 CTCAGCGCCGCCGTGTTCGACTGGATCTGGTTCTGCATCATCGCCGTCGGCATCGTTATC

BX-R_2_rA1-F+82 CTCAGCGCCGCCGTGTTCGACTGGATCTGGTTCTGCATCATCGCCGTCGGCATCGTTATC

BX-R_5_rA1-F+82 CTCAGCGCCGCCGTGTTCGACTGGATCTGGTTCTGCATCATCGCCGTCGGCATCGTTATC

************************************ ***********************

MG637361.1 GCCTGCGCCGCTTTTAACGTCATTGGGCTCTCTTCTGTCGATGAACTGGGTCGGATGTAC 3600

BX-R_2.42 GCCTGCGCCGCTTTTAACGTCATTGGGCTCTCTTCTGTCGATGAACTGGGTCGGATGTAC

BX-R_2.52 GCCTGCGCCGCTTTTAACGTCATTGGGCTCTCTTCTGTCGATGAACTGGGTCGGATGTAC

BX-R_5.11 GCCTGCGCCGCTTTTAACGTCATTGGGCTCTCTTCTGTCGATGAACTGGGTCGGATGTAC

BX-R_5.9 GCCTGCGCCGCTTTTAACGTCATTGGGCTCTCTTCTGTCGATGAACTGGGTCGGATGTAC

BX-R_5.8 GCCTGCGCCGCTTTTAACGTCATTGGGCTCTCTTCTGTCGATGAACTGGGTCGGATGTAC

BX-R_2.12 GCCTGCGCCGCTTTTAACGTCATTGGGCTCTCTTCTGTCGATGAACTGGGTCGGATGTAC

BX-R_1.40 GCCTGCGCCGCTTTTAACGTCATTGGGCTCTCTTCTGTCGATGAACTGGGTCGGATGTAC

BX-R_1.6 GCCTGCGCCGCTTTTAACGTCATTGGGCTCTCTTCTGTCGATGAACTGGGTCGGATGTAC

BX-R_1.5 GCCTGCGCCGCTTTTAACGTCATTGGGCTCTCTTCTGTCGATGAACTGGGTCGGATGTAC

BX-R_1_rA1-F+82 GCCTGCGCCGCTTTTAACGTCATTGGGCTCTCTTCTGTCGATGAACTGGGTCGGATGTAC

BX-R_2_rA1-F+82 GCCTGCGCCGCTTTTAACGTCATTGGGCTCTCTTCTGTCGATGAACTGGGTCGGATGTAC

BX-R_5_rA1-F+82 GCCTGCGCCGCTTTTAACGTCATTGGGCTCTCTTCTGTCGATGAACTGGGTCGGATGTAC

************************************************************

MG637361.1 TTGTGCATCATAGTGTATGGCGCCGCCAGTCTACCGATAGGCTACGTGTTCTCCTATTTC 3660

BX-R_2.42 TTGTGCATCATAGTGTATGGCGCCGCCAGTCTGCCGATAGGCTACGTGTTCTCCTATTTC

BX-R_2.52 TTGTGCATCATAGTGTATGGCGCCGCCAGTCTGCCGATAGGCTACGTGTTCTCCTATTTC

BX-R_5.11 TTGTGCATCATAGTGTATGGCGCCGCCAGTCTGCCGATAGGCTACGTGTTCTCCTATTTC

BX-R_5.9 TTGTGCATCATAGTGTATGGCGCCGCCAGTCTGCCGATAGGCTACGTGTTCTCCTATTTC

BX-R_5.8 TTGTGCATCATAGTGTATGGCGCCGCCAGTCTGCCGATAGGCTACGTGTTCTCCTATTTC

BX-R_2.12 TTGTGCATCATAGTGTATGGCGCCGCCAGTCTGCCGATAGGCTACGTGTTCTCCTATTTC

BX-R_1.40 TTGTGCATCATAGTGTATGGCGCCGCCAGTCTGCCGATAGGCTACGTGTTCTCCTATTTC

BX-R_1.6 TTGTGCATCATAGTGTATGGCGCCGCCAGTCTGCCGATAGGCTACGTGTTCTCCTATTTC

BX-R_1.5 TTGTGCATCATAGTGTATGGCGCCGCCAGTCTGCCGATAGGCTACGTGTTCTCCTATTTC

BX-R_1_rA1-F+82 TTGTGCATCATAGTGTATGGCGC-------------------------------------

BX-R_2_rA1-F+82 TTGTGCATCATAGTGTATGGCGC-------------------------------------

BX-R_5_rA1-F+82 TTGTGCATCATAGTGTATGGCGC-------------------------------------

***********************

G. 89+90

MG637361.1 ATGGGGGCGCAGATTGTGGAGGCCTTGTTGTCACCGCAGCTTGATACTGAAAATGTCGCT 3780

BX-R_2.42 ATGGGGGCGCAGATTGTGGAGGCCTTGTTGTCACCGCAGCTTGATACTGAAAATGTCGCT

BX-R_2.52 ATGGGGGCGCAGATTGTGGAGGCCTTGTTGTCACCGCAGCTTGATACTGAAAATGTCGCT

BX-R_5.11 ATGGGGGCGCAGATTGTGGAGGCCTTGTTGTCACCGCAGCTTGATACTGAAAATGTCGCT

BX-R_5.9 ATGGGGGCGCAGATTGTGGAGGCCTTGTTGTCACCGCAGCTTGATACTGAAAATGTCGCT

BX-R_5.8 ATGGGGGCGCAGATTGTGGAGGCCTTGTTGTCACCGCAGCTTGATACTGAAAATGTCGCT

BX-R_2.12 ATGGGGGCGCAGATTGTGGAGGCCTTGTTGTCACCGCAGCTTGATACTGAAAATGTCGCT

BX-R_1.40 ATGGGGGCGCAGATTGTGGAGGCCTTGTTGTCACCGCAGCTTGATACTGAAAATGTCGCT

BX-R_1.6 ATGGGGGCGCAGATTGTGGAGGCCTTGTTGTCACCGCAGCTTGATACTGAAAATGTCGCT

BX-R_1.5 ATGGGGGCGCAGATTGTGGAGGCCTTGTTGTCACCGCAGCTTGATACTGAAAATGTCGCT

BX-R_1_89+90 -------------------------------CACCGCAGCTTGATACTGAAAATGTCGCT

BX-R_2_89+90 -------------------------------CACCGCAGCTTGATACTGAAAATGTCGCT

BX-R_5_89+90 -------------------------------CACCGCAGCTTGATACTGAAAATGTCGCT

*****************************

MG637361.1 AATATACTTGACTCCATCTTGCAATTCTTCCCACTCTATAGTCTTGTCACATCTGCCAGA 3840

BX-R_2.42 AATATACTTGACTCCATCTTGCAATTCTTCCCACTCTATGGTCTTGTCACATCTGCCAGA

BX-R_2.52 AATATACTTGACTCCATCTTGCAATTCTTCCCACTCTATGGTCTTGTCACATCTGCCAGA

BX-R_5.11 AATATACTTGACTCCATCTTGCAATTCTTCCCACTCTATGGTCTTGTCACATCTGCCAGA

BX-R_5.9 AATATACTTGACTCCATCTTGCAATTCTTCCCACTCTATGGTCTTGTCACATCTGCCAGA

BX-R_5.8 AATATACTTGACTCCATCTTGCAATTCTTCCCACTCTATGGTCTTGTCACATCTGCCAGA

BX-R_2.12 AATATACTTGACTCCATCTTGCAATTCTTCCCACTCTATGGTCTTGTCACATCTGCCAGA

BX-R_1.40 AATATACTTGACTCCATCTTGCAATTCTTCCCACTCTATGGTCTTGTCACATCTGCCAGA

BX-R_1.6 AATATACTTGACTCCATCTTGCAATTCTTCCCACTCTATGGTCTTGTCACATCTGCCAGA

BX-R_1.5 AATATACTTGACTCCATCTTGCAATTCTTCCCACTCTATGGTCTTGTCACATCTGCCAGA

BX-R_1_89+90 AATATACTTGACTCCATCTTGCAATTCTTCCCACTCTATGGTCTTGTCACATCTGCCAGA

BX-R_2_89+90 AATATACTTGACTCCATCTTGCAATTCTTCCCACTCTATGGTCTTGTCACATCTGCCAGA

BX-R_5_89+90 AATATACTTGACTCCATCTTGCAATTCTTCCCACTCTATGGTCTTGTCACATCTGCCAGA

*************************************** ********************

MG637361.1 CTGTTGAATCAGGTGGGACTGCTGGAGTGGTCGTGCCTGCAGAACTGCGAGTACCTGTCC 3900

BX-R_2.42 CTGTTGAATCAGGTGGGACTGCTGGAGTGGTCATGCCTGCAGAACTGCGAGTACCTGTCC

BX-R_2.52 CTGTTGAATCAGGTGGGACTGCTGGAGTGGTCATGCCTGCAGAACTGCGAGTACCTGTCC

BX-R_5.11 CTGTTGAATCAGGTGGGACTGCTGGAGTGGTCATGCCTGCAGAACTGCGAGTACCTGTCC

BX-R_5.9 CTGTTGAATCAGGTGGGACTGCTGGAGTGGTCATGCCTGCAGAACTGCGAGTACCTGTCC

BX-R_5.8 CTGTTGAATCAGGTGGGACTGCTGGAGTGGTCATGCCTGCAGAACTGCGAGTACCTGTCC

BX-R_2.12 CTGTTGAATCAGGTGGGACTGCTGGAGTGGTCATGCCTGCAGAACTGCGAGTACCTGTCC

BX-R_1.40 CTGTTGAATCAGGTGGGACTGCTGGAGTGGTCATGCCTGCAGAACTGCGAGTACCTGTCC

BX-R_1.6 CTGTTGAATCAGGTGGGACTGCTGGAGTGGTCATGCCTGCAGAACTGCGAGTACCTGTCC

BX-R_1.5 CTGTTGAATCAGGTGGGACTGCTGGAGTGGTCATGCCTGCAGAACTGCGAGTACCTGTCC

BX-R_1_89+90 CTGTTGAATCAGGTGGGACTGCTGGAGTGGTCATGCCTGCAGAACTGCGAGTACCTGTCC

BX-R_2_89+90 CTGTTGAATCAGGTGGGACTGCTGGAGTGGTCATGCCTGCAGAACTGCGAGTACCTGTCC

BX-R_5_89+90 CTGTTGAATCAGGTGGGACTGCTGGAGTGGTCATGCCTGCAGAACTGCGAGTACCTGTCC

******************************** ***************************

MG637361.1 GCAGTGATGCCCAACTTGACCGAATGCTCCATGGACGTTATGTGCCAGACGTTCTCACAA 3960

BX-R_2.42 GCAGTGATGCCCAACTTGACCGAATGCTCCATGGACGTAATGTGCCAGACGTTCTCACAA

BX-R_2.52 GCAGTGATGCCCAACTTGACCGAATGCTCCATGGACGTAATGTGCCAGACGTTCTCACAA

BX-R_5.11 GCAGTGATGCCCAACTTGACCGAATGCTCCATGGACGTAATGTGCCAGACGTTCTCACAA

BX-R_5.9 GCAGTGATGCCCAACTTGACCGAATGCTCCATGGACGTAATGTGCCAGACGTTCTCACAA

BX-R_5.8 GCAGTGATGCCCAACTTGACCGAATGCTCCATGGACGTAATGTGCCAGACGTTCTCACAA

BX-R_2.12 GCAGTGATGCCCAACTTGACCGAATGCTCCATGGACGTAATGTGCCAGACGTTCTCACAA

BX-R_1.40 GCAGTGATGCCCAACTTGACCGAATGCTCCATGGACGTAATGTGCCAGACGTTCTCACAA

BX-R_1.6 GCAGTGATGCCCAACTTGACCGAATGCTCCATGGACGTAATGTGCCAGACGTTCTCACAA

BX-R_1.5 GCAGTGATGCCCAACTTGACCGAATGCTCCATGGACGTAATGTGCCAGACGTTCTCACAA

BX-R_1_89+90 GCAGTGATGCCCAACTTGACCGAATGCTCCATGGACGTAATGTGCCAGACGTTCTCACA-

BX-R_2_89+90 GCAGTGATGCCCAACTTGACCGAATGCTCCATGGACGTAATGTGCCAGACGTTCTCACA-

BX-R_5_89+90 GCAGTGATGCCCAACTTGACCGAATGCTCCATGGACGTAATGTGCCAGACGTTCTCACA-

************************************** ********************

H. 143+90

MG637361.1 AAAGAAATGCAATTGAAGGAAACGATGAAGATCATGGGACTCCCAACGTGGCTGCATTGG 1020

BX-R_2.42 AAAGAAATGCAATTGAAGGAAACGATGAAGATCATGGGACTCCCAACGTGGCTGCATTGG

BX-R_2.52 AAAGAAATGCAATTGAAGGAAACGATGAAGATCATGGGACTCCCAACGTGGCTGCATTGG

BX-R_5.11 AAAGAAATGCAATTGAAGGAAACGATGAAGATCATGGGACTCCCAACGTGGCTGCATTGG

BX-R_5.9 AAAGAAATGCAATTGAAGGAAACGATGAAGATCATGGGACTCCCAACGTGGCTGCATTGG

BX-R_5.8 AAAGAAATGCAATTGAAGGAAACGATGAAGATCATGGGACTCCCAACGTGGCTGCATTGG

BX-R_2.12 AAAGAAATGCAATTGAAGGAAACGATGAAGATCATGGGACTCCCAACGTGGCTGCATTGG

BX-R_1.40 AAAGAAATGCAATTGAAGGAAACGATGAAGATCATGGGACTCCCAACGTGGCTGCATTGG

BX-R_1.6 AAAGAAATGCAATTGAAGGAAACGATGAAGATCATGGGACTCCCAACGTGGCTGCATTGG

BX-R_1.5 AAAGAAATGCAATTGAAGGAAACGATGAAGATCATGGGACTCCCAACGTGGCTGCATTGG

BX-R_2_143+90 --------------------------------------ACTCCCAACGTGGCTGCATTGG

BX-R_5_143+90 --------------------------------------ACTCCCAACGTGGCTGCATTGG

**********************

MG637361.1 ATGGCATGGTTTTTTAAACAATTTATTTATTTGCTGATTGCTTCGGTTTTGATACTTGTT 1080

BX-R_2.42 ATGGCATGGTTTTTTAAACAATTTATTTATTTGCTAATTGCTTCGGTTTTGATACTTGTT

BX-R_2.52 ATGGCATGGTTTTTTAAACAATTTATTTATTTGCTAATTGCTTCGGTTTTGATACTTGTT

BX-R_5.11 ATGGCATGGTTTTTTAAACAATTTATTTATTTGCTAATTGCTTCGGTTTTGATACTTGTT

BX-R_5.9 ATGGCATGGTTTTTTAAACAATTTATTTATTTGCTAATTGCTTCGGTTTTGATACTTGTT

BX-R_5.8 ATGGCATGGTTTTTTAAACAATTTATTTATTTGCTAATTGCTTCGGTTTTGATACTTGTT

BX-R_2.12 ATGGCATGGTTTTTTAAACAATTTATTTATTTGCTAATTGCTTCGGTTTTGATACTTGTT

BX-R_1.40 ATGGCATGGTTTTTTAAACAATTTATTTATTTGCTAATTGCTTCGGTTTTGATACTTGTT

BX-R_1.6 ATGGCATGGTTTTTTAAACAATTTATTTATTTGCTAATTGCTTCGGTTTTGATACTTGTT

BX-R_1.5 ATGGCATGGTTTTTTAAACAATTTATTTATTTGCTAATTGCTTCGGTTTTGATACTTGTT

BX-R_2_143+90 ATGGCATGGTTTTTTAAACAATTTATTTATTTGCTAATTGCTTCGGTTTTGATACTTGTT

BX-R_5_143+90 ATGGCATGGTTTTTTAAACAATTTATTTATTTGCTAATTGCTTCGGTTTTGATACTTGTT

*********************************** ************************

MG637361.1 ATATTAAAGGTAAATTGGTTTACTACAGAAGAAGGCTTTAGCGACTATGCCGTATTCACT 1140

BX-R_2.42 ATATTAAAG---------------------------------------------------

BX-R_2.52 ATATTAAAG---------------------------------------------------

BX-R_5.11 ATATTAAAG---------------------------------------------------

BX-R_5.9 ATATTAAAGGTAAATTGGTTTACTACAGAAGAAGGCTTTAGCGACTATGCCGTATTCACT

BX-R_5.8 ATATTAAAGGTAAATTGGTTTACTACAGAAGAAGGCTTTAGCGACTATGCCGTATTCACT

BX-R_2.12 ATATTAAAGGTAAATTGGTTTACTACAGAAGAAGGCTTTAGCGACTATGCCGTATTCACT

BX-R_1.40 ATATTAAAGGTAAATTGGTTTACTACAGAAGAAGGCTTTAGCGACTATGCCGTATTCACT

BX-R_1.6 ATATTAAAGGTAAATTGGTTTACTACAGAAGAAGGCTTTAGCGACTATGCCGTATTCACT

BX-R_1.5 ATATTAAAGGTAAATTGGTTTACTACAGAAGAAGGCTTTAGCGACTATGCCGTATTCACT

BX-R_2_143+90 ATATTAAAGGTAAATTGGTTTACTACAGAAGAAGGCTTTAGCGACTATGCCGTATTCACT

BX-R_5_143+90 ATATTAAAGGTAAATTGGTTTACTACAGAAGAAGGCTTTAGCGACTATGCCGTATTCACT

*********

MG637361.1 AATACACCTTGGACCGTCCTCTTCTTCTTCCTAACACTGTATCTTACGTGTACCATATTT 1200

BX-R_2.42 ------------------------------------------------------------

BX-R_2.52 ------------------------------------------------------------

BX-R_5.11 ------------------------------------------------------------

BX-R_5.9 AATACACCTTGGACCGTCCTCTTCTTCTTCCTAACACTGTATCTTACGTGTACCATATTT

BX-R_5.8 AATACACCTTGGACCGTCCTCTTCTTCTTCCTAACACTGTATCTTACGTGTACCATATTT

BX-R_2.12 AATACACCTTGGACCGTCCTCTTCTTCTTCCTAACACTGTATCTTACGTGTACCATATTT

BX-R_1.40 AATACACCTTGGACCGTCCTCTTCTTCTTCCTAACACTGTATCTTACGTGTACCATATTT

BX-R_1.6 AATACACCTTGGACCGTCCTCTTCTTCTTCCTAACACTGTATCTTACGTGTACCATATTT

BX-R_1.5 AATACACCTTGGACCGTCCTCTTCTTCTTCCTAACACTGTATCTTACGTGTACCATATTT

BX-R_2_143+90 AATACACCTTGGACCGTCCTCTTCTTCTTCCTAACACTGTATCTTACGTGTACCATATTT

BX-R_5_143+90 AATACACCTTGGACCGTCCTCTTCTTCTTCCTAACACTGTATCTTACGTGTACCATATTT

MG637361.1 TTCTGTTTCATGATAAGTGGTTTCTTTTCAAAAGCCAGTACAGCGGCGTTGTTTGGTGGG 1260

BX-R_2.42 ----------------------------------CCAGTACAGCGGCGTTGTTTGGTGGG

BX-R_2.52 ----------------------------------CCAGTACAGCGGCGTTGTTTGGTGGG

BX-R_5.11 ----------------------------------CCAGTACAGCGGCGTTGTTTGGTGGG

BX-R_5.9 TTCTGTTTCATGATAAGTGGTTTCTTTTCAAAAGCCAGTACAGCGGCGTTGTTTGGTGGG

BX-R_5.8 TTCTGTTTCATGATAAGTGGTTTCTTTTCAAAAGCCAGTACAGCGGCGTTGTTTGGTGGG

BX-R_2.12 TTCTGTTTCATGATAAGTGGTTTCTTTTCAAAAGCCAGTACAGCGGCGTTGTTTGGTGGG

BX-R_1.40 TTCTGTTTCATGATAAGTGGTTTCTTTTCAAAAGCCAGTACAGCGGCGTTGTTTGGTGGG

BX-R_1.6 TTCTGTTTCATGATAAGTGGTTTCTTTTCAAAAGCCAGTACAGCGGCGTTGTTTGGTGGG

BX-R_1.5 TTCTGTTTCATGATAAGTGGTTTCTTTTCAAAAGCCAGTACAGCGGCGTTGTTTGGTGGG

BX-R_2_143+90 TTCTGTTTCATGATAAGTGGTTTCTTTTCAAAAGCCAGTACAGCGGCGTTGTTTGGTGGG

BX-R_5_143+90 TTCTGTTTCATGATAAGTGGTTTCTTTTCAAAAGCCAGTACAGCGGCGTTGTTTGGTGGG

**************************

MG637361.1 GTGATCTGGTTTCTGACGTATATCCCCGCATTCCTCCTGGCTATGGACGTGAACATGTCT 1320

BX-R_2.42 GTGATCTGGTTTCTGACGTATATCCCCGCATTCCTCCTGGCTATGGACGTGAACATGTCT

BX-R_2.52 GTGATCTGGTTTCTGACGTATATCCCCGCATTCCTCCTGGCTATGGACGTGAACATGTCT

BX-R_5.11 GTGATCTGGTTTCTGACGTATATCCCCGCATTCCTCCTGGCTATGGACGTGAACATGTCT

BX-R_5.9 GTGATCTGGTTTCTGACGTATATCCCCGCATTCCTCCTGGCTATGGACGTGAACATGTCT

BX-R_5.8 GTGATCTGGTTTCTGACGTATATCCCCGCATTCCTCCTGGCTATGGACGTGAACATGTCT

BX-R_2.12 GTGATCTGGTTTCTGACGTATATCCCCGCATTCCTCCTGGCTATGGACGTGAACATGTCT

BX-R_1.40 GTGATCTGGTTTCTGACGTATATCCCCGCATTCCTCCTGGCTATGGACGTGAACATGTCT

BX-R_1.6 GTGATCTGGTTTCTGACGTATATCCCCGCATTCCTCCTGGCTATGGACGTGAACATGTCT

BX-R_1.5 GTGATCTGGTTTCTGACGTATATCCCCGCATTCCTCCTGGCTATGGACGTGAACATGTCT

BX-R_2_143+90 GTGATCTGGTTTCTGACGTATATCCCCGCATTCCTCCTGGCTATG---------------

BX-R_5_143+90 GTGATCTGGTTTCTGACGTATATCCCCGCATTCCTCCTGGCTATG---------------

*********************************************

I. 186+185

MG637361.1 GCAACGGACTCGTCACGATTCGTATTCGGTCACGTCGTTATAATGATGGCTTTGAACTGT 1500

BX-R_2.42 GCAACGGACTCGTCACGATTCGTATTCGGTCACGTCGTTATAATGATGGCTTTGAACTGT

BX-R_2.52 GCAACGGACTCGTCACGATTCGTATTCGGTCACGTCGTTATAATGATGGCTTTGAACTGT

BX-R_5.8 GCAACGGACTCGTCACGATTCGTATTCGGTCACGTCGTTATAATGATGGCTTTGAACTGT

BX-R_5.11 GCAACGGACTCGTCACGATTCGTATTCGGTCACGTCGTTATAATGATGGCTTTGAACTGT

BX-R_5.9 GCAACGGACTCGTCACGATTCGTATTCGGTCACGTCGTTATAATGATGGCTTTGAACTGT

BX-R_2.12 GCAACGGACTCGTCACGATTCGTATTCGGTCACGTCGTTATAATGATGGCTTTGAACTGT

BX-R_1.40 GCAACGGACTCGTCACGATTCGTATTCGGTCACGTCGTTATAATGATGGCTTTGAACTGT

BX-R_1.6 GCAACGGACTCGTCACGATTCGTATTCGGTCACGTCGTTATAATGATGGCTTTGAACTGT

BX-R_1.5 GCAACGGACTCGTCACGATTCGTATTCGGTCACGTCGTTATAATGATGGCTTTGAACTGT

BX-R_2_186+185 --------------------------CGGTCACGTCGTTATAATGATGGCTTTGAACTGT

BX-R_5_186+185 --------------------------CGGTCACGTCGTTATAATGATGGCTTTGAACTGT

**********************************

MG637361.1 GTGCTCTACATGTTGATTGCCCTATATCTAGAGCAAGTACTACCCGGGCCGTATGGCACA 1560

BX-R_2.42 GTGCTCTACATGTTGATTGCCCTATATCTAGAGCAAGTACTACCCGGGCCGTATGGCACA

BX-R_2.52 GTGCTCTACATGTTGATTGCCCTATATCTAGAGCAAGTACTACCCGGGCCGTATGGCACA

BX-R_5.8 GTGCTCTACATGTTGATTGCCCTATATCTAGAGCAAGTACTACCCGGGCCGTATGGCACA

BX-R_5.11 GTGCTCTACATGTTGATTGCCCTATATCTAGAGCAAGTACTACCCGGGCCGTATGGCACA

BX-R_5.9 GTGCTCTACATGTTGATTGCCCTATATCTAGAGCAAGTACTACCCGGGCCGTATGGCACA

BX-R_2.12 GTGCTCTACATGTTGATTGCCCTATATCTAGAGCAAGTACTACCCGGGCCGTATGGCACA

BX-R_1.40 GTGCTCTACATGTTGATTGCCCTATATCTAGAGCAAGTACTACCCGGGCCGTATGGCACA

BX-R_1.6 GTGCTCTACATGTTGATTGCCCTATATCTAGAGCAAGTACTACCCGGGCCGTATGGCACA

BX-R_1.5 GTGCTCTACATGTTGATTGCCCTATATCTAGAGCAAGTACTACCCGGGCCGTATGGCACA

BX-R_2_186+185 GTGCTCTACATGTTGATTGCCCTATATCTAGAGCAAGTACTACCCGGGCCGTATGGCACA

BX-R_5_186+185 GTGCTCTACATGTTGATTGCCCTATATCTAGAGCAAGTACTACCCGGGCCGTATGGCACA

************************************************************

MG637361.1 CCGAAGCCCTGGTATTTCTTCGTCCAAAGACAGTTCTGGTGTAGCAGCAAAACTACTCAT 1620

BX-R_2.42 CCGAAGCCCTGGTATTTCTTCGTCCAAAGACAGTTCTGGTGTAGCAGCAAAACTACTCAT

BX-R_2.52 CCGAAGCCCTGGTATTTCTTCGTCCAAAGACAGTTCTGGTGTAGCAGCAAAACTACTCAT

BX-R_5.8 CCGAAGCCCTGGTATTTCTTCGTCCAAAGACAGTTCTGGTGTAGCAGC-AAACTACTCAT

BX-R_5.11 CCGAAGCCCTGGTATTTCTTCGTCCAAAGACAGTTCTGGTGTAGCAGCAAAACTACTCAT

BX-R_5.9 CCGAAGCCCTGGTATTTCTTCGTCCAAAGACAGTTCTGGTGTAGCAGCAAAACTACTCAT

BX-R_2.12 CCGAAGCCCTGGTATTTCTTCGTCCAAAGACAGTTCTGGTGTAGCAGCAAAACTACTCAT

BX-R_1.40 CCGAAGCCCTGGTATTTCTTCGTCCAAAGACAGTTCTGGTGTAGCAGCAAAACTACTCAT

BX-R_1.6 CCGAAGCCCTGGTATTTCTTCGTCCAAAGACAGTTCTGGTGTAGCAGCAAAACTACTCAT

BX-R_1.5 CCGAAGCCCTGGTATTTCTTCGTCCAAAGACAGTTCTGGTGTAGCAGCAAAACTACTCAT

BX-R_2_186+185 CCGAAGCCCTGGTATTTCTTCGTCCAAAGACAGTTCTGGTGTAGCAGCAAAACTACTCAT

BX-R_5_186+185 CCGAAGCCCTGGTATTTCTTCGTCCAAAGACAGTTCTGGTGTAGCAGCAAAACTACTCAT

************************************************ ***********

MG637361.1 GATATCGGTACAGACAACAGCGACACATCAAGTTTAACAAAAGAAAGCGACCCTACAGAC 1680

BX-R_2.42 GAT--CGGTACAGACAACAGCGACACATCAAGTTTAACAAAAGAAAGCGACCCTACAGAC

BX-R_2.52 GAT--CGGTACAGACAACAGCGACACATCAAGTTTAACAAAAGAAAGCGACCCTACAGAC

BX-R_5.8 GATATCGGTACAGACAACAGCGACACATCAAGTTTAACAAAAGAAAGCGACCCTACAGAC

BX-R_5.11 GATATCGGTACAGACAACAGCGACACATCAAGTTTAACAAAAGAAAGCGACCCTACAGAC

BX-R_5.9 GATATCGGTACAGACAACAGCGACACATCAAGTTTAACAAAAGAAAGCGACCCTACAGAC

BX-R_2.12 GATATCGGTACAGACAACAGCGACACATCAAGTTTAACAAAAGAAAGCGACCCTACAGAC

BX-R_1.40 GATATCGGTACAGACAACAGCGACACATCAAGTTTAACAAAAGAAAGCGACCCTACAGAC

BX-R_1.6 GATATCGGTACAGACAACAGCGACACATCAAGTTTAACAAAAGAAAGCGACCCTACAGAC

BX-R_1.5 GATATCGGTACAGACAACAGCGACACATCAAGTTTAACAAAAGAAAGCGACCCTACAGAC

BX-R_2_186+185 GATATCGGTACAGACAACAGCGACACATCAAGTTTAACAAAAGAAAGCGACCCTACAGAC

BX-R_5_186+185 GATATCGGTACAGACAACAGCGACACATCAAGTTTAACAAAAGAAAGCGACCCTACAGAC

*** *******************************************************

MG637361.1 CTTCCGATTGGAGTTAAAATACAAAACCTTAAAAAGGTTTACGGGAGCAACGTTGCGGTA 1740

BX-R_2.42 CTTCCGATTGGAGTTAAAATACAAAACCTTAAAAAGGTTTACGGGAGCAACGTTGCGGTA

BX-R_2.52 CTTCCGATTGGAGTTAAAATACAAAACCTTAAAAAGGTTTACGGGAGCAACGTTGCGGTA

BX-R_5.8 CTTCCGATTGGAGTTAAAATACAAAACCTTAAAAAGGTTTACGGGAGCAACGTTGCGGTA

BX-R_5.11 CTTCCGATTGGAGTTAAAATACAAAACCTTAAAAAGGTTTACGGGAGCAACGTTGCGGTA

BX-R_5.9 CTTCCGATTGGAGTTAAAATACAAAACCTTAAAAAGGTTTACGGGAGCAACGTTGCGGTA

BX-R_2.12 CTTCCGATTGGAGTTAAAATACAAAACCTTAAAAAGGTTTACGGGAGCAACGTTGCGGTA

BX-R_1.40 CTTCCGATTGGAGTTAAAATACAAAACCTTAAAAAGGTTTACGGGAGCAACGTTGCGGTA

BX-R_1.6 CTTCCGATTGGAGTTAAAATACAAAACCTTAAAAAGGTTTACGGGAGCAACGTTGCGGTA

BX-R_1.5 CTTCCGATTGGAGTTAAAATACAAAACCTTAAAAAGGTTTACGGGAGCAACGTTGCGGTA

BX-R_2_186+185 CTT---------------------------------------------------------

BX-R_5_186+185 CTT---------------------------------------------------------

***
