# Supplementary material for: Shared and Independent Genetic Basis of Resistance to Bt Toxin Cry2Ab in Two Strains of Pink Bollworm
Source: Sci Rep. 2020 May 14;10:7988. doi: 10.1038/s41598-020-64811-w (PMC7224296; doi:10.1038/s41598-020-64811-w)
Supplement: Supplementary file 4 — Supplementary figure S3. [file 41598_2020_64811_MOESM4_ESM.docx]

MG637361.1 ATGCGGGCGCGTGGAGAGCGGAAGGAGGCGGGCTCATGGGTGAAGTTTAGGCTGTTGATG 60

A1.9 ATGCGGGCGCGTGGAGAGCGGAAGGAGGCGGGCTCATGGGTGAAGTTTAGGCTGTTGATG 60

N1.8 ATGCGGGCGCGTGGAGAGCGGAAGGAGGCGGGCTCATGGGTGAAGTTTAGGCTGTTGATG 60

A1.4 ATGCGGGCGCGTGGAGAGCGGAAGGAGGCGGGCTCATGGGTGAAGTTTAGGCTGTTGATG 60

A3.1 ATGCGGGCGCGTGGAGAGCGGAAGGAGGCGGGCTCATGGGTGAAGTTTAGGCTGTTGATG 60

A3.4 ATGCGGGCGCGTGGAGAGCGGAAGGAGGCGGGCTCATGGGTGAAGTTTAGGCTGTTGATG 60

A3.6 ATGCGGGCGCGTGGAGAGCGGAAGGAGGCGGGCTCATGGGTGAAGTTTAGGCTGTTGATG 60

A3.7 ATGCGGGCGCGTGGAGAGCGGAAGGAGGCGGGCTCATGGGTGAAGTTTAGGCTGTTGATG 60

A3.8 ATGCGGGCGCGTGGAGAGCGGAAGGAGGCGGGCTCATGGGTGAAGTTTAGGCTGTTGATG 60

N3.1 ATGCGGGCGCGTGGAGAGCGGAAGGAGGCGGGCTCATGGGTGAAGTTTAGGCTGTTGATG 60

N3.6 ATGCGGGCGCGTGGAGAGCGGAAGGAGGCGGGCTCATGGGTGAAGTTTAGGCTGTTGATG 60

N3.3 ATGCGGGCGCGTGGAGAGCGGAAGGAGGCGGGCTCATGGGTGAAGTTTAGGCTGTTGATG 60

N3.10 ATGCGGGCGCGTGGAGAGCGGAAGGAGGCGGGCTCATGGGTGAAGTTTAGGCTGTTGATG 60

A1.3 ATGCGGGCGCGTGGAGAGCGGAAGGAGGCGGGCTCATGGGTGAAGTTTAGGCTGTTGATG 60

N1.2 ATGCGGGCGCGTGGAGAGCGGAAGGAGGCGGGCTCATGGGTGAAGTTTAGGCTGTTGATG 60

N1.5 ATGCGGGCGCGTGGAGAGCGGAAGGAGGCGGGCTCATGGGTGAAGTTTAGGCTGTTGATG 60

N1.27 ATGCGGGCGCGTGGAGAGCGGAAGGAGGCGGGCTCATGGGTGAAGTTTAGGCTGTTGATG 60

A1.2 ATGCGGGCGCGTGGAGAGCGGAAGGAGGCGGGCTCATGGGTGAAGTTTAGGCTGTTGATG 60

A1.7 ATGCGGGCGCGTGGAGAGCGGAAGGAGGCGGGCTCATGGGTGAAGTTTAGGCTGTTGATG 60

N3.9 ATGCGGGCGCGTGGAGAGCGGAAGGAGGCGGGCTCATGGGTGAAGTTTAGGCTGTTGATG 60

************************************************************

MG637361.1 TGGAAGAACTTCGTGCAGCAGTTGAGGCACCCAGTGCAGACGGCGGCTGAGCTGCTGCTA 120

A1.9 TGGAAGAACTTCGTGCAGCAGTTGAGGCACCCAGTGCAGATGGCGGCTGAGCTGCTGCTA 120

N1.8 TGGAAGAACTTCGTGCAGCAGTTGAGGCACCCAGTGCAGACGGCGGCTGAGCTGCTGCTA 120

A1.4 TGGAAGAACTTCGTGCAGCAGTTGAGGCACCCAGTGCAGACGGCGGCTGAGCTGCTGCTA 120

A3.1 TGGAAGAACTTCGTGCAGCAGTTGAGGCACCCAGTGCAGACGGCGGCTGAGCTGCTGCTA 120

A3.4 TGGAAGAACTTCGTGCAGCAGTTGAGGCACCCAGTGCAGACGGCGGCTGAGCTGCTGCTA 120

A3.6 TGGAAGAACTTCGTGCAGCAGTTGAGGCACCCAGTGCAGACGGCGGCTGAGCTGCTGCTA 120

A3.7 TGGAAGAACTTCGTGCAGCAGTTGAGGCACCCAGTGCAGACGGCGGCTGAGCTGCTGCTA 120

A3.8 TGGAAGAACTTCGTGCAGCAGTTGAGGCACCCAGTGCAGACGGCGGCTGAGCTGCTGCTA 120

N3.1 TGGAAGAACTTCGTGCAGCAGTTGAGGCACCCAGTGCAGACGGCGGCTGAGCTGCTGCTA 120

N3.6 TGGAAGAACTTCGTGCAGCAGTTGAGGCACCCAGTGCAGACGGCGGCTGAGCTGCTGCTA 120

N3.3 TGGAAGAACTTCGTGCAGCAGTTGAGGCACCCAGTGCAGACGGCGGCTGAGCTGCTGCTA 120

N3.10 TGGAAGAACTTCGTGCAGCAGTTGAGGCACCCAGTGCAGACGGCGGCTGAGCTGCTGCTA 120

A1.3 TGGAAGAACTTCGTGCAGCAGTTGAGGCACCCAGTGCAGACGGCGGCTGAGCTGCTGCTA 120

N1.2 TGGAAGAACTTCGTGCAGCAGTTGAGGCACCCAGTGCAGACGGCGGCTGAGCTGCTGCTA 120

N1.5 TGGAAGAACTTCGTGCAGCAGTTGAGGCACCCAGTGCAGACGGCGGCTGAGCTGCTGCTA 120

N1.27 TGGAAGAACTTCGTGCAGCAGTTGAGGCACCCAGTGCAGACGGCGGCTGAGCTGCTGCTA 120

A1.2 TGGAAGAACTTCGTGCAGCAGTTGAGGCACCCAGTGCAGATGGCGGCTGAGCTGCTGCTA 120

A1.7 TGGAAGAACTTCGTGCAGCAGTTGAGGCACCCAGTGCAGATGGCGGCTGAGCTGCTGCTA 120

N3.9 TGGAAGAACTTCGTGCAGCAGTTGAGGCACCCAGTGCAGACGGCGGCTGAGCTGCTGCTA 120

**************************************** *******************

MG637361.1 CCAGTCCTAACCATGAGCCTGGTCCTGGTGCTACGGTCACAGATCGACCCCGAAGTCTTG 180

A1.9 CCAGTCCTAACCATGAGCCTGGTCCTGGTGCTACGGTCACAGATCGACCCCGAAGTCTTG 180

N1.8 CCAGTCCTAACCATGAGCCTGGTCCTGGTGCTACGGTCACAGATCGACCCCGAAGTCTTG 180

A1.4 CCAGTCCTAACCATGAGCCTGGTCCTGGTGCTACGGTCACAGATCGACCCCGAAGTCTTG 180

A3.1 CCAGTCCTAACCATGAGCCTGGTCCTGGTGCTACGGTCACAGATCGACCCCGAAGTCTTG 180

A3.4 CCAGTCCTAACCATGAGCCTGGTCCTGGTGCTACGGTCACAGATCGACCCCGAAGTCTTG 180

A3.6 CCAGTCCTAACCATGAGCCTGGTCCTGGTGCTACGGTCACAGATCGACCCCGAAGTCTTG 180

A3.7 CCAGTCCTAACCATGAGCCTGGTCCTGGTGCTACGGTCACAGATCGACCCCGAAGTCTTG 180

A3.8 CCAGTCCTAACCATGAGCCTGGTCCTGGTGCTACGGTCACAGATCGACCCCGAAGTCTTG 180

N3.1 CCAGTCCTAACCATGAGCCTGGTCCTGGTGCTACGGTCACAGATCGACCCCGAAGTCTTG 180

N3.6 CCAGTCCTAACCATGAGCCTGGTCCTGGTGCTACGGTCACAGATCGACCCCGAAGTCTTG 180

N3.3 CCAGTCCTAACCATGAGCCTGGTCCTGGTGCTACGGTCACAGATCGACCCCGAAGTCTTG 180

N3.10 CCAGTCCTAACCATGAGCCTGGTCCTGGTGCTACGGTCACAGATCGACCCCGAAGTCTTG 180

A1.3 CCAGTCCTAACCATGAGCCTGGTCCTGGTGCTACGGTCACAGATCGACCCCGAAGTCTTG 180

N1.2 CCAGTCCTAACCATGAGCCTGGTCCTGGTGCTACGGTCACAGATCGACCCCGAAGTCTTG 180

N1.5 CCAGTCCTAACCATGAGCCTGGTCCTGGTGCTACGGTCACAGATCGACCCCGAAGTCTTG 180

N1.27 CCAGTCCTAACCATGAGCCTGGTCCTGGTGCTACGGTCACAGATCGACCCCGAAGTCTTG 180

A1.2 CCAGTCCTAACCATGAGCCTGGTCCTGGTGCTACGGTCACAGATCGACCCCGAAGTCTTG 180

A1.7 CCAGTCCTAACCATGAGCCTGGTCCTGGTGCTACGGTCACAGATCGACCCCGAAGTCTTG 180

N3.9 CCAGTCCTAACCATGAGCCTGGTCCTGGTGCTACGGTCACAGATCGACCCCGAAGTCTTG 180

************************************************************

MG637361.1 GAAACCAGAACCTACCCGCCAATACCAGCCCACACTTTAAACTATTCCGTGACTGTTTTG 240

A1.9 GAAACCAGAACCTACCCGCCAATACCAGCCCACACTTTAAACTATTCCGTGACTGTTTTG 240

N1.8 GAAACCAGAACCTACCCGCCAATACCAGCCCACACTTTAAACTATTCCGTGACTGTTTTG 240

A1.4 GAAACCAGAACCTACCCGCCAATACCAGCCCACACTTTAAACTATTCCGTGACTGTTTTG 240

A3.1 GAAACCAGAACCTACCCGCCAATACCAGCCCACACTTTAAACTATTCCGTGACTGTTTTG 240

A3.4 GAAACCAGAACCTACCCGCCAATACCAGCCCACACTTTAAACTATTCCGTGACTGTTTTG 240

A3.6 GAAACCAGAACCTACCCGCCAATACCAGCCCACACTTTAAACTATTCCGTGACTGTTTTG 240

A3.7 GAAACCAGAACCTACCCGCCAATACCAGCCCACACTTTAAACTATTCCGTGACTGTTTTG 240

A3.8 GAAACCAGAACCTACCCGCCAATACCAGCCCACACTTTAAACTATTCCGTGACTGTTTTG 240

N3.1 GAAACCAGAACCTACCCGCCAATACCAGCCCACACTTTAAACTATTCCGTGACTGTTTTG 240

N3.6 GAAACCAGAACCTACCCGCCAATACCAGCCCACACTTTAAACTATTCCGTGACTGTTTTG 240

N3.3 GAAACCAGAACCTACCCGCCAATACCAGCCCACACTTTAAACTATTCCGTGACTGTTTTG 240

N3.10 GAAACCAGAACCTACCCGCCAATACCAGCCCACACTTTAAACTATTCCGTGACTGTTTTG 240

A1.3 GAAACCAGAACCTACCCGCCAATACCAGCCCACACTTTAAACTATTCCGTGACTGTTTTG 240

N1.2 GAAACCAGAACCTACCCGCCAATACCAGCCCACACTTTAAACTATTCCGTGACTGTTTTG 240

N1.5 GAAACCAGAACCTACCCGCCAATACCAGCCCACACTTTAAACTATTCCGTGACTGTTTTG 240

N1.27 GAAACCAGAACCTACCCGCCAATACCAGCCCACACTTTAAACTATTCCGTGACTGTTTTG 240

A1.2 GAAACCAGAACCTACCCGCCAATACCAGCCCACACTTTAAACTATTCCGTGACTGTTTTG 240

A1.7 GAAACCAGAACCTACCCGCCAATACCAGCCCACACTTTAAACTATTCCGTGACTGTTTTG 240

N3.9 GAAACCAGAACCTACCCGCCAATACCAGCCCACACTTTAAACTATTCCGTGACTGTTTTG 240

************************************************************

MG637361.1 GGCGGAATGAATTTAACAAGAATGTCCATGGCATTCTCACCCGAGAATGCCGTATTGAGG 300

A1.9 GGCGGAATGAATTTAACAAGAATGTCCATGGCATTCTCACCCGAGAATGCCGTATTGAGG 300

N1.8 GGCGGAATGAATTTAACAAGAATGTCCATGGCATTCTCACCCGAGAATGCCGTATTGAGG 300

A1.4 GGCGGAATGAATTTAACAAGAATGTCCATGGCATTCTCACCCGAGAATGCCGTATTGAGG 300

A3.1 GGCGGAATGAATTTAACAAGAATGTCCATGGCATTCTCACCCGAGAATGCCGTATTGAGG 300

A3.4 GGCGGAATGAATTTAACAAGAATGTCCATGGCATTCTCACCCGAGAATGCCGTATTGAGG 300

A3.6 GGCGGAATGAATTTAACAAGAATGTCCATGGCATTCTCACCCGAGAATGCCGTATTGAGG 300

A3.7 GGCGGAATGAATTTAACAAGAATGTCCATGGCATTCTCACCCGAGAATGCCGTATTGAGG 300

A3.8 GGCGGAATGAATTTAACAAGAATGTCCATGGCATTCTCACCCGAGAATGCCGTATTGAGG 300

N3.1 GGCGGAATGAATTTAACAAGAATGTCCATGGCATTCTCACCCGAGAATGCCGTATTGAGG 300

N3.6 GGCGGAATGAATTTAACAAGAATGTCCATGGCATTCTCACCCGAGAATGCCGTATTGAGG 300

N3.3 GGCGGAATGAATTTAACAAGAATGTCCATGGCATTCTCACCCGAGAATGCCGTATTGAGG 300

N3.10 GGCGGAATGAATTTAACAAGAATGTCCATGGCATTCTCACCCGAGAATGCCGTATTGAGG 300

A1.3 GGCGGAATGAATTTAACAAGAATGTCCATGGCATTCTCACCCGAGAATGCCGTATTGAGG 300

N1.2 GGCGGAATGAATTTAACAAGAATGTCCATGGCATTCTCACCCGAGAATGCCGTATTGAGG 300

N1.5 GGCGGAATGAATTTAACAAGAATGTCCATGGCATTCTCACCCGAGAATGCCGTATTGAGG 300

N1.27 GGCGGAATGAATTTAACAAGAATGTCCATGGCATTCTCACCCGAGAATGCCGTATTGAGG 300

A1.2 GGCGGAATGAATTTAACAAGAATGTCCATGGCATTCTCACCCGAGAATGCCGTATTGAGG 300

A1.7 GGCGGAATGAATTTAACAAGAATGTCCATGGCATTCTCACCCGAGAATGCCGTATTGAGG 300

N3.9 GGCGGAATGAATTTAACAAGAATGTCCATGGCATTCTCACCCGAGAATGCCGTATTGAGG 300

************************************************************

MG637361.1 GACGTCGTATCCAGTGCTACAACAAAGTTACTGCTTAAAAACATGAGAGACCAAGTACTG 360

A1.9 GACGTCGTATCCAGTGCTACAACAAAGTTACTGCTTAAAAACATGAGAGACCAAGTACTG 360

N1.8 GACGTCGTATCCAGTGCTACAACAAAGTTACTGCTTAAAAACATGAGAGACCAAGTACTG 360

A1.4 GACGTCGTATCCAGTGCTACAACAAAGTTACTGCTTAAAAACATGAGAGACCAAGTACTG 360

A3.1 GACGTCGTATCCAGTGCTACAACAAAGTTACTGCTTAAAAACATGAGAGACCAAGTACTG 360

A3.4 GACGTCGTATCCAGTGCTACAACAAAGTTACTGCTTAAAAACATGAGAGACCAAGTACTG 360

A3.6 GACGTCGTATCCAGTGCTACAACAAAGTTACTGCTTAAAAACATGAGAGACCAAGTACTG 360

A3.7 GACGTCGTATCCAGTGCTACAACAAAGTTACTGCTTAAAAACATGAGAGACCAAGTACTG 360

A3.8 GACGTCGTATCCAGTGCTACAACAAAGTTACTGCTTAAAAACATGAGAGACCAAGTACTG 360

N3.1 GACGTCGTATCCAGTGCTACAACAAAGTTACTGCTTAAAAACATGAGAGACCAAGTACTG 360

N3.6 GACGTCGTATCCAGTGCTACAACAAAGTTACTGCTTAAAAACATGAGAGACCAAGTACTG 360

N3.3 GACGTCGTATCCAGTGCTACAACAAAGTTACTGCTTAAAAACATGAGAGACCAAGTACTG 360

N3.10 GACGTCGTATCCAGTGCTACAACAAAGTTACTGCTTAAAAACATGAGAGACCAAGTACTG 360

A1.3 GACGTCGTATCCAGTGCTACAACAAAGTTACTGCTTAAAAACATGAGAGACCAAGTACTG 360

N1.2 GACGTCGTATCCAGTGCTACAACAAAGTTACTGCTTAAAAACATGAGAGACCAAGTACTG 360

N1.5 GACGTCGTATCCAGTGCTACAACAAAGTTACTGCTTAAAAACATGAGAGACCAAGTACTG 360

N1.27 GACGTCGTATCCAGTGCTACAACAAAGTTACTGCTTAAAAACATGAGAGACCAAGTACTG 360

A1.2 GACGTCGTATCCAGTGCTACAACAAAGTTACTGCTTAAAAACATGAGAGACCAAGTACTG 360

A1.7 GACGTCGTATCCAGTGCTACAACAAAGTTACTGCTTAAAAACATGAGAGACCAAGTACTG 360

N3.9 GACGTCGTATCCAGTGCTACAACAAAGTTACTGCTTAAAAACATGAGAGACCAAGTACTG 360

************************************************************

MG637361.1 CCCATCATTGAGGCATTGCCAATAGAAATACCGCCGGGACTGGTAAACTCGTCACAGGTG 420

A1.9 CCCATCATTGAGGCATTGCCAATAGAAATACCGCCGGGACTGGTAAACTCGTCACAGGTG 420

N1.8 CCCATCATTGAGGCATTGCCAATAGAAATACCGCCGGGACTGGTAAACTCGTCACAGGTG 420

A1.4 CCCATCATTGAGGCATTGCCAATAGAAATACCGCCGGGACTGGTAAACTCGTCACAGGTG 420

A3.1 CCCATCATTGAGGCATTGCCAATAGAAATACCGCCGGGACTGGTAAACTCGTCACAGGTG 420

A3.4 CCCATCATTGAGGCATTGCCAATAGAAATACCGCCGGGACTGGTAAACTCGTCACAGGTG 420

A3.6 CCCATCATTGAGGCATTGCCAATAGAAATACCGCCGGGACTGGTAAACTCGTCACAGGTG 420

A3.7 CCCATCATTGAGGCATTGCCAATAGAAATACCGCCGGGACTGGTAAACTCGTCACAGGTG 420

A3.8 CCCATCATTGAGGCATTGCCAATAGAAATACCGCCGGGACTGGTAAACTCGTCACAGGTG 420

N3.1 CCCATCATTGAGGCATTGCCAATAGAAATACCGCCGGGACTGGTAAACTCGTCACAGGTG 420

N3.6 CCCATCATTGAGGCATTGCCAATAGAAATACCGCCGGGACTGGTAAACTCGTCACAGGTG 420

N3.3 CCCATCATTGAGGCATTGCCAATAGAAATACCGCCGGGACTGGTAAACTCGTCACAGGTG 420

N3.10 CCCATCATTGAGGCATTGCCAATAGAAATACCGCCGGGACTGGTAAACTCGTCACAGGTG 420

A1.3 CCCATCATTGAGGCATTGCCAATAGAAATACCGCCGGGACTGGTAAACTCGTCACAGGTG 420

N1.2 CCCATCATTGAGGCATTGCCAATAGAAATACCGCCGGGACTGGTAAACTCGTCACAGGTG 420

N1.5 CCCATCATTGAGGCATTGCCAATAGAAATACCGCCGGGACTGGTAAACTCGTCACAGGTG 420

N1.27 CCCATCATTGAGGCATTGCCAATAGAAATACCGCCGGGACTGGTAAACTCGTCACAGGTG 420

A1.2 CCCATCATTGAGGCATTGCCAATAGAAATACCGCCGGGACTGGTAAACTCGTCACAGGTG 420

A1.7 CCCATCATTGAGGCATTGCCAATAGAAATACCGCCGGGACTGGTAAACTCGTCACAGGTG 420

N3.9 CCCATCATTGAGGCATTGCCAATAGAAATACCGCCGGGACTGGTAAACTCGTCACAGGTG 420

************************************************************

MG637361.1 TACGAAATAGTT-AAATTATTTGTCGACGAGAACGTTGTTACCGGATACAATAGCAGTGC 479

A1.9 TACGAAATAGTT-AAATTATTTGTCGACGAGAACGTTGTTACCGGATACAATAGCAGTGC 479

N1.8 TACGAAATAGTTTAAATTATTTGTCGACGAGAACGTTGTTACCGGATACAATAGCAGTGC 480

A1.4 TACGAAATAGTT-AAATTATTTGTCGACGAGAACGTTGTTACCGGATACAATAGCAGTGC 479

A3.1 TACGAAATAGTT-AAATTATTTGTCGACGAGAACGTTGTTACCGGATACAATAGCAGTGC 479

A3.4 TACGAAATAGTT-AAATTATTTGTCGACGAGAACGTTGTTACCGGATACAATAGCAGTGC 479

A3.6 TACGAAATAGTT-AAATTATTTGTCGACGAGAACGTTGTTACCGGATACAATAGCAGTGC 479

A3.7 TACGAAATAGTT-AAATTATTTGTCGACGAGAACGTTGTTACCGGATACAATAGCAGTGC 479

A3.8 TACGAAATAGTT-AAATTATTTGTCGACGAGAACGTTGTTACCGGATACAATAGCAGTGC 479

N3.1 TACGAAATAGTT-AAATTATTTGTCGACGAGAACGTTGTTACCGGATACAATAGCAGTGC 479

N3.6 TACGAAATAGTT-AAATTATTTGTCGACGAGAACGTTGTTACCGGATACAATAGCAGTGC 479

N3.3 TACGAAATAGTT-AAATTATTTGTCGACGAGAACGTTGTTACCGGATACAATAGCAGTGC 479

N3.10 TACGAAATAGTT-AAATTATTTGTCGACGAGAACGTTGTTACCGGATACAATAGCAGTGC 479

A1.3 TACGAAATAGTT-AAATTATTTGTCGACGAGAACGTTGTTACCGGATACAATAGCAGTGC 479

N1.2 TACGAAATAGTT-AAATTATTTGTCGACGAGAACGTTGTTACCGGATACAATAGCAGTGC 479

N1.5 TACGAAATAGTT-AAATTATTTGTCGACGAGAACGTTGTTACCGGATACAATAGCAGTGC 479

N1.27 TACGAAATAGTT-AAATTATTTGTCGACGAGAACGTTGTTACCGGATACAATAGCAGTGC 479

A1.2 TACGAAATAGTT-AAATTATTTGTCGACGAGAACGTTGTTACCGGATACAATAGCAGTGC 479

A1.7 TACGAAATAGTT-AAATTATTTGTCGACGAGAACGTTGTTACCGGATACAATAGCAGTGC 479

N3.9 TACGAAATAGTT-AAATTATTTGTCGACGAGAACGTTGTTACCGGATACAATAGCAGTGC 479

************ ***********************************************

MG637361.1 GGCAATGAGAGGAATATACGCAGAGGAAGAAGCCACGAGAAGGGTGATAGCTGGCATAGA 539

A1.9 GGCAATGAGAGGAATATACGCAGAGGAAGAAGCCACGAGAAGGGTGATAGCTGGCATAGA 539

N1.8 GGCAATGAGAGGAATATACGCAGAGGAAGAAGCCACGAGAAGGGTGATAGCTGGCATAGA 540

A1.4 GGCAATGAGAGGAATATACGCAGAGGAAGAAGCCACGAGAAGGGTGATAGCTGGCATAGA 539

A3.1 GGCAATGAGAGGAATATACGCAGAGGAAGAAGCCACGAGAAGGGTGATAGCTGGCATAGA 539

A3.4 GGCAATGAGAGGAATATACGCAGAGGAAGAAGCCACGAGAAGGGTGATAGCTGGCATAGA 539

A3.6 GGCAATGAGAGGAATATACGCAGAGGAAGAAGCCACGAGAAGGGTGATAGCTGGCATAGA 539

A3.7 GGCAATGAGAGGAATATACGCAGAGGAAGAAGCCACGAGAAGGGTGATAGCTGGCATAGA 539

A3.8 GGCAATGAGAGGAATATACGCAGAGGAAGAAGCCACGAGAAGGGTGATAGCTGGCATAGA 539

N3.1 GGCAATGAGAGGAATATACGCAGAGGAAGAAGCCACGAGAAGGGTGATAGCTGGCATAGA 539

N3.6 GGCAATGAGAGGAATATACGCAGAGGAAGAAGCCACGAGAAGGGTGATAGCTGGCATAGA 539

N3.3 GGCAATGAGAGGAATATACGCAGAGGAAGAAGCCACGAGAAGGGTGATAGCTGGCATAGA 539

N3.10 GGCAATGAGAGGAATATACGCAGAGGAAGAAGCCACGAGAAGGGTGATAGCTGGCATAGA 539

A1.3 GGCAATGAGAGGAATATACGCAGAGGAAGAAGCCACGAGAAGGGTGATAGCTGGCATAGA 539

N1.2 GGCAATGAGAGGAATATACGCAGAGGAAGAAGCCACGAGAAGGGTGATAGCTGGCATAGA 539

N1.5 GGCAATGAGAGGAATATACGCAGAGGAAGAAGCCACGAGAAGGGTGATAGCTGGCATAGA 539

N1.27 GGCAATGAGAGGAATATACGCAGAGGAAGAAGCCACGAGAAGGGTGATAGCTGGCATAGA 539

A1.2 GGCAATGAGAGGAATATACGCAGAGGAAGAAGCCACGAGAAGGGTGATAGCTGGCATAGA 539

A1.7 GGCAATGAGAGGAATATACGCAGAGGAAGAAGCCACGAGAAGGGTGATAGCTGGCATAGA 539

N3.9 GGCAATGAGAGGAATATACGCAGAGGAAGAAGCCACGAGAAGGGTGATAGCTGGCATAGA 539

************************************************************

MG637361.1 ATTCGATGACTCATTGCGTGAAATAACGGAGCTACCACTAGACTTGTCGTATGCGCTTCG 599

A1.9 ATTCGATGACTCATTGCGTGAAATAACGGAGCTACCACTAGACTTGTCGTATGCGCTTCG 599

N1.8 ATTCGATGACTCATTGCGTGAAATAACGGAGCTACCACTAGACTTGTCGTATGCGCTTCG 600

A1.4 ATTCGATGACTCATTGCGTGAAATAACGGAGCTACCACTAGACTTGTCGTATGCGCTTCG 599

A3.1 ATTCGATGACTCATTGCGTGAAATAACGGAGCTACCACTAGACTTGTCGTATGCGCTTCG 599

A3.4 ATTCGATGACTCATTGCGTGAAATAACGGAGCTACCACTAGACTTGTCGTATGCGCTTCG 599

A3.6 ATTCGATGACTCATTGCGTGAAATAACGGAGCTACCACTAGACTTGTCGTATGCGCTTCG 599

A3.7 ATTCGATGACTCATTGCGTGAAATAACGGAGCTACCACTAGACTTGTCGTATGCGCTTCG 599

A3.8 ATTCGATGACTCATTGCGTGAAATAACGGAGCTACCACTAGACTTGTCGTATGCGCTTCG 599

N3.1 ATTCGATGACTCATTGCGTGAAATAACGGAGCTACCACTAGACTTGTCGTATGCGCTTCG 599

N3.6 ATTCGATGACTCATTGCGTGAAATAACGGAGCTACCACTAGACTTGTCGTATGCGCTTCG 599

N3.3 ATTCGATGACTCATTGCGTGAAATAACGGAGCTACCACTAGACTTGTCGTATGCGCTTCG 599

N3.10 ATTCGATGACTCATTGCGTGAAATAACGGAGCTACCACTAGACTTGTCGTATGCGCTTCG 599

A1.3 ATTCGATGACTCATTGCGTGAAATAACGGAGCTACCACTAGACTTGTCGTATGCGCTTCG 599

N1.2 ATTCGATGACTCATTGCGTGAAATAACGGAGCTACCACTAGACTTGTCGTATGCGCTTCG 599

N1.5 ATTCGATGACTCATTGCGTGAAATAACGGAGCTACCACTAGACTTGTCGTATGCGCTTCG 599

N1.27 ATTCGATGACTCATTGCGTGAAATAACGGAGCTACCACTAGACTTGTCGTATGCGCTTCG 599

A1.2 ATTCGATGACTCATTGCGTGAAATAACGGAGCTACCACTAGACTTGTCGTATGCGCTTCG 599

A1.7 ATTCGATGACTCATTGCGTGAAATAACGGAGCTACCACTAGACTTGTCGTATGCGCTTCG 599

N3.9 ATTCGATGACTCATTGCGTGAAATAACGGAGCTACCACTAGACTTGTCGTATGCGCTTCG 599

************************************************************

MG637361.1 TTTTCCGGAGAGACCTCGCTTGAATTCCTTCTTCATGACAGGCGGTCGGACTTGGCGCAC 659

A1.9 TTTTCCGGAGAGACCTCGCTTGAATTCCTTCTTCATGACAGGCGGTCGGACTTGGCGCAC 659

N1.8 TTTTCCGGAGAGACCTCGCTTGAATTCCTTCTTCATGACAGGCGGTCGGACTTGGCGCAC 660

A1.4 TTTTCCGGAGAGACCTCGCTTGAATTCCTTCTTCATGACAGGCGGTCGGACTTGGCGCAC 659

A3.1 TTTTCCGGAGAGACCTCGCTTGAATTCCTTCTTCATGACAGGCGGTCGGACTTGGCGCAC 659

A3.4 TTTTCCGGAGAGACCTCGCTTGAATTCCTTCTTCATGACAGGCGGTCGGACTTGGCGCAC 659

A3.6 TTTTCCGGAGAGACCTCGCTTGAATTCCTTCTTCATGACAGGCGGTCGGACTTGGCGCAC 659

A3.7 TTTTCCGGAGAGACCTCGCTTGAATTCCTTCTTCATGACAGGCGGTCGGACTTGGCGCAC 659

A3.8 TTTTCCGGAGAGACCTCGCTTGAATTCCTTCTTCATGACAGGCGGTCGGACTTGGCGCAC 659

N3.1 TTTTCCGGAGAGACCTCGCTTGAATTCCTTCTTCATGACAGGCGGTCGGACTTGGCGCAC 659

N3.6 TTTTCCGGAGAGACCTCGCTTGAATTCCTTCTTCATGACAGGCGGTCGGACTTGGCGCAC 659

N3.3 TTTTCCGGAGAGACCTCGCTTGAATTCCTTCTTCATGACAGGCGGTCGGACTTGGCGCAC 659

N3.10 TTTTCCGGAGAGACCTCGCTTGAATTCCTTCTTCATGACAGGCGGTCGGACTTGGCGCAC 659

A1.3 TTTTCCGGAGAGACCTCGCTTGAATTCCTTCTTCATGACAGGCGGTCGGACTTGGCGCAC 659

N1.2 TTTTCCGGAGAGACCTCGCTTGAATTCCTTCTTCATGACAGGCGGTCGGACTTGGCGCAC 659

N1.5 TTTTCCGGAGAGACCTCGCTTGAATTCCTTCTTCATGACAGGCGGTCGGACTTGGCGCAC 659

N1.27 TTTTCCGGAGAGACCTCGCTTGAATTCCTTCTTCATGACAGGCGGTCGGACTTGGCGCAC 659

A1.2 TTTTCCGGAGAGACCTCGCTTGAATTCCTTCTTCATGACAGGCGGTCGGACTTGGCGCAC 659

A1.7 TTTTCCGGAGAGACCTCGCTTGAATTCCTTCTTCATGACAGGCGGTCGGACTTGGCGCAC 659

N3.9 TTTTCCGGAGAGACCTCGCTTGAATTCCTTCTTCATGACAGGCGGTCGGACTTGGCGCAC 659

************************************************************

MG637361.1 AGATAACGTGTTTCCTATGTTCGAAGTTCCCGGACCTCGCTTTCCGTATTCATGGGAAGG 719

A1.9 AGATAACGTGTTTCCTATGTTCGAAGTTCCCGGACCTCGCTTTCCGTATTCATGGGAAGG 719

N1.8 AGATAACGTGTTTCCTATGTTCGAAGTTCCCGGACCTCGCTTTCCGTATTCATGGGAAGG 720

A1.4 AGATAACGTGTTTCCTATGTTCGAAGTTCCCGGACCTCGCTTTCCGTATTCATGGGAAGG 719

A3.1 AGATAACGTGTTTCCTATGTTCGAAGTTCCCGGACCTCGCTTTCCGTATTCATGGGAAGG 719

A3.4 AGATAACGTGTTTCCTATGTTCGAAGTTCCCGGACCTCGCTTTCCGTATTCATGGGAAGG 719

A3.6 AGATAACGTGTTTCCTATGTTCGAAGTTCCCGGACCTCGCTTTCCGTATTCATGGGAAGG 719

A3.7 AGATAACGTGTTTCCTATGTTCGAAGTTCCCGGACCTCGCTTTCCGTATTCATGGGAAGG 719

A3.8 AGATAACGTGTTTCCTATGTTCGAAGTTCCCGGACCTCGCTTTCCGTATTCATGGGAAGG 719

N3.1 AGATAACGTGTTTCCTATGTTCGAAGTTCCCGGACCTCGCTTTCCGTATTCATGGGAAGG 719

N3.6 AGATAACGTGTTTCCTATGTTCGAAGTTCCCGGACCTCGCTTTCCGTATTCATGGGAAGG 719

N3.3 AGATAACGTGTTTCCTATGTTCGAAGTTCCCGGACCTCGCTTTCCGTATTCATGGGAAGG 719

N3.10 AGATAACGTGTTTCCTATGTTCGAAGTTCCCGGACCTCGCTTTCCGTATTCATGGGAAGG 719

A1.3 AGATAACGTGTTTCCTATGTTCGAAGTTCCCGGACCTCGCTTTCCGTATTCATGGGAAGG 719

N1.2 AGATAACGTGTTTCCTATGTTCGAAGTTCCCGGACCTCGCTTTCCGTATTCATGGGAAGG 719

N1.5 AGATAACGTGTTTCCTATGTTCGAAGTTCCCGGACCTCGCTTTCCGTATTCATGGGAAGG 719

N1.27 AGATAACGTGTTTCCTATGTTCGAAGTTCCCGGACCTCGCTTTCCGTATTCATGGGAAGG 719

A1.2 AGATAACGTGTTTCCTATGTTCGAAGTTCCCGGACCTCGCTTTCCGTATTCATGGGAAGG 719

A1.7 AGATAACGTGTTTCCTATGTTCGAAGTTCCCGGACCTCGCTTTCCGTATTCATGGGAAGG 719

N3.9 AGATAACGTGTTTCCTATGTTCGAAGTTCCCGGACCTCGCTTTCCGTATTCATGGGAAGG 719

************************************************************

MG637361.1 TGGAAATGATCCAGGATACGTAAACGAGATGTTCATAGCCTTGCAGCACATGATATCTTC 779

A1.9 TGGAAATGATCCAGGATACGTAAACGAGATGTTCATAGCCTTGCAGCACATGATATCTTC 779

N1.8 TGGAAATGATCCAGGATACGTAAACGAGATGTTCATAGCCTTGCAGCACATGATATCTTC 780

A1.4 TGGAAATGATCCAGGATACGTAAACGAGATGTTCATAGCCTTGCAGCACATGATATCTTC 779

A3.1 TGGAAATGATCCAGGATACGTAAACGAGATGTTCATAGCCTTGCAGCACATGATATCTTC 779

A3.4 TGGAAATGATCCAGGATACGTAAACGAGATGTTCATAGCCTTGCAGCACATGATATCTTC 779

A3.6 TGGAAATGATCCAGGATACGTAAACGAGATGTTCATAGCCTTGCAGCACATGATATCTTC 779

A3.7 TGGAAATGATCCAGGATACGTAAACGAGATGTTCATAGCCTTGCAGCACATGATATCTTC 779

A3.8 TGGAAATGATCCAGGATACGTAAACGAGATGTTCATAGCCTTGCAGCACATGATATCTTC 779

N3.1 TGGAAATGATCCAGGATACGTAAACGAGATGTTCATAGCCTTGCAGCACATGATATCTTC 779

N3.6 TGGAAATGATCCAGGATACGTAAACGAGATGTTCATAGCCTTGCAGCACATGATATCTTC 779

N3.3 TGGAAATGATCCAGGATACGTAAACGAGATGTTCATAGCCTTGCAGCACATGATATCTTC 779

N3.10 TGGAAATGATCCAGGATACGTAAACGAGATGTTCATAGCCTTGCAGCACATGATATCTTC 779

A1.3 TGGAAATGTTCCAGGATACGTAAACGAGATGTTCATAGCCTTGCAGCACATGATATCTTC 779

N1.2 TGGAAATGATCCAGGATACGTAAACGAGATGTTCATAGCCTTGCAGCACATGATATCTTC 779

N1.5 TGGAAATGATCCAGGATACGTAAACGAGATGTTCATAGCCTTGCAGCACATGATATCTTC 779

N1.27 TGGAAATGATCCAGGATACGTAAACGAGATGTTCATAGCCTTGCAGCACATGATATCTTC 779

A1.2 TGGAAATGATCCAGGATACGTAAACGAGATGTTCATAGCCTTGCAGCACATGATATCTTC 779

A1.7 TGGAAATGATCCAGGATACGTAAACGAGATGTTCATAGCCTTGCAGCACATGATATCTTC 779

N3.9 TGGAAATGATCCAGGATACGTAAACGAGATGTTCATAGCCTTGCAGCACATGATATCTTC 779

******** ***************************************************

MG637361.1 AGAACTGGTATCTAAAGTGGCGGGAGTGAACCTAGACTTCGATGTGCACATACAGAGGTA 839

A1.9 AGAACTGGTATCTAAAGTGGCGGGAGTGAACCTAGACTTCGATGTGCACATACAGAGGTA 839

N1.8 AGAACTGGTATCTAAAGTGGCGGGAGTGAACCTAGACTTCGATGTGCACATACAGAGGTA 840

A1.4 AGAACTGGTATCTAAAGTGGCGGGAGTGAACCTAGACTTCGATGTGCACATACAGAGGTA 839

A3.1 AGAACTGGTATCTAAAGTGGCGGGAGTGAACCTAGACTTCGATGTGCACATACAGAGGTA 839

A3.4 AGAACTGGTATCTAAAGTGGCGGGAGTGAACCTAGACTTCGATGTGCACATACAGAGGTA 839

A3.6 AGAACTGGTATCTAAAGTGGCGGGAGTGAACCTAGACTTCGATGTGCACATACAGAGGTA 839

A3.7 AGAACTGGTATCTAAAGTGGCGGGAGTGAACCTAGACTTCGATGTGCACATACAGAGGTA 839

A3.8 AGAACTGGTATCTAAAGTGGCGGGAGTGAACCTAGACTTCGATGTGCACATACAGAGGTA 839

N3.1 AGAACTGGTATCTAAAGTGGCGGGAGTGAACCTAGACTTCGATGTGCACATACAGAGGTA 839

N3.6 AGAACTGGTATCTAAAGTGGCGGGAGTGAACCTAGACTTCGATGTGCACATACAGAGGTA 839

N3.3 AGAACTGGTATCTAAAGTGGCGGGAGTGAACCTAGACTTCGATGTGCACATACAGAGGTA 839

N3.10 AGAACTGGTATCTAAAGTGGCGGGAGTGAACCTAGACTTCGATGTGCACATACAGAGGTA 839

A1.3 AGAACTGGTATCTAAAGTGGCGGGAGTGAACCTAGACTTCGATGTGCACATACAGAGGTA 839

N1.2 AGAACTGGTATCTAAAGTGGCGGGAGTGAACCTAGACTTCGATGTGCACATACAGAGGTA 839

N1.5 AGAACTGGTATCTAAAGTGGCGGGAGTGAACCTAGACTTCGATGTGCACATACAGAGGTA 839

N1.27 AGAACTGGTATCTAAAGTGGCGGGAGTGAACCTAGACTTCGATGTGCACATACAGAGGTA 839

A1.2 AGAACTGGTATCTAAAGTGGCGGGAGTGAACCTAGACTTCGATGTGCACATACAGAGGTA 839

A1.7 AGAACTGGTATCTAAAGTGGCGGGAGTGAACCTAGACTTCGATGTGCACATACAGAGGTA 839

N3.9 AGAACTGGTATCTAAAGTGGCGGGAGTGAACCTAGACTTCGATGTGCACATACAGAGGTA 839

************************************************************

MG637361.1 CCCACATCCAGCATACATCATGGACTTGGCGAAGGAAGCCCTGCAGTTCCTCTTCCCATC 899

A1.9 CCCACATCCAGCATACATCATGGACTTGGCGAAGGAAGCCCTGCAGTTCCTCTTCCCATC 899

N1.8 CCCACATCCAGCATACATCATGGACTTGGCGAAGGAAGCCCTGCAGTTCCTCTTCCCATC 900

A1.4 CCCACATCCAGCATACATCATGGACTTGGCGAAGGAAGCCCTGCAGTTCCTCTTCCCATC 899

A3.1 CCCACATCCAGCATACATCATGGACTTGGCGAAGGAAGCCCTGCAGTTCCTCTTCCCATC 899

A3.4 CCCACATCCAGCATACATCATGGACTTGGCGAAGGAAGCCCTGCAGTTCCTCTTCCCATC 899

A3.6 CCCACATCCAGCATACATCATGGACTTGGCGAAGGAAGCCCTGCAGTTCCTCTTCCCATC 899

A3.7 CCCACATCCAGCATACATCATGGACTTGGCGAAGGAAGCCCTGCAGTTCCTCTTCCCATC 899

A3.8 CCCACATCCAGCATACATCATGGACTTGGCGAAGGAAGCCCTGCAGTTCCTCTTCCCATC 899

N3.1 CCCACATCCAGCATACATCATGGACTTGGCGAAGGAAGCCCTGCAGTTCCTCTTCCCATC 899

N3.6 CCCACATCCAGCATACATCATGGACTTGGCGAAGGAAGCCCTGCAGTTCCTCTTCCCATC 899

N3.3 CCCACATCCAGCATACATCATGGACTTGGCGAAGGAAGCCCTGCAGTTCCTCTTCCCATC 899

N3.10 CCCACATCCAGCATACATCATGGACTTGGCGAAGGAAGCCCTGCAGTTCCTCTTCCCATC 899

A1.3 CCCACATCCAGCATACATCATGGACTTGGCGAAGGAAGCCCTGCAGTTCCTCTTCCCATC 899

N1.2 CCCACATCCAGCATACATCATGGACTTGGCGAAGGAAGCCCTGCAGTTCCTCTTCCCATC 899

N1.5 CCCACATCCAGCATACATCATGGACTTGGCGAAGGAAGCCCTGCAGTTCCTCTTCCCATC 899

N1.27 CCCACATCCAGCATACATCATGGACTTGGCGAAGGAAGCCCTGCAGTTCCTCTTCCCATC 899

A1.2 CCCACATCCAGCATACATCATGGACTTGGCGAAGGAAGCCCTGCAGTTCCTCTTCCCATC 899

A1.7 CCCACATCCAGCATACATCATGGACTTGGCGAAGGAAGCCCTGCAGTTCCTCTTCCCATC 899

N3.9 CCCACATCCAGCATACATCATGGACTTGGCGAAGGAAGCCCTGCAGTTCCTCTTCCCATC 899

************************************************************

MG637361.1 ATTCATCATGATCAGCTTCAGTTACACCGCTATCAATATTATACGATCCGTGACCGTGGA 959

A1.9 ATTCATCATGATCAGCTTCAGTTACACCGCTATCAATATTATACGATCCGTGACCGTGGA 959

N1.8 ATTCATCATGATCAGCTTCAGTTACACCGCTATCAATATTATACGATCCGTGACCGTGGA 960

A1.4 ATTCATCATGATCAGCTTCAGTTACACCGCTATCAATATTATACGATCCGTGACCGTGGA 959

A3.1 ATTCATCATGATCAGCTTCAGTTACACCGCTATCAATATTATACGATCCGTGACCGTGGA 959

A3.4 ATTCATCATGATCAGCTTCAGTTACACCGCTATCAATATTATACGATCCGTGACCGTGGA 959

A3.6 ATTCATCATGATCAGCTTCAGTTACACCGCTATCAATATTATACGATCCGTGACCGTGGA 959

A3.7 ATTCATCATGATCAGCTTCAGTTACACCGCTATCAATATTATACGATCCGTGACCGTGGA 959

A3.8 ATTCATCATGATCAGCTTCAGTTACACCGCTATCAATATTATACGATCCGTGACCGTGGA 959

N3.1 ATTCATCATGATCAGCTTCAGTTACACCGCTATCAATATTATACGATCCGTGACCGTGGA 959

N3.6 ATTCATCATGATCAGCTTCAGTTACACCGCTATCAATATTATACGATCCGTGACCGTGGA 959

N3.3 ATTCATCATGATCAGCTTCAGTTACACCGCTATCAATATTATACGATCCGTGACCGTGGA 959

N3.10 ATTCATCATGATCAGCTTCAGTTACACCGCTATCAATATTATACGATCCGTGACCGTGGA 959

A1.3 ATTCATCATGATCAGCTTCAGTTACACCGCTATCAATATTATACGATCCGTGACCGTGGA 959

N1.2 ATTCATCATGATCAGCTTCAGTTACACCGCTATCAATATTATACGATCCGTGACCGTGGA 959

N1.5 ATTCATCATGATCAGCTTCAGTTACACCGCTATCAATATTATACGATCCGTGACCGTGGA 959

N1.27 ATTCATCATGATCAGCTTCAGTTACACCGCTATCAATATTATACGATCCGTGACCGTGGA 959

A1.2 ATTCATCATGATCAGCTTCAGTTACACCGCTATCAATATTATACGATCCGTGACCGTGGA 959

A1.7 ATTCATCATGATCAGCTTCAGTTACACCGCTATCAATATTATACGATCCGTGACCGTGGA 959

N3.9 ATTCATCATGATCAGCTTCAGTTACACCGCTATCAATATTATACGATCCGTGACCGTGGA 959

************************************************************

MG637361.1 AAAAGAAATGCAATTGAAGGAAACGATGAAGATCATGGGACTCCCAACGTGGCTGCATTG 1019

A1.9 AAAAGAAATGCAATTGAAGGAAACGATGAAGATCATGGGACTCCCAACGTGGCTGCATTG 1019

N1.8 AAAAGAAATGCAATTGAAGGAAACGATGAAGATCATGGGACTCCCAACGTGGCTGCATTG 1020

A1.4 AAAAGAAATGCAATTGAAGGAAACGATGAAGATCATGGGACTCCCAACGTGGCTGCATTG 1019

A3.1 AAAAGAAATGCAATTGAAGGAAACGATGAAGATCATGGGACTCCCAACGTGGCTGCATTG 1019

A3.4 AAAAGAAATGCAATTGAAGGAAACGATGAAGATCATGGGACTCCCAACGTGGCTGCATTG 1019

A3.6 AAAAGAAATGCAATTGAAGGAAACGATGAAGATCATGGGACTCCCAACGTGGCTGCATTG 1019

A3.7 AAAAGAAATGCAATTGAAGGAAACGATGAAGATCATGGGACTCCCAACGTGGCTGCATTG 1019

A3.8 AAAAGAAATGCAATTGAAGGAAACGATGAAGATCATGGGACTCCCAACGTGGCTGCATTG 1019

N3.1 AAAAGAAATGCAATTGAAGGAAACGATGAAGATCATGGGACTCCCAACGTGGCTGCATTG 1019

N3.6 AAAAGAAATGCAATTGAAGGAAACGATGAAGATCATGGGACTCCCAACGTGGCTGCATTG 1019

N3.3 AAAAGAAATGCAATTGAAGGAAACGATGAAGATCATGGGACTCCCAACGTGGCTGCATTG 1019

N3.10 AAAAGAAATGCAATTGAAGGAAACGATGAAGATCATGGGACTCCCAACGTGGCTGCATTG 1019

A1.3 AAAAGAAATGCAATTGAAGGAAACGATGAAGATCATGGGACTCCCAACGTGGCTGCATTG 1019

N1.2 AAAAGAAATGCAATTGAAGGAAACGATGAAGATCATGGGACTCCCAACGTGGCTGCATTG 1019

N1.5 AAAAGAAATGCAATTGAAGGAAACGATGAAGATCATGGGACTCCCAACGTGGCTGCATTG 1019

N1.27 AAAAGAAATGCAATTGAAGGAAACGATGAAGATCATGGGACTCCCAACGTGGCTGCATTG 1019

A1.2 AAAAGAAATGCAATTGAAGGAAACGATGAAGATCATGGGACTCCCAACGTGGCTGCATTG 1019

A1.7 AAAAGAAATGCAATTGAAGGAAACGATGAAGATCATGGGACTCCCAACGTGGCTGCATTG 1019

N3.9 AAAAGAAATGCAATTGAAGGAAACGATGAAGATCATGGGACTCCCAACGTGGCTGCATTG 1019

************************************************************

MG637361.1 GATGGCATGGTTTTTTAAACAATTTATTTATTTGCTGATTGCTTCGGTTTTGATACTTGT 1079

A1.9 GATGGCATGGTTTTTTAAACAATTTATTTATTTGCTAATTGCTTCGGTTTTGATACTTGT 1079

N1.8 GATGGCATGGTTTTTTAAACAATTTATTTATTTGCTGATTGCTTCGGTTTTGATACTTGT 1080

A1.4 GATGGCATGGTTTTTTAAACAATTTATTTATTTGCTAATTGCTTCGGTTTTGATACTTGT 1079

A3.1 GATGGCATGGTTTTTTAAACAATTTATTTATTTGCTGATTGCTTCGGTTTTGATACTTGT 1079

A3.4 GATGGCATGGTTTTTTAAACAATTTATTTATTTGCTGATTGCTTCGGTTTTGATACTTGT 1079

A3.6 GATGGCATGGTTTTTTAAACAATTTATTTATTTGCTGATTGCTTCGGTTTTGATACTTGT 1079

A3.7 GATGGCATGGTTTTTTAAACAATTTATTTATTTGCTGATTGCTTCGGTTTTGATACTTGT 1079

A3.8 GATGGCATGGTTTTTTAAACAATTTATTTATTTGCTGATTGCTTCGGTTTTGATACTTGT 1079

N3.1 GATGGCATGGTTTTTTAAACAATTTATTTATTTGCTGATTGCTTCGGTTTTGATACTTGT 1079

N3.6 GATGGCATGGTTTTTTAAACAATTTATTTATTTGCTAATTGCTTCGGTTTTGATACTTGT 1079

N3.3 GATGGCATGGTTTTTTAAACAATTTATTTATTTGCTGATTGCTTCGGTTTTGATACTTGT 1079

N3.10 GATGGCATGGTTTTTTAAACAATTTATTTATTTGCTGATTGCTTCGGTTTTGATACTTGT 1079

A1.3 GATGGCATGGTTTTTTAAACAATTTATTTATTTGCTGATTGCTTCGGTTTTGATACTTGT 1079

N1.2 GATGGCATGGTTTTTTAAACAATTTATTTATTTGCTGATTGCTTCGGTTTTGATACTTGT 1079

N1.5 GATGGCATGGTTTTTTAAACAATTTATTTATTTGCTGATTGCTTCGGTTTTGATACTTGT 1079

N1.27 GATGGCATGGTTTTTTAAACAATTTATTTATTTGCTGATTGCTTCGGTTTTGATACTTGT 1079

A1.2 GATGGCATGGTTTTTTAAACAATTTATTTATTTGCTAATTGCTTCGGTTTTGATACTTGT 1079

A1.7 GATGGCATGGTTTTTTAAACAATTTATTTATTTGCTAATTGCTTCGGTTTTGATACTTGT 1079

N3.9 GATGGCATGGTTTTTTAAACAATTTATTTATTTGCTAATTGCTTCGGTTTTGATACTTGT 1079

************************************ ***********************

MG637361.1 TATATTAAAGGTAAATTGGTTTACTACAGAAGAAGGCTTTAGCGACTATGCCGTATTCAC 1139

A1.9 TATATTAAAGGTAAATTGGTTTACTACAGAAGAAGGCTTTAGCGACTATGCCGTATTCAC 1139

N1.8 TATATTAAAG-------------------------------------------------- 1090

A1.4 TATATTAAAGGTAAATTGGTTTACTACAGAAGAAGGCTTTAGCGACTATGCCGTATTCAC 1139

A3.1 TATATTAAAGGTAAATTGGTTTACTACAGAAGAAGGCTTTAGCGACTATGCCGTATTCAC 1139

A3.4 TATATTAAAGGTAAATTGGTTTACTACAGAAGAAGGCTTTAGCGACTATGCCGTATTCAC 1139

A3.6 TATATTAAAGGTAAATTGGTTTACTACAGAAGAAGGCTTTAGCGACTATGCCGTATTCAC 1139

A3.7 TATATTAAAGGTAAATTGGTTTACTACAGAAGAAGGCTTTAGCGACTATGCCGTATTCAC 1139

A3.8 TATATTAAAGGTAAATTGGTTTACTACAGAAGAAGGCTTTAGCGACTATGCCGTATTCAC 1139

N3.1 TATATTAAAGGTAAATTGGTTTACTACAGAAGAAGGCTTTAGCGACTATGCCGTATTCAC 1139

N3.6 TATATTAAAGGTAAATTGGTTTACTACAGAAGAAGGCTTTAGCGACTATGCCGTATTCAC 1139

N3.3 TATATTAAAGGTAAATTGGTTTACTACAGAAGAAGGCTTTAGCGACTATGCCGTATTCAC 1139

N3.10 TATATTAAAGGTAAATTGGTTTACTACAGAAGAAGGCTTTAGCGACTATGCCGTATTCAC 1139

A1.3 TATATTAAAG-------------------------------------------------- 1089

N1.2 TATATTAAAG-------------------------------------------------- 1089

N1.5 TATATTAAAG-------------------------------------------------- 1089

N1.27 TATATTAAAG-------------------------------------------------- 1089

A1.2 TATATTAAAGGTAAATTGGTTTACTACAGAAGAAGGCTTTAGCGACTATGCCGTATTCAC 1139

A1.7 TATATTAAAGGTAAATTGGTTTACTACAGAAGAAGGCTTTAGCGACTATGCCGTATTCAC 1139

N3.9 TATATTAAAGGTAAATTGGTTTACTACAGAAGAAGGCTTTAGCGACTATGCCGTATTCAC 1139

**********

MG637361.1 TAATACACCTTGGACCGTCCTCTTCTTCTTCCTAACACTGTATCTTACGTGTACCATATT 1199

A1.9 TAATACACCTTGGACCGTCCTCTTCTTCTTCCTAACACTGTATCTTACGTGTACCATATT 1199

N1.8 ------------------------------------------------------------ 1090

A1.4 TAATACACCTTGGACCGTCCTCTTCTTCTTCCTAACACTGTATCTTACGTGTACCATATT 1199

A3.1 TAATACACCTTGGACCGTCCTCTTCTTCTTCCTAACACTGTATCTTACGTGTACCATATT 1199

A3.4 TAATACACCTTGGACCGTCCTCTTCTTCTTCCTAACACTGTATCTTACGTGTACCATATT 1199

A3.6 TAATACACCTTGGACCGTCCTCTTCTTCTTCCTAACACTGTATCTTACGTGTACCATATT 1199

A3.7 TAATACACCTTGGACCGTCCTCTTCTTCTTCCTAACACTGTATCTTACGTGTACCATATT 1199

A3.8 TAATACACCTTGGACCGTCCTCTTCTTCTTCCTAACACTGTATCTTACGTGTACCATATT 1199

N3.1 TAATACACCTTGGACCGTCCTCTTCTTCTTCCTAACACTGTATCTTACGTGTACCATATT 1199

N3.6 TAATACACCTTGGACCGTCCTCTTCTTCTTCCTAACACTGTATCTTACGTGTACCATATT 1199

N3.3 TAATACACCTTGGACCGTCCTCTTCTTCTTCCTAACACTGTATCTTACGTGTACCATATT 1199

N3.10 TAATACACCTTGGACCGTCCTCTTCTTCTTCCTAACACTGTATCTTACGTGTACCATATT 1199

A1.3 ------------------------------------------------------------ 1089

N1.2 ------------------------------------------------------------ 1089

N1.5 ------------------------------------------------------------ 1089

N1.27 ------------------------------------------------------------ 1089

A1.2 TAATACACCTTGGACCGTCCTCTTCTTCTTCCTAACACTGTATCTTACGTGTACCATATT 1199

A1.7 TAATACACCTTGGACCGTCCTCTTCTTCTTCCTAACACTGTATCTTACGTGTACCATATT 1199

N3.9 TAATACACCTTGGACCGTCCTCTTCTTCTTCCTAACACTGTATCTTACGTGTACCATATT 1199

MG637361.1 TTTCTGTTTCATGATAAGTGGTTTCTTTTCAAAAGCCAGTACAGCGGCGTTGTTTGGTGG 1259

A1.9 TTTCTGTTTCATGATAAGTGGTTTCTTTTCAAAAGCCAGTACAGCGGCGTTGTTTGGTGG 1259

N1.8 -----------------------------------CCAGTACAGCGGCGTTGTTTGGTGG 1115

A1.4 TTTCTGTTTCATGATAAGTGGTTTCTTTTCAAAAGCCAGTACAGCGGCGTTGTTTGGTGG 1259

A3.1 TTTCTGTTTCATGATAAGTGGTTTCTTTTCAAAAGCCAGTACAGCGGCGTTGTTTGGTGG 1259

A3.4 TTTCTGTTTCATGATAAGTGGTTTCTTTTCAAAAGCCAGTACAGCGGCGTTGTTTGGTGG 1259

A3.6 TTTCTGTTTCATGATAAGTGGTTTCTTTTCAAAAGCCAGTACAGCGGCGTTGTTTGGTGG 1259

A3.7 TTTCTGTTTCATGATAAGTGGTTTCTTTTCAAAAGCCAGTACAGCGGCGTTGTTTGGTGG 1259

A3.8 TTTCTGTTTCATGATAAGTGGTTTCTTTTCAAAAGCCAGTACAGCGGCGTTGTTTGGTGG 1259

N3.1 TTTCTGTTTCATGATAAGTGGTTTCTTTTCAAAAGCCAGTACAGCGGCGTTGTTTGGTGG 1259

N3.6 TTTCTGTTTCATGATAAGTGGTTTCTTTTCAAAAGCCAGTACAGCGGCGTTGTTTGGTGG 1259

N3.3 TTTCTGTTTCATGATAAGTGGTTTCTTTTCAAAAGCCAGTACAGCGGCGTTGTTTGGTGG 1259

N3.10 TTTCTGTTTCATGATAAGTGGTTTCTTTTCAAAAGCCAGTACAGCGGCGTTGTTTGGTGG 1259

A1.3 -----------------------------------CCAGTACAGCGGCGTTGTTTGGTGG 1114

N1.2 -----------------------------------CCAGTACAGCGGCGTTGTTTGGTGG 1114

N1.5 -----------------------------------CCAGTACAGCGGCGTTGTTTGGTGG 1114

N1.27 -----------------------------------CCAGTACAGCGGCGTTGTTTGGTGG 1114

A1.2 TTTCTGTTTCATGATAAGTGGTTTCTTTTCAAAAGCCAGTACAGCGGCGTTGTTTGGTGG 1259

A1.7 TTTCTGTTTCATGATAAGTGGTTTCTTTTCAAAAGCCAGTACAGCGGCGTTGTTTGGTGG 1259

N3.9 TTTCTGTTTCATGATAAGTGGTTTCTTTTCAAAAGCCAGTACAGCGGCGTTGTTTGGTGG 1259

*************************

MG637361.1 GGTGATCTGGTTTCTGACGTATATCCCCGCATTCCTCCTGGCTATGGACGTGAACATGTC 1319

A1.9 GGTGATCTGGTTTCTGACGTATATCCCCGCATTCCTCCTGGCTATGGACGTGAACATGTC 1319

N1.8 GGTGATCTGGTTTCTGACGTATATCCCCGCATTCCTCCTGGCTATGGACGTGAACATGTC 1175

A1.4 GGTGATCTGGTTTCTGACGTATATCCCCGCATTCCTCCTGGCTATGGACGTGAACATGTC 1319

A3.1 GGTGATCTGGTTTCTGACGTATATCCCCGCATTCCTCCTGGCTATGGACGTGAACATGTC 1319

A3.4 GGTGATCTGGTTTCTGACGTATATCCCCGCATTCCTCCTGGCTATGGACGTGAACATGTC 1319

A3.6 GGTGATCTGGTTTCTGACGTATATCCCCGCATTCCTCCTGGCTATGGACGTGAACATGTC 1319

A3.7 GGTGATCTGGTTTCTGACGTATATCCCCGCATTCCTCCTGGCTATGGACGTGAACATGTC 1319

A3.8 GGTGATCTGGTTTCTGACGTATATCCCCGCATTCCTCCTGGCTATGGACGTGAACATGTC 1319

N3.1 GGTGATCTGGTTTCTGACGTATATCCCCGCATTCCTCCTGGCTATGGACGTGAACATGTC 1319

N3.6 GGTGATCTGGTTTCTGACGTATATCCCCGCATTCCTCCTGGCTATGGACGTGAACATGTC 1319

N3.3 GGTGATCTGGTTTCTGACGTATATCCCCGCATTCCTCCTGGCTATGGACGTGAACATGTC 1319

N3.10 GGTGATCTGGTTTCTGACGTATATCCCCGCATTCCTCCTGGCTATGGACGTGAACATGTC 1319

A1.3 GGTGATCTGGTTTCTGACGTATATCCCCGCATTCCTCCTGGCTATGGACGTGAACATGTC 1174

N1.2 GGTGATCTGGTTTCTGACGTATATCCCCGCATTCCTCCTGGCTATGGACGTGAACATGTC 1174

N1.5 GGTGATCTGGTTTCTGACGTATATCCCCGCATTCCTCCTGGCTATGGACGTGAACATGTC 1174

N1.27 GGTGATCTGGTTTCTGACGTATATCCCCGCATTCCTCCTGGCTATGGACGTGAACATGTC 1174

A1.2 GGTGATCTGGTTTCTGACGTATATCCCCGCATTCCTCCTGGCTATGGACGTGAACATGTC 1319

A1.7 GGTGATCTGGTTTCTGACGTATATCCCCGCATTCCTCCTGGCTATGGACGTGAACATGTC 1319

N3.9 GGTGATCTGGTTTCTGACGTATATCCCCGCATTCCTCCTGGCTATGGACGTGAACATGTC 1319

************************************************************

MG637361.1 TACCTCTCTACAAGCGGTCACCTGCCTAATGCTCAACTCCGCCATGTCTTACGGCTTCCA 1379

A1.9 TACCTCTCTACAAGCGGTCACCTGCCTAATGCTCAACTCCGCCATGTCTTACGGCTTCCA 1379

N1.8 TACCTCTCTACAAGCGGTCACCTGCCTAATGCTCAACTCCGCCATGTCTTACGGCTTCCA 1235

A1.4 TACCTCTCTACAAGCGGTCACCTGCCTAATGCTCAACTCCGCCATGTCTTACGGCTTCCA 1379

A3.1 TACCTCTCTACAAGCGGTCACCTGCCTAATGCTCAACTCCGCCATGTCTTACGGCTTCCA 1379

A3.4 TACCTCTCTACAAGCGGTCACCTGCCTAATGCTCAACTCCGCCATGTCTTACGGCTTCCA 1379

A3.6 TACCTCTCTACAAGCGGTCACCTGCCTAATGCTCAACTCCGCCATGTCTTACGGCTTCCA 1379

A3.7 TACCTCTCTACAAGCGGTCACCTGCCTAATGCTCAACTCCGCCATGTCTTACGGCTTCCA 1379

A3.8 TACCTCTCTACAAGCGGTCACCTGCCTAATGCTCAACTCCGCCATGTCTTACGGCTTCCA 1379

N3.1 TACCTCTCTACAAGCGGTCACCTGCCTAATGCTCAACTCCGCCATGTCTTACGGCTTCCA 1379

N3.6 TACCTCTCTACAAGCGGTCACCTGCCTAATGCTCAACTCCGCCATGTCTTACGGCTTCCA 1379

N3.3 TACCTCTCTACAAGCGGTCACCTGCCTAATGCTCAACTCCGCCATGTCTTACGGCTTCCA 1379

N3.10 TACCTCTCTACAAGCGGTCACCTGCCTAATGCTCAACTCCGCCATGTCTTACGGCTTCCA 1379

A1.3 TACCTCTCTACAAGCGGTCACCTGCCTAATGCTCAACTCCGCCATGTCTTACGGCTTCCA 1234

N1.2 TACCTCTCTACAAGCGGTCACCTGCCTAATGCTCAACTCCGCCATGTCTTACGGCTTCCA 1234

N1.5 TACCTCTCTACAAGCGGTCACCTGCCTAATGCTCAACTCCGCCATGTCTTACGGCTTCCA 1234

N1.27 TACCTCTCTACAAGCGGTCACCTGCCTAATGCTCAACTCCGCCATGTCTTACGGCTTCCA 1234

A1.2 TACCTCTCTACAAGCGGTCACCTGCCTAATGCTCAACTCCGCCATGTCTTACGGCTTCCA 1379

A1.7 TACCTCTCTACAAGCGGTCACCTGCCTAATGCTCAACTCCGCCATGTCTTACGGCTTCCA 1379

N3.9 TACCTCTCTACAAGCGGTCACCTGCCTAATGCTCAACTCCGCCATGTCTTACGGCTTCCA 1379

************************************************************

MG637361.1 GCTGTTACTGGCCCGGGAAAGTACCGGAGGAATGCAGTGGGGTGATTTTATGACGTCACC 1439

A1.9 GCTGTTACTGGCCCGGGAAAGTACCGGAGGAATGCAGTGGGGTGATTTTATGACGTCACC 1439

N1.8 GCTGTTACTGGCCCGGGAAAGTACCGGAGGAATGCAGTGGGGTGATTTTATGACGTCACC 1295

A1.4 GCTGTTACTGGCCCGGGAAAGTACCGGAGGAATGCAGTGGGGTGATTTTATGACGTCACC 1439

A3.1 GCTGTTACTGGCCCGGGAAAGTACCGGAGGAATGCAGTGGGGTGATTTTATGACGTCACC 1439

A3.4 GCTGTTACTGGCCCGGGAAAGTACCGGAGGAATGCAGTGGGGTGATTTTATGACGTCACC 1439

A3.6 GCTGTTACTGGCCCGGGAAAGTACCGGAGGAATGCAGTGGGGTGATTTTATGACGTCACC 1439

A3.7 GCTGTTACTGGCCCGGGAAAGTACCGGAGGAATGCAGTGGGGTGATTTTATGACGTCACC 1439

A3.8 GCTGTTACTGGCCCGGGAAAGTACCGGAGGAATGCAGTGGGGTGATTTTATGACGTCACC 1439

N3.1 GCTGTTACTGGCCCGGGAAAGTACCGGAGGAATGCAGTGGGGTGATTTTATGACGTCACC 1439

N3.6 GCTGTTACTGGCCCGGGAAAGTACCGGAGGAATGCAGTGGGGTGATTTTATGACGTCACC 1439

N3.3 GCTGTTACTGGCCCGGGAAAGTACCGGAGGAATGCAGTGGGGTGATTTTATGACGTCACC 1439

N3.10 GCTGTTACTGGCCCGGGAAAGTACCGGAGGAATGCAGTGGGGTGATTTTATGACGTCACC 1439

A1.3 GCTGTTACTGGCCCGGGAAAGTACCGGAGGAATGCAGTGGGGTGATTTTATGACGTCACC 1294

N1.2 GCTGTTACTGGCCCGGGAAAGTACCGGAGGAATGCAGTGGGGTGATTTTATGACGTCACC 1294

N1.5 GCTGTTACTGGCCCGGGAAAGTACCGGAGGAATGCAGTGGGGTGATTTTATGACGTCACC 1294

N1.27 GCTGTTACTGGCCCGGGAAAGTACCGGAGGAATGCAGTGGGGTGATTTTATGACGTCACC 1294

A1.2 GCTGTTACTGGCCCGGGAAAGTACCGGAGGAATGCAGTGGGGTGATTTTATGACGTCACC 1439

A1.7 GCTGTTACTGGCCCGGGAAAGTACCGGAGGAATGCAGTGGGGTGATTTTATGACGTCACC 1439

N3.9 GCTGTTACTGGCCCGGGAAAGTACCGGAGGAATGCAGTGGGGTGATTTTATGACGTCACC 1439

************************************************************

MG637361.1 AGCAACGGACTCGTCACGATTCGTATTCGGTCACGTCGTTATAATGATGGCTTTGAACTG 1499

A1.9 AGCAACGGACTCGTCACGATTCGTATTCGGTCACGTCGTTATAATGATGGCTTTGAACTG 1499

N1.8 AGCAACGGACTCGTCACGATTCGTATTCGGTCACGTCGTTATAATGATGGCTTTGAACTG 1355

A1.4 AGCAACGGACTCGTCACGATTCGTATTCGGTCACGTCGTTATAATGATGGCTTTGAACTG 1499

A3.1 AGCAACGGACTCGTCACGATTCGTATTCGGTCACGTCGTTATAATGATGGCTTTGAACTG 1499

A3.4 AGCAACGGACTCGTCACGATTCGTATTCGGTCACGTCGTTATAATGATGGCTTTGAACTG 1499

A3.6 AGCAACGGACTCGTCACGATTCGTATTCGGTCACGTCGTTATAATGATGGCTTTGAACTG 1499

A3.7 AGCAACGGACTCGTCACGATTCGTATTCGGTCACGTCGTTATAATGATGGCTTTGAACTG 1499

A3.8 AGCAACGGACTCGTCACGATTCGTATTCGGTCACGTCGTTATAATGATGGCTTTGAACTG 1499

N3.1 AGCAACGGACTCGTCACGATTCGTATTCGGTCACGTCGTTATAATGATGGCTTTGAACTG 1499

N3.6 AGCAACGGACTCGTCACGATTCGTATTCGGTCACGTCGTTATAATGATGGCTTTGAACTG 1499

N3.3 AGCAACGGACTCGTCACGATTCGTATTCGGTCACGTCGTTATAATGATGGCTTTGAACTG 1499

N3.10 AGCAACGGACTCGTCACGATTCGTATTCGGTCACGTCGTTATAATGATGGCTTTGAACTG 1499

A1.3 AGCAACGGACTCGTCACGATTCGTATTCGGTCACGTCGTTATAATGATGGCTTTGAACTG 1354

N1.2 AGCAACGGACTCGTCACGATTCGTATTCGGTCACGTCGTTATAATGATGGCTTTGAACTG 1354

N1.5 AGCAACGGACTCGTCACGATTCGTATTCGGTCACGTCGTTATAATGATGGCTTTGAACTG 1354

N1.27 AGCAACGGACTCGTCACGATTCGTATTCGGTCACGTCGTTATAATGATGGCTTTGAACTG 1354

A1.2 AGCAACGGACTCGTCACGATTCGTATTCGGTCACGTCGTTATAATGATGGCTTTGAACTG 1499

A1.7 AGCAACGGACTCGTCACGATTCGTATTCGGTCACGTCGTTATAATGATGGCTTTGAACTG 1499

N3.9 AGCAACGGACTCGTAACGATTCGTATTCGGTCACGTCGTTATAATGATGGCTTTGAACTG 1499

************** *********************************************

MG637361.1 TGTGCTCTACATGTTGATTGCCCTATATCTAGAGCAAGTACTACCCGGGCCGTATGGCAC 1559

A1.9 TGTGCTCTACATGTTGATTGCCCTATATCTAGAGCAAGTACTACCCGGGCCGTATGGCAC 1559

N1.8 TGTGCTCTACATGTTGATTGCCCTATATCTAGAGCAAGTACTACCCGGGCCGTATGGCAC 1415

A1.4 TGTGCTCTACATGTTGATTGCCCTATATCTAGAGCAAGTACTACCCGGGCCGTATGGCAC 1559

A3.1 TGTGCTCTACATGTTGATTGCCCTATATCTAGAGCAAGTACTACCCGGGCCGTATGGCAC 1559

A3.4 TGTGCTCTACATGTTGATTGCCCTATATCTAGAGCAAGTACTACCCGGGCCGTATGGCAC 1559

A3.6 TGTGCTCTACATGTTGATTGCCCTATATCTAGAGCAAGTACTACCCGGGCCGTATGGCAC 1559

A3.7 TGTGCTCTACATGTTGATTGCCCTATATCTAGAGCAAGTACTACCCGGGCCGTATGGCAC 1559

A3.8 TGTGCTCTACATGTTGATTGCCCTATATCTAGAGCAAGTACTACCCGGGCCGTATGGCAC 1559

N3.1 TGTGCTCTACATGTTGATTGCCCTATATCTAGAGCAAGTACTACCCGGGCCGTATGGCAC 1559

N3.6 TGTGCTCTACATGTTGATTGCCCTATATCTAGAGCAAGTACTACCCGGGCCGTATGGCAC 1559

N3.3 TGTGCTCTACATGTTGATTGCCCTATATCTAGAGCAAGTACTACCCGGGCCGTATGGCAC 1559

N3.10 TGTGCTCTACATGTTGATTGCCCTATATCTAGAGCAAGTACTACCCGGGCCGTATGGCAC 1559

A1.3 TGTGCTCTACATGTTGATTGCCCTATATCTAGAGCAAGTACTACCCGGGCCGTATGGCAC 1414

N1.2 TGTGCTCTACATGTTGATTGCCCTATATCTAGAGCAAGTACTACCCGGGCCGTATGGCAC 1414

N1.5 TGTGCTCTACATGTTGATTGCCCTATATCTAGAGCAAGTACTACCCGGGCCGTATGGCAC 1414

N1.27 TGTGCTCTACATGTTGATTGCCCTATATCTAGAGCAAGTACTACCCGGGCCGTATGGCAC 1414

A1.2 TGTGCTCTACATGTTGATTGCCCTATATCTAGAGCAAGTACTACCCGGGCCGTATGGCAC 1559

A1.7 TGTGCTCTACATGTTGATTGCCCTATATCTAGAGCAAGTACTACCCGGGCCGTATGGCAC 1559

N3.9 TGTGCTCTACATGTTGATTGCCCTATATCTAGAGCAAGTACTACCCGGGCCGTATGGCAC 1559

************************************************************

MG637361.1 ACCGAAGCCCTGGTATTTCTTCGTCCAAAGACAGTTCTGGTGTAGCAGCAAAACTACTCA 1619

A1.9 ACCGAAGCCCTGGTATTTCTTCGTCCAAAGACAGTTCTGGTGTAGCAGCAAAACTACTCA 1619

N1.8 ACCGAAGCCCTGGTATTTCTTCGTCCAAAGACAGTTCTGGTGTAGCAGCAAAACTACTCA 1475

A1.4 ACCGAAGCCCTGGTATTTCTTCGTCCAAAGACAGTTCTGGTGTAGCAGCAAAACTACTCA 1619

A3.1 ACCGAAGCCCTGGTATTTCTTCGTCCAAAGACAGTTCTGGTGTAGCAGCAAAACTACTCA 1619

A3.4 ACCGAAGCCCTGGTATTTCTTCGTCCAAAGACAGTTCTGGTGTAGCAGCAAAACTACTCA 1619

A3.6 ACCGAAGCCCTGGTATTTCTTCGTCCAAAGACAGTTCTGGTGTAGCAGCAAAACTACTCA 1619

A3.7 ACCGAAGCCCTGGTATTTCTTCGTCCAAAGACAGTTCTGGTGTAGCAGCAAAACTACTCA 1619

A3.8 ACCGAAGCCCTGGTATTTCTTCGTCCAAAGACAGTTCTGGTGTAGCAGCAAAACTACTCA 1619

N3.1 ACCGAAGCCCTGGTATTTCTTCGTCCAAAGACAGTTCTGGTGTAGCAGCAAAACTACTCA 1619

N3.6 ACCGAAGCCCTGGTATTTCTTCGTCCAAAGACAGTTCTGGTGTAGCAGCAAAACTACTCA 1619

N3.3 ACCGAAGCCCTGGTATTTCTTCGTCCAAAGACAGTTCTGGTGTAGCAGCAAAACTACTCA 1619

N3.10 ACCGAAGCCCTGGTATTTCTTCGTCCAAAGACAGTTCTGGTGTAGCAGCAAAACTACTCA 1619

A1.3 ACCGAAGCCCTGGTATTTCTTCGTCCAAAGACAGTTCTGGTGTAGCAGCAAAACTACTCA 1474

N1.2 ACCGAAGCCCTGGTATTTCTTCGTCCAAAGACAGTTCTGGTGTAGCAGCAAAACTACTCA 1474

N1.5 ACCGAAGCCCTGGTATTTCTTCGTCCAAAGACAGTTCTGGTGTAGCAGCAAAACTACTCA 1474

N1.27 ACCGAAGCCCTGGTATTTCTTCGTCCAAAGACAGTTCTGGTGTAGCAGCAAAACTACTCA 1474

A1.2 ACCGAAGCCCTGGTATTTCTTCGTCCAAAGACAGTTCTGGTGTAGCAGCAAAACTACTCA 1619

A1.7 ACCGAAGCCCTGGTATTTCTTCGTCCAAAGACAGTTCTGGTGTAGCAGCAAAACTACTCA 1619

N3.9 ACCGAAGCCCTGGTATTTCTTCGTCCAAAGACAGTTCTGGTGTAGCAGCAAAACTACTCA 1619

************************************************************

MG637361.1 TGATATCGGTACAGACAACAGCGACACATCAAGTTTAACAAAAGAAAGCGACCCTACAGA 1679

A1.9 TGATATCGGTACAGACAACAGCGACACATCAAGTTTAACAAAAGAAAGCGACCCTACAGA 1679

N1.8 TGATATCGGTACAGACAACAGCGACACATCAAGTTTAACAAAAGAAAGCGACCCTACAGA 1535

A1.4 TGATATCGGTACAGACAACAGCGACACATCAAGTTTAACAAAAGAAAGCGACCCTACAGA 1679

A3.1 TGATATCGGTACAGACAACAGCGACACATCAAGTTTAACAAAAGAAAGCGACCCTACAGA 1679

A3.4 TGATATCGGTACAGACAACAGCGACACATCAAGTTTAACAAAAGAAAGCGACCCTACAGA 1679

A3.6 TGATATCGGTACAGACAACAGCGACACATCAAGTTTAACAAAAGAAAGCGACCCTACAGA 1679

A3.7 TGATATCGGTACAGACAACAGCGACACATCAAGTTTAACAAAAGAAAGCGACCCTACAGA 1679

A3.8 TGATATCGGTACAGACAACAGCGACACATCAAGTTTAACAAAAGAAAGCGACCCTACAGA 1679

N3.1 TGATATCGGTACAGACAACAGCGACACATCAAGTTTAACAAAAGAAAGCGACCCTACAGA 1679

N3.6 TGATATCGGTACAGACAACAGCGACACATCAAGTTTAACAAAAGAAAGCGACCCTACAGA 1679

N3.3 TGATATCGGTACAGACAACAGCGACACATCAAGTTTAACAAAAGAAAGCGACCCTACAGA 1679

N3.10 TGATATCGGTACAGACAACAGCGACACATCAAGTTTAACAAAAGAAAGCGACCCTACAGA 1679

A1.3 TGATATCGGTACAGACAACAGCGACACATCAAGTTTAACAAAAGAAAGCGACCCTACAGA 1534

N1.2 TGATATCGGTACAGACAACAGCGACACATCAAGTTTAACAAAAGAAAGCGACCCTACAGA 1534

N1.5 TGATATCGGTACAGACAACAGCGACACATCAAGTTTAACAAAAGAAAGCGACCCTACAGA 1534

N1.27 TGATATCGGTACAGACAACAGCGACACATCAAGTTTAACAAAAGAAAGCGACCCTACAGA 1534

A1.2 TGATATCGGTACAGACAACAGCGACACATCAAGTTTAACAAAAGAAAGCGACCCTACAGA 1679

A1.7 TGATATCGGTACAGACAACAGCGACACATCAAGTTTAACAAAAGAAAGCGACCCTACAGA 1679

N3.9 TGATATCGGTACAGACAACAGCGACACATCAAGTTTAACAAAAGAAAGCGACCCTACAGA 1679

************************************************************

MG637361.1 CCTTCCGATTGGAGTTAAAATACAAAACCTTAAAAAGGTTTACGGGAGCAACGTTGCGGT 1739

A1.9 CCTTCCGATTGGAGTTAAAATACAAAACCTTAAAAAGGTTTACGGGAGCAACGTTGCGGT 1739

N1.8 CCTTCCGATTGGAGTTAAAATACAAAACCTTAAAAAGGTTTACGGGAGCAACGTTGCGGT 1595

A1.4 CCTTCCGATTGGAGTTAAAATACAAAACCTTAAAAAGGTTTACGGGAGCAACGTTGCGGT 1739

A3.1 CCTTCCGATTGGAGTTAAAATACAAAACCTTAAAAAGGTTTACGGGAGCAACGTTGCGGT 1739

A3.4 CCTTCCGATTGGAGTTAAAATACAAAACCTTAAAAAGGTTTACGGGAGCAACGTTGCGGT 1739

A3.6 CCTTCCGATTGGAGTTAAAATACAAAACCTTAAAAAGGTTTACGGGAGCAACGTTGCGGT 1739

A3.7 CCTTCCGATTGGAGTTAAAATACAAAACCTTAAAAAGGTTTACGGGAGCAACGTTGCGGT 1739

A3.8 CCTTCCGATTGGAGTTAAAATACAAAACCTTAAAAAGGTTTACGGGAGCAACGTTGCGGT 1739

N3.1 CCTTCCGATTGGAGTTAAAATACAAAACCTTAAAAAGGTTTACGGGAGCAACGTTGCGGT 1739

N3.6 CCTTCCGATTGGAGTTAAAATACAAAACCTTAAAAAGGTTTACGGGAGCAACGTTGCGGT 1739

N3.3 CCTTCCGATTGGAGTTAAAATACAAAACCTTAAAAAGGTTTACGGGAGCAACGTTGCGGT 1739

N3.10 CCTTCCGATTGGAGTTAAAATACAAAACCTTAAAAAGGTTTACGGGAGCAACGTTGCGGT 1739

A1.3 CCTTCCGATTGGAGTTAAAATACAAAACCTTAAAAAGGTTTACGGGAGCAACGTTGCGGT 1594

N1.2 CCTTCCGATTGGAGTTAAAATACAAAACCTTAAAAAGGTTTACGGGAGCAACGTTGCGGT 1594

N1.5 CCTTCCGATTGGAGTTAAAATACAAAACCTTAAAAAGGTTTACGGGAGCAACGTTGCGGT 1594

N1.27 CCTTCCGATTGGAGTTAAAATACAAAACCTTAAAAAGGTTTACGGGAGCAACGTTGCGGT 1594

A1.2 CCTTCCGATTGGAGTTAAAATACAAAACCTTAAAAAGGTTTACGGGAGCAACGTTGCGGT 1739

A1.7 CCTTCCGATTGGAGTTAAAATACAAAACCTTAAAAAGGTTTACGGGAGCAACGTTGCGGT 1739

N3.9 CCTTCCGATTGGAGTTAAAATAAAAAACCTTAAAAAGGTTTACGGGAGCAACGTTGCGGT 1739

********************** *************************************

MG637361.1 AAACAATTTATCCCTCAACATTTACGACGACCAAATCACGGTTCTACTTGGACACAACGG 1799

A1.9 AAACAATTTATCCCTCAATATTTACGACGACCAAATCACGGTTCTACTTGGACACAACGG 1799

N1.8 AAACAATTTATCCCTCAACATTTACGACGACCAAATCACGGTTCTACTTGGACACAACGG 1655

A1.4 AAACAATTTATCCCTCAATATTTACGACGACCAAATCACGGTTCTACTTGGACACAACGG 1799

A3.1 AAACAATTTATCCCTCAACATTTACGACGACCAAATCACGGTTCTACTTGGACACAACGG 1799

A3.4 AAACAATTTATCCCTCAACATTTACGACGACCAAATCACGGTTCTACTTGGACACAACGG 1799

A3.6 AAACAATTTATCCCTCAACATTTACGACGACCAAATCACGGTTCTACTTGGACACAACGG 1799

A3.7 AAACAATTTATCCCTCAACATTTACGACGACCAAATCACGGTTCTACTTGGACACAACGG 1799

A3.8 AAACAATTTATCCCTCAACATTTACGACGACCAAATCACGGTTCTACTTGGACACAACGG 1799

N3.1 AAACAATTTATCCCTCAACATTTACGACGACCAAATCACGGTTCTACTTGGACACAACGG 1799

N3.6 AAACAATTTATCCCTCAACATTTACGACGACCAAATCACGGTTCTACTTGGACACAACGG 1799

N3.3 AAACAATTTATCCCTCAACATTTACGACGACCAAATCACGGTTCTACTTGGACACAACGG 1799

N3.10 AAACAATTTATCCCTCAACATTTACGACGACCAAATCACGGTTCTACTTGGACACAACGG 1799

A1.3 AAACAATTTATCCCTCAACATTTACGACGACCAAATCACGGTTCTACTTGGACACAACGG 1654

N1.2 AAACAATTTATCCCTCAACATTTACGACGACCAAATCACGGTTCTACTTGGACACAACGG 1654

N1.5 AAACAATTTATCCCTCAACATTTACGACGACCAAATCACGGTTCTACTTGGACACAACGG 1654

N1.27 AAACAATTTATCCCTCAACATTTACGACGACCAAATCACGGTTCTACTTGGACACAACGG 1654

A1.2 AAACAATTTATCCCTCAATATTTACGACGACCAAATCACGGTTCTACTTGGACACAACGG 1799

A1.7 AAACAATTTATCCCTCAATATTTACGACGACCAAATCACGGTTCTACTTGGACACAACGG 1799

N3.9 AAACAATTTATCCCTCAATATTTACGACGACCAAATCACGGTTCTACTTGGACACAACGG 1799

****************** *****************************************

MG637361.1 AGCGGGAAAATCCACAACCATTTCAATGCTCACAGGTAACGTGGACATAACCAGCGGGTC 1859

A1.9 AGCGGGAAAATCCACGACCATTTCAATGCTCACAGGTAACGTGGACATAACCAGCGGGTC 1859

N1.8 AGCGGGAAAATCCACAACCATTTCAATGCTCACAGGTAACGTGGACATAACCAGCGGGTC 1715

A1.4 AGCGGGAAAATCCACGACCATTTCAATGCTCACAGGTAACGTGGACATAACCAGCGGGTC 1859

A3.1 AGCGGGAAAATCCACAACCATTTCAATGCTCACAGGTAACGTGGACATAACCAGCGGGTC 1859

A3.4 AGCGGGAAAATCCACAACCATTTCAATGCTCACAGGTAACGTGGACATAACCAGCGGGTC 1859

A3.6 AGCGGGAAAATCCACAACCATTTCAATGCTCACAGGTAACGTGGACATAACCAGCGGGTC 1859

A3.7 AGCGGGAAAATCCACAACCATTTCAATGCTCACAGGTAACGTGGACATAACCAGCGGGTC 1859

A3.8 AGCGGGAAAATCCACAACCATTTCAATGCTCACAGGTAACGTGGACATAACCAGCGGGTC 1859

N3.1 AGCGGGAAAATCCACAACCATTTCAATGCTCACAGGTAACGTGGACATAACCAGCGGGTC 1859

N3.6 AGCGGGAAAATCCACAACCATTTCAATGCTCACAGGTAACGTGGACATAACCAGCGGGTC 1859

N3.3 AGCGGGAAAATCCACAACCATTTCAATGCTCACAGGTAACGTGGACATAACCAGCGGGTC 1859

N3.10 AGCGGGAAAATCCACAACCATTTCAATGCTCACAGGTAACGTGGACATAACCAGCGGGTC 1859

A1.3 AGCGGGAAAATCCACAACCATTTCAATGCTCACAGGTAACGTGGACATAACCAGCGGGTC 1714

N1.2 AGCGGGAAAATCCACAACCATTTCAATGCTCACAGGTAACGTGGACATAACCAGCGGGTC 1714

N1.5 AGCGGGAAAATCCACAACCATTTCAATGCTCACAGGTAACGTGGACATAACCAGCGGGTC 1714

N1.27 AGCGGGAAAATCCACAACCATTTCAATGCTCACAGGTAACGTGGACATAACCAGCGGGTC 1714

A1.2 AGCGGGAAAATCCACGACCATTTCAATGCTCACAGGTAACGTGGACATAACCAGCGGGTC 1859

A1.7 AGCGGGAAAATCCACGACCATTTCAATGCTCACAGGTAACGTGGACATAACCAGCGGGTC 1859

N3.9 AGCGGGAAAATCCACGACCATTTCAATGCTCACAGGTAACGTGGACATAACCAGCGGGTC 1859

*************** ********************************************

MG637361.1 GGTGACGGTGGCTGGCTACGACATAGAAAAACAAACAAGTTCAGCACGCTCACACATTGG 1919

A1.9 GGTGACGGTGGCTGGCTACGACATAGAAAAACAAACAAGTTCAGCACGCTCACACATTGG 1919

N1.8 GGTGACGGTGGCTGGCTACGACATAGAAAAACAAACAAGTTCAGCACGCTCACACATTGG 1775

A1.4 GGTGACGGTGGCTGGCTACGACATAGAAAAACAAACAAGTTCAGCACGCTCACACATTGG 1919

A3.1 GGTGACGGTGGCTGGCTACGACATAGAAAAACAAACAAGTTCAGCACGCTCACACATTGG 1919

A3.4 GGTGACGGTGGCTGGCTACGACATAGAAAAACAAACAAGTTCAGCACGCTCACACATTGG 1919

A3.6 GGTGACGGTGGCTGGCTACGACATAGAAAAACAAACAAGTTCAGCACGCTCACACATTGG 1919

A3.7 GGTGACGGTGGCTGGCTACGACATAGAAAAACAAACAAGTTCAGCACGCTCACACATTGG 1919

A3.8 GGTGACGGTGGCTGGCTACGACATAGAAAAACAAACAAGTTCAGCACGCTCACACATTGG 1919

N3.1 GGTGACGGTGGCTGGCTACGACATAGAAAAACAAACAAGTTCAGCACGCTCACACATTGG 1919

N3.6 GGTGACGGTGGCTGGCTACGACATAGAAAAACAAACAAGTTCAGCACGCTCACACATTGG 1919

N3.3 GGTGACGGTGGCTGGCTACGACATAGAAAAACAAACAAGTTCAGCACGCTCACACATTGG 1919

N3.10 GGTGACGGTGGCTGGCTACGACATAGAAAAACAAACAAGTTCAGCACGCTCACACATTGG 1919

A1.3 GGTGACGGTGGCTGGCTACGACATAGAAAAACAAACAAGTTCAGCACGCTCACACATTGG 1774

N1.2 GGTGACGGTGGCTGGCTACGACATAGAAAAACAAACAAGTTCAGCACGCTCACACATTGG 1774

N1.5 GGTGACGGTGGCTGGCTACGACATAGAAAAACAAACAAGTTCAGCACGCTCACACATTGG 1774

N1.27 GGTGACGGTGGCTGGCTACGACATAGAAAAACAAACAAGTTCAGCACGCTCACACATTGG 1774

A1.2 GGTGACGGTGGCTGGCTACGACATAGAAAAACAAACAAGTTCAGCACGCTCACACATTGG 1919

A1.7 GGTGACGGTGGCTGGCTACGACATAGAAAAACAAACAAGTTCAGCACGCTCACACATTGG 1919

N3.9 GGTGACGGTGGCTGGCTACGACATAGAAAAACAAACAAGTTCAGCACGCTCACACATTGG 1919

************************************************************

MG637361.1 ACTCTGCCCTCAACATAACGTACTCTTCAACGAACTCACAGTCAAAGAACATTTACAGTT 1979

A1.9 ACTCTGCCCTCAACATAACGTACTCTTCAACGAACTCACAGTCAAAGAACATTTACAGTT 1979

N1.8 ACTCTGCCCTCAACATAACGTACTCTTCAACGAACTCACAGTCAAAGAACATTTACAGTT 1835

A1.4 ACTCTGCCCTCAACATAACGTACTCTTCAACGAACTCACAGTCAAAGAACATTTACAGTT 1979

A3.1 ACTCTGCCCTCAACATAACGTACTCTTCAACGAACTCACAGTCAAAGAACATTTACAGTT 1979

A3.4 ACTCTGCCCTCAACATAACGTACTCTTCAACGAACTCACAGTCAAAGAACATTTACAGTT 1979

A3.6 ACTCTGCCCTCAACATAACGTACTCTTCAACGAACTCACAGTCAAAGAACATTTACAGTT 1979

A3.7 ACTCTGCCCTCAACATAACGTACTCTTCAACGAACTCACAGTCAAAGAACATTTACAGTT 1979

A3.8 ACTCTGCCCTCAACATAACGTACTCTTCAACGAACTCACAGTCAAAGAACATTTACAGTT 1979

N3.1 ACTCTGCCCTCAACATAACGTACTCTTCAACGAACTCACAGTCAAAGAACATTTACAGTT 1979

N3.6 ACTCTGCCCTCAACATAACGTACTCTTCAACGAACTCACAGTCAAAGAACATTTACAGTT 1979

N3.3 ACTCTGCCCTCAACATAACGTACTCTTCAACGAACTCACAGTCAAAGAACATTTACAGTT 1979

N3.10 ACTCTGCCCTCAACATAACGTACTCTTCAACGAACTCACAGTCAAAGAACATTTACAGTT 1979

A1.3 ACTCTGCCCTCAACATAACGTACTCTTCAACGAACTCACAGTCAAAGAACATTTACAGTT 1834

N1.2 ACTCTGCCCTCAACATAACGTACTCTTCAACGAACTCACAGTCAAAGAACATTTACAGTT 1834

N1.5 ACTCTGCCCTCAACATAACGTACTCTTCAACGAACTCACAGTCAAAGAACATTTACAGTT 1834

N1.27 ACTCTGCCCTCAACATAACGTACTCTTCAACGAACTCACAGTCAAAGAACATTTACAGTT 1834

A1.2 ACTCTGCCCTCAACATAACGTACTCTTCAACGAACTCACAGTCAAAGAACATTTACAGTT 1979

A1.7 ACTCTGCCCTCAACATAACGTACTCTTCAACGAACTCACAGTCAAAGAACATTTACAGTT 1979

N3.9 ACTCTGCCCTCAACATAACGTACTCTTCAACGAACTCACAGTCAAAGAACATTTACAGTT 1979

************************************************************

MG637361.1 CTTCTCTCGTCTGAAAGGCTTCAGCGGTAAAGAGTTGGATGAAGAAATTGAGACGCTTAT 2039

A1.9 CTTCTCTCGTCTGAAAGGCTTCAGCGGTAAAGAGTTGGATGAAGAAATTGTGACGCTTAT 2039

N1.8 CTTCTCTCGTCTGAAAGGCTTCAGCGGTAAAGAGTTGGATGAAGAAATTGAGACGCTTAT 1895

A1.4 CTTCTCTCGTCTGAAAGGCTTCAGCGGTAAAGAGTTGGATGAAGAAATTGTGACGCTTAT 2039

A3.1 CTTCTCTCGTCTGAAAGGCTTCAGCGGTAAAGAGTTGGATGAAGAAATTGAGACGCTTAT 2039

A3.4 CTTCTCTCGTCTGAAAGGCTTCAGCGGTAAAGAGTTGGATGAAGAAATTGAGACGCTTAT 2039

A3.6 CTTCTCTCGTCTGAAAGGCTTCAGCGGTAAAGAGTTGGATGAAGAAATTGAGACGCTTAT 2039

A3.7 CTTCTCTCGTCTGAAAGGCTTCAGCGGTAAAGAGTTGGATGAAGAAATTGAGACGCTTAT 2039

A3.8 CTTCTCTCGTCTGAAAGGCTTCAGCGGTAAAGAGTTGGATGAAGAAATTGAGACGCTTAT 2039

N3.1 CTTCTCTCGTCTGAAAGGCTTCAGCGGTAAAGAGTTGGATGAAGAAATTGAGACGCTTAT 2039

N3.6 CTTCTCTCGTCTGAAAGGCTTCAGCGGTAAAGAGTTGGATGAAGAAATTGAGACGCTTAT 2039

N3.3 CTTCTCTCGTCTGAAAGGCTTCAGCGGTAAAGAGTTGGATGAAGAAATTGAGACGCTTAT 2039

N3.10 CTTCTCTCGTCTGAAAGGCTTCAGCGGTAAAGAGTTGGATGAAGAAATTGAGACGCTTAT 2039

A1.3 CTTCTCTCGTCTGAAAGGCTTCAGCGGTAAAGAGTTGGATGAAGAAATTGAGACGCTTAT 1894

N1.2 CTTCTCTCGTCTGAAAGGCTTCAGCGGTAAAGAGTTGGATGAAGAAATTGAGACGCTTAT 1894

N1.5 CTTCTCTCGTCTGAAAGGCTTCAGCGGTAAAGAGTTGGATGAAGAAATTGAGACGCTTAT 1894

N1.27 CTTCTCTCGTCTGAAAGGCTTCAGCGGTAAAGAGTTGGATGAAGAAATTGAGACGCTTAT 1894

A1.2 CTTCTCTCGTCTGAAAGGCTTCAGCGGTAAAGAGTTGGATGAAGAAATTGTGACGCTTAT 2039

A1.7 CTTCTCTCGTCTGAAAGGCTTCAGCGGTAAAGAGTTGGATGAAGAAATTGTGACGCTTAT 2039

N3.9 CTTCTCTCGTCTGAAAGGCTTCAGCGGTAAAGAGTTGGATGAAGAAATTGTGACGCTTAT 2039

************************************************** *********

MG637361.1 TGAAAAATTGGAATTGCAAGAAAAGAGGGATTACCAATCAGCGGGGTTATCAGGGGGACA 2099

A1.9 TGAAAAATTGGAATTGCAAGAAAAGAGGGATTACCAATCAGCGGGATTATCAGGGGGACA 2099

N1.8 TGAAAAATTGGAATTGCAAGAAAAGAGGGATTACCAATCAGCGGGGTTATCAGGGGGACA 1955

A1.4 TGAAAAATTGGAATTGCAAGAAAAGAGGGATTACCAATCAGCGGGATTATCAGGGGGACA 2099

A3.1 TGAAAAATTGGAATTGCAAGAAAAGAGGGATTACCAATCAGCGGGGTTATCAGGGGGACA 2099

A3.4 TGAAAAATTGGAATTGCAAGAAAAGAGGGATTACCAATCAGCGGGGTTATCAGGGGGACA 2099

A3.6 TGAAAAATTGGAATTGCAAGAAAAGAGGGATTACCAATCAGCGGGGTTATCAGGGGGACA 2099

A3.7 TGAAAAATTGGAATTGCAAGAAAAGAGGGATTACCAATCAGCGGGGTTATCAGGGGGACA 2099

A3.8 TGAAAAATTGGAATTGCAAGAAAAGAGGGATTACCAATCAGCGGGGTTATCAGGGGGACA 2099

N3.1 TGAAAAATTGGAATTGCAAGAAAAGAGGGATTACCAATCAGCGGGGTTATCAGGGGGACA 2099

N3.6 TGAAAAATTGGAATTGCAAGAAAAGAGGGATTACCAATCAGCGGGGTTATCAGGGGGACA 2099

N3.3 TGAAAAATTGGAATTGCAAGAAAAGAGGGATTACCAATCAGCGGGGTTATCAGGGGGACA 2099

N3.10 TGAAAAATTGGAATTGCAAGAAAAGAGGGATTACCAATCAGCGGGGTTATCAGGGGGACA 2099

A1.3 TGAAAAATTGGAATTGCAAGAAAAGAGGGATTACCAATCAGCGGGGTTATCAGGGGGACA 1954

N1.2 TGAAAAATTGGAATTGCAAGAAAAGAGGGATTACCAATCAGCGGGGTTATCAGGGGGACA 1954

N1.5 TGAAAAATTGGAATTGCAAGAAAAGAGGGATTACCAATCAGCGGGGTTATCAGGGGGACA 1954

N1.27 TGAAAAATTGGAATTGCAAGAAAAGAGGGATTACCAATCAGCGGGGTTATCAGGGGGACA 1954

A1.2 TGAAAAATTGGAATTGCAAGAAAAGAGGGATTACCAATCAGCGGGATTATCAGGGGGACA 2099

A1.7 TGAAAAATTGGAATTGCAAGAAAAGAGGGATTACCAATCAGCGGGATTATCAGGGGGACA 2099

N3.9 TGAAAAATTGGAATTGCAAGAAAAGAGGGATTACCAATCAGCGGGATTATCAGGGGGACA 2099

********************************************* **************

MG637361.1 GAAGCGACGATTAGGAGTGGGCGTCGCGCTATGCGGGGCGGCTAAAGTGGTTCTACTGGA 2159

A1.9 GAAGCGACGACTAGGAGTGGGCGTCGCGCTATGCGGGGCGGCTAAAGTGGTTCTACTGGA 2159

N1.8 GAAGCGACGATTAGGAGTGGGCGTCGCGCTATGCGGGGCGGCTAAAGTGGTTCTACTGGA 2015

A1.4 GAAGCGACGATTAGGAGTGGGCGTCGCGCTATGCGGGGCGGCTAAAGTGGTTCTACTGGA 2159

A3.1 GAAGCGACGATTAGGAGTGGGCGTCGCGCTATGCGGGGCGGCTAAAGTGGTTCTACTGGA 2159

A3.4 GAAGCGACGATTAGGAGTGGGCGTCGCGCTATGCGGGGCGGCTAAAGTGGTTCTACTGGA 2159

A3.6 GAAGCGACGATTAGGAGTGGGCGTCGCGCTATGCGGGGCGGCTAAAGTGGTTCTACTGGA 2159

A3.7 GAAGCGACGATTAGGAGTGGGCGTCGCGCTATGCGGGGCGGCTAAAGTGGTTCTACTGGA 2159

A3.8 GAAGCGACGATTAGGAGTGGGCGTCGCGCTATGCGGGGCGGCTAAAGTGGTTCTACTGGA 2159

N3.1 GAAGCGACGATTAGGAGTGGGCGTCGCGCTATGCGGGGCGGCTAAAGTGGTTCTACTGGA 2159

N3.6 GAAGCGACGATTAGGAGTGGGCGTCGCGCTATGCGGGGCGGCTAAAGTGGTTCTACTGGA 2159

N3.3 GAAGCGACGATTAGGAGTGGGCGTCGCGCTATGCGGGGCGGCTAAAGTGGTTCTACTGGA 2159

N3.10 GAAGCGACGATTAGGAGTGGGCGTCGCGCTATGCGGGGCGGCTAAAGTGGTTCTACTGGA 2159

A1.3 GAAGCGACGATTAGGAGTGGGCGTCGCGCTATGCGGGGCGGCTAAAGTGGTTCTACTGGA 2014

N1.2 GAAGCGACGATTAGGAGTGGGCGTCGCGCTATGCGGGGCGGCTAAAGTGGTTCTACTGGA 2014

N1.5 GAAGCGACGATTAGGAGTGGGCGTCGCGCTATGCGGGGCGGCTAAAGTGGTTCTACTGGA 2014

N1.27 GAAGCGACGATTAGGAGTGGGCGTCGCGCTATGCGGGGCGGCTAAAGTGGTTCTACTGGA 2014

A1.2 GAAGCGACGACTAGGAGTGGGCGTCGCGCTATGCGGGGCGGCTAAAGTGGTTCTACTGGA 2159

A1.7 GAAGCGACGACTAGGAGTGGGCGTCGCGCTATGCGGGGCGGCTAAAGTGGTTCTACTGGA 2159

N3.9 GAAGCGACGACTAGGAGTGGGCGTCGCGCTATGCGGGGCGGCTAAAGTGGTTCTACTGGA 2159

********** *************************************************

MG637361.1 CGAGCCCACTTCTGGCATGGACCCGGCCTCACGTCGTGCCCTATGGGACTTGTTGCAGAG 2219

A1.9 CGAGCCCACTTCTGGCATGGACCCGGCCTCACGTCGTGCCCTATGGGACTTGTTGCAGAG 2219

N1.8 CGAGCCCACTTCTGGCATGGACCCGGCCTCACGTCGTGCCCTATGGGACTTGTTGCAGAG 2075

A1.4 CGAGCCCACTTCTGGCATGGACCCGGCCTCACGTCGTGCCCTATGGGACTTGTTGCAGAG 2219

A3.1 CGAGCCCACTTCTGGCATGGACCCGGCCTCACGTCGTGCCCTATGGGACTTGTTGCAGAG 2219

A3.4 CGAGCCCACTTCTGGCATGGACCCGGCCTCACGTCGTGCCCTATGGGACTTGTTGCAGAG 2219

A3.6 CGAGCCCACTTCTGGCATGGACCCGGCCTCACGTCGTGCCCTATGGGACTTGTTGCAGAG 2219

A3.7 CGAGCCCACTTCTGGCATGGACCCGGCCTCACGTCGTGCCCTATGGGACTTGTTGCAGAG 2219

A3.8 CGAGCCCACTTCTGGCATGGACCCGGCCTCACGTCGTGCCCTATGGGACTTGTTGCAGAG 2219

N3.1 CGAGCCCACTTCTGGCATGGACCCGGCCTCACGTCGTGCCCTATGGGACTTGTTACAGAG 2219

N3.6 CGAGCCCACTTCTGGCATGGACCCGGCCTCACGTCGTGCCCTATGGGACTTGTTACAGAG 2219

N3.3 CGAGCCCACTTCTGGCATGGACCCGGCCTCACGTCGTGCCCTATGGGACTTGTTGCAGAG 2219

N3.10 CGAGCCCACTTCTGGCATGGACCCGGCCTCACGTCGTGCCCTATGGGACTTGTTGCAGAG 2219

A1.3 CGAGCCCACTTCTGGCATGGACCCGGCCTCACGTCGTGCCCTATGGGACTTGTTGCAGAG 2074

N1.2 CGAGCCCACTTCTGGCATGGACCCGGCCTCACGTCGTGCCCTATGGGACTTGTTGCAGAG 2074

N1.5 CGAGCCCACTTCTGGCATGGACCCGGCCTCACGTCGTGCCCTATGGGACTTGTTGCAGAG 2074

N1.27 CGAGCCCACTTCTGGCATGGACCCGGCCTCACGTCGTGCCCTATGGGACTTGTTGCAGAG 2074

A1.2 CGAGCCCACTTCTGGCATGGACCCGGCCTCACGTCGTGCCCTGTGGGACTTGTTGCAGAG 2219

A1.7 CGAGCCCACTTCTGGCATGGACCCGGCCTCACGTCGTGCCCTGTGGGACTTGTTGCAGAG 2219

N3.9 CGAGCCCACTTCTGGCATGGACCCGGCCTCACGTCGTGCCCTGTGGGACTTGTTGCAGAG 2219

****************************************** *********** *****

MG637361.1 AGAGAAGAAAGGTCGATCGATGATCCTGACGACACACTTCATGGACGAAGCGGACATATT 2279

A1.9 AGAGAAGAAAG------------------------------------------------- 2230

N1.8 AGAGAAGAAAGGTCGATCGATGATCCTGACGACACACTTCATGGACGAAGCGGACATATT 2135

A1.4 AGAGAAGAAAGGTCGATCGATGATCCTGACGACACACTTCATGGACGAAGCGGACATATT 2279

A3.1 AGAGAAGAAAGGTCGATCGATGATCCTGACGACACACTTCATGGACGAAGCGGACATATT 2279

A3.4 AGAGAAGAAAGGTCGATCGATGATCCTGACGACACACTTCATGGACGAAGCGGACATATT 2279

A3.6 AGAGAAGAAAGGTCGATCGATGATCCTGACGACACACTTCATGGACGAAGCGGACATATT 2279

A3.7 AGAGAAGAAAGGTCGATCGATGATCCTGACGACACACTTCATGGACGAAGCGGACATATT 2279

A3.8 AGAGAAGAAAGGTCGATCGATGATCCTGACGACACACTTCATGGACGAAGCGGACATATT 2279

N3.1 AGAGAAGAAAGGTCGATCGATGATCCTGACGACACACTTCATGGACGAAGCGGACATATT 2279

N3.6 AGAGAAGAAAGGTCGATCGATGATCCTGACGACACACTTCATGGACGAAGCGGACATATT 2279

N3.3 AGAGAAGAAAGGTCGATCGATGATCCTGACGACACACTTCATGGACGAAGCGGACATATT 2279

N3.10 AGAGAAGAAAGGTCGATCGATGATCCTGACGACACACTTCATGGACGAAGCGGACATATT 2279

A1.3 AGAGAAGAAAGGTCGATCGATGATCCTGACGACACACTTCATGGACGAAGCGGACATATT 2134

N1.2 AGAGAAGAAAGGTCGATCGATGATCCTGACGACACACTTCATGGACGAAGCGGACATATT 2134

N1.5 AGAGAAGAAAGGTCGATCGATGATCCTGACGACACACTTCATGGACGAAGCGGACATATT 2134

N1.27 AGAGAAGAAAGGTCGATCGATGATCCTGACGACACACTTCATGGACGAAGCGGACATATT 2134

A1.2 AGAGAAGAAAGGTCGATCGATGATCCTGACGACACACTTCATGGACGAAGCGGACATATT 2279

A1.7 AGAGAAGAAAGGTCGATCGATGATCCTGACGACACACTTCATGGACGAAGCGGACATATT 2279

N3.9 AGAGAAGAAAGGTCGATCGATGATCCTGACGACACACTTCATGGACGAAGCGGACATATT 2279

*******

MG637361.1 AGGGGATAGAGTTGCCATTATGGCGGACGGTCGTCTCCAGTGCGTGGGCTCACCTTACTT 2339

A1.9 ------------------------------------------------------------ 2230

N1.8 AGGGGATAGAGTTGCCATTATGGCGGACGGTCGTCTCCAGTGCGTGGGCTCACCTTACTT 2195

A1.4 AGGGGATAGAGTTGCCATTATGGCGGACGGTCGTCTCCAGTGCGTGGGTTCACCTTACTT 2339

A3.1 AGGGGATAGAGTTGCCATTATGGCGGACGGTCGTCTCCAGTGCGTGGGCTCACCTTACTT 2339

A3.4 AGGGGATAGAGTTGCCATTATGGCGGACGGTCGTCTCCAGTGCGTGGGCTCACCTTACTT 2339

A3.6 AGGGGATAGAGTTGCCATTATGGCGGACGGTCGTCTCCAGTGCGTGGGCTCACCTTACTT 2339

A3.7 AGGGGATAGAGTTGCCATTATGGCGGACGGTCGTCTCCAGTGCGTGGGCTCACCTTACTT 2339

A3.8 AGGGGATAGAGTTGCCATTATGGCGGACGGTCGTCTCCAGTGCGTGGGCTCACCTTACTT 2339

N3.1 AGGGGATAGAGTTGCCATTATGGCGGACGGTCGTCTCCAGTGCGTGGGCTCACCTTACTT 2339

N3.6 AGGGGATAGAGTTGCCATTATGGCGGACGGTCGTCTCCAGTGCGTGGGCTCACCTTACTT 2339

N3.3 AGGGGATAGAGTTGCCATTATGGCGGACGGTCGTCTCCAGTGCGTGGGCTCACCTTACTT 2339

N3.10 AGGGGATAGAGTTGCCATTATGGCGGACGGTCGTCTCCAGTGCGTGGGCTCACCTTACTT 2339

A1.3 AGGGGATAGAGTTGCCATTATGGCGGACGGTCGTCTCCAGTGCGTGGGCTCACCTTACTT 2194

N1.2 AGGGGATAGAGTTGCCATTATGGCGGACGGTCGTCTCCAGTGCGTGGGCTCACCTTACTT 2194

N1.5 AGGGGATAGAGTTGCCATTATGGCGGACGGTCGTCTCCAGTGCGTGGGCTCACCTTACTT 2194

N1.27 AGGGGATAGAGTTGCCATTATGGCGGACGGTCGTCTCCAGTGCGTGGGCTCACCTTACTT 2194

A1.2 AGGGGATAGAGTTGCCATTATGGCGGACGGTCGTCTCCAGTGCGTGGGCTCACCTTACTT 2339

A1.7 AGGGGATAGAGTTGCCATTATGGCGGACGGTCGTCTCCAGTGCGTGGGCTCACCTTACTT 2339

N3.9 AGGGGATAGAGTTGCCATTATGGCGGACGGTCGTCTCCAGTGCGTGGGCTCACCTTACTT 2339

MG637361.1 CCTCAAGAGACACTATGGAGTCGGCTACACGCTAGTTGTGGTCAAGAAGGAAGATTTCCG 2399

A1.9 ------------------------------------------------------------ 2230

N1.8 CCTCAAGAGACACTATGGAGTCGGCTACACGCTAGTTGTGGTCAAGAAGGAAGATTTCCG 2255

A1.4 CCTCAAGAGACACTATGGAGTCGGCTACACGCTAGTTGTGGTCAAGAAGGAAGATTTCCG 2399

A3.1 CCTCAAGAGACACTATGGAGTCGGCTACACGCTAGTTGTGGTCAAGAAGGAAGATTTCCG 2399

A3.4 CCTCAAGAGACACTATGGAGTCGGCTACACGCTAGTTGTGGTCAAGAAGGAAGATTTCCG 2399

A3.6 CCTCAAGAGACACTATGGAGTCGGCTACACGCTAGTTGTGGTCAAGAAGGAAGATTTCCG 2399

A3.7 CCTCAAGAGACACTATGGAGTCGGCTACACGCTAGTTGTGGTCAAGAAGGAAGATTTCCG 2399

A3.8 CCTCAAGAGACACTATGGAGTCGGCTACACGCTAGTTGTGGTCAAGAAGGAAGATTTCCG 2399

N3.1 CCTCAAGAGACACTATGGAGTCGGCTACACGCTAGTTGTGGTCAAGAAGGAAGATTTCCG 2399

N3.6 CCTCAAGAGACACTATGGAGTCGGCTACACGCTAGTTGTGGTCAAGAAGGAAGATTTCCG 2399

N3.3 CCTCAAGAGACACTATGGAGTCGGCTACACGCTAGTTGTGGTCAAGAAGGAAGATTTCCG 2399

N3.10 CCTCAAGAGACACTATGGAGTCGGCTACACGCTAGTTGTGGTCAAGAAGGAAGATTTCCG 2399

A1.3 CCTCAAGAGACACTATGGAGTCGGCTACACGCTAGTTGTGGTCAAGAAGGAAGATTTCCG 2254

N1.2 CCTCAAGAGACACTATGGAGTCGGCTACACGCTAGTTGTGGTCAAGAAGGAAGATTTCCG 2254

N1.5 CCTCAAGAGACACTATGGAGTCGGCTACACGCTAGTTGTGGTCAAGAAGGAAGATTTCCG 2254

N1.27 CCTCAAGAGACACTATGGAGTCGGCTACACGCTAGTTGTGGTCAAGAAGGAAGATTTCCG 2254

A1.2 CCTCAAGAGACACTATGGAGTCGGCTACACGCTAGTTGTGGTCAAGAAGGAAGATTTCCG 2399

A1.7 CCTCAAGAGACACTATGGAGTCGGCTACACGCTAGTTGTGGTCAAGAAGGAAGATTTCCG 2399

N3.9 CCTCAAGAGACACTATGGAGTCGGCTACACGCTAGTTGTGGTCAAGAAGGAAGATTTCCG 2399

MG637361.1 ACTGGACACCTGCACAGAGCTGATCAATAGATACATCCCTGGAACTGTTGTGAAGGAAGA 2459

A1.9 ------------------------------------------------------------ 2230

N1.8 ACTGGACACCTGCACAGAGCTGATCAATAGATACATCCCTGGAACTGTTGTGAAGGAAGA 2315

A1.4 ACTGGACACCTGCACAGAGCTGATCAATAGATACATCCCTGGAACTGTTGTGAAGGAAGA 2459

A3.1 ACTGGACACCTGCACAGAGCTGATCAATAGATACATCCCTGGAACTGTTGTGAAGGAAGA 2459

A3.4 ACTGGACACCTGCACAGAGCTGATCAATAGATACATCCCTGGAACTGTTGTGAAGGAAGA 2459

A3.6 ACTGGACACCTGCACAGAGCTGATCAATAGATACATCCCTGGAACTGTTGTGAAGGAAGA 2459

A3.7 ACTGGACACCTGCACAGAGCTGATCAATAGATACATCCCTGGAACTGTTGTGAAGGAAGA 2459

A3.8 ACTGGACACCTGCACAGAGCTGATCAATAGATACATCCCTGGAACTGTTGTGAAGGAAGA 2459

N3.1 ACTGGACACCTGCACAGAGCTGATCAATAGATACATCCCTGGAACTGTTGTGAAGGAAGA 2459

N3.6 ACTGGACACCTGCACAGAGCTGATCAATAGATACATCCCTGGAACTGTTGTGAAGGAAGA 2459

N3.3 ACTGGACACCTGCACAGAGCTGATCAATAGATACATCCCTGGAACTGTTGTGAAGGAAGA 2459

N3.10 ACTGGACACCTGCACAGAGCTGATCAATAGATACATCCCTGGAACTGTTGTGAAGGAAGA 2459

A1.3 ACTGGACACCTGCACAGAGCTGATCAATAGATACATCCCTGGAACTGTTGTGAAGGAAGA 2314

N1.2 ACTGGACACCTGCACAGAGCTGATCAATAGATACATCCCTGGAACTGTTGTGAAGGAAGA 2314

N1.5 ACTGGACACCTGCACAGAGCTGATCAATAGATACATCCCTGGAACTGTTGTGAAGGAAGA 2314

N1.27 ACTGGACACCTGCACAGAGCTGATCAATAGATACATCCCTGGAACTGTTGTGAAGGAAGA 2314

A1.2 ACTGGACACCTGCACAGAGCTGATCAATAGATACATCCCTGGAACTGTTGTGAAGGAAGA 2459

A1.7 ACTGGACACCTGCACAGAGCTGATCAATAGATACATCCCTGGAACTGTTGTGAAGGAAGA 2459

N3.9 ACTGGACACCTGCACAGAGCTGATCAATAGATACATCCCTGGAACTGTTGTGAAGGAAGA 2459

MG637361.1 CCGAGGCACTGAAGTGACATATAGCATGACTAATGAGTATTCGCACGTGTTTGAATCTAT 2519

A1.9 ------------------------------------------------------------ 2230

N1.8 CCGAGGCACTGAAGTGACATATAGCATGACTAATGAGTATTCGCACGTGTTTGAATCTAT 2375

A1.4 CCGAGGCACTGAAGTGACATATAGCATGACTAATGAGTATTCGCACGTGTTTGAATCTAT 2519

A3.1 CCGAGGCACTGAAGTGACATATAGCATGACTAATGAGTATTCGCACGTGTTTGAATCTAT 2519

A3.4 CCGAGGCACTGAAGTGACATATAGCATGACTAATGAGTATTCGCACGTGTTTGAATCTAT 2519

A3.6 CCGAGGCACTGAAGTGACATATAGCATGACTAATGAGTATTCGCACGTGTTTGAATCTAT 2519

A3.7 CCGAGGCACTGAAGTGACATATAGCATGACTAATGAGTATTCGCACGTGTTTGAATCTAT 2519

A3.8 CCGAGGCACTGAAGTGACATATAGCATGACTAATGAGTATTCGCACGTGTTTGAATCTAT 2519

N3.1 CCGAGGCACTGAAGTGACATATAGCATGACTAATGAGTATTCGCACGTGTTTGAATCTAT 2519

N3.6 CCGAGGCACTGAAGTGACATATAGCATGACTAATGAGTATTCGCACGTGTTTGAATCTAT 2519

N3.3 CCGAGGCACTGAAGTGACATATAGCATGACTAATGAGTATTCGCACGTGTTTGAATCTAT 2519

N3.10 CCGAGGCACTGAAGTGACATATAGCATGACTAATGAGTATTCGCACGTGTTTGAATCTAT 2519

A1.3 CCGAGGCACTGAAGTGACATATAGCATGACTAATGAGTATTCGCACGTGTTTGAATCTAT 2374

N1.2 CCGAGGCACTGAAGTGACATATAGCATGACTAATGAGTATTCGCACGTGTTTGAATCTAT 2374

N1.5 CCGAGGCACTGAAGTGACATATAGCATGACTAATGAGTATTCGCACGTGTTTGAATCTAT 2374

N1.27 CCGAGGCACTGAAGTGACATATAGCATGACTAATGAGTATTCGCACGTGTTTGAATCTAT 2374

A1.2 CCGAGGCACTGAAGTGACATATAGCATGACTAATGAGTATTCGCACGTGTTTGAATCTAT 2519

A1.7 CCGAGGCACTGAAGTGACATATAGCATGACTAATGAGTATTCGCACGTGTTTGAATCTAT 2519

N3.9 CCGAGGCACTGAAGTGACATATAGCATGACTAATGAGTATTCGCACGTGTTTGAATCTAT 2519

MG637361.1 GCTGCGCGATTTGGAGGCAAAGGCCGATGAGATAAACTTTAAAAACTACGGCCTACTGGC 2579

A1.9 ------------------------------------------------------------ 2230

N1.8 GCTGCGCGATTTGGAGGCAAAGGCCGATGAGATAAACTTTAAAAACTACGGCCTACTGGC 2435

A1.4 GCTGCGCGATTTGGAGGCAAAGGCCGATGAGATAAACTTTAAAAACTACGGCCTACTGGC 2579

A3.1 GCTGCGCGATTTGGAGGCAAAGGCCGATGAGATAAACTTTAAAAACTACGGCCTACTGGC 2579

A3.4 GCTGCGCGATTTGGAGGCAAAGGCCGATGAGATAAACTTTAAAAACTACGGCCTACTGGC 2579

A3.6 GCTGCGCGATTTGGAGGCAAAGGCCGATGAGATAAACTTTAAAAACTACGGCCTACTGGC 2579

A3.7 GCTGCGCGATTTGGAGGCAAAGGCCGATGAGATAAACTTTAAAAACTACGGCCTACTGGC 2579

A3.8 GCTGCGCGATTTGGAGGCAAAGGCCGATGAGATAAACTTTAAAAACTACGGCCTACTGGC 2579

N3.1 GCTGCGCGATTTGGAGGCAAAGGCCGATGAGATAAACTTTAAAAACTACGGCCTACTGGC 2579

N3.6 GCTGCGCGATTTGGAGGCAAAGGCCGATGAGATAAACTTTAAAAACTACGGCCTACTGGC 2579

N3.3 GCTGCGCGATTTGGAGGCAAAGGCCGATGAGATAAACTTTAAAAACTACGGCCTACTGGC 2579

N3.10 GCTGCGCGATTTGGAGGCAAAGGCCGATGAGATAAACTTTAAAAACTACGGCCTACTGGC 2579

A1.3 GCTGCGCGATTTGGAGGCAAAGGCCGATGAGATAAACTTTAAAAACTACGGCCTACTGGC 2434

N1.2 GCTGCGCGATTTGGAGGCAAAGGCCGATGAGATAAACTTTAAAAACTACGGCCTACTGGC 2434

N1.5 GCTGCGCGATTTGGAGGCAAAGGCCGATGAGATAAACTTTAAAAACTACGGCCTACTGGC 2434

N1.27 GCTGCGCGATTTGGAGGCAAAGGCCGATGAGATAAACTTTAAAAACTACGGCCTACTGGC 2434

A1.2 GCTGCGCGATTTGGAGGCAAAAGCCGATGAGATAAACTTTAAAAACTACGGCCTACTGGC 2579

A1.7 GCTGCGCGATTTGGAGGCAAAAGCCGATGAGATAAACTTTAAAAACTACGGCCTACTGGC 2579

N3.9 GCTGCGCGATTTGGAGGCAAAAGCCGATGAGATAAACTTTAAAAACTACGGCCTACTGGC 2579

MG637361.1 TACTACATTAGAAGATGTGTTCATGTCCGTGGGCACAGATGTGGTCGCAACTTCAGATGT 2639

A1.9 ------------------------------------------------------------ 2230

N1.8 TACTACATTAGAAGATGTGTTCATGTCCGTGGGCACAGATGTGGTCGCAACTTCAGATGT 2495

A1.4 TACTACATTAGAAGATGTGTTCATGTCCGTGGGCACAGATGTGGTCGCAACTTCAGATGT 2639

A3.1 TACTACATTAGAAGATGTGTTCATGTCCGTGGGCACAGATGTGGTCGCAACTTCAGATGT 2639

A3.4 TACTACATTAGAAGATGTGTTCATGTCCGTGGGCACAGATGTGGTCGCAACTTCAGATGT 2639

A3.6 TACTACATTAGAAGATGTGTTCATGTCCGTGGGCACAGATGTGGTCGCAACTTCAGATGT 2639

A3.7 TACTACATTAGAAGATGTGTTCATGTCCGTGGGCACAGATGTGGTCGCAACTTCAGATGT 2639

A3.8 TACTACATTAGAAGATGTGTTCATGTCCGTGGGCACAGATGTGGTCGCAACTTCAGATGT 2639

N3.1 TACTACATTAGAAGATGTGTTCATGTCCGTGGGCACAGATGTGGTCGCAACTTCAGATGT 2639

N3.6 TACTACATTAGAAGATGTGTTCATGTCCGTGGGCACAGATGTGGTCGCAACTTCAGATGT 2639

N3.3 TACTACATTAGAAGATGTGTTCATGTCCGTGGGCACAGATGTGGTCGCAACTTCAGATGT 2639

N3.10 TACTACATTAGAAGATGTGTTCATGTCCGTGGGCACAGATGTGGTCGCAACTTCAGATGT 2639

A1.3 TACTACATTAGAAGATGTGTTCATGTCCGTGGGCACAGATGTGGTCGCAACTTCAGATGT 2494

N1.2 TACTACATTAGAAGATGTGTTCATGTCCGTGGGCACAGATGTGGTCGCAACTTCAGATGT 2494

N1.5 TACTACATTAGAAGATGTGTTCATGTCCGTGGGCACAGATGTGGTCGCAACTTCAGATGT 2494

N1.27 TACTACATTAGAAGATGTGTTCATGTCCGTGGGCACAGATGTGGTCGCAACTTCAGATGT 2494

A1.2 TACTACATTAGAAGATGTGTTCATGTCCGTGGGCACAGATGTGGTCGCAACTTCAGATGT 2639

A1.7 TACTACATTAGAAGATGTGTTCATGTCCGTGGGCACAGATGTGGTCGCAACTTCAGATGT 2639

N3.9 TACTACATTAGAAGATGTGTTCATGTCCGTGGGCACAGATGTGGTCGCAACTTCAGATGT 2639

MG637361.1 GGACGACAATACAACCGTTTCATCTAGTGCTGATACTCTAGCATTTGAATATGATTCTTT 2699

A1.9 ------------------------------------------------------------ 2230

N1.8 GGACGACAATACAACCGTTTCATCTAGTGCTGATACTCTAGCATTTGAATATGATTCTTT 2555

A1.4 GGACGACAATACAACCGTTTCATCTAGTGCTGATACTCTAGCATTTGAATATGATTCTTT 2699

A3.1 GGACGACAATACAACCGTTTCATCTAGTGCTGATACTCTAGCATTTGAATATGATTCTTT 2699

A3.4 GGACGACAATACAACCGTTTCATCTAGTGCTGATACTCTAGCATTTGAATATGATTCTTT 2699

A3.6 GGACGACAATACAACCGTTTCATCTAGTGCTGATACTCTAGCATTTGAATATGATTCTTT 2699

A3.7 GGACGACAATACAACCGTTTCATCTAGTGCTGATACTCTAGCATTTGAATATGATTCTTT 2699

A3.8 GGACGACAATACAACCGTTTCATCTAGTGCTGATACTCTAGCATTTGAATATGATTCTTT 2699

N3.1 GGACGACAATACAACCGTTTCATCTAGTGCTGATACTCTAGCATTTGAATATGATTCTTT 2699

N3.6 GGACGACAATACAACCGTTTCATCTAGTGCTGATACTCTAGCATTTGAATATGATTCTTT 2699

N3.3 GGACGACAATACAACCGTTTCATCTAGTGCTGATACTCTAGCATTTGAATATGATTCTTT 2699

N3.10 GGACGACAATACAACCGTTTCATCTAGTGCTGATACTCTAGCATTTGAATATGATTCTTT 2699

A1.3 GGACGACAATACAACCGTTTCATCTAGTGCTGATACTCTAGCATTTGAATATGATTCTTT 2554

N1.2 GGACGACAATACAACCGTTTCATCTAGTGCTGATACTCTAGCATTTGAATATGATTCTTT 2554

N1.5 GGACGACAATACAACCGTTTCATCTAGTGCTGATACTCTAGCATTTGAATATGATTCTTT 2554

N1.27 GGACGACAATACAACCGTTTCATCTAGTGCTGATACTCTAGCATTTGAATATGATTCTTT 2554

A1.2 GGACGACAATACAACCGTTTCATCTAGTGCTGATACTCTAGCATTTGAATATGATTCTTT 2699

A1.7 GGACGACAATACAACCGTTTCATCTAGTGCTGATACTCTAGCATTTGAATATGATTCTTT 2699

N3.9 GGACGACAATACAACCGTTTCATCTAGTGCTGATACTCTAGCATTTGAATATGATTCTTT 2699

MG637361.1 AGAAAAATTGGACGGGACTGGCTATGGGGATGAAAAAGGGATCCGATTAATTTGCCAACA 2759

A1.9 ------------------------------------------------------------ 2230

N1.8 AGAAAAATTGGACGGGACTGGCTATGGGGATGAAAAAGGGATCCGATTAATTTGCCAACA 2615

A1.4 AGAAAAATTGGACGGGACTGGCTATGGGGATGAAAAAGGGATCCGATTAATTTGCCAACA 2759

A3.1 AGAAAAATTGGACGGGACTGGCTATGGGGATGAAAAAGGGATCCGATTAATTTGCCAACA 2759

A3.4 AGAAAAATTGGACGGGACTGGCTATGGGGATGAAAAAGGGATCCGATTAATTTGCCAACA 2759

A3.6 AGAAAAATTGGACGGGACTGGCTATGGGGATGAAAAAGGGATCCGATTAATTTGCCAACA 2759

A3.7 AGAAAAATTGGACGGGACTGGCTATGGGGATGAAAAAGGGATCCGATTAATTTGCCAACA 2759

A3.8 AGAAAAATTGGACGGGACTGGCTATGGGGATGAAAAAGGGATCCGATTAATTTGCCAACA 2759

N3.1 AGAAAAATTGGACGGGACTGGCTATGGGGATGAAAAAGGGATCCGATTAATTTGCCAACA 2759

N3.6 AGAAAAATTGGACGGGACTGGCTATGGGGATGAAAAAGGGATCCGATTAATTTGCCAACA 2759

N3.3 AGAAAAATTGGACGGGACTGGCTATGGGGATGAAAAAGGGATCCGATTAATTTGCCAACA 2759

N3.10 AGAAAAATTGGACGGGACTGGCTATGGGGATGAAAAAGGGATCCGATTAATTTGCCAACA 2759

A1.3 AGAAAAATTGGACGGGACTGGCTATGGGGATGAAAAAGGGATCCGATTAATTTGCCAACA 2614

N1.2 AGAAAAATTGGACGGGACTGGCTATGGGGATGAAAAAGGGATCCGATTAATTTGCCAACA 2614

N1.5 AGAAAAATTGGACGGGACTGGCTATGGGGATGAAAAAGGGATCCGATTAATTTGCCAACA 2614

N1.27 AGAAAAATTGGACGGGACTGGCTATGGGGATGAAAAAGGGATCCGATTAATTTGCCAACA 2614

A1.2 AGAAAAATTGGACGGGACTGGCTATGGGGATGAAAAAGGGATCCGATTAATTTCCCAACA 2759

A1.7 AGAAAAATTGGACGGGACTGGCTATGGGGATGAAAAAGGGATCCGATTAATTTCCCAACA 2759

N3.9 AGAAAAATTGGACGGGACTGGCTATGGGGATGAAAAAGGGATCCGATTAATTTCCCAACA 2759

MG637361.1 CGTGGTAGCAATATGGATGAAACTGTTTCTGGTGCTGACAAGGTCTTGGCTTATCCTGTT 2819

A1.9 ------------------------------------------------------------ 2230

N1.8 CGTGGTAGCAATATGGATGAAACTGTTTCTGGTGCTGACAAGGTCTTGGCTTATCCTGTT 2675

A1.4 CGTGGTAGCAATATGGATGAAACTGTTTCTGGTGCTGACAAGGTCTTGGCTTATCCTGTT 2819

A3.1 CGTGGTAGCAATATGGATGAAACTGTTTCTGGTGCTGACAAGGTCTTGGCTTATCCTGTT 2819

A3.4 CGTGGTAGCAATATGGATGAAACTGTTTCTGGTGCTGACAAGGTCTTGGCTTATCCTGTT 2819

A3.6 CGTGGTAGCAATATGGATGAAACTGTTTCTGGTGCTGACAAGGTCTTGGCTTATCCTGTT 2819

A3.7 CGTGGTAGCAATATGGATGAAACTGTTTCTGGTGCTGACAAGGTCTTGGCTTATCCTGTT 2819

A3.8 CGTGGTAGCAATATGGATGAAACTGTTTCTGGTGCTGACAAGGTCTTGGCTTATCCTGTT 2819

N3.1 CGTGGTAGCAATATGGATGAAACTGTTTCTGGTGCTGACAAGGTCTTGGCTTATCCTGTT 2819

N3.6 CGTGGTAGCAATATGGATGAAACTGTTTCTGGTGCTGACAAGGTCTTGGCTTATCCTGTT 2819

N3.3 CGTGGTAGCAATATGGATGAAACTGTTTCTGGTGCTGACAAGGTCTTGGCTTATCCTGTT 2819

N3.10 CGTGGTAGCAATATGGATGAAACTGTTTCTGGTGCTGACAAGGTCTTGGCTTATCCTGTT 2819

A1.3 CGTGGTAGCAATATGGATGAAACTGTTTCTGGTGCTGACAAGGTCTTGGCTTATCCTGTT 2674

N1.2 CGTGGTAGCAATATGGATGAAACTGTTTCTGGTGCTGACAAGGTCTTGGCTTATCCTGTT 2674

N1.5 CGTGGTAGCAATATGGATGAAACTGTTTCTGGTGCTGACAAGGTCTTGGCTTATCCTGTT 2674

N1.27 CGTGGTAGCAATATGGATGAAACTGTTTCTGGTGCTGACAAGGTCTTGGCTTATCCTGTT 2674

A1.2 CGTGGTAGCAATATGGATGAAACTGTTTCTGGTGCTGACAAGGTCTTGGCTTATCCTGTT 2819

A1.7 CGTGGTAGCAATATGGATGAAACTGTTTCTGGTGCTGACAAGGTCTTGGCTTATCCTGTT 2819

N3.9 CGTGGTAGCAATATGGATGAAACTGTTTCTGGTGCTGACAAGGTCTTGGCTTATCCTGTT 2819

MG637361.1 GCTCCAAGTATTGGTGTCCTTGGTACAAATCATTGCCACACTCGGAGTCATGCAGTATGT 2879

A1.9 ------------------------------------------------------------ 2230

N1.8 GCTCCAAGTATTGGTGTCCTTGGTACAAATCATTGCCACACTCGGAGTCATGCAGTATGT 2735

A1.4 GCTCCAAGTATTGGTGTCCTTGGTACAAATCATTGCCACACTCGGAGTCATGCAGTATGT 2879

A3.1 GCTCCAAGTATTGGTGTCCTTGGTACAAATCATTGCCACACTCGGAGTCATGCAGTATGT 2879

A3.4 GCTCCAAGTATTGGTGTCCTTGGTACAAATCATTGCCACACTCGGAGTCATGCAGTATGT 2879

A3.6 GCTCCAAGTATTGGTGTCCTTGGTACAAATCATTGCCACACTCGGAGTCATGCAGTATGT 2879

A3.7 GCTCCAAGTATTGGTGTCCTTGGTACAAATCATTGCCACACTCGGAGTCATGCAGTATGT 2879

A3.8 GCTCCAAGTATTGGTGTCCTTGGTACAAATCATTGCCACACTCGGAGTCATGCAGTATGT 2879

N3.1 GCTCCAAGTATTGGTGTCCTTGGTACAAATCATTGCCACACTCGGAGTCATGCAGTATGT 2879

N3.6 GCTCCAAGTATTGGTGTCCTTGGTACAAATCATTGCCACACTCGGAGTCATGCAGTATGT 2879

N3.3 GCTCCAAGTATTGGTGTCCTTGGTACAAATCATTGCCACACTCGGAGTCATGCAGTATGT 2879

N3.10 GCTCCAAGTATTGGTGTCCTTGGTACAAATCATTGCCACACTCGGAGTCATGCAGTATGT 2879

A1.3 GCTCCAAGTATTGGTGTCCTTGGTACAAATCATTGCCACACTCGGAGTCATGCAGTATGT 2734

N1.2 GCTCCAAGTATTGGTGTCCTTGGTACAAATCATTGCCACACTCGGAGTCATGCAGTATGT 2734

N1.5 GCTCCAAGTATTGGTGTCCTTGGTACAAATCATTGCCACACTCGGAGTCATGCAGTATGT 2734

N1.27 GCTCCAAGTATTGGTGTCCTTGGTACAAATCATTGCCACACTCGGAGTCATGCAGTATGT 2734

A1.2 GCTCCAAGTATTGGTGTCCTTGGTACAAATCATTGCCACACTCGGAGTCATGCAGTATGT 2879

A1.7 GCTCCAAGTATTGGTGTCCTTGGTACAAATCATTGCCACACTCGGAGTCATGCAGTATGT 2879

N3.9 GCTCCAAGTATTGGTGTCCTTGGTACAAATCATTGCCACACTCGGAGTCATGCAGTATGT 2879

MG637361.1 CATCTCTATGACCGAGCATATACAAAGAAGAGAACTTTCATTGGCTGAAGGTTTCGCAGG 2939

A1.9 ---------------------------------------------------TTTCGCAGG 2239

N1.8 CATCTCTATGACCGAGCATATACAAAGAAGAGAACTTTCATTGGCTGAAGGTTTCGCAGG 2795

A1.4 CATCTCTATGACCGAGCATATACAAAGAAGAGAACTTTCATTGGCTGAAGGTTTCGCAGG 2939

A3.1 CATCTCTATGACCGAGCATATACAAAGAAGAGAACTTTCATTGGCTGAAGGTTTCGCAGG 2939

A3.4 CATCTCTATGACCGAGCATATACAAAGAAGAGAACTTTCATTGGCTGAAGGTTTCGCAGG 2939

A3.6 CATCTCTATGACCGAGCATATACAAAGAAGAGAACTTTCATTGGCTGAAGGTTTCGCAGG 2939

A3.7 CATCTCTATGACCGAGCATATACAAAGAAGAGAACTTTCATTGGCTGAAGGTTTCGCAGG 2939

A3.8 CATCTCTATGACCGAGCATATACAAAGAAGAGAACTTTCATTGGCTGAAGGTTTCGCAGG 2939

N3.1 CATCTCTATGACCGAGCATATACAAAGAAGAGAACTTTCATTGGCTGAAGGTTTCGCAGG 2939

N3.6 CATCTCTATGACCGAGCATATACAAAGAAGAGAACTTTCATTGGCTGAAGGTTTCGCAGG 2939

N3.3 CATCTCTATGACCGAGCATATACAAAGAAGAGAACTTTCATTGGCTGAAGGTTTCGCAGG 2939

N3.10 CATCTCTATGACCGAGCATATACAAAGAAGAGAACTTTCATTGGCTGAAGGTTTCGCAGG 2939

A1.3 CATCTCTATGACCGAGCATATACAAAGAAGAGAACTTTCATTGGCTGAAGGTTTCGCAGG 2794

N1.2 CATCTCTATGACCGAGCATATACAAAGAAGAGAACTTTCATTGGCTGAAGGTTTCGCAGG 2794

N1.5 CATCTCTATGACCGAGCATATACAAAGAAGAGAACTTTCATTGGCTGAAGGTTTCGCAGG 2794

N1.27 CATCTCTATGACCGAGCATATACAAAGAAGAGAACTTTCATTGGCTGAAGGTTTCGCAGG 2794

A1.2 CATCTCTATGACCGAGCATATACAAAGAAGAGAACTTTCATTGGCTGAAGGTTTCGCAGG 2939

A1.7 CATCTCTATGACCGAGCATATACAAAGAAGAGAACTTTCATTGGCTGAAGGTTTCGCAGG 2939

N3.9 CATCTCTATGACCGAGCATATACAAAGAAGAGAACTTTCATTGGCTGAAGGTTTCGCAGG 2939

** **********

MG637361.1 CACAGAAACATTAGTTAGTTTCAAAGGGTTGTCCCCTACATCGACAGGTTCGCTAGCGAA 2999

A1.9 CACAGAAACATTAGTTAGTTTCAAAGGGTTGTCCCCTACATCGACAGGTTCGCTAGCGAA 2299

N1.8 CACAGAAACATTAGTTAGTTTCAAAGGGTTGTCCCCTACATCGACAGGTTCGCTAGCGAA 2855

A1.4 CACAGAAACATTAGTTAGTTTCAAAGGGTTGTCCCCTACATCGACAGGTTCGCTAGCGAA 2999

A3.1 CACAGAAACATTAGTTAGTTTCAAAGGGTTGTCCCCTACATCGACAGGTTCGCTAGCGAA 2999

A3.4 CACAGAAACATTAGTTAGTTTCAAAGGGTTGTCCCCTACATCGACAGGTTCGCTAGCGAA 2999

A3.6 CACAGAAACATTAGTTAGTTTCAAAGGGTTGTCCCCTACATCGACAGGTTCGCTAGCGAA 2999

A3.7 CACAGAAACATTAGTTAGTTTCAAAGGGTTGTCCCCTACATCGACAGGTTCGCTAGCGAA 2999

A3.8 CACAGAAACATTAGTTAGTTTCAAAGGGTTGTCCCCTACATCGACAGGTTCGCTAGCGAA 2999

N3.1 CACAGAAACATTAGTTAGTTTCAAAGGGTTGTCCCCTACATCGACAGGTTCGCTAGCGAA 2999

N3.6 CACAGAAACATTAGTTAGTTTCAAAGGGTTGTCCCCTACATCGACAGGTTCGCTAGCGAA 2999

N3.3 CACAGAAACATTAGTTAGTTTCAAAGGGTTGTCCCCTACATCGACAGGTTCGCTAGCGAA 2999

N3.10 CACAGAAACATTAGTTAGTTTCAAAGGGTTGTCCCCTACATCGACAGGTTCGCTAGCGAA 2999

A1.3 CACAGAAACATTAGTTAGTTTCAAAGGGTTGTCCCCTACATCGACAGGTTCGCTAGCGAA 2854

N1.2 CACAGAAACATTAGTTAGTTTCAAAGGGTTGTCCCCTACATCGACAGGTTCGCTAGCGAA 2854

N1.5 CACAGAAACATTAGTTAGTTTCAAAGGGTTGTCCCCTACATCGACAGGTTCGCTAGCGAA 2854

N1.27 CACAGAAACATTAGTTAGTTTCAAAGGGTTGTCCCCTACATCGACAGGTTCGCTAGCGAA 2854

A1.2 CACAGAAACGCTAGTTAGTTTCAAAGGGTCGTCCCTTACATCGACAGGTTCGCTAGCGAA 2999

A1.7 CACAGAAACGCTAGTTAGTTTCAAAGGGTCGTCCCTTACATCGACAGGTTCGCTAGCGAA 2999

N3.9 CACAGAAACGCTAGTTAGTTTCAAAGGGTCGTCCCTTACATCGACAGGTTCGCTAGCGAA 2999

********* ****************** ***** ************************

MG637361.1 GGCTGCCTACGAGTCGATATTTGTAACCGCCAATAATCCCACAATGGAAATCACTGTTGT 3059

A1.9 GGCTGCCTACGAGTCGATATTTGTAACCGCCAATAATCCCACAATGGAAATCACTGTTGT 2359

N1.8 GGCTGCCTACGAGTCGATATTTGTAACCGCCAATAATCCCACAATGGAAATCACTGTTGT 2915

A1.4 GGCTGCCTACGAGTCGATATTTGTAACCGCCAATAATCCCACAATGGAAATCACTGTTGT 3059

A3.1 GGCTGCCTACGAGTCGATATTTGTAACCGCCAATAATCCCACAATGGAAATCACTGTTGT 3059

A3.4 GGCTGCCTACGAGTCGATATTTGTAACCGCCAATAATCCCACAATGGAAATCACTGTTGT 3059

A3.6 GGCTGCCTACGAGTCGATATTTGTAACCGCCAATAATCCCACAATGGAAATCACTGTTGT 3059

A3.7 GGCTGCCTACGAGTCGATATTTGTAACCGCCAATAATCCCACAATGGAAATCACTGTTGT 3059

A3.8 GGCTGCCTACGAGTCGATATTTGTAACCGCCAATAATCCCACAATGGAAATCACTGTTGT 3059

N3.1 GGCTGCCTACGAGTCGATATTTGTAACCGCCAATAATCCCACAATGGAAATCACTGTTGT 3059

N3.6 GGCTGCCTACGAGTCGATATTTGTAACCGCCAATAATCCCACAATGGAAATCACTGTTGT 3059

N3.3 GGCTGCCTACGAGTCGATATTTGTAACCGCCAATAATCCCACAATGGAAATCACTGTTGT 3059

N3.10 GGCTGCCTACGAGTCGATATTTGTAACCGCCAATAATCCCACAATGGAAATCACTGTTGT 3059

A1.3 GGCTGCCTACGAGTCGATATTTGTAACCGCCAATAATCCCACAATGGAAATCACTGTTGT 2914

N1.2 GGCTGCCTACGAGTCGATATTTGTAACCGCCAATAATCCCACAATGGAAATCACTGTTGT 2914

N1.5 GGCTGCCTACGAGTCGATATTTGTAACCGCCAATAATCCCACAATGGAAATCACTGTTGT 2914

N1.27 GGCTGCCTACGAGTCGATATTTGTAACCGCCAATAATCCCACAATGGAAATCACTGTTGT 2914

A1.2 GGCTGCCTACGAGTCGATATTTGTAACCGCCAATAATCCCACAATGGAAATCACTGTTGT 3059

A1.7 GGCTGCCTACGAGTCGATATTTGTAACCGCCAATAATCCCACAATGGAAATCACTGTTGT 3059

N3.9 GGCTGCCTACGAGTCGATATTTGTAACCGCCAATAATCCCACAATGGAAATCACTGTTGT 3059

************************************************************

MG637361.1 TGATAATACACCTATAGATGAATATTATTTGGAAAGAACAGATGACGTATCAGCGATGGC 3119

A1.9 TGATAATACACCTATAGATGAATATTATTTGGAAAGAACAGATGACGTATCAGCGATGGC 2419

N1.8 TGATAATACACCTATAGATGAATATTATTTGGAAAGAACAGATGACGTATCAGCGATGGC 2975

A1.4 TGATAATACACCTATAGATGAATATTATTTGGAAAGAACAGATGACGTATCAGCGATGGC 3119

A3.1 TGATAATACACCTATAGATGAATATTATTTGGAAAGAACAGATGACGTATCAGCGATGGC 3119

A3.4 TGATAATACACCTATAGATGAATATTATTTGGAAAGAACAGATGACGTATCAGCGATGGC 3119

A3.6 TGATAATACACCTATAGATGAATATTATTTGGAAAGAACAGATGACGTATCAGCGATGGC 3119

A3.7 TGATAATACACCTATAGATGAATATTATTTGGAAAGAACAGATGACGTATCAGCGATGGC 3119

A3.8 TGATAATACACCTATAGATGAATATTATTTGGAAAGAACAGATGACGTATCAGCGATGGC 3119

N3.1 TGATAATACACCTATAGATGAATATTATTTGGAAAGAACAGATGACGTATCAGCGATGGC 3119

N3.6 TGATAATACACCTATAGATGAATATTATTTGGAAAGAACAGATGACGTATCAGCGATGGC 3119

N3.3 TGATAATACACCTATAGATGAATATTATTTGGAAAGAACAGATGACGTATCAGCGATGGC 3119

N3.10 TGATAATACACCTATAGATGAATATTATTTGGAAAGAACAGATGACGTATCAGCGATGGC 3119

A1.3 TGATAATACACCTATAGATGAATATTATTTGGAAAGAACAGATGACGTATCAGCGATGGC 2974

N1.2 TGATAATACACCTATAGATGAATATTATTTGGAAAGAACAGATGACGTATCAGCGATGGC 2974

N1.5 TGATAATACACCTATAGATGAATATTATTTGGAAAGAACAGATGACGTATCAGCGATGGC 2974

N1.27 TGATAATACACCTATAGATGAATATTATTTGGAAAGA----ATGACGTATCAGCGATGGC 2970

A1.2 TGATAATACACCTATAGATGAATATTATTTGGAAAGAACAGATGACGTATCAGCGATGGC 3119

A1.7 TGATAATACACCTATAGATGAATATTATTTGGAAAGAACAGATGACGTATCAGCGATGGC 3119

N3.9 TGATAATACACCTATAGATGAATATTATTTGGAAAGAACAGATGACGTATCAGCGATGGC 3119

************************************* *******************

MG637361.1 GGTGCTCCGGCACAGTCTGTTGATCGGCGCGACGTTCGACGACCACTCCGCGACCGCGTG 3179

A1.9 GGTGCTCCGGCACAGTCTGTTGATCGGCGCGACGTTCGACGACCACTCCGCGACCGCGTG 2479

N1.8 GGTGCTCCGGCACAGTCTGTTGATCGGCGCGACGTTCGACGACCACTCCGCGACCGCGTG 3035

A1.4 GGTGCTCCGGCACAGTCTGTTGATCGGCGCGACGTTCGACGACCACTCCGCGACCGCGTG 3179

A3.1 GGTGCTCCGGCACAGTCTGTTGATCGGCGCGACGTTCGACGACCACTCCGCGACCGCGTG 3179

A3.4 GGTGCTCCGGCACAGTCTGTTGATCGGCGCGACGTTCGACGACCACTCCGCGACCGCGTG 3179

A3.6 GGTGCTCCGGCACAGTCTGTTGATCGGCGCGACGTTCGACGACCACTCCGCGACCGCGTG 3179

A3.7 GGTGCTCCGGCACAGTCTGTTGATCGGCGCGACGTTCGACGACCACTCCGCGACCGCGTG 3179

A3.8 GGTGCTCCGGCACAGTCTGTTGATCGGCGCGACGTTCGACGACCACTCCGCGACCGCGTG 3179

N3.1 GGTGCTCCGGCACAGTCTGTTGATCGGCGCGACGTTCGACGACCACTCCGCGACCGCGTG 3179

N3.6 GGTGCTCCGGCACAGTCTGTTGATCGGCGCGACGTTCGACGACCACTCCGCGACCGCGTG 3179

N3.3 GGTGCTCCGGCACAGTCTGTTGATCGGCGCGACGTTCGACGACCACTCCGCGACCGCGTG 3179

N3.10 GGTGCTCCGGCACAGTCTGTTGATCGGCGCGACGTTCGACGACCACTCCGCGACCGCGTG 3179

A1.3 GGTGCTCCGGCACAGTCTGTTGATCGGCGCGACGTTCGACGACCACTCCGCGACCGCGTG 3034

N1.2 GGTGCTCCGGCACAGTCTGTTGATCGGCGCGACGTTCGACGACCACTCCGCGACCGCGTG 3034

N1.5 GGTGCTCCGGCACAGTCTGTTGATCGGCGCGACGTTCGACGACCACTCCGCGACCGCGTG 3034

N1.27 GGTGCTCCGGCACAGTCTGTTGATCGGCGCGACGTTCGACGACCACTCCGCGACCGCGTG 3030

A1.2 GGTGCTCCGGCACAGTCTGTTGATCGGCGCGACGTTCGACGACAACTCCGCGACCGCGTG 3179

A1.7 GGTGCTCCGGCACAGTCTGTTGATCGGCGCGACGTTCGACGACAACTCCGCGACCGCGTG 3179

N3.9 GGTGCTCCGGCACAGTCTGTTGATCGGCGCGACGTTCGACGACAACTCCGCGACCGCGTG 3179

******************************************* ****************

MG637361.1 GTTCAGCAACTTCGGTTACCACGACGTGGCCATGTCACTGGCTGCTGTGCACGCCGCCTT 3239

A1.9 GTTCAGCAACTTCGGTTACCACGACGTGGCCATGTCACTGGCTGCTGTGCACGCCGCCTT 2539

N1.8 GTTCAGCAACTTCGGTTACCACGACGTGGCCATGTCACTGGCTGCTGTGCACGCCGCCTT 3095

A1.4 GTTCAGCAACTTCGGTTACCACGACGTGGCCATGTCACTGGCTGCTGTGCACGCCGCCTT 3239

A3.1 GTTCAGCAACTTCGGTTACCACGACGTGGCCATGTCACTGGCTGCTGTGCACGCCGCCTT 3239

A3.4 GTTCAGCAACTTCGGTTACCACGACGTGGCCATGTCACTGGCTGCTGTGCACGCCGCCTT 3239

A3.6 GTTCAGCAACTTCGGTTACCACGACGTGGCCATGTCACTGGCTGCTGTGCACGCCGCCTT 3239

A3.7 GTTCAGCAACTTCGGTTACCACGACGTGGCCATGTCACTGGCTGCTGTGCACGCCGCCTT 3239

A3.8 GTTCAGCAACTTCGGTTACCACGACGTGGCCATGTCACTGGCTGCTGTGCACGCCGCCTT 3239

N3.1 GTTCAGCAACTTCGGTTACCACGACGTGGCCATGTCACTGGCTGCTGTGCACGCCGCCTT 3239

N3.6 GTTCAGCAACTTCGGTTACCACGACGTGGCCATGTCACTGGCTGCTGTGCACGCCGCCTT 3239

N3.3 GTTCAGCAACTTCGGTTACCACGACGTGGCCATGTCACTGGCTGCTGTGCACGCCGCCTT 3239

N3.10 GTTCAGCAACTTCGGTTACCACGACGTGGCCATGTCACTGGCTGCTGTGCACGCCGCCTT 3239

A1.3 GTTCAGCAACTTCGGTTACCACGACGTGGCCATGTCACTGGCTGCTGTGCACGCCGCCTT 3094

N1.2 GTTCAGCAACTTCGGTTACCACGACGTGGCCATGTCACTGGCTGCTGTGCACGCCGCCTT 3094

N1.5 GTTCAGCAACTTCGGTTACCACGACGTGGCCATGTCACTGGCTGCTGTGCACGCCGCCTT 3094

N1.27 GTTCAGCAACTTCGGTTACCACGACGTGGCCATGTCACTGGCTGCTGTGCACGCCGCCTT 3090

A1.2 GTTCAGCAACTTCGGTTACCACGACGTGGCCATGTCACTGGCGGCTGTGCACGCCGCCTT 3239

A1.7 GTTCAGCAACTTCGGTTACCACGACGTGGCCATGTCACTGGCGGCTGTGCACGCCGCCTT 3239

N3.9 GTTCAGCAACTTCGGTTACCACGACGTGGCCATGTCACTGGCGGCTGTGCACGCCGCCTT 3239

****************************************** *****************

MG637361.1 GCTCAGAGCTGTCAATCCTGCAGCCAACTTGACTGTTTACAACCACCCACTTGAGGCCAA 3299

A1.9 GCTCAGAGCTGTCAATCCTGCAGCCAACTTGACTGTTTACAACCACCCACTTGAGGCCAA 2599

N1.8 GCTCAGAGCTGTCAATCCTGCAGCCAACTTGACTGTTTACAACCACCCACTTGAGGCCAA 3155

A1.4 GCTCAGAGCTGTCAATCCTGCAGCCAACTTGACTGTTTACAACCACCCACTTGAGGCCAA 3299

A3.1 GCTCAGAGCTGTCAATCCTGCAGCCAACTTGACTGTTTACAACCACCCACTTGAGGCCAA 3299

A3.4 GCTCAGAGCTGTCAATCCTGCAGCCAACTTGACTGTTTACAACCACCCACTTGAGGCCAA 3299

A3.6 GCTCAGAGCTGTCAATCCTGCAGCCAACTTGACTGTTTACAACCACCCACTTGAGGCCAA 3299

A3.7 GCTCAGAGCTGTCAATCCTGCAGCCAACTTGACTGTTTACAACCACCCACTTGAGGCCAA 3299

A3.8 GCTCAGAGCTGTCAATCCTGCAGCCAACTTGACTGTTTACAACCACCCACTTGAGGCCAA 3299

N3.1 GCTCAGAGCTGTCAATCCTGCAGCCAACTTGACTGTTTACAACCACCCACTTGAGGCCAA 3299

N3.6 GCTCAGAGCTGTCAATCCTGCAGCCAACTTGACTGTTTACAACCACCCACTTGAGGCCAA 3299

N3.3 GCTCAGAGCTGTCAATCCTGCAGCCAACTTGACTGTTTACAACCACCCACTTGAGGCCAA 3299

N3.10 GCTCAGAGCTGTCAATCCTGCAGCCAACTTGACTGTTTACAACCACCCACTTGAGGCCAA 3299

A1.3 GCTCAGAGCTGTCAATCCTGCAGCCAACTTGACTGTTTACAACCACCCACTTGAGGCCAA 3154

N1.2 GCTCAGAGCTGTCAATCCTGCAGCCAACTTGACTGTTTACAACCACCCACTTGAGGCCAA 3154

N1.5 GCTCAGAGCTGTCAATCCTGCAGCCAACTTGACTGTTTACAACCACCCACTTGAGGCCAA 3154

N1.27 GCTCAGAGCTGTCAATCCTGCAGCCAACTTGACTGTTTACAACCACCCACTTGAGGCCAA 3150

A1.2 GCTCAGAGCTGTCAATCCTGCAGCCAACTTGACTGTTTACAACCACCCACTTGAGGCCAA 3299

A1.7 GCTCAGAGCTGTCAATCCTGCAGCCAACTTGACTGTTTACAACCACCCACTTGAGGCCAA 3299

N3.9 GCTCAGAGCTGTCAATCCTGCAGCCAACTTGACTGTTTACAACCACCCACTTGAGGCCAA 3299

************************************************************

MG637361.1 TTATGTCAACCAGAACGACATGCAAACAATGGTAGCGTTCCTCTCGATGCAACTTGCGTC 3359

A1.9 TTATGTCAACCAGCAATCAGACCCAATACAATTGGTTCCCCAGCCTC--CCTCTT----- 2652

N1.8 TTATGTCAACCAGAACGACATGCAAACAATGGTAGCGTTCCTCTCGATGCAACTTGCGTC 3215

A1.4 TTATGTCAACCAGAACGACATGCAAACAATGGTAGCGTTCCTCTCGATGCAACTTGCGTC 3359

A3.1 TTATGTCAACCAGAACGACATGCAAACAATGGTAGCGTTCCTCTCGATGCAACTTGCGTC 3359

A3.4 TTATGTCAACCAGAACGACATGCAAACAATGGTAGCGTTCCTCTCGATGCAACTTGCGTC 3359

A3.6 TTATGTCAACCAGAACGACATGCAAACAATGGTAGCGTTCCTCTCGATGCAACTTGCGTC 3359

A3.7 TTATGTCAACCAGAACGACATGCAAACAATGGTAGCGTTCCTCTCGATGCAACTTGCGTC 3359

A3.8 TTATGTCAACCAGAACGACATGCAAACAATGGTAGCGTTCCTCTCGATGCAACTTGCGTC 3359

N3.1 TTATGTCAACCA------------------------------------------------ 3311

N3.6 TTATGTCAACCA------------------------------------------------ 3311

N3.3 TTATGTCAACCA------------------------------------------------ 3311

N3.10 TTATGTCAACCA------------------------------------------------ 3311

A1.3 TTATGTCAACCA------------------------------------------------ 3166

N1.2 TTATGTCAACCAGAACGACATGCAAACAATGGTAGCGTTCCTCTCGATGCAACTTGCGTC 3214

N1.5 TTATGTCAACCAGAACGACATGCAAACAATGGTAGCGTTCCTCTCGATGCAACTTGCGTC 3214

N1.27 TTATGTCAACCAGAACGACATGCAAACAATGGTAGCGTTCCTCTCGATGCAACTTGCGTC 3210

A1.2 TTATGTCAACCAGAACGACATGCAAACGATGGTAGCGTTCCTCTCGATGCAACTTGCGTC 3359

A1.7 TTATGTCAACCAGAACGACATGCAAACGATGGTAGCGTTCCTCTCGATGCAACTTGCGTC 3359

N3.9 TTATGTCAACCAGAACGACATGCAAACGATGGTAGCGTTCCTCTCGATGCAACTTGCGTC 3359

************

MG637361.1 GGGCATCGGCAGCAGTCTGTCAATTGTCAGTGCTGTGTTCATCATGTTCTATATCAAGGA 3419

A1.9 --TAA----------------------------------------------TACAAAGCA 2664

N1.8 GGGCATCGGCAGCAGTCTGTCAATTGTCAGTGCTGTGTTTATCATGTTCTATATCAAGGA 3275

A1.4 GGGCATCGGCAGCAGTCTGTCAATTGTCAGTGCTGTGTTCATCATGTTCTATATCAAG-- 3417

A3.1 GGGCATCGGCAGCAGTCTGTCAATTGTCAGTGCTGTGTTCATCATGTTCTATATCAAGGA 3419

A3.4 GGGCATCGGCAGCAGTCTGTCAATTGTCAGTGCTGTGTTCATCATGTTCTATATCAAGGA 3419

A3.6 GGGCATCGGCAGCAGTCTGTCAATTGTCAGTGCTGTGTTCATCATGTTCTATATCAAGGA 3419

A3.7 GGGCATCGGCAGCAGTCTGTCAATTGTCAGTGCTGTGTTCATCATGTTCTATATCAAGGA 3419

A3.8 GGGCATCGGCAGCAGTCTGTCAATTGTCAGTGCTGTGTTCATCATGTTCTATATCAAGGA 3419

N3.1 ------------------------------------------------------------ 3311

N3.6 ------------------------------------------------------------ 3311

N3.3 ------------------------------------------------------------ 3311

N3.10 ------------------------------------------------------------ 3311

A1.3 ------------------------------------------------------------ 3166

N1.2 GGGCATCGGCAGCAGTCTGTCAATTGTCAGTGCTGTGTTCATCATGTTCTATATCAAG-- 3272

N1.5 GGGCATCGGCAGCAGTCTGTCAATTGTCAGTGCTGTGTTCATCATGTTCTATATCAAG-- 3272

N1.27 GGGCATCGGCAGCAGTCTGTCAATTGTCAGTGCTGTGTTCATCATGTTCTATATCAAG-- 3268

A1.2 GGGCATCGGCAGCAGTCTGTCAATTGTCAGTGCTGTGTTCATCATGTTCTATATCAAGGA 3419

A1.7 GGGCATCGGCAGCAGTCTGTCAATTGTCAGTGCTGTGTTCATCATGTTCTATATCAAGGA 3419

N3.9 GGGCATCGGCAGCAGTCTGTCAATTGTCAGTGCTGTGTTCATCATGTTCTATATCAAGGA 3419

MG637361.1 GCGAGTATCTCGCGCCAAGCTGCTGCAGAAGGCGGCAGGCATCCAGCCGTTAGTGATGTG 3479

A1.9 GTGGACATTC-------AGAAATTTCAGTGAGT---TGAAGAGCAGCAGTTAGAAATTCT 2714

N1.8 GCGAGTATCTCGCGCCAAGCTGCTGCAGAAGGCGGCAGGCATCCAGCCGTTAGTGATGTG 3335

A1.4 ------------------------------------------------------------ 3417

A3.1 GCGAGTATCTCGCGCCAAGCTGCTGCAGAAGGCGGCAGGCATCCAGCCGTTAGTGATGTG 3479

A3.4 GCGAGTATCTCGCGCCAAGCTGCTGCAGAAGGCGGCAGGCATCCAGCCGTTAGTGATGTG 3479

A3.6 GCGAGTATCTCGCGCCAAGCTGCTGCAGAAGGCGGCAGGCATCCAGCCGTTAGTGATGTG 3479

A3.7 GCGAGTATCTCGCGCCAAGCTGCTGCAGAAGGCGGCAGGCATCCAGCCGTTAGTGATGTG 3479

A3.8 GCGAGTATCTCGCGCCAAGCTGCTGCAGAAGGCGGCAGGCATCCAGCCGTTAGTGATGTG 3479

N3.1 ------------------------------------------------------------ 3311

N3.6 ------------------------------------------------------------ 3311

N3.3 ------------------------------------------------------------ 3311

N3.10 ------------------------------------------------------------ 3311

A1.3 ------------------------------------------------------------ 3166

N1.2 ------------------------------------------------------------ 3272

N1.5 ------------------------------------------------------------ 3272

N1.27 ------------------------------------------------------------ 3268

A1.2 GCGAGTATCTCGCGCCAAGCTGCTGCAGAAGGCGGCAGGCATCCAGCCGTTAGTGATGTG 3479

A1.7 GCGAGTATCTCGCGCCAAGCTGCTGCAGAAGGCGGCAGGCATCCAGCCGTTAGTGATGTG 3479

N3.9 GCGAGTATCTCGCGCCAAGCTGCTGCAGAAGGCGGCAGGCATCCAGCCGTTAGTGATGTG 3479

MG637361.1 GCTCAGCGCCGCCGTGTTCGACTGGATCTGGTTCTGCGTCATCGCCGTCGGCATCGTTAT 3539

A1.9 GCGGCCCAATGC------------------------------------------------ 2726

N1.8 GCTCAGCGCCGCCGTGTTCGACTGGATCTGGTTCTGCGTCATCGCCGTCGGCATCGTTAT 3395

A1.4 ------------------------------------------------------------ 3417

A3.1 GCTCAGCGCCGCCGTGTTCGACTGGATCTGGTTCTGCGTCATCGCCGTCGGCATCGTTAT 3539

A3.4 GCTCAGCGCCGCCGTGTTCGACTGGATCTGGTTCTGCGTCATCGCCGTCGGCATCGTTAT 3539

A3.6 GCTCAGCGCCGCCGTGTTCGACTGGATCTGGTTCTGCGTCATCGCCGTCGGCATCGTTAT 3539

A3.7 GCTCAGCGCCGCCGTGTTCGACTGGATCTGGTTCTGCGTCATCGCCGTCGGCATCGTTAT 3539

A3.8 GCTCAGCGCCGCCGTGTTCGACTGGATCTGGTTCTGCGTCATCGCCGTCGGCATCGTTAT 3539

N3.1 ------------------------------------------------------------ 3311

N3.6 ------------------------------------------------------------ 3311

N3.3 ------------------------------------------------------------ 3311

N3.10 ------------------------------------------------------------ 3311

A1.3 ------------------------------------------------------------ 3166

N1.2 ------------------------------------------------------------ 3272

N1.5 ------------------------------------------------------------ 3272

N1.27 ------------------------------------------------------------ 3268

A1.2 GCTCAGCGCCGCCGTGTTCGACTGGATCTGGTTCTGCATCATCGCCGTCGGCATCGTTAT 3539

A1.7 GCTCAGCGCCGCCGTGTTCGACTGGATCTGGTTCTGCATCATCGCCGTCGGCATCGTTAT 3539

N3.9 GCTCAGCGCCGCCGTGTTCGACTGGATCTGGTTCTGCATCATCGCCGTCGGCATCGTTAT 3539

MG637361.1 CGCCTGCGCCGCTTTTAACGTCATTGGGCT------------------------------ 3569

A1.9 ----------------------ACTGGTCAAGGCGAACATG----------ATGAAGCGC 2754

N1.8 CGCCTGCGCCGCTTTTGCGCGCCGTGTTTATTACGGTCATGGCGTCAAAAGATATAGAGC 3455

A1.4 ------------------------------------------------------------ 3417

A3.1 CGCCTGCGCCGCTTTTGCGCGCCGTGTTTATTACGGTCATGGCGTCAAAAGATATAGAGC 3599

A3.4 CGCCTGCGCCGCTTTTGCGCGCCGTGTTTATTACGGTCATGGCGTCAAAAGATATAGAGC 3599

A3.6 CGCCTGCGCCGCTTTTGCGCGCCGTGTTTATTACGGTCATGGCGTCAAAAGATATAGAGC 3599

A3.7 CGCCTGCGCCGCTTTTGCGCGCCGTGTTTATTACGGTCATGGCGTCAAAAGATATAGAGC 3599

A3.8 CGCCTGCGCCGCTTTTGCGCGCCGTGTTTATTACGGTCATGGCGTCAAAAGATATAGAGC 3599

N3.1 ------------------------------------------------------------ 3311

N3.6 ------------------------------------------------------------ 3311

N3.3 ------------------------------------------------------------ 3311

N3.10 ------------------------------------------------------------ 3311

A1.3 ------------------------------------------------------------ 3166

N1.2 ------------------------------------------------------------ 3272

N1.5 ------------------------------------------------------------ 3272

N1.27 ------------------------------------------------------------ 3268

A1.2 CGCCTGCGCCGCTTTTAACGTCATTGGGCT------------------------------ 3569

A1.7 CGCCTGCGCCGCTTTTAACGTCATTGGGCT------------------------------ 3569

N3.9 CGCCTGCGCCGCTTTTAACGTCATTGGGCT------------------------------ 3569

MG637361.1 ------------------------------------------------------------ 3569

A1.9 AG--------CATT---------------------------------------------- 2760

N1.8 TGAATACCTTTATTGTGTATTTCAGTGTTCCACATCGCTTTACTCGGTGAAGTATGCATA 3515

A1.4 ------------------------------------------------------------ 3417

A3.1 TGAATACCTTTATTGTGTATTTCAGTGTTCCACATCGCTTTACTCGGTGAAGTATGCATA 3659

A3.4 TGAATACCTTTATTGTGTATTTCAGTGTTCCACATCGCTTTACTCGGTGAAGTATGCATA 3659

A3.6 TGAATACCTTTATTGTGTATTTCAGTGTTCCACATCGCTTTACTCGGTGAAGTATGCATA 3659

A3.7 TGAATACCTTTATTGTGTATTTCAGTGTTCCACATCGCTTTACTCGGTGAAGTATGCATA 3659

A3.8 TGAATACCTTTATTGTGTATTTCAGTGTTCCACATCGCTTTACTCGGTGAAGTATGCATA 3659

N3.1 ------------------------------------------------------------ 3311

N3.6 ------------------------------------------------------------ 3311

N3.3 ------------------------------------------------------------ 3311

N3.10 ------------------------------------------------------------ 3311

A1.3 ------------------------------------------------------------ 3166

N1.2 ------------------------------------------------------------ 3272

N1.5 ------------------------------------------------------------ 3272

N1.27 ------------------------------------------------------------ 3268

A1.2 ------------------------------------------------------------ 3569

A1.7 ------------------------------------------------------------ 3569

N3.9 ------------------------------------------------------------ 3569

MG637361.1 ------------------------------------------------------------ 3569

A1.9 ----------------------------GAGAGAG-ACGGAATTGCCGCTAACGCTGTGG 2791

N1.8 AAATATGTCGCCTTTTCGGAACTGGCTTGATGGAAATCAATATTTATGTTATCAGTCAGG 3575

A1.4 ------------------------------------------------------------ 3417

A3.1 AAATATGTCGCCTTTTCGGAACTGGCTTGATGGAAATCAATATTTATGTTATCAGTCAGG 3719

A3.4 AAATATGTCGCCTTTTCGGAACTGGCTTGATGGAAATCAATATTTATGTTATCAGTCAGG 3719

A3.6 AAATATGTCGCCTTTTCGGAACTGGCTTGATGGAAATCAATATTTATGTTATCAGTCAGG 3719

A3.7 AAATATGTCGCCTTTTCGGAACTGGCTTGATGGAAATCAATATTTATGTTATCAGTCAGG 3719

A3.8 AAATATGTCGCCTTTTCGGAACTGGCTTGATGGAAATCAATATTTATGTTATCAGTCAGG 3719

N3.1 ------------------------------------------------------------ 3311

N3.6 ------------------------------------------------------------ 3311

N3.3 ------------------------------------------------------------ 3311

N3.10 ------------------------------------------------------------ 3311

A1.3 ------------------------------------------------------------ 3166

N1.2 ------------------------------------------------------------ 3272

N1.5 ------------------------------------------------------------ 3272

N1.27 ------------------------------------------------------------ 3268

A1.2 ------------------------------------------------------------ 3569

A1.7 ------------------------------------------------------------ 3569

N3.9 ------------------------------------------------------------ 3569

MG637361.1 ------------------------------------------------------------ 3569

A1.9 CGGCAGTGGAGGAAGGATTA---------------------------------------- 2811

N1.8 ATTAGTTGGACGACTCATAAAATATCAATCAATATTCTCTTGCAGCCTCATGAGATCGAT 3635

A1.4 ------------------------------------------------------------ 3417

A3.1 ATTAGTTGGACGACTCATAAAATATCAATCAATATTCTCTTGCAGCCTCATGAGATCGAT 3779

A3.4 ATTAGTTGGACGACTCATAAAATATCAATCAATATTCTCTTGCAGCCTCATGAGATCGAT 3779

A3.6 ATTAGTTGGACGACTCATAAAATATCAATCAATATTCTCTTGCAGCCTCATGAGATCGAT 3779

A3.7 ATTAGTTGGACGACTCATAAAATATCAATCAATATTCTCTTGCAGCCTCATGAGATCGAT 3779

A3.8 ATTAGTTGGACGACTCATAAAATATCAATCAATATTCTCTTGCAGCCTCATGAGATCGAT 3779

N3.1 ------------------------------------------------------------ 3311

N3.6 ------------------------------------------------------------ 3311

N3.3 ------------------------------------------------------------ 3311

N3.10 ------------------------------------------------------------ 3311

A1.3 ------------------------------------------------------------ 3166

N1.2 ------------------------------------------------------------ 3272

N1.5 ------------------------------------------------------------ 3272

N1.27 ------------------------------------------------------------ 3268

A1.2 ------------------------------------------------------------ 3569

A1.7 ------------------------------------------------------------ 3569

N3.9 ------------------------------------------------------------ 3569

MG637361.1 ------------------------------------------------------------ 3569

A1.9 -------AA--------------------------------------------------- 2813

N1.8 ATGACTCAATGTACTTTACTGAAGTGCTTGCACCTACTGGGTAATAGATAAAACCAATTG 3695

A1.4 ------------------------------------------------------------ 3417

A3.1 ATGACTCAATGTACTTTACTGAAGTGCTTGCACCTACTGGGTAATAGATAAAACCAATTG 3839

A3.4 ATGACTCAATGTACTTTACTGAAGTGCTTGCACCTACTGGGTAATAGATAAAACCAATTG 3839

A3.6 ATGACTCAATGTACTTTACTGAAGTGCTTGCACCTACTGGGTAATAGATAAAACCAATTG 3839

A3.7 ATGACTCAATGTACTTTACTGAAGTGCTTGCACCTACTGGGTAATAGATAAAACCAATTG 3839

A3.8 ATGACTCAATGTACTTTACTGAAGTGCTTGCACCTACTGGGTAATAGATAAAACCAATTG 3839

N3.1 ------------------------------------------------------------ 3311

N3.6 ------------------------------------------------------------ 3311

N3.3 ------------------------------------------------------------ 3311

N3.10 ------------------------------------------------------------ 3311

A1.3 ------------------------------------------------------------ 3166

N1.2 ------------------------------------------------------------ 3272

N1.5 ------------------------------------------------------------ 3272

N1.27 ------------------------------------------------------------ 3268

A1.2 ------------------------------------------------------------ 3569

A1.7 ------------------------------------------------------------ 3569

N3.9 ------------------------------------------------------------ 3569

MG637361.1 ---------------------CTCTTCTGTCGATGAACTGGGTCGGATGTACTTGTGCAT 3608

A1.9 ---------TGCGCTAGCAGAAGCTATTAGG---------------TTG----------- 2838

N1.8 TTTTGTTCATATGTTAGAACCATGTTTTTTTTTTCCTTCAGGTCGGATGTACTTGTGCAT 3755

A1.4 -----------------------------------------GTCGGATGTACTTGTGCAT 3436

A3.1 TTTTGTTCATATGTTAGAACCATGTTTTTTTTTTCCTTCAGGTCGGATGTACTTGTGCAT 3899

A3.4 TTTTGTTCATATGTTAGAACCATGTTTTTTTTTTCCTTCAGGTCGGATGTACTTGTGCAT 3899

A3.6 TTTTGTTCATATGTTAGAACCATGTTTTTTTTTTCCTTCAGGTCGGATGTACTTGTGCAT 3899

A3.7 TTTTGTTCATATGTTAGAACCATGTTTTTTTTTTCCTTCAGGTCGGATGTACTTGTGCAT 3899

A3.8 TTTTGTTCATATGTTAGAACCATGTTTTTTTTTTCCTTCAGGTCGGATGTACTTGTGCAT 3899

N3.1 ----------------------------------------GGTCGGATGTACTTGTGCAT 3331

N3.6 ----------------------------------------GGTCGGATGTACTTGTGCAT 3331

N3.3 ----------------------------------------GGTCGGATGTACTTGTGCAT 3331

N3.10 ----------------------------------------GGTCGGATGTACTTGTGCAT 3331

A1.3 ----------------------------------------GGTCGGATGTACTTGTGCAT 3186

N1.2 -----------------------------------------GTCGGATGTACTTGTGCAT 3291

N1.5 -----------------------------------------GTCGGATGTACTTGTGCAT 3291

N1.27 -----------------------------------------GTCGGATGTACTTGTGCAT 3287

A1.2 ---------------------CTCTTCTGTCGATGAACTGG------------------- 3590

A1.7 ---------------------CTCTTCTGTCGATGAACTGG------------------- 3590

N3.9 ---------------------CTCTTCTGTCGATGAACTGGGTCGGATGTACTTGTGCAT 3608

MG637361.1 CATAGTGTATGGCGCCGCCAGTCTACCGATAGGCTACGTGTTCTCCTATTTCTTCAAAGG 3668

A1.9 ------------------------------------------------------------ 2838

N1.8 CATAGTGTATGGCGCCGCCAGTCTACCGATAGGCTACGTGTTCTCCTATTTCTTCAAAGG 3815

A1.4 CATAGTGTATGGCGCCGCCAGTCTACCGATAGGCTACGTGTTCTCCTATTTCTTCAAAGG 3496

A3.1 CATAGTGTATGGCGCCGCCAGTCTACCGATAGGCTACGTGTTCTCCTATTTCTTCAAAGG 3959

A3.4 CATAGTGTATGGCGCCGCCAGTCTACCGATAGGCTACGTGTTCTCCTATTTCTTCAAAGG 3959

A3.6 CATAGTGTATGGCGCCGCCAGTCTACCGATAGGCTACGTGTTCTCCTATTTCTTCAAAGG 3959

A3.7 CATAGTGTATGGCGCCGCCAGTCTACCGATAGGCTACGTGTTCTCCTATTTCTTCAAAGG 3959

A3.8 CATAGTGTATGGCGCCGCCAGTCTACCGATAGGCTACGTGTTCTCCTATTTCTTCAAAGG 3959

N3.1 CATAGTGTATGGCGCCGCCAGTCTACCGATAGGCTACGTGTTCTCCTATTTCTTCAAAGG 3391

N3.6 CATAGTGTATGGCGCCGCCAGTCTACCGATAGGCTACGTGTTCTCCTATTTCTTCAAAGG 3391

N3.3 CATAGTGTATGGCGCCGCCAGTCTACCGATAGGCTACGTGTTCTCCTATTTCTTCAAAGG 3391

N3.10 CATAGTGTATGGCGCCGCCAGTCTACCGATAGGCTACGTGTTCTCCTATTTCTTCAAAGG 3391

A1.3 CATAGTGTATGGCGCCGCCAGTCTACCGATAGGCTACGTGTTCTCCTATTTCTTCAAAGG 3246

N1.2 CATAGTGTATGGCGCCGCCAGTCTACCGATAGGCTACGTGTTCTCCTATTTCTTCAAAGG 3351

N1.5 CATAGTGTATGGCGCCGCCAGTCTACCGATAGGCTACGTGTTCTCCTATTTCTTCAAAGG 3351

N1.27 CATAGTGTATGGCGCCGCCAGTCTACCGATAGGCTACGTGTTCTCCTATTTCTTCAAAGG 3347

A1.2 ------------------------------------------------------------ 3590

A1.7 ------------------------------------------------------------ 3590

N3.9 CATAGTGTATGGCGCCGCCAGTCTGCCGATAGGCTACGTGTTCTCCTATTTCTTCAAAGG 3668

MG637361.1 CCCTGCCGTCGGTTTTGTCACCATGTTCTTTATCAACATTCTCTTTGGTATGATGGGGGC 3728

A1.9 AACTGGGGTCAAATAAATAAGTATTTAAATTAACG---------------------ACAT 2877

N1.8 CCCTGCCGTCGGTTTTGTCACCATGTTCTTTATCAACATTCTCTTTGGTATGATGGGGGC 3875

A1.4 CCCTGCCGTCGGTTTTGTCACCATGTTCTTTATCAACATTCTCTTTGGTATGATGGGGGC 3556

A3.1 CCCTGCCGTCGGTTTTGTCACCATGTTCTTTATCAACATTCTCTTTGGTATGATGGGGGC 4019

A3.4 CCCTGCCGTCGGTTTTGTCACCATGTTCTTTATCAACATTCTCTTTGGTATGATGGGGGC 4019

A3.6 CCCTGCCGTCGGTTTTGTCACCATGTTCTTTATCAACATTCTCTTTGGTATGATGGGGGC 4019

A3.7 CCCTGCCGTCGGTTTTGTCACCATGTTCTTTATCAACATTCTCTTTGGTATGATGGGGGC 4019

A3.8 CCCTGCCGTCGGTTTTGTCACCATGTTCTTTATCAACATTCTCTTTGGTATGATGGGGGC 4019

N3.1 CCCTGCCGTCGGTTTTGTCACCATGTTCTTTATCAACATTCTCTTTGGTATGATGGGGGC 3451

N3.6 CCCTGCCGTCGGTTTTGTCACCATGTTCTTTATCAACATTCTCTTTGGTATGATGGGGGC 3451

N3.3 CCCTGCCGTCGGTTTTGTCACCATGTTCTTTATCAACATTCTCTTTGGTATGATGGGGGC 3451

N3.10 CCCTGCCGTCGGTTTTGTCACCATGTTCTTTATCAACATTCTCTTTGGTATGATGGGGGC 3451

A1.3 CCCTGCCGTCGGTTTTGTCACCATGTTCTTTATCAACATTCTCTTTGGTATGATGGGGGC 3306

N1.2 CCCTGCCGTCGGTTTTGTCACCATGTTCTTTATCAACATTCTCTTTGGTATGATGGGGGC 3411

N1.5 CCCTGCCGTCGGTTTTGTCACCATGTTCTTTATCAACATTCTCTTTGGTATGATGGGGGC 3411

N1.27 CCCTGCCGTCGGTTTTGTCACCATGTTCTTTATCAACATTCTCTTTGGTATGATGGGGGC 3407

A1.2 -----------------------------------------------GTATGATGGGGGC 3603

A1.7 -----------------------------------------------GTATGATGGGGGC 3603

N3.9 CCCTGCCGTCGGTTTTGTCACCATGTTCTTTATCAACATTCTCTTTGGTATGATGGGGGC 3728

MG637361.1 GCAGATTGTGGAGGCCTTGTTGTCACCGCAGCTTGATACTGAAAATGTCGCTAATATACT 3788

A1.9 GCAAACAATGGTAGCGTTCCTCTCGATGCAACTTGCG----------------------- 2914

N1.8 GCAGATTGTGGAGGCCTTGTTGTCACCGCAGCTTGATACTGAAAATGTCGCTAATATACT 3935

A1.4 GCAGATTGTGGAGGCCTTGTTGTCACCGCAGCTTGATACTGAAAATGTCGCTAATATACT 3616

A3.1 GCAGATTGTGGAGGCCTTGTTGTCACCGCAGCTTGATACTGAAAATGTCGCTAATATACT 4079

A3.4 GCAGATTGTGGAGGCCTTGTTGTCACCGCAGCTTGATACTGAAAATGTCGCTAATATACT 4079

A3.6 GCAGATTGTGGAGGCCTTGTTGTCACCGCAGCTTGATACTGAAAATGTCGCTAATATACT 4079

A3.7 GCAGATTGTGGAGGCCTTGTTGTCACCGCAGCTTGATACTGAAAATGTCGCTAATATACT 4079

A3.8 GCAGATTGTGGAGGCCTTGTTGTCACCGCAGCTTGATACTGAAAATGTCGCTAATATACT 4079

N3.1 GCAGATTGTGGAGGCCTTGTTGTCACCGCAGCTTGATACTGAAAATGTCGCTAATATACT 3511

N3.6 GCAGATTGTGGAGGCCTTGTTGTCACCGCAGCTTGATACTGAAAATGTCGCTAATATACT 3511

N3.3 GCAGATTGTGGAGGCCTTGTTGTCACCGCAGCTTGATACTGAAAATGTCGCTAATATACT 3511

N3.10 GCAGATTGTGGAGGCCTTGTTGTCACCGCAGCTTGATACTGAAAATGTCGCTAATATACT 3511

A1.3 GCAGATTGTGGAGGCCTTGTTGTCACCGCAGCTTGATACTGAAAATGTCGCTAATATACT 3366

N1.2 GCAGATTGTGGAGGCCTTGTTGTCACCGCAGCTTGATACTGAAAATGTCGCTAATATACT 3471

N1.5 GCAGATTGTGGAGGCCTTGTTGTCACCGCAGCTTGATACTGAAAATGTCGCTAATATACT 3471

N1.27 GCAGATTGTGGAGGCCTTGTTGTCACCGCAGCTTGATACTGAAAATGTCGCTAATATACT 3467

A1.2 GCAGATTGTGGAGGCCTTGTTGTCACCGCAGCTTGATACTGAAAATGTCGCTAATATACT 3663

A1.7 GCAGATTGTGGAGGCCTTGTTGTCACCGCAGCTTGATACTGAAAATGTCGCTAATATACT 3663

N3.9 GCAGATTGTGGAGGCCTTGTTGTCACCGCAGCTTGATACTGAAAATGTCGCTAATATACT 3788

*** * *** ** ** * ** *** ****

MG637361.1 TGACTCCATCTTGCAATTCTTCCCACTCTATAGTCTTGTCACATCTGCCAGACTGTTGAA 3848

A1.9 ------------------------------------------------------------ 2914

N1.8 TGACTCCATCTTGCAATTCTTCCCACTCTATAGTCTTGTCACATCTGCCAGACTGTTGAA 3995

A1.4 TGACTCCATCTTGCAATTCTTCCCACTCTATAGTCTTGTCACATCTGCCAGACTGTTGAA 3676

A3.1 TGACTCCATCTTGCAATTCTTCCCACTCTATAGTCTTGTCACATCTGCCAGACTGTTGAA 4139

A3.4 TGACTCCATCTTGCAATTCTTCCCACTCTATAGTCTTGTCACATCTGCCAGACTGTTGAA 4139

A3.6 TGACTCCATCTTGCAATTCTTCCCACTCTATAGTCTTGTCACATCTGCCAGACTGTTGAA 4139

A3.7 TGACTCCATCTTGCAATTCTTCCCACTCTATAGTCTTGTCACATCTGCCAGACTGTTGAA 4139

A3.8 TGACTCCATCTTGCAATTCTTCCCACTCTATAGTCTTGTCACATCTGCCAGACTGTTGAA 4139

N3.1 TGACTCCATCTTGCAATTCTTCCCACTCTATAGTCTTGTCACATCTGCCAGACTGTTGAA 3571

N3.6 TGACTCCATCTTGCAATTCTTCCCACTCTATAGTCTTGTCACATCTGCCAGACTGTTGAA 3571

N3.3 TGACTCCATCTTGCAATTCTTCCCACTCTATAGTCTTGTCACATCTGCCAGACTGTTGAA 3571

N3.10 TGACTCCATCTTGCAATTCTTCCCACTCTATAGTCTTGTCACATCTGCCAGACTGTTGAA 3571

A1.3 TGACTCCATCTTGCAATTCTTCCCACTCTATAGTCTTGTCACATCTGCCAGACTGTTGAA 3426

N1.2 TGACTCCATCTTGCAATTCTTCCCACTCTATAGTCTTGTCACATCTGCCAGACTGTTGAA 3531

N1.5 TGACTCCATCTTGCAATTCTTCCCACTCTATAGTCTTGTCACATCTGCCAGACTGTTGAA 3531

N1.27 TGACTCCATCTTGCAATTCTTCCCACTCTATAGTCTTGTCACATCTGCCAGACTGTTGAA 3527

A1.2 TGACTCCATCTTGCAATTCTTCCCACTCTATGGTCTTGTCACATCTGCCAG--------- 3714

A1.7 TGACTCCATCTTGCAATTCTTCCCACTCTATGGTCTTGTCACATCTGCCAG--------- 3714

N3.9 TGACTCCATCTTGCAATTCTTCCCACTCTATGGTCTTGTCACATCTGCCAGACTGTTGAA 3848

MG637361.1 TCAGGTGGGACTGCTGGAGTGGTCGTGCCTGCAGAACTGCGAGTACCTGTCCGCAGTGAT 3908

A1.9 ----------------------TCGGGCATCGGCAGCA------GTCTGTCAAT------ 2940

N1.8 TCAGGTGGGACTGCTGGAGTGGTCGTGCCTGCAGAACTGCGAGTACCTGTCCGCAGTGAT 4055

A1.4 TCAGGTGGGACTGCTGGAGTGGTCGTGCCTGCAGAACTGCGAGTACCTGTCCGCAGTGAT 3736

A3.1 TCAGGTGGGACTGCTGGAGTGGTCGTGCCTGCAGAACTGCGAGTACCTGTCCGCAGTGAT 4199

A3.4 TCAGGTGGGACTGCTGGAGTGGTCGTGCCTGCAGAACTGCGAGTACCTGTCCGCAGTGAT 4199

A3.6 TCAGGTGGGACTGCTGGAGTGGTCGTGCCTGCAGAACTGCGAGTACCTGTCCGCAGTGAT 4199

A3.7 TCAGGTGGGACTGCTGGAGTGGTCGTGCCTGCAGAACTGCGAGTACCTGTCCGCAGTGAT 4199

A3.8 TCAGGTGGGACTGCTGGAGTGGTCGTGCCTGCAGAACTGCGAGTACCTGTCCGCAGTGAT 4199

N3.1 TCAGGTGGGACTGCTGGAGTGGTCGTGCCTGCAGAACTGCGAGTACCTGTCCGCAGTGAT 3631

N3.6 TCAGGTGGGACTGCTGGAGTGGTCGTGCCTGCAGAACTGCGAGTACCTGTCCGCAGTGAT 3631

N3.3 TCAGGTGGGACTGCTGGAGTGGTCGTGCCTGCAGAACTGCGAGTACCTGTCCGCAGTGAT 3631

N3.10 TCAGGTGGGACTGCTGGAGTGGTCGTGCCTGCAGAACTGCGAGTACCTGTCCGCAGTGAT 3631

A1.3 TCAGGTGGGACTGCTGGAGTGGTCGTGCCTGCAGAACTGCGAGTACCTGTCCGCAGTGAT 3486

N1.2 TCAGGTGGGACTGCTGGAGTGGTCGTGCCTGCAGAACTGCGAGTACCTGTCCGCAGTGAT 3591

N1.5 TCAGGTGGGACTGCTGGAGTGGTCGTGCCTGCAGAACTGCGAGTACCTGTCCGCAGTGAT 3591

N1.27 TCAGGTGGGACTGCTGGAGTGGTCGTGCCTGCAGAACTGCGAGTACCTGTCCGCAGTGAT 3587

A1.2 ------------------------------------------------------------ 3714

A1.7 ------------------------------------------------------------ 3714

N3.9 TCAGGTGGGACTGCTGGAGTGGTCATGCCTGCAGAACTGCGAGTACCTGTCCGCAGTGAT 3908

MG637361.1 GCCCAACTTGACCGAATGCTCCATGGACGTTATGTGCCAGACGTTCTCACAATGTTGCAT 3968

A1.9 -----------------------TGTCAGT--GCTGTGTTCATCATGTTCTATATCAAGT 2975

N1.8 GCCCAACTTGACCGAATGCTCCATGGACGTTATGTGCCAGACGTTCTCACAATGTTGCAT 4115

A1.4 GCCCAACTTGACCGAATGCTCCATGGACGTTATGTGCCAGACGTTCTCACAATGTTGCAT 3796

A3.1 GCCCAACTTGACCGAATGCTCCATGGACGTTATGTGCCAGACGTTCTCACAATGTTGCAT 4259

A3.4 GCCCAACTTGACCGAATGCTCCATGGACGTTATGTGCCAGACGTTCTCACAATGTTGCAT 4259

A3.6 GCCCAACTTGACCGAATGCTCCATGGACGTTATGTGCCAGACGTTCTCACAATGTTGCAT 4259

A3.7 GCCCAACTTGACCGAATGCTCCATGGACGTTATGTGCCAGACGTTCTCACAATGTTGCAT 4259

A3.8 GCCCAACTTGACCGAATGCTCCATGGACGTTATGTGCCAGACGTTCTCACAATGTTGCAT 4259

N3.1 GCCCAACTTGACCGAATGCTCCATGGACGTTATGTGCCAGACGTTCTCACAATGTTGCAT 3691

N3.6 GCCCAACTTGACCGAATGCTCCATGGACGTTATGTGCCAGACGTTCTCACAATGTTGCAT 3691

N3.3 GCCCAACTTGACCGAATGCTCCATGGACGTTATGTGCCAGACGTTCTCACAATGTTGCAT 3691

N3.10 GCCCAACTTGACCGAATGCTCCATGGACGTTATGTGCCAGACGTTCTCACAATGTTGCAT 3691

A1.3 GCCCAACTTGACCGAATGCTCCATGGACGTTATGTGCCAGACGTTCTCACAATGTTGCAT 3546

N1.2 GCCCAACTTGACCGAATGCTCCATGGACGTTATGTGCCAGACGTTCTCACAATGTTGCAT 3651

N1.5 GCCCAACTTGACCGAATGCTCCATGGACGTTATGTGCCAGACGTTCTCACAATGTTGCAT 3651

N1.27 GCCCAACTTGACCGAATGCTCCATGGACGTTATGTGCCAGACGTTCTCACAATGTTGCAT 3647

A1.2 -----------------------------------------------------------T 3715

A1.7 -----------------------------------------------------------T 3715

N3.9 GCCCAACTTGACCGAATGCTCCATGGACGTAATGTGCCAGACGTTCTCACAATGTTGCAT 3968

*

MG637361.1 CCCAGACGATCCTTGGTTCATGTGGGATCACCCTGGAGTACTCCGCTACATAGTATGCAT 4028

A1.9 CCCAGACGATCCTTGGTTCATGTGGGATCACCCTGGAGTACTCCGCTACATAGTATGCAT 3035

N1.8 CCCAGACGATCCTTGGTTCATGTGGGATCACCCTGGAGTACTCCGCTACATAGTATGCAT 4175

A1.4 CCCAGACGATCCTTGGTTCATGTGGGATCACCCTGGAGTACTCCGCTACATAGTATGCAT 3856

A3.1 CCCAGACGATCCTTGGTTCATGTGGGATCACCCTGGAGTACTCCGCTACATAGTATGCAT 4319

A3.4 CCCAGACGATCCTTGGTTCATGTGGGATCACCCTGGAGTACTCCGCTACATAGTATGCAT 4319

A3.6 CCCAGACGATCCTTGGTTCATGTGGGATCACCCTGGAGTACTCCGCTACATAGTATGCAT 4319

A3.7 CCCAGACGATCCTTGGTTCATGTGGGATCACCCTGGAGTACTCCGCTACATAGTATGCAT 4319

A3.8 CCCAGACGATCCTTGGTTCATGTGGGATCACCCTGGAGTACTCCGCTACATAGTATGCAT 4319

N3.1 CCCAGACGATCCTTGGTTCATGTGGGATCACCCTGGAGTACTCCGCTACATAGTATGCAT 3751

N3.6 CCCAGACGATCCTTGGTTCATGTGGGATCACCCTGGAGTACTCCGCTACATAGTATGCAT 3751

N3.3 CCCAGACGATCCTTGGTTCATGTGGGATCACCCTGGAGTACTCCGCTACATAGTATGCAT 3751

N3.10 CCCAGACGATCCTTGGTTCATGTGGGATCACCCTGGAGTACTCCGCTACATAGTATGCAT 3751

A1.3 CCCAGACGATCCTTGGTTCATGTGGGATCACCCTGGAGTACTCCGCTACATAGTATGCAT 3606

N1.2 CCCAGACGATCCTTGGTTCATGTGGGATCACCCTGGAGTACTCCGCTACATAGTATGCAT 3711

N1.5 CCCAGACGATCCTTGGTTCATGTGGGATCACCCTGGAGTACTCCGCTACATAGTATGCAT 3711

N1.27 CCCAGACGATCCTTGGTTCATGTGGGATCACCCTGGAGTACTCCGCTACATAGTATGCAT 3707

A1.2 CCCTGACGATCCTTGGTTCATGTGGGATCACCCTGGAGTACTCCGCTACATAGTATGCAT 3775

A1.7 CCCTGACGATCCTTGGTTCATGTGGGATCACCCTGGAGTACTCCGCTACATAGTATGCAT 3775

N3.9 CCCTGACGATCCTTGGTTCATGTGGGATCACCCTGGAGTACTCCGCTACATAGTATGCAT 4028

*** ********************************************************

MG637361.1 GATCGTCAGTGGAGTTGTCATGTGGTTCGTACTCTTGATCGCCGAGTATCGATTGTTCCA 4088

A1.9 GATCGTCAGTGGAGTTGTCATGTGGTTCGTACTCTTGATCGCCGAGTATCGATTGTTCCA 3095

N1.8 GATCGTCAGTGGAGTTGTCATGTGGTTCGTACTCTTGATCGCCGAGTATCGATTGTTCCA 4235

A1.4 GATCGTCAGTGGAGTTGTCATGTGGTTCGTACTCTTGATCGCCGAGTATCGATTGTTCCA 3916

A3.1 GATCGTCAGTGGAGTTGTCATGTGGTTCGTACTCTTGATCGCCGAGTATCGATTGTTCCA 4379

A3.4 GATCGTCAGTGGAGTTGTCATGTGGTTCGTACTCTTGATCGCCGAGTATCGATTGTTCCA 4379

A3.6 GATCGTCAGTGGAGTTGTCATGTGGTTCGTACTCTTGATCGCCGAGTATCGATTGTTCCA 4379

A3.7 GATCGTCAGTGGAGTTGTCATGTGGTTCGTACTCTTGATCGCCGAGTATCGATTGTTCCA 4379

A3.8 GATCGTCAGTGGAGTTGTCATGTGGTTCGTACTCTTGATCGCCGAGTATCGATTGTTCCA 4379

N3.1 GATCGTCAGTGGAGTTGTCATGTGGTTCGTACTCTTGATCGCCGAGTATCGATTGTTCCA 3811

N3.6 GATCGTCAGTGGAGTTGTCATGTGGTTCGTACTCTTGATCGCCGAGTATCGATTGTTCCA 3811

N3.3 GATCGTCAGTGGAGTTGTCATGTGGTTCGTACTCTTGATCGCCGAGTATCGATTGTTCCA 3811

N3.10 GATCGTCAGTGGAGTTGTCATGTGGTTCGTACTCTTGATCGCCGAGTATCGATTGTTCCA 3811

A1.3 GATCGTCAGTGGAGTTGTCATGTGGTTCGTACTCTTGATCGCCGAGTATCGATTGTTCCA 3666

N1.2 GATCGTCAGTGGAGTTGTCATGTGGTTCGTACTCTTGATCGCCGAGTATCGATTGTTCCA 3771

N1.5 GATCGTCAGTGGAGTTGTCATGTGGTTCGTACTCTTGATCGCCGAGTATCGATTGTTCCA 3771

N1.27 GATCGTCAGTGGAGTTGTCATGTGGTTCGTACTCTTGATCGCCGAGTATCGATTGTTCCA 3767

A1.2 GATCGTCAGTGGAGTTGTCATGTGGTTCGTACTCTTGATCGCCGAGTATCGATTGTTCCA 3835

A1.7 GATCGTCAGTGGAGTTGTCATGTGGTTCGTACTCTTGATCGCCGAGTATCGATTGTTCCA 3835

N3.9 GATCGTCAGTGGAGTTGTCATGTGGTTCGTACTCTTGATCGCCGAGTATCGATTGTTCCA 4088

************************************************************

MG637361.1 GAAGGTGATCTACCGGGAAAAGAAAGCTCCTCCAGTTGATGAGAGCGCACTGGACAATGA 4148

A1.9 GAAGGTGATCTACCGGGAAAAGAAAGCTCCTCCAGTTGATGAGAGCGCACTGGACAATGA 3155

N1.8 GAAGGTGATCTACCGGGAAAAGAAAGCTCCTCCAGTTGATGAGAGCGCACTGGACAATGA 4295

A1.4 GAAGGTGATCTACCGGGAAAAGAAAGCTCCTCCAGTTGATGAGAGCGCACTGGACAATGA 3976

A3.1 GAAGGTGATCTACCGGGAAAAGAAAGCTCCTCCAGTTGATGAGAGCGCACTGGACAATGA 4439

A3.4 GAAGGTGATCTACCGGGAAAAGAAAGCTCCTCCAGTTGATGAGAGCGCACTGGACAATGA 4439

A3.6 GAAGGTGATCTACCGGGAAAAGAAAGCTCCTCCAGTTGATGAGAGCGCACTGGACAATGA 4439

A3.7 GAAGGTGATCTACCGGGAAAAGAAAGCTCCTCCAGTTGATGAGAGCGCACTGGACAATGA 4439

A3.8 GAAGGTGATCTACCGGGAAAAGAAAGCTCCTCCAGTTGATGAGAGCGCACTGGACAATGA 4439

N3.1 GAAGGTGATCTACCGGGAAAAGAAAGCTCCTCCAGTTGATGAGAGCGCACTGGACAATGA 3871

N3.6 GAAGGTGATCTACCGGGAAAAGAAAGCTCCTCCAGTTGATGAGAGCGCACTGGACAATGA 3871

N3.3 GAAGGTGATCTACCGGGAAAAGAAAGCTCCTCCAGTTGATGAGAGCGCACTGGACAATGA 3871

N3.10 GAAGGTGATCTACCGGGAAAAGAAAGCTCCTCCAGTTGATGAGAGCGCACTGGACAATGA 3871

A1.3 GAAGGTGATCTACCGGGAAAAGAAAGCTCCTCCAGTTGATGAGAGCGCACTGGACAATGA 3726

N1.2 GAAGGTGATCTACCGGGAAAAGAAAGCTCCTCCAGTTGATGAGAGCGCACTGGACAATGA 3831

N1.5 GAAGGTGATCTACCGGGAAAAGAAAGCTCCTCCAGTTGATGAGAGCGCACTGGACAATGA 3831

N1.27 GAAGGTGATCTACCGGGAAAAGAAAGCTCCTCCAGTTGATGAGAGCGCACTGGACAATGA 3827

A1.2 GAAGGTGATCTACCGGGAAAAGAAAGCTCCTCCAGTTGATGAGAGCGCACTGGACAATGA 3895

A1.7 GAAGGTGATCTACCGGGAAAAGAAAGCTCCTCCAGTTGATGAGAGCGCACTGGACAATGA 3895

N3.9 GAAGGTGATCTACCGGGAAAAGAAAGCTCCTCCAGTTGATGAGAGCGCACTGGACAATGA 4148

************************************************************

MG637361.1 CGTGGCGGACGAGGCCAGACACGTGGCGCGAGTTGGAGCAGGAGCAATCCTCGGGCAGCA 4208

A1.9 CGTGGCGGACGAGGCCAGACACGTGGCGCGAGTTGGAGCAGGAGCAATCCTCGGGCAGCA 3215

N1.8 CGTGGCGGACGAGGCCAGACACGTGGCGCGAGTTGGAGCAGGAGCAATCCTCGGGCAGCA 4355

A1.4 CGTGGCGGACGAGGCCAGACACGTGGCGCGAGTTGGAGCAGGAGCAATCCTCGGGCAGCA 4036

A3.1 CGTGGCGGACGAGGCCAGACACGTGGCGCGAGTTGGAGCAGGAGCAATCCTCGGGCAGCA 4499

A3.4 CGTGGCGGACGAGGCCAGACACGTGGCGCGAGTTGGAGCAGGAGCAATCCTCGGGCAGCA 4499

A3.6 CGTGGCGGACGAGGCCAGACACGTGGCGCGAGTTGGAGCAGGAGCAATCCTCGGGCAGCA 4499

A3.7 CGTGGCGGACGAGGCCAGACACGTGGCGCGAGTTGGAGCAGGAGCAATCCTCGGGCAGCA 4499

A3.8 CGTGGCGGACGAGGCCAGACACGTGGCGCGAGTTGGAGCAGGAGCAATCCTCGGGCAGCA 4499

N3.1 CGTGGCGGACGAGGCCAGACACGTGGCGCGAGTTGGAGCAGGAGCAATCCTCGGGCAGCA 3931

N3.6 CGTGGCGGACGAGGCCAGACACGTGGCGCGAGTTGGAGCAGGAGCAATCCTCGGGCAGCA 3931

N3.3 CGTGGCGGACGAGGCCAGACACGTGGCGCGAGTTGGAGCAGGAGCAATCCTCGGGCAGCA 3931

N3.10 CGTGGCGGACGAGGCCAGACACGTGGCGCGAGTTGGAGCAGGAGCAATCCTCGGGCAGCA 3931

A1.3 CGTGGCGGACGAGGCCAGACACGTGGCGCGAGTTGGAGCAGGAGCAATCCTCGGGCAGCA 3786

N1.2 CGTGGCGGACGAGGCCAGACACGTGGCGCGAGTTGGAGCAGGAGCAATCCTCGGGCAGCA 3891

N1.5 CGTGGCGGACGAGGCCAGACACGTGGCGCGAGTTGGAGCAGGAGCAATCCTCGGGCAGCA 3891

N1.27 CGTGGCGGACGAGGCCAGACACGTGGCGCGAGTTGGAGCAGGAGCAATCCTCGGGCAGCA 3887

A1.2 CGTGGCGGACGAGGCCAGACACGTGGCGCGAGTTGGAGCAGGAGCAATCCTCGGGCAGCA 3955

A1.7 CGTGGCGGACGAGGCCAGACACGTGGCGCGAGTTGGAGCAGGAGCAATCCTCGGGCAGCA 3955

N3.9 CGTGGCGGACGAGGCCAGACACGTGGCGCGAGTTGGAGCAGGAGCAATCCTCGGGCAGCA 4208

************************************************************

MG637361.1 CAGCCTAGTAGCAAATGGCCTCACCAAGTATTATGGGAAACACCTTGCAGTCAATCAAGT 4268

A1.9 CAGCCTAGTAGCAAATGGCCTCACCAAGTATTATGGGAAACACCTTGCAGTCAATCAAGT 3275

N1.8 CAGCCTAGTAGCAAATGGCCTCACCAAGTATTATGGGAAACACCTTGCAGTCAATCAAGT 4415

A1.4 CAGCCTAGTAGCAAATGGCCTCACCAAGTATTATGGGAAACACCTTGCAGTCAATCAAGT 4096

A3.1 CAGCCTAGTAGCAAATGGCCTCACCAAGTATTATGGGAAACACCTTGCAGTCAATCAAGT 4559

A3.4 CAGCCTAGTAGCAAATGGCCTCACCAAGTATTATGGGAAACACCTTGCAGTCAATCAAGT 4559

A3.6 CAGCCTAGTAGCAAATGGCCTCACCAAGTATTATGGGAAACACCTTGCAGTCAATCAAGT 4559

A3.7 CAGCCTAGTAGCAAATGGCCTCACCAAGTATTATGGGAAACACCTTGCAGTCAATCAAGT 4559

A3.8 CAGCCTAGTAGCAAATGGCCTCACCAAGTATTATGGGAAACACCTTGCAGTCAATCAAGT 4559

N3.1 CAGCCTAGTAGCAAATGGCCTCACCAAGTATTATGGGAAACACCTTGCAGTCAATCAAGT 3991

N3.6 CAGCCTAGTAGCAAATGGCCTCACCAAGTATTATGGGAAACACCTTGCAGTCAATCAAGT 3991

N3.3 CAGCCTAGTAGCAAATGGCCTCACCAAGTATTATGGGAAACACCTTGCAGTCAATCAAGT 3991

N3.10 CAGCCTAGTAGCAAATGGCCTCACCAAGTATTATGGGAAACACCTTGCAGTCAATCAAGT 3991

A1.3 CAGCCTAGTAGCAAATGGCCTCACCAAGTATTATGGGAAACACCTTGCAGTCAATCAAGT 3846

N1.2 CAGCCTAGTAGCAAATGGCCTCACCAAGTATTATGGGAAACACCTTGCAGTCAATCAAGT 3951

N1.5 CAGCCTAGTAGCAAATGGCCTCACCAAGTATTATGGGAAACACCTTGCAGTCAATCAAGT 3951

N1.27 CAGCCTAGTAGCAAATGGCCTCACCAAGTATTATGGGAAACACCTTGCAGTCAATCAAGT 3947

A1.2 CAGCCTAGTAGCAAATGGCCTCACCAAGTATTATGGGAAACACCTTGCAGTCAATCAAGT 4015

A1.7 CAGCCTAGTAGCAAATGGCCTCACCAAGTATTATGGGAAACACCTTGCAGTCAATCAAGT 4015

N3.9 CAGCCTAGTAGCAAATGGCCTCACCAAGTATTATGGGAAACACCTTGCAGTCAATCAAGT 4268

************************************************************

MG637361.1 GTCATTCACCGTGGGCGACACGGAATGCTTTGGTCTTCTGGGTGTGAACGGCGCCGGTAA 4328

A1.9 GTCATTCACCGTGGGCGACACGGAATGCTTTGGTCTTCTGGGTGTGAACGGCGCCGGTAA 3335

N1.8 GTCATTCACCGTGGGCGACACGGAATGCTTTGGTCTTCTGGGTGTGAACGGCGCCGGTAA 4475

A1.4 GTCATTCACCGTGGGCGACACGGAATGCTTTGGTCTTCTGGGTGTGAACGGCGCCGGTAA 4156

A3.1 GTCATTCACCGTGGGCGACACGGAATGCTTTGGTCTTCTGGGTGTGAACGGCGCCGGTAA 4619

A3.4 GTCATTCACCGTGGGCGACACGGAATGCTTTGGTCTTCTGGGTGTGAACGGCGCCGGTAA 4619

A3.6 GTCATTCACCGTGGGCGACACGGAATGCTTTGGTCTTCTGGGTGTGAACGGCGCCGGTAA 4619

A3.7 GTCATTCACCGTGGGCGACACGGAATGCTTTGGTCTTCTGGGTGTGAACGGCGCCGGTAA 4619

A3.8 GTCATTCACCGTGGGCGACACGGAATGCTTTGGTCTTCTGGGTGTGAACGGCGCCGGTAA 4619

N3.1 GTCATTCACCGTGGGCGACACGGAATGCTTTGGTCTTCTGGGTGTGAACGGCGCCGGTAA 4051

N3.6 GTCATTCACCGTGGGCGACACGGAATGCTTTGGTCTTCTGGGTGTGAACGGCGCCGGTAA 4051

N3.3 GTCATTCACCGTGGGCGACACGGAATGCTTTGGTCTTCTGGGTGTGAACGGCGCCGGTAA 4051

N3.10 GTCATTCACCGTGGGCGACACGGAATGCTTTGGTCTTCTGGGTGTGAACGGCGCCGGTAA 4051

A1.3 GTCATTCACCGTGGGCGACACGGAATGCTTTGGTCTTCTGGGTGTGAACGGCGCCGGTAA 3906

N1.2 GTCATTCACCGTGGGCGACACGGAATGCTTTGGTCTTCTGGGTGTGAACGGCGCCGGTAA 4011

N1.5 GTCATTCACCGTGGGCGACACGGAATGCTTTGGTCTTCTGGGTGTGAACGGCGCCGGTAA 4011

N1.27 GTCATTCACCGTGGGCGACACGGAATGCTTTGGTCTTCTGGGTGTGAACGGCGCCGGTAA 4007

A1.2 GTCATTCACCGTGGGCGACACGGAATGCTTTGGTCTTCTGGGTGTGAACGGCGCCGGTAA 4075

A1.7 GTCATTCACCGTGGGCGACACGGAATGCTTTGGTCTTCTGGGTGTGAACGGCGCCGGTAA 4075

N3.9 GTCATTCACCGTGGGCGACACGGAATGCTTTGGTCTTCTGGGTGTGAACGGCGCCGGTAA 4328

************************************************************

MG637361.1 GACGACCACCTTCAAGATGTTGATGGGAGATGAGACCGTCTCCAGCGGAGATGCCTTCGT 4388

A1.9 GACGACCACCTTCAAGATGTTGATGGGAGATGAGACCGTCTCCAGCGGAGATGCCTTCGT 3395

N1.8 GACGACCACCTTCAAGATGTTGATGGGAGATGAGACCGTCTCCAGCGGAGATGCCTTCGT 4535

A1.4 GACGACCACCTTCAAGATGTTGATGGGAGATGAGACCGTCTCCAGCGGAGATGCCTTCGT 4216

A3.1 GACGACCACCTTCAAGATGTTGATGGGAGATGAGACCGTCTCCAGCGGAGATGCCTTCGT 4679

A3.4 GACGACCACCTTCAAGATGTTGATGGGAGATGAGACCGTCTCCAGCGGAGATGCCTTCGT 4679

A3.6 GACGACCACCTTCAAGATGTTGATGGGAGATGAGACCGTCTCCAGCGGAGATGCCTTCGT 4679

A3.7 GACGACCACCTTCAAGATGTTGATGGGAGATGAGACCGTCTCCAGCGGAGATGCCTTCGT 4679

A3.8 GACGACCACCTTCAAGATGTTGATGGGAGATGAGACCGTCTCCAGCGGAGATGCCTTCGT 4679

N3.1 GACGACCACCTTCAAGATGTTGATGGGAGATGAGACCGTCTCCAGCGGAGATGCCTTCGT 4111

N3.6 GACGACCACCTTCAAGATGTTGATGGGAGATGAGACCGTCTCCAGCGGAGATGCCTTCGT 4111

N3.3 GACGACCACCTTCAAGATGTTGATGGGAGATGAGACCGTCTCCAGCGGAGATGCCTTCGT 4111

N3.10 GACGACCACCTTCAAGATGTTGATGGGAGATGAGACCGTCTCCAGCGGAGATGCCTTCGT 4111

A1.3 GACGACCACCTTCAAGATGTTGATGGGAGATGAGACCGTCTCCAGCGGAGATGCCTTCGT 3966

N1.2 GACGACCACCTTCAAGATGTTGATGGGAGATGAGACCGTCTCCAGCGGAGATGCCTTCGT 4071

N1.5 GACGACCACCTTCAAGATGTTGATGGGAGATGAGACCGTCTCCAGCGGAGATGCCTTCGT 4071

N1.27 GACGACCACCTTCAAGATGTTGATGGGAGATGAGACCGTCTCCAGCGGAGATGCCTTCGT 4067

A1.2 GACGACCACCTTCAAGATGTTGATGGGAGATGAGACCGTCTCCAGCGGAGATGCCTTCGT 4135

A1.7 GACGACCACCTTCAAGATGTTGATGGGAGATGAGACCGTCTCCAGCGGAGATGCCTTCGT 4135

N3.9 GACGACCACCTTCAAGATGTTGATGGGAGATGAGACCGTCTCCAGCGGAGATGCCTTCGT 4388

************************************************************

MG637361.1 GAGTGGCCATTCTGTCAAGACTAATATCACTCAAGTTTACAAAAATATTGGTTACTGTCC 4448

A1.9 GAGTGGCCATTCTGTCAAGACTAATATCACTCAAGTTTACAAAAATATTGGTTACTGTCC 3455

N1.8 GAGTGGCCATTCTGTCAAGACTAATATCACTCAAGTTTACAAAAATATTGGTTACTGTCC 4595

A1.4 GAGTGGCCATTCTGTCAAGACTAATATCACTCAAGTTTACAAAAATATTGGTTACTGTCC 4276

A3.1 GAGTGGCCATTCTGTCAAGACTAATATCACTCAAGTTTACAAAAATATTGGTTACTGTCC 4739

A3.4 GAGTGGCCATTCTGTCAAGACTAATATCACTCAAGTTTACAAAAATATTGGTTACTGTCC 4739

A3.6 GAGTGGCCATTCTGTCAAGACTAATATCACTCAAGTTTACAAAAATATTGGTTACTGTCC 4739

A3.7 GAGTGGCCATTCTGTCAAGACTAATATCACTCAAGTTTACAAAAATATTGGTTACTGTCC 4739

A3.8 GAGTGGCCATTCTGTCAAGACTAATATCACTCAAGTTTACAAAAATATTGGTTACTGTCC 4739

N3.1 GAGTGGCCATTCTGTCAAGACTAATATCACTCAAGTTTACAAAAATATTGGTTACTGTCC 4171

N3.6 GAGTGGCCATTCTGTCAAGACTAATATCACTCAAGTTTACAAAAATATTGGTTACTGTCC 4171

N3.3 GAGTGGCCATTCTGTCAAGACTAATATCACTCAAGTTTACAAAAATATTGGTTACTGTCC 4171

N3.10 GAGTGGCCATTCTGTCAAGACTAATATCACTCAAGTTTACAAAAATATTGGTTACTGTCC 4171

A1.3 GAGTGGCCATTCTGTCAAGACTAATATCACTCAAGTTTACAAAAATATTGGTTACTGTCC 4026

N1.2 GAGTGGCCATTCTGTCAAGACTAATATCACTCAAGTTTACAAAAATATTGGTTACTGTCC 4131

N1.5 GAGTGGCCATTCTGTCAAGACTAATATCACTCAAGTTTACAAAAATATTGGTTACTGTCC 4131

N1.27 GAGTGGCCATTCTGTCAAGACTAATATCACTCAAGTTTACAAAAATATTGGTTACTGTCC 4127

A1.2 GAGTGGCCATTCTGTCAAGACTAATATCACTCAAGTTTACAAAAATATTGGTTACTGTCC 4195

A1.7 GAGTGGCCATTCTGTCAAGACTAATATCACTCAAGTTTACAAAAATATTGGTTACTGTCC 4195

N3.9 GAGTGGCCATTCTGTCAAGACTAATATCACTCAAGTTTACAAAAATATTGGTTACTGTCC 4448

************************************************************

MG637361.1 GCAATTCGAAGCGACATTCGGCGAGCTGACGGGACGCGAGACACTACGGCTGTTCTCGGC 4508

A1.9 GCAATTCGAAGCGACATTCGGCGAGCTGACGGGACGCGAGACACTACGGCTGTTCTCGGC 3515

N1.8 GCAATTCGAAGCGACATTCGGCGAGCTGACGGGACGCGAGACACTACGGCTGTTCTCGGC 4655

A1.4 GCAATTCGAAGCGACATTCGGCGAGCTGACGGGACGCGAGACACTACGGCTGTTCTCGGC 4336

A3.1 GCAATTCGAAGCGACATTCGGCGAGCTGACGGGACGCGAGACACTACGGCTGTTCTCGGC 4799

A3.4 GCAATTCGAAGCGACATTCGGCGAGCTGACGGGACGCGAGACACTACGGCTGTTCTCGGC 4799

A3.6 GCAATTCGAAGCGACATTCGGCGAGCTGACGGGACGCGAGACACTACGGCTGTTCTCGGC 4799

A3.7 GCAATTCGAAGCGACATTCGGCGAGCTGACGGGACGCGAGACACTACGGCTGTTCTCGGC 4799

A3.8 GCAATTCGAAGCGACATTCGGCGAGCTGACGGGACGCGAGACACTACGGCTGTTCTCGGC 4799

N3.1 GCAATTCGAAGCGACATTCGGCGAGCTGACGGGACGCGAGACACTACGGCTGTTCTCGGC 4231

N3.6 GCAATTCGAAGCGACATTCGGCGAGCTGACGGGACGCGAGACACTACGGCTGTTCTCGGC 4231

N3.3 GCAATTCGAAGCGACATTCGGCGAGCTGACGGGACGCGAGACACTACGGCTGTTCTCGGC 4231

N3.10 GCAATTCGAAGCGACATTCGGCGAGCTGACGGGACGCGAGACACTACGGCTGTTCTCGGC 4231

A1.3 GCAATTCGAAGCGACATTCGGCGAGCTGACGGGACGCGAGACACTACGGCTGTTCTCGGC 4086

N1.2 GCAATTCGAAGCGACATTCGGCGAGCTGACGGGACGCGAGACACTACGGCTGTTCTCGGC 4191

N1.5 GCAATTCGAAGCGACATTCGGCGAGCTGACGGGACGCGAGACACTACGGCTGTTCTCGGC 4191

N1.27 GCAATTCGAAGCGACATTCGGCGAGCTGACGGGACGCGAGACACTACGGCTGTTCTCGGC 4187

A1.2 GCAATTCGAAGCGACATTCGGCGAGCTGACGGGACGCGAGACACTACGGCTGTTCTCGGC 4255

A1.7 GCAATTCGAAGCGACATTCGGCGAGCTGACGGGACGCGAGACACTACGGCTGTTCTCGGC 4255

N3.9 GCAATTCGAAGCGACATTCGGCGAGCTGACGGGACGCGAGACACTACGGCTGTTCTCGGC 4508

************************************************************

MG637361.1 GCTGCGAGGGTTGCCAGTGCGAGGCGCCACGCTCCACGCGGAGGCCTTAGCACATGCTCT 4568

A1.9 GCTGCGAGGGTTGCCAGTGCGAGGCGCCACGCTCCACGCGGAGGCCTTAGCACATGCTCT 3575

N1.8 GCTGCGAGGGTTGCCAGTGCGAGGCGCCACGCTCCACGCGGAGGCCTTAGCACATGCTCT 4715

A1.4 GCTGCGAGGGTTGCCAGTGCGAGGCGCCACGCTCCACGCGGAGGCCTTAGCACATGCTCT 4396

A3.1 GCTGCGAGGGTTGCCAGTGCGAGGCGCCACGCTCCACGCGGAGGCCTTAGCACATGCTCT 4859

A3.4 GCTGCGAGGGTTGCCAGTGCGAGGCGCCACGCTCCACGCGGAGGCCTTAGCACATGCTCT 4859

A3.6 GCTGCGAGGGTTGCCAGTGCGAGGCGCCACGCTCCACGCGGAGGCCTTAGCACATGCTCT 4859

A3.7 GCTGCGAGGGTTGCCAGTGCGAGGCGCCACGCTCCACGCGGAGGCCTTAGCACATGCTCT 4859

A3.8 GCTGCGAGGGTTGCCAGTGCGAGGCGCCACGCTCCACGCGGAGGCCTTAGCACATGCTCT 4859

N3.1 GCTGCGAGGGTTGCCAGTGCGAGGCGCCACGCTCCACGCGGAGGCCTTAGCACATGCTCT 4291

N3.6 GCTGCGAGGGTTGCCAGTGCGAGGCGCCACGCTCCACGCGGAGGCCTTAGCACATGCTCT 4291

N3.3 GCTGCGAGGGTTGCCAGTGCGAGGCGCCACGCTCCACGCGGAGGCCTTAGCACATGCTCT 4291

N3.10 GCTGCGAGGGTTGCCAGTGCGAGGCGCCACGCTCCACGCGGAGGCCTTAGCACATGCTCT 4291

A1.3 GCTGCGAGGGTTGCCAGTGCGAGGCGCCACGCTCCACGCGGAGGCCTTAGCACATGCTCT 4146

N1.2 GCTGCGAGGGTTGCCAGTGCGAGGCGCCACGCTCCACGCGGAGGCCTTAGCACATGCTCT 4251

N1.5 GCTGCGAGGGTTGCCAGTGCGAGGCGCCACGCTCCACGCGGAGGCCTTAGCACATGCTCT 4251

N1.27 GCTGCGAGGGTTGCCAGTGCGAGGCGCCACGCTCCACGCGGAGGCCTTAGCACATGCTCT 4247

A1.2 GCTGCGAGGGTTGCCAGTGCGAGGCGCCACGCTCCACGCGGAGGCCTTAGCACATGCTCT 4315

A1.7 GCTGCGAGGGTTGCCAGTGCGAGGCGCCACGCTCCACGCGGAGGCCTTAGCACATGCTCT 4315

N3.9 GCTGCGAGGGTTGCCAGTGCGAGGCGCCACGCTCCACGCGGAGGCCTTAGCACATGCTCT 4568

************************************************************

MG637361.1 TGGTTTCTATAAGCATCTTGATAAAAGGGTGGACCACTACTCTGGTGGCAACAAGCGCAA 4628

A1.9 TGGTTTCTATAAGCATCTTGATAAAAGGGTGGACCACTACTCTGGTGGCAACAAGCGCAA 3635

N1.8 TGGTTTCTATAAGCATCTTGATAAAAGGGTGGACCACTACTCTGGTGGCAACAAGCGCAA 4775

A1.4 TGGTTTCTATAAGCATCTTGATAAAAGGGTGGACCACTACTCTGGTGGCAACAAGCGCAA 4456

A3.1 TGGTTTCTATAAGCATCTTGATAAAAGGGTGGACCACTACTCTGGTGGCAACAAGCGCAA 4919

A3.4 TGGTTTCTATAAGCATCTTGATAAAAGGGTGGACCACTACTCTGGTGGCAACAAGCGCAA 4919

A3.6 TGGTTTCTATAAGCATCTTGATAAAAGGGTGGACCACTACTCTGGTGGCAACAAGCGCAA 4919

A3.7 TGGTTTCTATAAGCATCTTGATAAAAGGGTGGACCACTACTCTGGTGGCAACAAGCGCAA 4919

A3.8 TGGTTTCTATAAGCATCTTGATAAAAGGGTGGACCACTACTCTGGTGGCAACAAGCGCAA 4919

N3.1 TGGTTTCTATAAGCATCTTGATAAAAGGGTGGACCACTACTCTGGTGGCAACAAGCGCAA 4351

N3.6 TGGTTTCTATAAGCATCTTGATAAAAGGGTGGACCACTACTCTGGTGGCAACAAGCGCAA 4351

N3.3 TGGTTTCTATAAGCATCTTGATAAAAGGGTGGACCACTACTCTGGTGGCAACAAGCGCAA 4351

N3.10 TGGTTTCTATAAGCATCTTGATAAAAGGGTGGACCACTACTCTGGTGGCAACAAGCGCAA 4351

A1.3 TGGTTTCTATAAGCATCTTGATAAAAGGGTGGACCACTACTCTGGTGGCAACAAGCGCAA 4206

N1.2 TGGTTTCTATAAGCATCTTGATAAAAGGGTGGACCACTACTCTGGTGGCAACAAGCGCAA 4311

N1.5 TGGTTTCTATAAGCATCTTGATAAAAGGGTGGACCACTACTCTGGTGGCAACAAGCGCAA 4311

N1.27 TGGTTTCTATAAGCATCTTGATAAAAGGGTGGACCACTACTCTGGTGGCAACAAGCGCAA 4307

A1.2 TGGTTTCTATAAGCATCTTGATAAAAGGGTGGACCACTACTCTGGTGGCAACAAGCGCAA 4375

A1.7 TGGTTTCTATAAGCATCTTGATAAAAGGGTGGACCACTACTCTGGTGGCAACAAGCGCAA 4375

N3.9 TGGTTTCTATAAGCATCTTGATAAAAGGGTGGACCACTACTCTGGTGGCAACAAGCGCAA 4628

************************************************************

MG637361.1 GTTGAGCACGGCTGTGGCGTTGCTGGGGCGCACGCGGCTTATATTCGTCGACGAACCCAC 4688

A1.9 GTTGAGCACGGCTGTGGCGTTGCTGGGGCGCACGCGGCTTATATTCGTCGACGAACCCAC 3695

N1.8 GTTGAGCACGGCTGTGGCGTTGCTGGGGCGCACGCGGCTTATATTCGTCGACGAACCCAC 4835

A1.4 GTTGAGCACGGCTGTGGCGTTGCTGGGGCGCACGCGGCTTATATTCGTCGACGAACCCAC 4516

A3.1 GTTGAGCACGGCTGTGGCGTTGCTGGGGCGCACGCGGCTTATATTCGTCGACGAACCCAC 4979

A3.4 GTTGAGCACGGCTGTGGCGTTGCTGGGGCGCACGCGGCTTATATTCGTCGACGAACCCAC 4979

A3.6 GTTGAGCACGGCTGTGGCGTTGCTGGGGCGCACGCGGCTTATATTCGTCGACGAACCCAC 4979

A3.7 GTTGAGCACGGCTGTGGCGTTGCTGGGGCGCACGCGGCTTATATTCGTCGACGAACCCAC 4979

A3.8 GTTGAGCACGGCTGTGGCGTTGCTGGGGCGCACGCGGCTTATATTCGTCGACGAACCCAC 4979

N3.1 GTTGAGCACGGCTGTGGCGTTGCTGGGGCGCACGCGGCTTATATTCGTCGACGAACCCAC 4411

N3.6 GTTGAGCACGGCTGTGGCGTTGCTGGGGCGCACGCGGCTTATATTCGTCGACGAACCCAC 4411

N3.3 GTTGAGCACGGCTGTGGCGTTGCTGGGGCGCACGCGGCTTATATTCGTCGACGAACCCAC 4411

N3.10 GTTGAGCACGGCTGTGGCGTTGCTGGGGCGCACGCGGCTTATATTCGTCGACGAACCCAC 4411

A1.3 GTTGAGCACGGCTGTGGCGTTGCTGGGGCGCACGCGGCTTATATTCGTCGACGAACCCAC 4266

N1.2 GTTGAGCACGGCTGTGGCGTTGCTGGGGCGCACGCGGCTTATATTCGTCGACGAACCCAC 4371

N1.5 GTTGAGCACGGCTGTGGCGTTGCTGGGGCGCACGCGGCTTATATTCGTCGACGAACCCAC 4371

N1.27 GTTGAGCACGGCTGTGGCGTTGCTGGGGCGCACGCGGCTTATATTCGTCGACGAACCCAC 4367

A1.2 GTTGAGCACGGCTGTGGCGTTGCTTGGGCGCACGCGGCTTATATTCGTCGACGAACCCAC 4435

A1.7 GTTGAGCACGGCTGTGGCGTTGCTTGGGCGCACGCGGCTTATATTCGTCGACGAACCCAC 4435

N3.9 GTTGAGCACGGCTGTGGCGTTGCTTGGGCGCACGCGGCTTATATTCGTCGACGAACCCAC 4688

************************ ***********************************

MG637361.1 TACTGGAGTCGATCCTGCTGCTAAGAGACAGATGTGGAACGCGGTTCGAGAAGCTCGCCG 4748

A1.9 TACTGGAGTCGATCCTGCTGCTAAGAGACAGATGTGGAACGCGGTTCGAGAAGCTCGCCG 3755

N1.8 TACTGGAGTCGATCCTGCTGCTAAGAGACAGATGTGGAACGCGGTTCGAGAAGCTCGCCG 4895

A1.4 TACTGGAGTCGATCCTGCTGCTAAGAGACAGATGTGGAACGCGGTTCGAGAAGCTCGCCG 4576

A3.1 TACTGGAGTCGATCCTGCTGCTAAGAGACAGATGTGGAACGCGGTTCGAGAAGCTCGCCG 5039

A3.4 TACTGGAGTCGATCCTGCTGCTAAGAGACAGATGTGGAACGCGGTTCGAGAAGCTCGCCG 5039

A3.6 TACTGGAGTCGATCCTGCTGCTAAGAGACAGATGTGGAACGCGGTTCGAGAAGCTCGCCG 5039

A3.7 TACTGGAGTCGATCCTGCTGCTAAGAGACAGATGTGGAACGCGGTTCGAGAAGCTCGCCG 5039

A3.8 TACTGGAGTCGATCCTGCTGCTAAGAGACAGATGTGGAACGCGGTTCGAGAAGCTCGCCG 5039

N3.1 TACTGGAGTCGATCCTGCTGCTAAGAGACAGATGTGGAACGCGGTTCGAGAAGCTCGCCG 4471

N3.6 TACTGGAGTCGATCCTGCTGCTAAGAGACAGATGTGGAACGCGGTTCGAGAAGCTCGCCG 4471

N3.3 TACTGGAGTCGATCCTGCTGCTAAGAGACAGATGTGGAACGCGGTTCGAGAAGCTCGCCG 4471

N3.10 TACTGGAGTCGATCCTGCTGCTAAGAGACAGATGTGGAACGCGGTTCGAGAAGCTCGCCG 4471

A1.3 TACTGGAGTCGATCCTGCTGCTAAGAGACAGATGTGGAACGCGGTTCGAGAAGCTCGCCG 4326

N1.2 TACTGGAGTCGATCCTGCTGCTAAGAGACAGATGTGGAACGCGGTTCGAGAAGCTCGCCG 4431

N1.5 TACTGGAGTCGATCCTGCTGCTAAGAGACAGATGTGGAACGCGGTTCGAGAAGCTCGCCG 4431

N1.27 TACTGGAGTCGATCCTGCTGCTAAGAGACAGATGTGGAACGCGGTTCGAGAAGCTCGCCG 4427

A1.2 TACTGGAGTCGATCCCGCTGCTAAGAGACAGATGTGGAACGCGGTTCGAGAAGCTCGCCG 4495

A1.7 TACTGGAGTCGATCCCGCTGCTAAGAGACAGATGTGGAACGCGGTTCGAGAAGCTCGCCG 4495

N3.9 TACTGGAGTCGATCCCGCTGCTAAGAGACAGATGTGGAACGCGGTTCGAGAAGCTCGCCG 4748

*************** ********************************************

MG637361.1 GTCGGGTCGTGGTGTGGTGCTGACATCACACAGCATGGAGGAGTGTGAGGCTCTGTGCTC 4808

A1.9 GTCGGGTCGTGGTGTGGTGCTGACATCACACAGCATGGAGGAGTGTGAGGCTCTGTGCTC 3815

N1.8 GTCGGGTCGTGGTGTGGTGCTGACATCACACAGCATGGAGGAGTGTGAGGCTCTGTGCTC 4955

A1.4 GTCGGGTCGTGGTGTGGTGCTGACATCACACAGCATGGAGGAGTGTGAGGCTCTGTGCTC 4636

A3.1 GTCGGGTCGTGGTGTGGTGCTGACATCACACAGCATGGAGGAGTGTGAGGCTCTGTGCTC 5099

A3.4 GTCGGGTCGTGGTGTGGTGCTGACATCACACAGCATGGAGGAGTGTGAGGCTCTGTGCTC 5099

A3.6 GTCGGGTCGTGGTGTGGTGCTGACATCACACAGCATGGAGGAGTGTGAGGCTCTGTGCTC 5099

A3.7 GTCGGGTCGTGGTGTGGTGCTGACATCACACAGCATGGAGGAGTGTGAGGCTCTGTGCTC 5099

A3.8 GTCGGGTCGTGGTGTGGTGCTGACATCACACAGCATGGAGGAGTGTGAGGCTCTGTGCTC 5099

N3.1 GTCGGGTCGTGGTGTGGTGCTGACATCACACAGCATGGAGGAGTGTGAGGCTCTGTGCTC 4531

N3.6 GTCGGGTCGTGGTGTGGTGCTGACATCACACAGCATGGAGGAGTGTGAGGCTCTGTGCTC 4531

N3.3 GTCGGGTCGTGGTGTGGTGCTGACATCACACAGCATGGAGGAGTGTGAGGCTCTGTGCTC 4531

N3.10 GTCGGGTCGTGGTGTGGTGCTGACATCACACAGCATGGAGGAGTGTGAGGCTCTGTGCTC 4531

A1.3 GTCGGGTCGTGGTGTGGTGCTGACATCACACAGCATGGAGGAGTGTGAGGCTCTGTGCTC 4386

N1.2 GTCGGGTCGTGGTGTGGTGCTGACATCACACAGCATGGAGGAGTGTGAGGCTCTGTGCTC 4491

N1.5 GTCGGGTCGTGGTGTGGTGCTGACATCACACAGCATGGAGGAGTGTGAGGCTCTGTGCTC 4491

N1.27 GTCGGGTCGTGGTGTGGTGCTGACATCACACAGCATGGAGGAGTGTGAGGCTCTGTGCTC 4487

A1.2 GTCGGGTCGTGGTGTGGTGCTGACATCACACAGCATGGAGGAGTGTGAGGCTCTGTGCTC 4555

A1.7 GTCGGGTCGTGGTGTGGTGCTGACATCACACAGCATGGAGGAGTGTGAGGCTCTGTGCTC 4555

N3.9 GTCGGGTCGTGGTGTGGTGCTGACATCACACAGCATGGAGGAGTGTGAGGCTCTGTGCTC 4808

************************************************************

MG637361.1 GCGGCTCACAATCATGGTCAACGGACAGTTCCAGTGCCTCGGCACGCCGCAACATTTAAA 4868

A1.9 GCGGCTCACAATCATGGTCAACGGACAGTTCCAGTGCCTCGGCACGCCGCAACATTTAAA 3875

N1.8 GCGGCTCACAATCATGGTCAACGGACAGTTCCAGTGCCTCGGCACGCCGCAACATTTAAA 5015

A1.4 GCGGCTCACAATCATGGTCAACGGACAGTTCCAGTGCCTCGGCACGCCGCAACATTTAAA 4696

A3.1 GCGGCTCACAATCATGGTCAACGGACAGTTCCAGTGCCTCGGCACGCCGCAACATTTAAA 5159

A3.4 GCGGCTCACAATCATGGTCAACGGACAGTTCCAGTGCCTCGGCACGCCGCAACATTTAAA 5159

A3.6 GCGGCTCACAATCATGGTCAACGGACAGTTCCAGTGCCTCGGCACGCCGCAACATTTAAA 5159

A3.7 GCGGCTCACAATCATGGTCAACGGACAGTTCCAGTGCCTCGGCACGCCGCAACATTTAAA 5159

A3.8 GCGGCTCACAATCATGGTCAACGGACAGTTCCAGTGCCTCGGCACGCCGCAACATTTAAA 5159

N3.1 GCGGCTCACAATCATGGTCAACGGACAGTTCCAGTGCCTCGGCACGCCGCAACATTTAAA 4591

N3.6 GCGGCTCACAATCATGGTCAACGGACAGTTCCAGTGCCTCGGCACGCCGCAACATTTAAA 4591

N3.3 GCGGCTCACAATCATGGTCAACGGACAGTTCCAGTGCCTCGGCACGCCGCAACATTTAAA 4591

N3.10 GCGGCTCACAATCATGGTCAACGGACAGTTCCAGTGCCTCGGCACGCCGCAACATTTAAA 4591

A1.3 GCGGCTCACAATCATGGTCAACGGACAGTTCCAGTGCCTCGGCACGCCGCAACATTTAAA 4446

N1.2 GCGGCTCACAATCATGGTCAACGGACAGTTCCAGTGCCTCGGCACGCCGCAACATTTAAA 4551

N1.5 GCGGCTCACAATCATGGTCAACGGACAGTTCCAGTGCCTCGGCACGCCGCAACATTTAAA 4551

N1.27 GCGGCTCACAATCATGGTCAACGGACAGTTCCAGTGCCTCGGCACGCCGCAACATTTAAA 4547

A1.2 GCGGCTCACAATCATGGTCAACGGACAGTTCCAGTGCCTCGGCACGCCGCAACATTTAAA 4615

A1.7 GCGGCTCACAATCATGGTCAACGGACAGTTCCAGTGCCTCGGCACGCCGCAACATTTAAA 4615

N3.9 GCGGCTCACAATCATGGTCAACGGACAGTTCCAGTGCCTCGGCACGCCGCAACATTTAAA 4868

************************************************************

MG637361.1 GAATAAGTTCTCTGAAGGTTTCACATTGACAATTAAAATTAAAGTGGACGACGAGACGAA 4928

A1.9 GAATAAGTTCTCTGAAGGTTTCACATTGACAATTAAAATTAAAGTGGACGACGAGACGAA 3935

N1.8 GAATAAGTTCTCTGAAGGTTTCACATTGACAATTAAAATTAAAGTGGACGACGAGACGAA 5075

A1.4 GAATAAGTTCTCTGAAGGTTTCACATTGACAATTAAAATTAAAGTGGACGACGAGACGAA 4756

A3.1 GAATAAGTTCTCTGAAGGTTTCACATTGACAATTAAAATTAAAGTGGACGACGAGACGAA 5219

A3.4 GAATAAGTTCTCTGAAGGTTTCACATTGACAATTAAAATTAAAGTGGACGACGAGACGAA 5219

A3.6 GAATAAGTTCTCTGAAGGTTTCACATTGACAATTAAAATTAAAGTGGACGACGAGACGAA 5219

A3.7 GAATAAGTTCTCTGAAGGTTTCACATTGACAATTAAAATTAAAGTGGACGACGAGACGAA 5219

A3.8 GAATAAGTTCTCTGAAGGTTTCACATTGACAATTAAAATTAAAGTGGACGACGAGACGAA 5219

N3.1 GAATAAGTTCTCTGAAGGTTTCACATTGACAATTAAAATTAAAGTGGACGACGAGACGAA 4651

N3.6 GAATAAGTTCTCTGAAGGTTTCACATTGACAATTAAAATTAAAGTGGACGACGAGACGAA 4651

N3.3 GAATAAGTTCTCTGAAGGTTTCACATTGACAATTAAAATTAAAGTGGACGACGAGACGAA 4651

N3.10 GAATAAGTTCTCTGAAGGTTTCACATTGACAATTAAAATTAAAGTGGACGACGAGACGAA 4651

A1.3 GAATAAGTTCTCTGAAGGTTTCACATTGACAATTAAAATTAAAGTGGACGACGAGACGAA 4506

N1.2 GAATAAGTTCTCTGAAGGTTTCACATTGACAATTAAAATTAAAGTGGACGACGAGACGAA 4611

N1.5 GAATAAGTTCTCTGAAGGTTTCACATTGACAATTAAAATTAAAGTGGACGACGAGACGAA 4611

N1.27 GAATAAGTTCTCTGAAGGTTTCACATTGACAATTAAAATTAAAGTGGACGACGAGACGAA 4607

A1.2 GAATAAGTTCTCTGAAGGTTTCACATTGACAATTAAAATTAAAGTGGACGACGAGACGAA 4675

A1.7 GAATAAGTTCTCTGAAGGTTTCACATTGACAATTAAAATTAAAGTGGACGACGAGACGAA 4675

N3.9 GAATAAGTTCTCTGAAGGTTTCACATTGACAATTAAAATTAAAGTGGACGACGAGACGAA 4928

************************************************************

MG637361.1 GACTGTACGGCCTGAAGTCTGCGATGCTGTGAAGCATTACGTCAGTACCAACTTCAGAGA 4988

A1.9 GACTGTACGGCCTGAAGTCTGCGATGCTGTGAAGCATTACGTCAGTACCAACTTCAGAGA 3995

N1.8 GACTGTACGGCCTGAAGTCTGCGATGCTGTGAAGCATTACGTCAGTACCAACTTCAGAGA 5135

A1.4 GACTGTACGGCCTGAAGTCTGCGATGCTGTGAAGCATTACGTCAGTACCAACTTCAGAGA 4816

A3.1 GACTGTACGGCCTGAAGTCTGCGATGCTGTGAAGCATTACGTCAGTACCAACTTCAGAGA 5279

A3.4 GACTGTACGGCCTGAAGTCTGCGATGCTGTGAAGCATTACGTCAGTACCAACTTCAGAGA 5279

A3.6 GACTGTACGGCCTGAAGTCTGCGATGCTGTGAAGCATTACGTCAGTACCAACTTCAGAGA 5279

A3.7 GACTGTACGGCCTGAAGTCTGCGATGCTGTGAAGCATTACGTCAGTACCAACTTCAGAGA 5279

A3.8 GACTGTACGGCCTGAAGTCTGCGATGCTGTGAAGCATTACGTCAGTACCAACTTCAGAGA 5279

N3.1 GACTGTACGGCCTGAAGTCTGCGATGCTGTGAAGCATTACGTCAGTACCAACTTCAGAGA 4711

N3.6 GACTGTACGGCCTGAAGTCTGCGATGCTGTGAAGCATTACGTCAGTACCAACTTCAGAGA 4711

N3.3 GACTGTACGGCCTGAAGTCTGCGATGCTGTGAAGCATTACGTCAGTACCAACTTCAGAGA 4711

N3.10 GACTGTACGGCCTGAAGTCTGCGATGCTGTGAAGCATTACGTCAGTACCAACTTCAGAGA 4711

A1.3 GACTGTACGGCCTGAAGTCTGCGATGCTGTGAAGCATTACGTCAGTACCAACTTCAGAGA 4566

N1.2 GACTGTACGGCCTGAAGTCTGCGATGCTGTGAAGCATTACGTCAGTACCAACTTCAGAGA 4671

N1.5 GACTGTACGGCCTGAAGTCTGCGATGCTGTGAAGCATTACGTCAGTACCAACTTCAGAGA 4671

N1.27 GACTGTACGGCCTGAAGTCTGCGATGCTGTGAAGCATTACGTCAGTACCAACTTCAGAGA 4667

A1.2 GACTGTACGGCCTGAAGTCTGCGATGCTGTGAAGCATTACGTCAGTACCAACTTCAGAGA 4735

A1.7 GACTGTACGGCCTGAAGTCTGCGATGCTGTGAAGCATTACGTCAGTACCAACTTCAGAGA 4735

N3.9 GACTGTACGGCCTGAAGTCTGCGATGCTGTGAAGCATTACGTCAGTACCAACTTCAGAGA 4988

************************************************************

MG637361.1 GCCGAAGATTATGGAGGAGTACCAGGGTCTGTTAACATACTATTTGCCAGACAAGTCGGT 5048

A1.9 GCCGAAGATTATGGAGGAGTACCAGGGTCTGTTAACATACTATTTGCCAGACAAGTCGGT 4055

N1.8 GCCGAAGATTATGGAGGAGTACCAGGGTCTGTTAACATACTATTTGCCAGACAAGTCGGT 5195

A1.4 GCCGAAGATTATGGAGGAGTACCAGGGTCTGTTAACATACTATTTGCCAGACAAGTCGGT 4876

A3.1 GCCGAAGATTATGGAGGAGTACCAGGGTCTGTTAACATACTATTTGCCAGACAAGTCGGT 5339

A3.4 GCCGAAGATTATGGAGGAGTACCAGGGTCTGTTAACATACTATTTGCCAGACAAGTCGGT 5339

A3.6 GCCGAAGATTATGGAGGAGTACCAGGGTCTGTTAACATACTATTTGCCAGACAAGTCGGT 5339

A3.7 GCCGAAGATTATGGAGGAGTACCAGGGTCTGTTAACATACTATTTGCCAGACAAGTCGGT 5339

A3.8 GCCGAAGATTATGGAGGAGTACCAGGGTCTGTTAACATACTATTTGCCAGACAAGTCGGT 5339

N3.1 GCCGAAGATTATGGAGGAGTACCAGGGTCTGTTAACATACTATTTGCCAGACAAGTCGGT 4771

N3.6 GCCGAAGATTATGGAGGAGTACCAGGGTCTGTTAACATACTATTTGCCAGACAAGTCGGT 4771

N3.3 GCCGAAGATTATGGAGGAGTACCAGGGTCTGTTAACATACTATTTGCCAGACAAGTCGGT 4771

N3.10 GCCGAAGATTATGGAGGAGTACCAGGGTCTGTTAACATACTATTTGCCAGACAAGTCGGT 4771

A1.3 GCCGAAGATTATGGAGGAGTACCAGGGTCTGTTAACATACTATTTGCCAGACAAGTCGGT 4626

N1.2 GCCGAAGATTATGGAGGAGTACCAGGGTCTGTTAACATACTATTTGCCAGACAAGTCGGT 4731

N1.5 GCCGAAGATTATGGAGGAGTACCAGGGTCTGTTAACATACTATTTGCCAGACAAGTCGGT 4731

N1.27 GCCGAAGATTATGGAGGAGTACCAGGGTCTGTTAACATACTATTTGCCAGACAAGTCGGT 4727

A1.2 GCCGAAGATTATGGAGGAGTACCAGGGTCTGTTAACATACTATTTGCCAGACAAGTCGGT 4795

A1.7 GCCGAAGATTATGGAGGAGTACCAGGGTCTGTTAACATACTATTTGCCAGACAAGTCGGT 4795

N3.9 GCCGAAGATTATGGAGGAGTACCAGGGTCTGTTAACATACTATTTGCCAGACAAGTCGGT 5048

************************************************************

MG637361.1 GGCGTGGTCCAGAATGTTCGGCATAATGGAGGCGGCCAAACGCGACCTCCCCGTCGAAGA 5108

A1.9 GGCGTGGTCCAGAATGTTCGGCATAATGGAGGCGGCCAAACGCGACCTCCCCGTCGAAGA 4115

N1.8 GGCGTGGTCCAGAATGTTCGGCATAATGGAGGCGGCCAAACGCGACCTCCCCGTCGAAGA 5255

A1.4 GGCGTGGTCCAGAATGTTCGGCATAATGGAGGCGGCCAAACGCGACCTCCCCGTCGAAGA 4936

A3.1 GGCGTGGTCCAGAATGTTCGGCATAATGGAGGCGGCCAAACGCGACCTCCCCGTCGAAGA 5399

A3.4 GGCGTGGTCCAGAATGTTCGGCATAATGGAGGCGGCCAAACGCGACCTCCCCGTCGAAGA 5399

A3.6 GGCGTGGTCCAGAATGTTCGGCATAATGGAGGCGGCCAAACGCGACCTCCCCGTCGAAGA 5399

A3.7 GGCGTGGTCCAGAATGTTCGGCATAATGGAGGCGGCCAAACGCGACCTCCCCGTCGAAGA 5399

A3.8 GGCGTGGTCCAGAATGTTCGGCATAATGGAGGCGGCCAAACGCGACCTCCCCGTCGAAGA 5399

N3.1 GGCGTGGTCCAGAATGTTCGGCATAATGGAGGCGGCCAAACGCGACCTCCCCGTCGAAGA 4831

N3.6 GGCGTGGTCCAGAATGTTCGGCATAATGGAGGCGGCCAAACGCGACCTCCCCGTCGAAGA 4831

N3.3 GGCGTGGTCCAGAATGTTCGGCATAATGGAGGCGGCCAAACGCGACCTCCCCGTCGAAGA 4831

N3.10 GGCGTGGTCCAGAATGTTCGGCATAATGGAGGCGGCCAAACGCGACCTCCCCGTCGAAGA 4831

A1.3 GGCGTGGTCCAGAATGTTCGGCATAATGGAGGCGGCCAAACGCGACCTCCCCGTCGAAGA 4686

N1.2 GGCGTGGTCCAGAATGTTCGGCATAATGGAGGCGGCCAAACGCGACCTCCCCGTCGAAGA 4791

N1.5 GGCGTGGTCCAGAATGTTCGGCATAATGGAGGCGGCCAAACGCGACCTCCCCGTCGAAGA 4791

N1.27 GGCGTGGTCCAGAATGTTCGGCATAATGGAGGCGGCCAAACGCGACCTCCCCGTCGAAGA 4787

A1.2 GGCGTGGTCCAGAATGTTCGGCATAATGGAGGCGGCCAAACGCGACCTCCCCGTCGAAGA 4855

A1.7 GGCGTGGTCCAGAATGTTCGGCATAATGGAGGCGGCCAAACGCGACCTCCCCGTCGAAGA 4855

N3.9 GGCGTGGTCCAGAATGTTCGGCATAATGGAGGCGGCCAAACGCGACCTCCCCGTCGAAGA 5108

************************************************************

MG637361.1 CTACAGCATATCACAAACTACCCTCGAGCAGATATTCCTACAGTTCACAAAGTATCAACA 5168

A1.9 CTACAGCATATCACAAACTACCCTCGAG-------------------------------- 4143

N1.8 CTACAGCATATCACAAACTACCCTCGAG-------------------------------- 5283

A1.4 CTACAGCATATCACAAACTACCCTCGAG-------------------------------- 4964

A3.1 CTACAGCATATCACAAACTACCCTCGAG-------------------------------- 5427

A3.4 CTACAGCATATCACAAACTACCCTCGAG-------------------------------- 5427

A3.6 CTACAGCATATCACAAACTACCCTCGAG-------------------------------- 5427

A3.7 CTACAGCATATCACAAACTACCCTCGAG-------------------------------- 5427

A3.8 CTACAGCATATCACAAACTACCCTCGAG-------------------------------- 5427

N3.1 CTACAGCATATCACAAACTACCCTCGAG-------------------------------- 4859

N3.6 CTACAGCATATCACAAACTACCCTCGAG-------------------------------- 4859

N3.3 CTACAGCATATCACAAACTACCCTCGAG-------------------------------- 4859

N3.10 CTACAGCATATCACAAACTACCCTCGAG-------------------------------- 4859

A1.3 CTACAGCATATCACAAACTACCCTCGAG-------------------------------- 4714

N1.2 CTACAGCATATCACAAACTACCCTCGAG-------------------------------- 4819

N1.5 CTACAGCATATCACAAACTACCCTCGAG-------------------------------- 4819

N1.27 CTACAGCATATCACAAACTACCCTCGAG-------------------------------- 4815

A1.2 CTACAGCATATCACAAACTACCCTCGAG-------------------------------- 4882

A1.7 CTACAGCATATCACAAACTACCCTCGAG-------------------------------- 4882

N3.9 CTACAGCATATCACAAACTACCCTCGAG-------------------------------- 5136

****************************

MG637361.1 TGAAGCACAACAGACATAA 5187

A1.9 ------------------- 4143

N1.8 ------------------- 5283

A1.4 ------------------- 4964

A3.1 ------------------- 5427

A3.4 ------------------- 5427

A3.6 ------------------- 5427

A3.7 ------------------- 5427

A3.8 ------------------- 5427

N3.1 ------------------- 4859

N3.6 ------------------- 4859

N3.3 ------------------- 4859

N3.10 ------------------- 4859

A1.3 ------------------- 4714

N1.2 ------------------- 4819

N1.5 ------------------- 4819

N1.27 ------------------- 4815

A1.2 ------------------- 4882

A1.7 ------------------- 4882

N3.9 ------------------- 5136
