# Supplementary material for: Shared and Independent Genetic Basis of Resistance to Bt Toxin Cry2Ab in Two Strains of Pink Bollworm
Source: Sci Rep. 2020 May 14;10:7988. doi: 10.1038/s41598-020-64811-w (PMC7224296; doi:10.1038/s41598-020-64811-w)
Supplement: Supplementary file 5 — Supplementary table S1. [file 41598_2020_64811_MOESM5_ESM.docx]

**Supplementary Table S1. BX-R *PgABCA2* transcripts and cDNA mutations.**

| **Larva** | **Clone** | **cDNA (bp)** | **% Identity^a^** | **cDNA Mutation** | **Codon^b^** | **Exon^c^** | **Type^d^** | **Effect^e^** |
| --- | --- | --- | --- | --- | --- | --- | --- | --- |
| BX-R #1 | 5 | 5,074 | 98.3 | c.1786_1843del  c.2030A>T  c.2753G>C  c.2969T>C  c.2975C>T  c.3097_3100del  c.3163C>A  c.3517G>A  c.3820A>G | 596  677  918  990  992  1033  1055  1173  1274 | 10  11  16  17  17  18  18  20  22 | fs  ms  ms  ms  ms  fs  ms  ms  ms | stop at 597  E677V  C918S  L990S  P992L  stop at 1027  H1055N  V1173I  S1274G |
| BX-R #1 | 6 | 5,009 | 97.1 | c.1905_2031del  c.2753G>C  c.2969T>C  c.2975C>T  c.3163C>A  c.3517G>A  c.3820A>G | 635  918  990  992  1055  1173  1274 | 11  16  17  17  18  20  22 | fs  ms  ms  ms  ms  ms  ms | stop at 662  C918S  L990S  P992L  H1055N  V1173I  S1274G |
| BX-R #1 | 40 | 5,135 | 99.5 | c.1963del  c.2030A>T  c.2753G>C  c.2969T>C  c.2975C>T  c.3163C>A  c.3517G>A  c.3820A>G | 655  677  918  990  992  1055  1173  1274 | 11  11  16  17  17  18  20  22 | fs  ms  ms  ms  ms  ms  ms  ms | stop at 664  E677V  C918S  L990S  P992L  H1055N  V1173I  S1274G |
| BX-R #2 | 12 | 5,136 | 99.5 | c.2030A>T  c.2753G>C  c.2969T>C  c.2975C>T  c.3163C>A  c.3517G>A  c.3820A>G | 677  918  990  992  1055  1173  1274 | 11  16  17  17  18  20  22 | ms  ms  ms  ms  ms  ms  ms | E677V  C918S  L990S  P992L  H1055N  V1173I  S1274G |
| BX-R #2 | 42 | 4,985 | 96.6 | c.1090_1234del  c.1622_1623del  c.2030A>T  c.2753G>C  c.2969T>C  c.2975C>T  c.3097_3100del  c.3163C>A  c.3517G>A  c.3820A>G | 364  541  677  918  990  992  1033  1055  1173  1274 | 6  9  11  16  17  17  18  18  20  22 | fs  fs  ms  ms  ms  ms  fs  ms  ms  ms | stop at 373  stop at 517  E677V  C918S  L990S  P992L  stop at 997  H1055N  V1173I  S1274G |
| BX-R #2 | 52 | 4,985 | 96.6 | c.1090_1234del  c.1622_1623del  c.2030A>T  c.2753G>C  c.2969T>C  c.2975C>T  c.3097_3100del  c.3163C>A  c.3517G>A  c.3820A>G | 364  541  677  918  990  992  1033  1055  1173  1274 | 6  9  11  16  17  17  18  18  20  22 | fs  fs  ms  ms  ms  ms  fs  ms  ms  ms | stop at 373  stop at 517  E677V  C918S  L990S  P992L  stop at 997  H1055N  V1173I  S1274G |
| BX-R #5 | 8 | 5,135 | 99.5 | c.1609del  c.2030A>T  c.2753G>C  c.2969T>C  c.2975C>T  c.3163C>A  c.3517G>A  c.3820A>G | 537  677  918  990  992  1055  1173  1274 | 8  11  16  17  17  18  20  22 | fs  ms  ms  ms  ms  ms  ms  ms | stop at 552  E677V  C918S  L990S  P992L  H1055N  V1173I  S1274G |
| BX-R #5 | 9 | 5,117 | 99.1 | c.1832_1850del  c.2030A>T  c.2753G>C  c.2969T>C  c.2975C>T  c.3163C>A  c.3517G>A  c.3820A>G | 611  677  918  990  992  1055  1173  1274 | 10  11  16  17  17  18  20  22 | fs  ms  ms  ms  ms  ms  ms  ms | stop at 615  E677V  C918S  L990S  P992L  H1055N  V1173I  S1274G |
| BX-R #5 | 11 | 4,991 | 96.7 | c.1090_1234del  c.2030A>T  c.2753G>C  c.2969T>C  c.2975C>T  c.3163C>A  c.3517G>A  c.3820A>G | 364  677  918  990  992  1055  1173  1274 | 6  11  16  17  17  18  20  22 | fs  ms  ms  ms  ms  ms  ms  ms | stop at 373  E677V  C918S  L990S  P992L  H1055N  V1173I  S1274G |

^a^ Sequence identity calculated from paired sequence alignments between the cloned BX-R cDNA sequences and the full-length wildtype *PgABCA2* cDNA (MG637361.1).

^b^ Codon where the mutation occurs in the full-length *PgABCA2* cDNA sequence (MG637361.1).

^c^ Exon where the mutation occurs.

^d^ fs, frameshift caused by deletion; ms, missense (point mutation, single amino acid substitution caused by single bp change).

^e^ Position of amino acid substitution or premature stop codon.
