# Supplementary material for: Shared and Independent Genetic Basis of Resistance to Bt Toxin Cry2Ab in Two Strains of Pink Bollworm
Source: Sci Rep. 2020 May 14;10:7988. doi: 10.1038/s41598-020-64811-w (PMC7224296; doi:10.1038/s41598-020-64811-w)
Supplement: Supplementary file 6 — Supplementary table S2. [file 41598_2020_64811_MOESM6_ESM.docx]

**Supplementary Table S2. BX-R *PgABCA2* gDNA fragments corresponding to cDNA mutations.**

| **Larva** | **Primer Pair^a^** | **Exon^b^** | **cDNA Mutation^c^** | **In gDNA^d^** | **Source of mutation** |
| --- | --- | --- | --- | --- | --- |
| BX-R #1 | 68PgABCA2-5 + 141PgABCA2-3  126PgABCA2-5 + 127PgABCA2-3  86PgABC5 + 87PgABC3  124PgABCA2-5 + 154PgABCA2-3  85PgABC5 +  88PgABC3  rA1-F + 82PgABCA2-3  89PgABC5 + 90PgABC3 | 10-12  14-16  16-17  17-18  18  20-21  22-23 | c.1786_1843del  c.1905_2031del  c.1963del  c.2030A>T  c.2753G>C  c.2969T>C  c.2975C>T  c.3097_3100del  c.3163C>A  c.3517G>A  c.3820A>G | no  no  no  yes  yes  yes  yes  no  yes  yes  yes | mRNA  mRNA  mRNA  gDNA  gDNA  gDNA  gDNA  mRNA  gDNA  gDNA  gDNA |
| BX-R #2 | 143PgABCA2-5 + 90PgABCA2-3  186PgABCA2-5 + 185PgABCA2-3  68PgABCA2-5 + 141PgABCA2-3  126PgABCA2-5 + 127PgABCA2-3  86PgABC5 + 87PgABC3  124PgABCA2-5 + 154PgABCA2-3  85PgABC5 +  88PgABC3  rA1-F + 82PgABCA2-3  89PgABC5 + 90PgABC3 | 5-7  8-9  10-12  14-16  16-17  17-18  18  20-21  22-23 | c.1090_1234del  c.1622_1623del  c.2030A>T  c.2753G>C  c.2969T>C  c.2975C>T  c.3097_3100del  c.3163C>A  c.3517G>A  c.3820A>G | no  no  yes  yes  yes  yes  no  yes  yes  yes | mRNA, exon 6 mis-splice  mRNA  gDNA  gDNA  gDNA  gDNA  mRNA  gDNA  gDNA  gDNA |
| BX-R #5 | 143PgABCA2-5 + 90PgABCA2-3  186PgABCA2-5 + 185PgABCA2-3  68PgABCA2-5 + 141PgABCA2-3  126PgABCA2-5 + 127PgABCA2-3  86PgABC5 + 87PgABC3  85PgABC5 +  88PgABC3  rA1-F + 82PgABCA2-3  89PgABC5 + 90PgABC3 | 5-7  8-9  10-12  14-16  16-17  18  20-21  22-23 | c.1090_1234del  c.1609del  c.1832_1850del  c.2030A>T  c.2753G>C  c.2969T>C  c.2975C>T  c.3163C>A  c.3517G>A  c.3820A>G | no  no  no  yes  yes  yes  yes  yes  yes  yes | mRNA, exon 6 mis-splice  mRNA  mRNA  gDNA  gDNA  gDNA  gDNA  gDNA  gDNA  gDNA |

^a^ Oligonucleotide primer pairs used to PCR amplify gDNA fragments corresponding to cDNA mutations for cloning and Sanger sequencing.

^b^ Exons where primers used to PCR amplify gDNA.

^c^ cDNA mutation from Table 1.

^d^ Indicates if cDNA mutation also occurs in corresponding gDNA.
