# Supplementary material for: Shared and Independent Genetic Basis of Resistance to Bt Toxin Cry2Ab in Two Strains of Pink Bollworm
Source: Sci Rep. 2020 May 14;10:7988. doi: 10.1038/s41598-020-64811-w (PMC7224296; doi:10.1038/s41598-020-64811-w)
Supplement: Supplementary file 7 — Supplementary table S3. [file 41598_2020_64811_MOESM7_ESM.docx]

**Supplementary Table S3. *PgABCA2* transcripts and cDNA mutations from BX-R X Bt4-R2 F_1_ larvae.**

| **Larva^a^** | **Clone** | **cDNA (bp)** | **% Identity^a^** | **cDNA Mutation** | **Codon^b^** | **Exon^c^** | **Type^d^** | **Effect^e^** |
| --- | --- | --- | --- | --- | --- | --- | --- | --- |
| A1 | 2 | 4,882 | 94.6 | c.101C>T  c.2030A>T  c.2753G>C  c.2969T>C  c.2975C>T  c.3163C>A  c.3517G>A  c.3590_3715del  c.3820A>G  c.3840_3967del  c.3972A>T | 34  677  918  990  992  1055  1173  1197  1274  1280  1325 | 1  11  16  17  17  18  20  21  22  23  24 | ms  ms  ms  ms  ms  ms  ms  InF  ms  fs  ns | T34M  E677V  C918S  L990S  P992L  H1055N  V1173I  loss of 42 aa  S1274G  stop at 1291  stop at 1240 |
| A1 | 3 | 4,714 | 91.8 | c.728A>T  c.1090_1234del  c.3313_3589del | 243  364  1105 | 3  6  19-20 | ms  fs  fs | D243V  stop at 373  stop at 1063 |
| A1 | 4 | 4,964 | 96.5 | c.2030A>T  c.3418_3589del | 677  1140 | 11  20 | ms  fs | E677V  stop at 1147 |
| A1 | 7 | 4,882 | 94.6 | c.101C>T  c.2030A>T  c.2753G>C  c.2969T>C  c.2975C>T  c.3163C>A  c.3517G>A  c.3590_3715del  c.3820A>G  c.3840_3967del  c.3972A>T | 34  677  918  990  992  1055  1173  1197  1274  1280  1325 | 1  11  16  17  17  18  20  21  22  23  24 | ms  ms  ms  ms  ms  ms  ms  InF  ms  fs  ns | T34M  E677V  C918S  L990S  P992L  H1055N  V1173I  loss of 42 aa  S1274G  stop at 1291  stop at 1240 |
| A1 | 9 | 4,143 | 77.4 | c.101C>T  c.2030A>T  c.2230_2930del  c.3313_3967delins^f^ | 34  677  744  1106 | 1  11  13-16  19-23 | ms  ms  fs  fs | T34M  E677V  stop at 751  stop at 897 |
| A3 | 1 | 5,427 | 94.5 | c.3556_3588delins^f^ | 1186 | 20 | fs | stop at 1220 |
| A3 | 4 | 5,427 | 94.5 | c.3556_3588delins^f^ | 1186 | 20 | fs | stop at 1220 |
| A3 | 6 | 5,427 | 94.5 | c.3556_3588delins^f^ | 1186 | 20 | fs | stop at 1220 |
| A3 | 7 | 5,427 | 94.5 | c.3556_3588delins^f^ | 1186 | 20 | fs | stop at 1220 |
| A3 | 8 | 5,427 | 94.5 | c.3556_3588delins^f^ | 1186 | 20 | fs | stop at 1220 |
| N1 | 2 | 4,819 | 93.8 | c.1090_1234del  c.3418_3589del | 364  1140 | 6  20 | fs  fs | stop at 373  stop at 1098 |
| N1 | 5 | 4,819 | 93.8 | c.1090_1234del  c.3418_3589del | 364  1140 | 6  20 | fs  fs | stop at 373  stop at 1098 |
| N1 | 8 | 5,283 | 91.7 | c.432_433insT  c.1090_1234del  c.3556_3588delins^f^ | 144  364  1186 | 2  6  20 | fs  fs  fs | stop at 145  stop at 373  stop at 1172 |
| N1 | 27 | 4,815 | 93.8 | c.1090_1234del  c.3097_3100del  c.3418_3589del | 364  1033  1140 | 6  18  20 | fs  fs  fs | stop at 373  stop at 997  stop at 1097 |
| N3 | 1 | 4,859 | 94.6 | c.3313_3589del | 1105 | 19-20 | fs | stop at 1112 |
| N3 | 3 | 4,859 | 94.6 | c.3313_3589del | 1105 | 19-20 | fs | stop at 1112 |
| N3 | 6 | 4,859 | 94.6 | c.3313_3589del | 1105 | 19-20 | fs | stop at 1112 |
| N3 | 9 | 5,136 | 99.5 | c.1454C>A  c.1702C>A  c.2030A>T  c.2753G>C  c.2969T>C  c.2975C>T  c.3163C>A  c.3517G>A  c.3820A>G | 485  568  677  918  990  992  1055  1173  1274 | 8  9  11  16  17  17  18  20  22 | ns  ms  ms  ms  ms  ms  ms  ms  ms | stop at 485  Q568K  E677V  C918S  L990S  P992L  H1055N  V1173I  S1274G |
| N3 | 10 | 4,859 | 94.6 | c.3313_3589del | 1105 | 19-20 | fs | stop at 1112 |

^a^ Sequence identity calculated from paired sequence alignments between the cloned BX-R X Bt4-R2 cDNA sequences and the full-length wildtype *PgABCA2* cDNA (MG637361.1).

^b^ Codon where the mutation occurs in the full-length *PgABCA2* cDNA sequence (MG637361.1).

^c^ Exon where the mutation occurs.

^d^ ms, missense (point mutation, single amino acid substitution caused by single bp change); InF, in-frame deletion; fs, frameshift caused by deletion; ns, nonsense mutation (single base-pair substitution that introduces premature stop codon).

^e^ Position of amino acid substitution, amino acids lost, or premature stop codon.

^f^ Sequence corresponding to insertion not shown due to large number of nucleotide bases.
