# Supplementary material for: Shared and Independent Genetic Basis of Resistance to Bt Toxin Cry2Ab in Two Strains of Pink Bollworm
Source: Sci Rep. 2020 May 14;10:7988. doi: 10.1038/s41598-020-64811-w (PMC7224296; doi:10.1038/s41598-020-64811-w)
Supplement: Supplementary file 8 — Supplementary table S4. [file 41598_2020_64811_MOESM8_ESM.docx]

**Supplementary Table S4. Nucleotide primers used to amplify, genotype, and/or DNA sequence *PgABCA2*.**

| **Primer** | **Sequence** | **Direction** | **Application** |
| --- | --- | --- | --- |
| M13 reverse | 5' - CAGGAAACAGCTATGAC - 3' | - | Vector primer for DNA sequencing |
| T7 | 5' - TAATACGACTCACTATAGGG - 3' | - | Vector primer for DNA sequencing |
| r_A1_-F (83PgABCA2-5) | 5' - CGCCAAGCTGCTGCAGAAG - 3' | Sense | Allele specific PCR & PCR amplification of gDNA |
| r_A1_-R (84PgABCA2-3) | 5' - TGAGTCGTCCAACTAATCCTGACTGATAACAT - 3' | Antisense | Allele specific PCR |
| r_A1_-F2 (85PgABCA2-5) | 5' - CTCTTCTGTCGATGAACTGGGTAATTATT - 3' | Sense | Allele specific PCR |
| 163PgABCA2-5 | 5' - GAATTTGGATTTGAGGCG - 3' | Sense | 5'-UTR primer for amplifying & cloning near full-length cDNA |
| 166PgABCA2-3 | 5' - TCGAGGGTAGTTTGTGAT - 3' | Antisense | Exon 31 primer for amplification & cloning near full-length cDNA |
| 63PgABCA2-3 | 5' - CGCCTGTCATGAAGAAGGA - 3' | Antisense | DNA sequencing cDNA clones |
| 64PgABCA2-5 | 5' - ATTGCGTGAAATAACGGAGC - 3' | Sense | DNA sequencing cDNA clones |
| 65PgABCA2-3 | 5' - CCGCTGTACTGGCTTTTGA - 3' | Antisense | DNA sequencing cDNA clones |
| 66PgABCA2-5 | 5' - ACCTTGGACCGTCCTCTTCT - 3' | Sense | DNA sequencing cDNA clones |
| 67PgABCA2-3 | 5' - GTCCACGTTACCTGTGAGCA - 3' | Antisense | DNA sequencing cDNA clones |
| 68PgABCA2-5 | 5' - TTTACGACGACCAAATCACG - 3' | Sense | DNA sequencing cDNA clones & PCR amplification of gDNA |
| 69PgABCA2-3 | 5' - TCACAACAGTTCCAGGGATG - 3' | Antisense | DNA sequencing cDNA clones |
| 70PgABCA2-5 | 5' - GGAGTCGGCTACACGCTAGT - 3' | Sense | DNA sequencing cDNA clones |
| 71PgABCA2-3 | 5' - TCCATTGTGGGATTATTGGC - 3' | Antisense | DNA sequencing cDNA clones |
| 72PgABCA2-5 | 5' - GTTTCAAAGGGTTGTCCCCT - 3' | Sense | DNA sequencing cDNA clones |
| 73PgABCA2-3 | 5' - CCTATCGGTAGACTGGCGG - 3' | Antisense | DNA sequencing cDNA clones |
| 74PgABCA2-5 | 5' - AACGTCATTGGGCTCTCTTC - 3' | Sense | DNA sequencing cDNA clones |
| 75PgABCA2-3 | 5' - CCATAATACTTGGTGAGGCCA - 3' | Antisense | DNA sequencing cDNA clones |
| 76PgABCA2-5 | 5' - CACAATGTTGCATCCCAGAC - 3' | Sense | DNA sequencing cDNA clones |
| 77PgABCA2-5 | 5' - GCAATTCGAAGCGACATTC - 3' | Sense | DNA sequencing cDNA clones |
| 78PgABCA2-5 | 5' - CGATGCTGTGAAGCATTACG - 3' | Sense | DNA sequencing cDNA clones |
| 143PgABCA2-5 | 5' - ACTCCCAACGTGGCTGCAT - 3' | Sense | PCR amplification of gDNA |
| 90PgABCA2-3 | 5' - CATAGCCAGGAGGAATGCG - 3' | Antisense | PCR amplification of gDNA |
| 186PgABCA2-5 | 5' - CGGTCACGTCGTTATAATGATGG - 3' | Sense | PCR amplification of gDNA |
| 185PgABCA2-3 | 5' - AAGGTCTGTAGGGTCGCTTTC - 3' | Antisense | PCR amplification of gDNA |
| 141PgABCA2-3 | 5' - CTGATAACCCCGCTGATTG - 3' | Antisense | PCR amplification of gDNA |
| 124PgABCA2-5 | 5’ - TTCGCTAGCGAAGGCTGCCT - 3’ | Sense | PCR amplification of gDNA |
| 154PgABCA2-3 | 5’ - GTCGAACGTCGCGCCGAT - 3’ | Antisense | PCR amplification of gDNA |
| 126PgABCA2-5 | 5' - TGACATATAGCATGACTAATGAGTA - 3' | Sense | PCR amplification of gDNA |
| 127PgABCA2-3 | 5' - CGAGTGTGGCAATGATTTGTA - 3' | Antisense | PCR amplification of gDNA |
| 86PgABC5 | 5' - GGCTTATCCTGTTGCTCCAA - 3' | Sense | PCR amplification of gDNA |
| 87PgABC3 | 5' - GGGATTATTGGCGGTTACAA - 3' | Antisense | PCR amplification of gDNA |
| 85PgABC5 | 5' - AGATGACGTATCAGCGATGG - 3' | Sense | PCR amplification of gDNA |
| 88PgABC3 | 5' - AGTCAAGTTGGCTGCAGGAT - 3' | Antisense | PCR amplification of gDNA |
| 82PgABCA2-3 | 5' - GCGCCATACACTATGATGCACAAGTAC - 3' | Antisense | PCR amplification of gDNA |
| 89PgABC5 | 5' - CACCGCAGCTTGATACTGAA - 3' | Sense | PCR amplification of gDNA |
| 90PgABC3 | 5' - TGTGAGAACGTCTGGCACAT - 3' | Antisense | PCR amplification of gDNA |
|  |  |  |  |
|  |  |  |  |
|  |  |  |  |
|  |  |  |  |
|  |  |  |  |
|  |  |  |  |
